# Supplementary material for: Efficient room temperature catalytic synthesis of alternating conjugated copolymers via C-S bond activation
Source: Nat Commun. 2022 Jan 10;13:144. doi: 10.1038/s41467-021-27832-1 (PMC8748944; doi:10.1038/s41467-021-27832-1)
Supplement: Supplementary file 1 — Supplementary Information [file 41467_2021_27832_MOESM1_ESM.pdf]

Supplementary Information

for

Efficient Room Temperature Catalytic Synthesis of Alternating Conjugated Copolymers via C-S Bond Activation

Zijie Li,[1,2] Qinqin Shi,[1*] Xiaoying Ma,[3] Yawen Li,[4] Kaikai Wen,[1] Linqing Qin,[1] Hao Chen,[1] Wei Huang,[5] Fengjiao Zhang,[3] Yuze Lin,[4] Tobin J. Marks,[5*] Hui Huang[1,2,6,7*]

[1] College of Materials Science and Opto-Electronic Technology, University of Chinese Academy of Sciences, Beijing 100049, P. R. China.

[2] Center of Materials Science and Optoelectronics Engineering, University of Chinese Academy of Sciences, Beijing 100049, P. R. China.

[3] School of Chemical Sciences, University of Chinese Academy of Sciences, Beijing 100049, P. R. China

[4] Beijing National Laboratory for Molecular Sciences, CAS Key Laboratory of Organic Solids, Institute of Chemistry, Chinese Academy of Sciences, Beijing 100190, P. R. China

[5] Department of Chemistry and the Materials Research Center, Northwestern University, 2145 Sheridan Road, Evanston, Illinois 60208, United States

[6] CAS Center for Excellence in Topological Quantum Computation, University of Chinese Academy of Sciences, Beijing 100049, P. R. China.

[7] CAS Key Laboratory of Vacuum Physic, University of Chinese Academy of Sciences, Beijing 100049, P. R. China.

Supplementary Methods

I. Measurements and characterization S3
II. Materials and synthesis S4
II-1. Materials S4
II-2. Preparation and characterization S6
II-3. Model reactions. S19
II-4. Optimization on the polymerization of P1 .... S23
II-5. Classic Stille coupling S25
II-6. Substrates scope on polymerization S30
III. Mechanistic studies S37
III-1. Competition reactions S38
III-2. Control experiments S39
III-3. Defect analysis S40
IV. Characterization S42
IV-1 Optical and electrochemical properties S42
IV-2. Thermal properties S44
IV-3. Trap studies on P2-CS and P2-CI S45
IV-4. Applications of high quality materials based on P2-CS, P4, and P5. .... S48
V. References S54
VI. NMR spectra and GPC traces S57

Supplementary Methods

I. Measurements and characterization

Proton (^1H) NMR, Carbon ($^{13}C\{^1H\}$) NMR, Tin (^{119}Sn) NMR were obtained on a JNM-ECZ 400 MHz, JNM-ECZ 500 MHz, and Bruker Avance 600 MHz nuclear magnetic resonance spectrometer. Chemicals shifts are given in parts per million (δ units) downfield from tetramethylsilane using the residual solvent signal ($CDCl_3$ 7.26, CD_5Cl 7.14, $C_2D_2Cl_4$ 5.98, or TMS 0.00 ppm) as internal standard. ^1H NMR information is given in the following format: multiplicity (s, singlet; d, doublet; t, triplet; m, multiplet), coupling constant(s) (*J*) in Hertz (Hz), number of protons. The prefix app is occasionally applied when the true signal multiplicity was unresolved and br indicates the signal in question is broadened. $^{13}C\{^1H\}$ NMR spectra are reported in ppm (δ) relative to residual $CDCl_3$ (δ 77.16 ppm). MALDI-TOF MS spectra were obtained on a Bruker Daltionics Biflex III MALDI-TOF Analyzer. ESI MS spectra were obtained on a Q Exactive Focus LCMS (Thermo Scientific Company, USA). Elemental analysis was performed on a FLASH EA-1112 elemental analyzer. Gas-chromatographic mass spectrometry was was performed on a SHIMADZU GCMS-TQ8040, using helium as carrier gas and anthracene as a standard; intial 100 °C, 1 min, then ramp 10 °C per min to 300 °C, hold 11 min. The relative molecular weights of the polymers were measured on an Angilent Technologies PL-GPC 220 high temperature chromatograph. FT-IR tests were performed on an infrared spectrometer (NICOLET iS50, Thermo Scientific Company, USA), taking 32 scans for each sample.

UV-Vis absorption spectra were measured on a Cary 60 UV-vis Spectrophotometer. The fluorescence spectra and fluorescence quantum yields (PLQY) of the polymers in diluted chloroform solution were measured with an Edinburgh FLS1000 transient fluorescence spectrometer. All film samples were spin-cast on quartz glass. Cyclic voltammetry (CV) was performed in 0.1 M of tetrabutylammonium hexafluorophosphate (*n*-Bu_4NPF_6), acetonitrile solution as the supporting electrolyte

with a scan speed at 0.05 V/s. A Pt wire, glassy carbon discs, and Ag/AgCl were purchased from Shanghai Chenhua Co., Ltd. and used as the counter electrode, working electrode, and reference electrode, respectively. A ferrocene/ferrocenium redox couple was used as an external standard. Thermogravimetric analysis (TGA) was performed on a on a Q50 instrument (Shanghai Chenhua Co., Ltd.) at a heating rate of 10 °C·min^{-1} under a N_2 atmosphere. Differential scanning calorimeter (DSC) was performed on a Q2000 at a heating rate of 10 °C·min^{-1} under N_2 atmosphere. The melting points (Mp) of new compounds are from the melting peak in DSC micrograph.

GIWAXS measurements were performed at National Center for Nanoscience and Technology (NCNST) of China. Samples were prepared on Si substrates using same solutions like those used in organic field effect transistors (OFETs) devices.

The preparation of the **P2** NPs was carried out an ultrasonic cell disruptor (SCIENTZ, JY88-IIN). Size and zeta potential of the **P2** nanoparticles (NPs) were recorded by dynamic laser light scattering (DLS, Zetasizer Nano ZS). The morphology and size of the NPs were measured by the transmission electron microscope (TEM, Tecnai G2 F20 S-TWIN (200KV)).

II. Materials and synthesis

II-1. Materials

All reactions were carried out in flame-dried glasswares under N_2 atmosphere with the exclusion of air and moisture using standard Schlenk techniques. Toluene and tetrahydrofuran were freshly distilled from sodium benzophenone ketyl immediately prior to use air- and moisture-sensitive liquids. Reactions were stirred using Teflon-coated magnetic stir bars. Room temperature (25 °C) and elevated temperatures were maintained using Thermostat-controlled silicone oil baths. Organic solutions were concentrated using a rotary evaporator with a diaphragm vacuum pump. Analytical TLC were performed on silica gel GF254 plates. The TLC plates were visualized by ultraviolet light (λ =254 nm). Purification of products was accomplished by column chromatography on silica gel. 4,8-bis((2-octyldodecyl)oxy)benzo[1,2-*b*:4,5-

b']dithiophene-2,6-diyl)bis(trimethylstannane) (**N1**), 3,3'''-bis(2-hexyldecyl)-[2,2':5',2'':5'',2'''-quaterthiophene]-5,5'''-diyl)bis(trimethylstannane) (**N4**), 3,3'''-bis(2-octyldodecyl)-[2,2':5',2'':5'',2'''-quaterthiophene]-5,5'''-diyl)bis(trimethylstannane) (**N5**), and 2,5-bis(3-(2-octyldodecyl)-5-(trimethylstannyl)thiophen-2-yl)thieno[3,2-*b*]thiophene (**N6**) were purified with recycling preparative HPLC LaboACE LC-5060, on a Japan Analytical Industry Co., Ltd. chromatograph, and the mobile phase was dichloromethane. Compounds (4,4,9,9-tetrakis(4-hexylphenyl)-4,9-dihydro-*s*-indaceno[1,2-*b*:5,6-*b*']dithiophene-2,7-diyl)bis(trimethyl-stannane) (**N2**), 2-(4,4,9,9-tetrakis(4-hexylphenyl)-4,9-dihydro-*s*-indaceno[1,2-*b*:5,6-*b*']dithiophen-2-yl)bis(trimethylstannane) (**N3**) and (E)-1,2-bis(3-tetradecyl-5-(trimethylstannyl)thiophen-2-yl)ethane (**N7**) (4,8-bis(5-(2-ethylhexyl)thiophen-2-yl)benzo[1,2-*b*:4,5-*b*']dithiophene-2,6-diyl)bis(trimethylstannane) **(N9)** and 2,7-dibromo-4,4,9,9-tetrakis(4-hexylphenyl)-4,9-dihydro-*s*-indaceno[1,2-*b*:5,6-*b*'] dithiophene (**IDT-Br**) were purchased from Derthon OPV Co., Ltd.. The reagent 2,5-bis(2-octyldodecyl)-3,6-bis(5-(trimethylstannyl)thiophen-2-yl)-2,5-dihydropyrrolo[3,4-*c*]pyrrole-1,4-dione (**N8**) was purchased from Derthon Optoelectronic Materials Science Technology Co., Ltd. Compounds 2,5-bis(methylsulfanyl)thiophene and 1,4-bis(methylsulfanyl)benzene were purchased from Chemieliva Pharmaceutical Co., Ltd.

Pluronic® F127 (PF127, M_w = 12.6 kDa) was obtained from Sigma-Aldrich LLC. Cell Counting Kit-8 (CCK-8) was obtained from the DOJINDO. Roswell Park Memorial Institute 1640 (PRMI-1640) culture medium, penicillin, streptomycin and phosphate buffer saline (PBS) were obtained from Thermo Fisher Technology (China) Co., Ltd. Fetal bovine serum (FBS) was obtained from the BkBio (Beijing) Tech. Co., Ltd.

Unless stated, all other reagents and chemicals were purchased from various commercial sources and were used as received.

II-2. Preparation and characterization

1. Synthesis of electrophiles

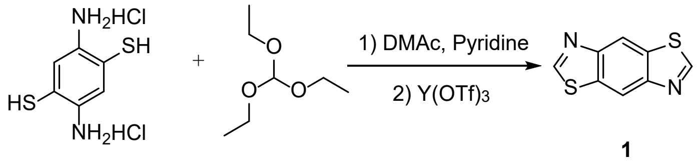

Benzo[1,2-*d*:4,5-*d'*]bis(thiazole) (1)

In a flask, 2,5-diaminobenzene-1,4-dithiol dihydrochloride (1.00 g, 4.08 mmol) and pyridine (645.5 mg, 8.16 mmol) were dissolved in DMAc (10.0 mL). The solution was added via a syringe to a mixture of triethylorthoformate (1.81 g, 12.24 mmol) and yttrium(III) trifluoromethanesulfonate (109.4 mg, 0.20 mmol) in a round-bottomed flask. The reaction mixture was stirred at 55 °C for 1 h and then cooled to room temperature. The mixture was diluted with water and the product collected by filtration. Recrystallization with dichloromethane/petroleum ether provided compound **1** as a yellow solid (330.0 mg, 42% yield). ^{1}H NMR (400 MHz, DMSO): δ 8.96 (s, 2H), 9.53 (s, 2H). The ^{1}H NMR is in agreement with the reported data.[1]

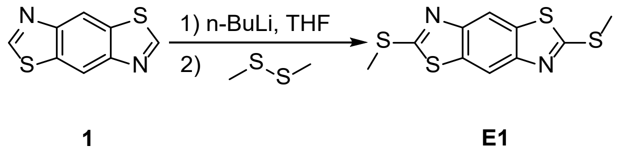

2,6-bis(methylthio)benzo[1,2-*d*:4,5-*d'*]bis(thiazole) (E1)

To a solution of compound **1** (135.0 mg, 0.70 mmol) in dry THF (30.0 mL) was added *n*-butyllithium (1.0 mL, 1.6 M in hexanes, 1.60 mmol) dropwise under N_2 atmosphere at -78 °C. After stirring at -78 °C for 1 h, 1,2-dimethyldisulfane was added (152.6 mg, 1.62 mmol) dropwise to the mixture. After the reaction solution was slowly warmed to room temperature and kept stirring overnight, the reaction was quenched with water, and extracted with dichloromethane three times. Then the organic layers were combined, dried over anhydrous $MgSO_4$, filtered, and concentrated to be purified by flash column chromatography on silica gel (dichloromethane). The compound **E1** was obtained as a golden-yellow solid (127.8 mg, 64% yield). ^{1}H NMR (400 MHz, $CDCl_3$): δ 8.20 (s,

2H), 2.82 (s, 6H). $^{13}C\{^1H\}$ NMR (100 MHz, $CDCl_3$): δ 168.80, 150.65, 134.42, 113.09, 16.09. IR (film), ν (cm^{-1}): 3077, 2926, 1469, 1420, 1393, 1302, 1202, 1202, 999, 960, 843, 883, 716, 603. HRMS (*m/z*): $[M+H]^+$ calcd. for $C_{10}H_9N_2S_4$, 284.9644; found, 284.9639.

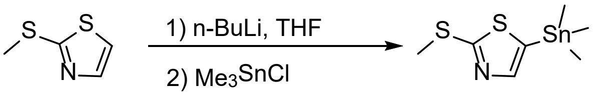

2

2-(methylthio)-5-(trimethylstannyl)thiazole (2)

To a solution of 2-(methylthio)thiazole (1.00 g, 7.62 mmol) in dry THF (20.0 mL) was added *n*-butyllithium (3.66 mL, 2.5 M in hexanes, 9.15 mmol) dropwise under N_2 atmosphere at -78 °C. After stirring at -78 °C for 2 h, trimethyltin chloride (9.91 mL, 1.0 M in hexane, 9.91 mmol) was added dropwise to the mixture. After the reaction solution was slowly warmed to room temperature and kept stirring overnight, the reaction was quenched with water, and extracted with dichloromethane three times. Then the organic layers were combined, dried over anhydrous $MgSO_4$, filtered, and concentrated to be purified by recycling preparative HPLC with dichloromethane. The compound **2** was obtained as a yellow liquid (1.34 g, 62% yield). ^1H NMR (500 MHz, $CDCl_3$): δ 7.53 (s, 1H), 2.63 (s, 3H), 0.33 (s, 9H). $^{13}C\{^1H\}$ NMR (125 MHz, $CDCl_3$): δ 170.91, 148.81, 128.53, 16.92, -8.13. ^{119}Sn NMR (224 MHz, $CDCl_3$): -32.78. IR (film), ν (cm^{-1}): 3060, 2980, 2917, 1469, 1429, 1370, 1312, 1243, 1193, 1142, 1022, 959, 851, 776, 719, 605, 537. HRMS (*m/z*): $[M+H]^+$ calcd. for $C_7H_{14}NS_2Sn$, 295.9585; found, 295.9577.

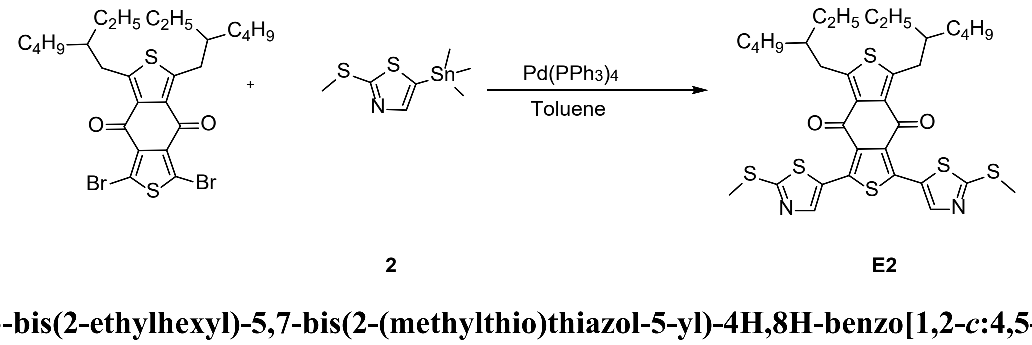

2 E2

1,3-bis(2-ethylhexyl)-5,7-bis(2-(methylthio)thiazol-5-yl)-4H,8H-benzo[1,2-*c*:4,5-*c'*]dithiophene-4,8-dione (E2)

Under N_2 atmosphere, a mixture of 1,3-dibromo-5,7-bis(2-ethylhexyl)-4H,8H-benzo[1,2-*c*:4,5-*c'*]dithiophene-4,8-dione (584.4 mg, 0.97 mmol), compound **2** (600.0 mg, 2.04 mmol) and $Pd(PPh_3)_4$ (56.0 mg, 48.5 μmol) in anhydrous toluene (20.0 mL) was stirred at 100 °C for 12 h. After cooling to room temperature, the solution was quenched with saturated KF solution and extracted with dichloromethane for three times, and then the organic layer was dried over anhydrous $MgSO_4$ and filtered. After removing the solvent from the filtrate under reduced pressure, the residue was purified by column chromatography using dichloromethane as the mobile phase to give the compound **E2** as an orange-red solid (500.0 mg, 73% yield). ^1H NMR (500 MHz, $CDCl_3$): δ 8.08 (s, 2H), 3.18 (d, J = 7.1 Hz, 4H), 2.71 (s, 6H), 1.71-1.66 (m, 2H), 1.38-1.25 (m, 16H), 0.91-0.85 (m, 12H). $^{13}C\{^1H\}$ NMR (125 MHz, $CDCl_3$): δ 177.25, 171.34, 154.18, 145.05, 138.32, 132.52, 132.13, 127.80, 41.11, 33.70, 32.85, 28.86, 26.07, 23.10, 16.34, 14.27, 10.93. IR (film), ν (cm^{-1}): 3049, 2956, 2918, 2854, 1647, 1487, 1448, 1350, 1310, 1280, 1196, 1150, 1067, 966, 867, 838, 758, 704, 608. HRMS (*m*/*z*): $[M+H]^+$ calcd. for : $C_{34}H_{42}N_2O_2S_6$, 703.1644; found, 703.1635.

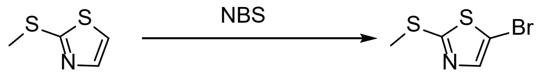

3

5-bromo-2-(methylthio)thiazole (3)

Compound 2-(methylthio)thiazole (1.00 g, 7.62 mmol) was dissolved in dichloromethane (10.0 mL) and acetic acid (10.0 mL). N-bromosuccinimide (1.63 g, 9.14 mmol) was added in batches at 0 °C under darkness. The solution was slowly restored to room temperature and stirred overnight. Saturated sodium carbonate was added to neutralize the reaction solution. And then the solution was extracted three times with dichloromethane. The organic phase was dried over anhydrous $MgSO_4$, filtered, and evaporated under reduced pressure. The crude product was purified by silica gel chromatography (petroleum ether : dichloromethane = 2:1, *v/v*)to give compound **3** as a light-yellow liquid (1.03 g, 64% yield). ^1H NMR (500 MHz, $CDCl_3$): δ 7.51 (s, 1H), 2.66 (s, 3H). The ^1H NMR spectra match the reported data.[2]

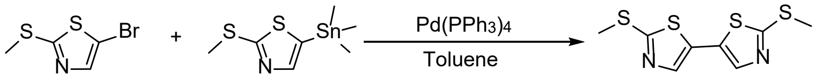

3 2 E3

2,2'-bis(methylthio)-5,5'-bithiazole (E3)

Under an N_2 atmosphere, a mixture of compound **2** (1.31 g, 4.46 mmol), compound **3** (1.03 g, 4.90 mmol) and Pd $(PPh_3)_4$ (257.7 mg, 0.22 mmol) in anhydrous toluene (20.0 mL) was stirred at 100 °C for 12 h. After cooling to room temperature, the solution was quenched with saturated KF solution and extracted with dichloromethane for three times, and then the organic layer was dried over anhydrous $MgSO_4$ and filtered. After removing the solvent under reduced pressure, the residue was purified by column chromatography using dichloromethane as mobile phase to give the compound **E3** as an orange solid (0.85 g, 73% yield). ^1H NMR (500 MHz, $CDCl_3$): δ 7.59 (s, 2H), 2.71 (s, 6H). $^{13}C\{^1H\}$ NMR (125 MHz, $CDCl_3$): δ 166.47, 139.89, 127.40, 16.72. Mp, 110 °C. IR (film), ν (cm^{-1}): 3094, 3060, 2991, 2946, 2917, 2850, 1705, 1534, 1429, 1371, 1318, 1259, 1213, 1162, 1096, 1048, 1008, 958, 924, 880, 831, 759, 717, 591. HRMS (*m*/*z*): $[M+H]^+$ calcd. for: $C_8H_8N_2S_4$, 260.9644; found, 260.9640.

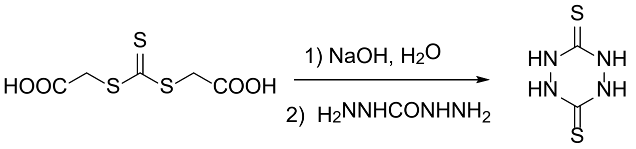

4

A suspension of bis(carboxymethyl)trithiocarbonate (6.79 g, 30.00 mmol) in H_2O (90.0 mL) was treated with NaOH (2.40 g, 60.00 mmol). Upon completion, the mixture was added to a suspension of thiocarbohydrazide (3.18 g, 30.00 mmol) in H_2O (120.0 mL) under a N_2 atmosphere by syringe and stirred at 25 °C for 3 d. The white precipitate was then collected by filtration, washed with H_2O (30 mL × 2), and vacuum dried to afford compound **4** (2.08 g, 47%) used without further purification.[3]

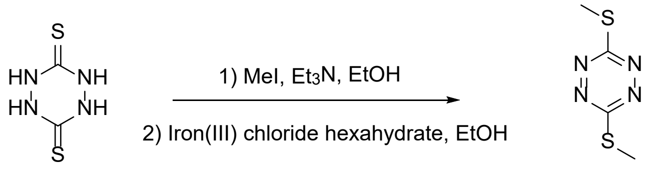

E4

A suspension of compound **4** (2.27 g, 15.30 mmol) in EtOH (30 mL) was treated with

Et_3N (3.10 g, 4.30 mL, 30.60 mmol) and MeI (4.30 g, 2.0 mL, 30.60 mmol) successively under N_2 atmosphere at 0 °C. The mixture was warmed to 25 °C and stirred for 2 h. The solvent was removed under reduced pressure. The residue was dissolved in EtOH (15.0 mL) and treated with $FeCl_3·6H_2O$ (10.30 g, 38.00 mmol). The mixture was stirred for 3 h at room temperature, which was then diluted with H_2O (60.0 mL) and extracted with EtOAc for three times. The combined organic phase was washed with saturated aqueous NaCl, dried over $MgSO_4$, and concentrated under reduced pressure. The residue was purified by column chromatography (petroleum ether : ethyl acetate = 10:1, *v*/*v*) to afford compound **E4** (1.73 g, 33% over 2 steps) as a red solid identical to material previously reported. ^{1}H NMR (500 MHz, $CDCl_3$) δ 2.71 (s, 6H). The ^{1}H NMR are matching with the reported data.[3]

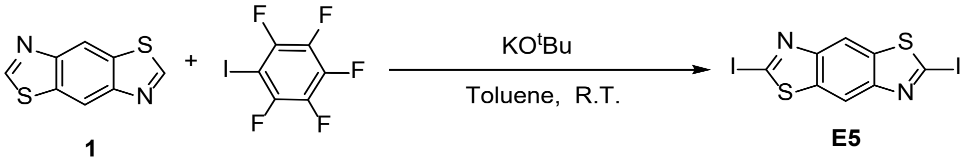

2,6-diiodobenzo[1,2-*d*:4,5-*d'*]bis(thiazole) (E5)

To a solution of compound **1** (200.0 mg, 1.04 mmol) in toluene (5.0 mL) were added pentafluoroiodobenzene (673.2 mg, 2.29 mmol) and potassium *t*-butoxide (117.0 mg, 1.04 mmol). The mixture was stirred at room temperature for 1 h. After the reaction was completed, the solution was filtered by suction filtration and the filter cake was washed several times with toluene, methanol, *n*-hexane and dichloromethane in that order. After drying several hours in vacuum oven, compound **E5** was obtained as a colorless solid (416.0 mg, 90% yield). ^{1}H NMR (400 MHz, $CDCl_3$): δ 8.49 (s, 2H). The ^{1}H NMR spectra match the reported data.[4]

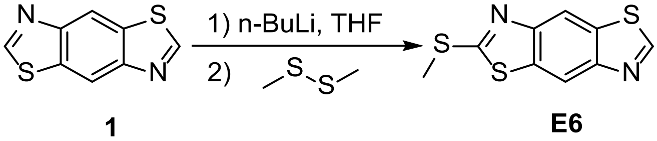

2-(methylthio)benzo[1,2-*d*:4,5-*d'*]bis(thiazole) (E6)

To a solution of compound **1** (400.0 mg, 2.08 mmol) in dry THF (100.0 mL) was added *n*-butyllithium (2.5 M, 0.83 mL in hexanes, 2.08 mmol) dropwise under N_2 atmosphere

at -78 °C. After stirring at -78 °C for 1 h, 1,2-dimethyldisulfane (200.0 mg, 2.12 mmol) was added dropwise to the mixture. After the reaction solution was slowly warmed to room temperature and kept stirring overnight, the reaction was quenched with water, and extracted with dichloromethane three times. Then the organic layers were combined, dried over anhydrous $MgSO_4$, filtered, and concentrated to be purified by flash column chromatography on silica gel (dichloromethane). The compound **E6** was obtained as a white solid (139.0 mg, 28% yield). ^{1}H NMR (400 MHz, $CDCl_3$): δ 9.00 (s, 1H), 8.46 (s, 1H), 8.37 (s, 1H), 2.81 (s, 3H). ^{13}C{^{1}H} NMR (100 MHz, $CDCl_3$): δ 169.92, 154.74, 151.83, 150.39, 134.72, 132.81, 115.43, 113.75, 16.02. Mp, 116 °C. IR (film), ν (cm^{-1}): 3070, 2930, 2850, 1480, 1400, 1260, 1100, 1020, 848, 799, 733, 704. HRMS (*m*/*z*): $[M+H]^+$ calcd. for $C_9H_7N_2S_3$, 238.9766; found, 238.9761.

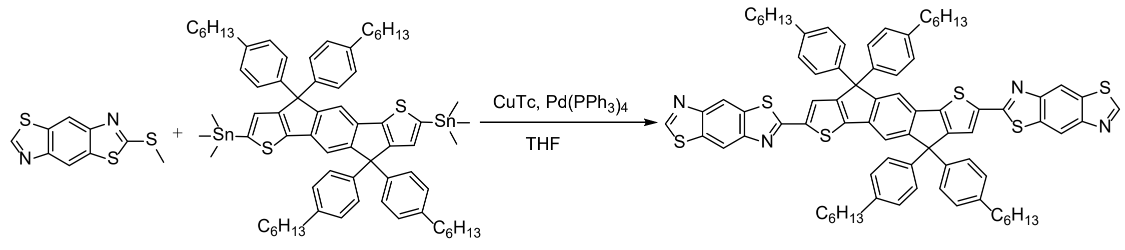

E6 **N2** **5**

2,2'-(4,4,9,9-tetrakis(4-hexylphenyl)-4,9-dihydro-*s*-indaceno[1,2-*b*:5,6-*b*']di-thiophene-2,7-diyl)bis(benzo[1,2-*d*:4,5-*d*']bis(thiazole)) (5)

Under N_2 atmosphere, a mixture of compound **E5** (139.0 mg, 0.58 mmol), **N2** (345.2 mg, 0.28 mmol), CuTc (267.0 mg, 1.40 mmol) and $Pd(PPh_3)_4$ (34.7 mg, 30.0 μmol) were dissolved in dry THF (20.0 mL). Then the mixture was stirred at 60 °C for 12 h. After quenching the reaction, the mixture was concentrated for purification by flash column chromatography on silica gel (petroleum ether : dichloromethane = 2:1, *v*/*v*) providing compound **5** as an orange-red solid (280.2 mg, 78% yield). Due to its poor solubility, the compound was not characterized by ^{13}C{^{1}H} NMR. ^{1}H NMR (400 MHz, $CDCl_3$): δ 9.02 (s, 2H), 8.54 (s, 2H), 8.52 (s, 2H), 7.61 (s, 2H), 7.54 (s, 2H), 7.22 (d, *J* = 8.4 Hz, 8H), 7.12 (d, *J* = 8.4 Hz, 8H), 2.60-2.56 (m, 8H), 1.64-1.56 (m, 8H), 1.36-1.27 (m, 24H), 0.88-0.85 (m, 12H). IR (film), ν (cm^{-1}): 2920, 2851, 1654, 1540, 1507, 1458, 1419, 1093, 876, 668. HRMS (*m*/*z*): $[M]^+$ calcd. for $C_{80}H_{78}N_4S_6$, 1286.4542;

found, 1286.4545.

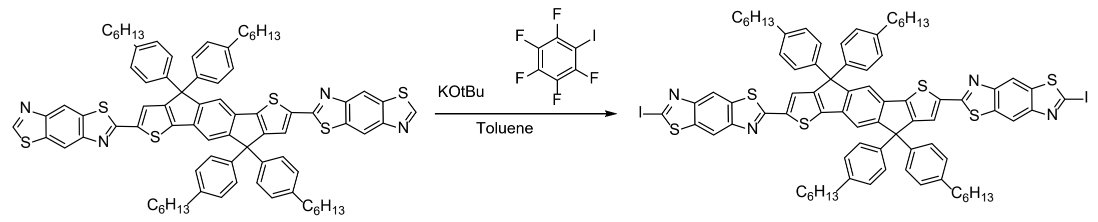

5 **E7**

6,6'-(4,4,9,9-tetrakis(4-hexylphenyl)-4,9-dihydro-*s*-indaceno[1,2-*b*:5,6-*b*']dithiophene-2,7-diyl)bis(2-iodobenzo[1,2-*d*:4,5-*d*']bis(thiazole)) (E7)

To a solution of compound **4** (280.2 mg, 0.22 mmol) in toluene (50.0 mL) were added pentafluoroiodobenzene (141.1 mg, 0.48 mmol) and potassium *t*-butoxide (24.7 mg, 0.22 mmol). The mixture was stirred at room temperature for 30 min. After the reaction was completed, the solution was filtered through Celite, concentrated, and subjected to chromatography on silica gel with petroleum ether : dichloromethane = 3:1 (*v*/*v*) as eluent. Compound **E7** was obtained as an orange-red solid (166.0 mg, 49% yield). Due to its poor solubility, the compound was not characterized by $^{13}C\{^1H\}$ NMR. ^1H NMR (400 MHz, $CDCl_3$): δ 8.42 (s, 2H), 8.38 (s, 2H), 7.60 (s, 2H), 7.53 (s, 2H), 7.21 (d, J = 7.8 Hz, 8H), 7.12 (d, J = 7.9 Hz, 8H), 2.60-2.56 (m, 8H), 1.63-1.56 (m, 8H), 1.35-1.25 (m, 24H), 0.91-0.83 (m, 12H). IR (film), ν (cm^{-1}): 2981, 2936, 1471, 1370, 1263, 1163, 1090, 982, 846, 799. HRMS (m/z): $[M]^+$ calcd. for $C_{80}H_{76}I_2N_4S_6$, 1538.2476; found, 1538.2478.

2. Synthesis of nucleophiles

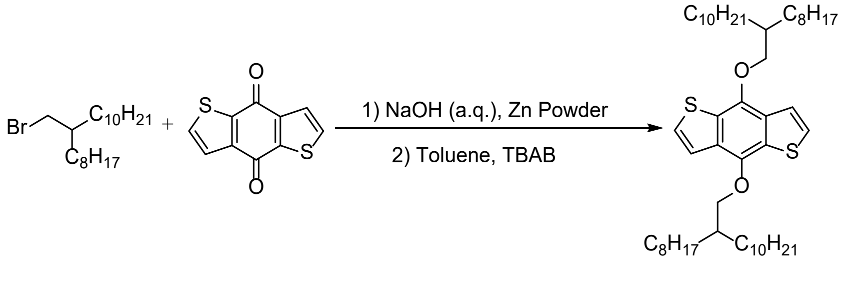

6

4,8-bis(2-octyldodecyloxy)benzo[1,2-*b*:3,4-*b*']dithiophene (6)

Benzo[1,2-*b*:4,5-*b*']dithiophene-4,8-dione (0.50 g, 2.27 mmol) was mixed with zinc powder (0.33 g, 5.05 mmol) and aqueous sodium hydroxide (NaOH) (15.0 mL, 12.0 *wt*%) solution under N_2 atmosphere. Then the resulting mixture was refluxed for 2 h. A

solution of 9-(bromomethyl)nonadecane (1.72 g, 4.76 mmol) in toluene (20.0 mL) and a catalytic amount of *n*-Bu_4NBr (173.5 mg, 0.48 mmol) were sequentially added into the mixture solution. After being refluxed for overnight, the reaction was poured into cold water and extracted with dichloromethane. The organic layer was dried over anhydrous $MgSO_4$, filtered, and the filtrate concentrated under vacuum. Then the crude product was purified by silica gel chromatography eluting with petroleum ether to give compound **6** as a colorless liquid (1.62 g, 91% yield). ^1H NMR (400 MHz, $CDCl_3$): δ 7.47 (d, J = 5.6 Hz, 2H), 7.36 (d, J = 5.6 Hz, 2H), 4.17 (d, J = 4.8 Hz, 4H), 1.88-1.83 (m, 2H), 1.67-1.59 (m, 4H), 1.52-1.45 (m, 4H), 1.44-1.37 (m, 8H), 1.37-1.24 (m, 48H), 0.90-0.86 (m, 12H). The ^1H NMR spectra match the reported data.[5]

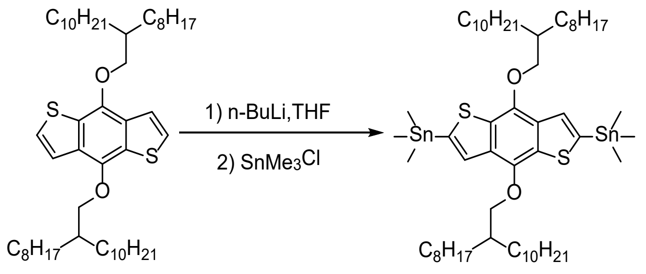

6 **N1**

2,6-bis(trimethyltin)-4,8-bis(2-octyldodecyloxy)benzo[1,2-*b*:3,4-*b*']dithiophene (N1)

A solution of *n*-butyllithium (2.98 mL, 1.6 M in hexanes, 4.78 mmol) was added dropwise to the solution of compound **6** (1.50 g, 1.91 mmol) in THF (20.0 mL) at 0 °C under N_2 atmosphere. After the mixture was stirred for 1 h, chlorotrimethylstannane (4.8 mL, 1.0 M in hexane, 4.80 mmol) was added into the mixture. The mixture was then allowed to warm up to room temperature, and stirred for 12 h. After diluting with dichloromethane and washing with water, the organic phase was dried over anhydrous $MgSO_4$, filtered, and the filtrate evaporated under reduced pressure. The crude was purified by recycling preparative HPLC with dichloromethane and recrystallized from methanol at 0 °C to afford **N1** as a white solid (1.25 g, 59% yield). ^1H NMR (400 MHz, $CDCl_3$): δ 7.51 (s, 2H), 4.18 (d, J = 8.0 Hz, 4H), 1.88-1.83 (m, 2H), 1.67-1.63 (m, 4H), 1.51-1.48 (m, 4H), 1.44-1.40 (m, 8H), 1.32-1.24 (m, 48H), 0.90-0.86 (m, 12H), 0.44 (s, 18H). The ^1H NMR spectra match the reported data.[5]

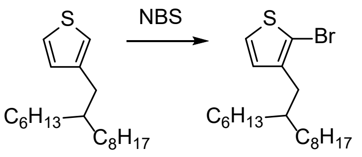

7

2-bromo-3-(2-hexyldecyl)thiophene (7)

Compound 3-(2-hexyldecyl)thiophene (600.0 mg, 1.94 mmol) was dissolved in dichloromethane (10.0 mL) and acetic acid (10.0 mL). N-bromosuccinimide (345.3 mg, 1.94 mmol) was added in batches at 0 °C under darkness. The solution was allowed to slowly warm to room temperature and stirred overnight. Saturated sodium carbonate was added to neutralize the reaction solution. And then the solution was extracted three times with dichloromethane. The organic phase was dried over anhydrous $MgSO_4$, filtered, and the filtrate was evaporated under reduced pressure. The crude product was purified by silica gel chromatography eluting with petroleum ether to give compound **7** as a colorless liquid (754.1 mg, 95% yield). ^{1}H NMR (400 MHz, $CDCl_3$): δ 7.17 (d, J = 5.6 Hz, 1H), 6.76 (d, J = 5.6 Hz, 1H), 2.49 (d, J = 7.2 Hz, 2H), 1.69-1.59 (m, 1H), 1.30-1.22 (m, 24H), 0.90-0.86 (m, 6H). The ^{1}H NMR spectra match the reported data.[7]

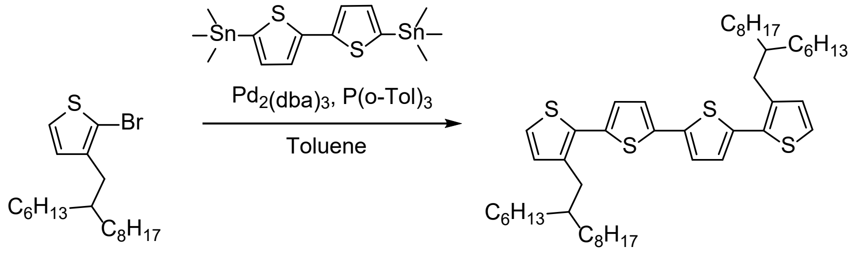

7 **8**

3,3'''-bis(2-hexyldecyl)-2,2':5',2'':5'',2'''-quaterthiophene (8)

Under N_2 atmosphere, a mixture of compound **7** (740.0 mg, 1.91 mmol), 5,5'-bis(trimethylstannyl)-2,2'-bithiophene (467.3 mg, 0.95 mmol), tris(2-methylphenyl)phosphine (58.1 mg, 0.19 mmol) and $Pd_2(dba)_3$ (43.7 mg, 47.7 μmol) in anhydrous toluene (15.0 mL) was stirred at 100 °C for 12 h. After cooling to room temperature, the solution was quenched with saturated KF solution and extracted with dichloromethane for three times, and then the organic layer was dried over anhydrous

$MgSO_4$ and filtered. After removing the solvent from the filtrate under reduced pressure, the residue was purified by column chromatography using petroleum ether as mobile phase to give the compound **8** as a yellow oil (610.0 mg, 82% yield). ^{1}H NMR (400 MHz, $CDCl_3$): δ 7.19 (d, J = 5.2 Hz, 2H), 7.12 (d, J = 3.7 Hz, 2H), 7.02 (d, J = 3.7 Hz, 2H), 6.91 (d, J = 5.2 Hz, 2H), 2.72 (d, J = 7.2 Hz, 4H), 1.74-1.65 (m, 2H), 1.27-1.21 (m, 48H), 0.90-0.85 (m, 12H). $^{13}C\{^1H\}$ NMR (100 MHz, $CDCl_3$): δ 139.27, 137.09, 135.38, 130.91, 130.71, 126.96, 123.86, 123.76, 39.09, 33.89, 33.60, 32.10, 30.22, 29.90, 29.82, 29.53, 26.65, 22.87, 14.31. IR (film), ν (cm^{-1}): 2921, 2851, 1456, 1376, 1236, 1042, 833, 792, 723, 694, 653. HRMS (*m/z*): $[M]^+$ calcd. for: $C_{48}H_{74}S_4$, 778.4673; found, 778.4663.

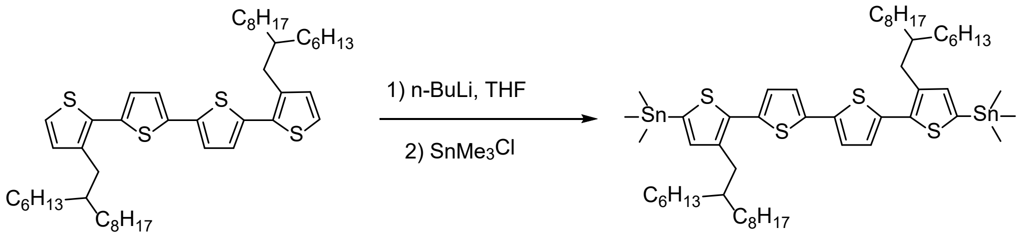

8 **N4**

(3,3'''-bis(2-hexyldecyl)-[2,2':5',2'':5'',2'''-quaterthiophene]-5,5'''-diyl)bis(tri-methylstannane) (N4)

To a solution of compound **8** (0.26 g, 0.33 mmol) in anhydrous THF (10.0 mL), was added *n*-butyllithium (0.46 mL, 1.6 M in hexanes, 0.73 mmol) dropwise via syringe at 0 °C under N_2 atmosphere. The mixture was stirred at 0 °C for 1 h. Afterwards, chlorotrimethylstannane (0.83 mL, 1 M in hexane, 0.83 mmol) was added to the solution. The mixture was warmed to room temperature and stirred for 12 h. After quenching the reaction with water, the solution was extracted with dichloromethane, and the organic layer was dried over anhydrous $MgSO_4$ and filtered. After removing the solvent from the filtrate under reduced pressure, the residue was purified by recycling preparative HPLC to give compound **N4** as a yellow oil (150.5 mg, 41% yield). ^{1}H NMR (400 MHz, $CDCl_3$): δ 7.13 (d, J = 4.2 Hz, 2H), 7.03 (d, J = 3.7 Hz, 2H), 7.02 (s, 2H), 2.77 (d, J = 7.2 Hz, 4H), 1.78-1.71 (m, 2H), 1.33-1.24 (m, 48H), 0.92-0.88 (m,

12H), 0.42 (s, 18H). $^{13}C\{^1H\}$ NMR (100 MHz, $CDCl_3$): δ 140.29, 139.10, 136.89, 136.59, 136.43, 135.63, 126.40, 123.68, 38.99, 33.71, 33.59, 32.08, 30.20, 29.87, 29.80, 29.52, 22.84, 14.29, -8.14. ^{119}Sn NMR (224 MHz, $CDCl_3$): -26.92. IR (film), ν (cm^{-1}): 2921, 2853, 1456, 1376, 1191, 1061, 976, 909, 850, 791, 773, 735. HRMS (*m/z*): $[M]^+$ calcd. for: $C_{54}H_{90}S_4Sn_2$, 1104.3963; found, 1104.3973.

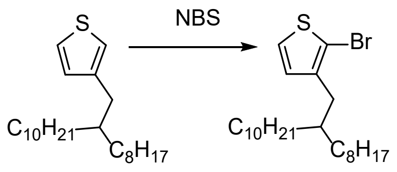

9

2-bromo-3-(2-octyldodecyl)thiophene (9)

3-(2-octyldodecyl)thiophene (700.0 mg, 1.92 mmol) was dissolved in dichloromethane (10.0 mL) and acetic acid (10.0 mL). N-bromosuccinimide (342.4 mg, 1.92 mmol) was added in batches at 0 °C in dark conditions. The solution was slowly cooled to room temperature and was stirred overnight. Saturated sodium carbonate was added to neutralize the reaction solution. And then the solution was extracted three times with dichloromethane. The organic phase was then dried over anhydrous $MgSO_4$, filtered, and the filtrate evaporated under reduced pressure. The crude product was purified by silica gel chromatography eluting with petroleum ether to give compound **9** as a colorless liquid (809.3 mg, 95% yield). ^1H NMR (400 MHz, $CDCl_3$): δ 7.18 (d, J = 5.6 Hz, 1H), 6.76 (d, J = 5.6 Hz, 1H), 2.49 (d, J = 7.2 Hz, 2H), 1.68-1.59 (m, 1H), 1.28-1.21 (m, 32H), 0.90-0.86 (m, 6H). The ^1H NMR spectra match the reported data.[7]

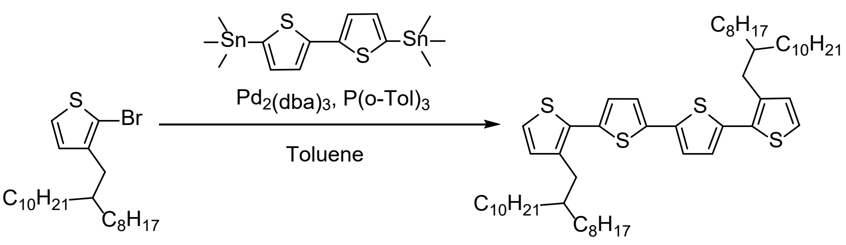

9 **10**

3,3'''-bis(2-octyldodecyl)-2,2':5',2'':5'',2'''-quaterthiophene (10)

A reaction flask was added compound **9** (840.0 mg, 1.89 mmol), 5,5'-

bis(trimethylstannyl)-2,2'-bithiophene (467.3 mg, 0.95 mmol), tris(2-methylphenyl)phosphine (57.8 mg, 0.19 mmol) and $Pd_2(dba)_3$ (43.5 mg, 47.5 μmol). Dry toluene (20.0 mL) was added to the mixture with a syringe under N_2 protection. The mixture was stirred at 100 °C for 12 h. After cooling to room temperature, the reaction system was quenched with saturated KF solution. After the solution was stratified, the liquid was separated and the aqueous phase was extracted three times by dichloromethane. The organic phase was dried over anhydrous $MgSO_4$, filtered, and evaporated under reduced pressure. The crude product was purified by silica gel chromatography eluting with petroleum ether to give compound **10** as a yellow oil (804.5 mg, 95% yield). ^{1}H NMR (400 MHz, $CDCl_3$): δ 7.19 (d, J = 5.2 Hz, 2H), 7.12 (d, J = 3.7 Hz, 2H), 7.02 (d, J = 3.8 Hz, 2H), 6.91 (d, J = 5.2 Hz, 2H), 2.72 (d, J = 7.2 Hz, 4H), 1.72-1.68 (m, 2H), 1.31-1.18 (m, 64H), 0.89-0.85 (m, 12H). The ^{1}H NMR spectra match the reported data.[8]

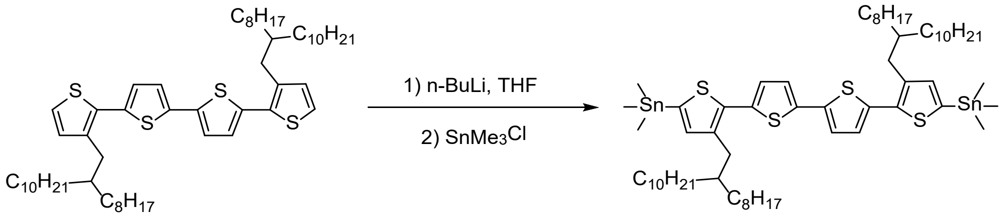

10 **N5**

(3,3'''-bis(2-octyldodecyl)-[2,2':5',2'':5'',2'''-quaterthiophene]-5,5'''-diyl)bis(trimethylstannane) (N5)

A solution of n-butyllithium (1.42 mL, 1.6 M in hexanes, 2.27 mmol) was added to the solution of compound **10** (810.0 mg, 0.91 mmol) in THF (20.0 mL) dropwise via vigorous stirring at 0 °C under N_2 atmosphere. After the mixture was stirred for 1 h, chlorotrimethylstannane (2.30 mL, 1.0 M in hexanes, 2.30 mmol) was added to the mixture. The mixture was allowed to warm up to room temperature and stirred for 12 h. After diluting with dichloromethane and washing with water, the organic phase was dried over anhydrous $MgSO_4$ and filtered. After removing the solvent under reduced pressure, the residue was purified by recycling preparative HPLC to give compound **N5** as a yellow oil (366.6 mg, 33% yield). ^{1}H NMR (400 MHz, $CDCl_3$): δ 7.11 (d, J = 3.7 Hz, 2H), 7.00 (d, J = 3.8 Hz, 2H), 6.96 (s, 2H), 2.73 (d, J = 7.1 Hz, 4H), 1.74-1.68

(m, 2H), 1.26-1.21 (m, 64H), 0.89-0.86 (m, 12H), 0.39 (s, 18H). The ^{1}H NMR spectra match the reported data.[8]

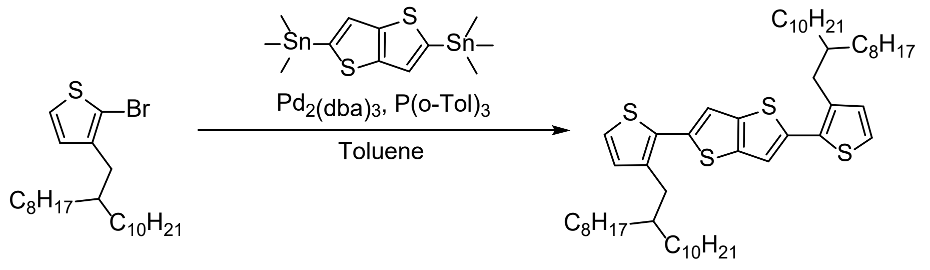

9 **11**

2,5-bis(3-(2-octyldodecyl)thiophen-2-yl)thieno[3,2-*b*]thiophene (11)

A flask was added compound **9** (570.0 mg, 1.29 mmol), 2,5-bis(trimethylstannyl)thieno[3,2-*b*]thiophene (314.7 mg, 0.64 mmol), tris(2-methylphenyl)phosphine (41.2 mg, 0.14 mmol) and $Pd_2(dba)_3$ (29.0 mg, 31.7 μmol). Dry toluene (20.0 mL) was added to the mixture under N_2 atmosphere. The mixture was stirred at 100 °C for 12 h. After cooling to room temperature, the reaction system was quenched with saturated KF solution. After the solution was stratified, the liquid was separated and the aqueous phase was extracted with dichloromethane. The organic phase was dried over anhydrous $MgSO_4$, filtered, and evaporated under reduced pressure. The crude product was purified by silica gel chromatography eluting with petroleum ether to give compound **11** as a yellow oil (526.2 mg, 95% yield). ^{1}H NMR (400 MHz, $CDCl_3$): δ 7.23 (s, 2H), 7.22 (d, J = 5.2 Hz, 2H), 6.93 (d, J = 5.2 Hz, 2H), 2.74 (d, J = 7.2 Hz, 4H), 1.76-1.64 (m, 2H), 1.29-1.19 (m, 64H), 0.90-0.86 (m, 12H). The ^{1}H NMR spectra match the reported data.[8]

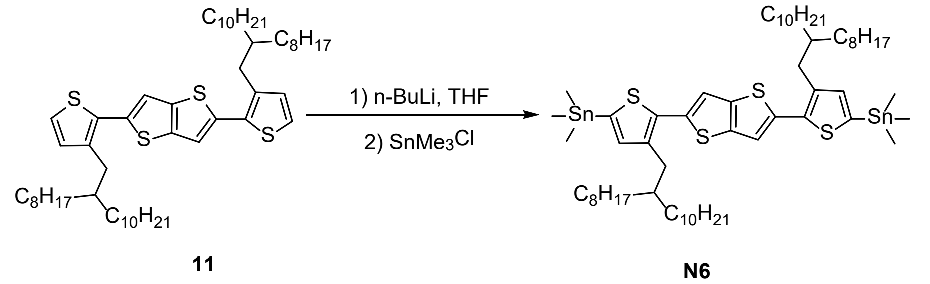

11 **N6**

2,5-bis(3-(2-octyldodecyl)-5-(trimethylstannyl)thiophen-2-yl)thieno[3,2-*b*]thiophene (N6)

A solution of *n*-butyllithium (0.88 mL, 1.6 M in hexane, 1.41 mmol) was added to the solution of compound **11** (550.0 mg, 0.64 mmol) in THF (20.0 mL) dropwise via vigorous stirring at 0 °C under N_2 atmosphere. After the mixture was stirred for 1 h, the solution of chlorotrimethylstannane (1.50 mL, 1.0 M in hexane, 1.50 mmol) was added. The mixture was then allowed to warm up to room temperature and stirred for 12 h. After diluting with dichloromethane and washing with water, the organic phase was dried over anhydrous $MgSO_4$ and filtered. After removing the solvent under reduced pressure, the residue was purified by recycling preparative HPLC to give compound **N6** as a yellow oil (468.0 mg, 61% yield). ^{1}H NMR (400 MHz, $CDCl_3$): δ 7.21 (s, 2H), 6.98 (s, 2H), 2.75 (d, J = 7.2 Hz, 4H), 1.75-1.69 (m, 2H), 1.31-1.17 (m, 64H), 0.89-0.86 (m, 12H), 0.39 (s, 18H). The ^{1}H NMR spectra match the reported data.[8]

II-3. Model reactions.

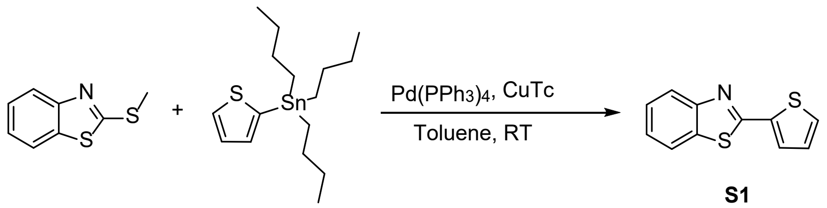

2-(thiophen-2-yl)benzo[*d*]thiazole (S1)

Under N_2 atmosphere, a mixture of compound 2-(methylthio)benzo[*d*]thiazole (MTBT) (0.50 g, 2.76 mmol), tributyl(thiophen-2-yl)stannane (1.24 g, 3.31 mmol), CuTc (2.63 g, 13.78 mmol) and $Pd(PPh_3)_4$ (324.0 mg, 0.28 mmol) were dissolved in dry toluene (15.0 mL). After 5 minutes, the solution turned dark, and then the mixture was stirred at room temperature for 12 h. After diluting with dichloromethane and washing with $NH_3{\cdot}H_2O$ (aq. 25–28%, *ω*/*ω*) for three times, the mixture was dried over anhydrous $MgSO_4$ and filtered. Then the combined organic phase was purified by flash column chromatography (silica gel, petroleum ether : dichloromethane = 3:1, *v*/*v*) to afford compound **S1** as a white solid (682.0 mg, 74% yield). ^{1}H NMR (400 MHz, $CDCl_3$): δ 8.03 (d, J = 8.0 Hz, 1H), 7.86 (d, J = 7.9 Hz, 1H), 7.67 (d, J = 4.0 Hz, 1H), 7.52 (d, J = 4.0 Hz, 1H), 7.48 (t, J = 8.2 Hz, 1H), 7.37 (t, J = 8.2 Hz, 1H), 7.15 (dd, J = 4.0 Hz, J =

4.0 Hz, 1H). The ^{1}H NMR spectr match the reported data.[9]

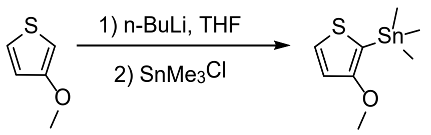

12

(3-methoxythiophen-2-yl)trimethylstannane (12)

To a solution of 3-methoxythiophene (1.00 g, 8.76 mmol) in dry THF (30.0 mL) was added *n*-butyllithium (3.86 mL, 2.5 M in hexane, 9.65 mmol) dropwise at 0 °C under N_2 atmosphere. After stirring at 0 °C for 1 h, the solution was added by trimethyltin chloride (10.00 mL, 1.0 M in hexane, 10.00 mmol) dropwise at 0 °C. Afterwards, the solution was warmed to room temperature slowly and stirred overnight. After the reaction was quenched with water, and extracted with dichloromethane for three times, the organic layers were combined and dried over anhydrous $MgSO_4$. After filtration, the solvent was removed by rotavap to afford the compound **12** as a rufous oil (2.30 g, 95% yield), and used in the next step without further purification. ^{1}H NMR (400 MHz, $CDCl_3$): δ 7.49 (d, *J* = 4.9 Hz, 1H), 6.98 (d, *J* = 4.9 Hz, 1H), 3.81 (s, 3H), 0.36 (s, 9H). $^{13}C\{^1H\}$ NMR (100 MHz, $CDCl_3$): δ 165.02, 130.80, 116.53, 59.04, -8.32. ^{119}Sn NMR (224 MHz, $CDCl_3$): -23.84. IR (film), ν (cm^{-1}): 2980, 2911, 2841, 1518, 1461, 1395, 1361, 1237, 1149, 1067, 971, 827, 776, 712, 530. HRMS (*m*/*z*): $[M+H]^+$ calcd. for: $C_8H_{14}OSSn$, 278.9861; found, 278.9857.

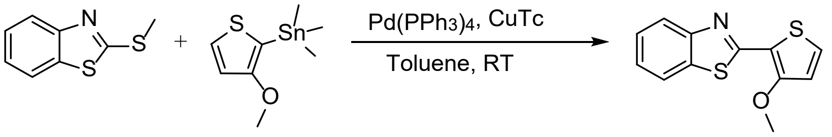

12 S2

2-(3-methoxythiophen-2-yl)benzo[*d*]thiazole (S2)

Under N_2 atmosphere, a mixture of compound MTBT (500.0 mg, 2.76 mmol), compound **11** (842.0 mg, 3.040 mmol), CuTc (2.63 g, 13.79 mmol) and $Pd(PPh_3)_4$ (323.6 mg, 0.280 mmol) were dissolved in dry toluene (15.0 mL). After 5 minutes, the solution turned dark. And then the mixture was stirred at room temperature for 12 h.

After the mixture was concentrated under vacuum, diluted with dichloromethane and washed with $NH_3{\cdot}H_2O$ (aq. 25–28%, *ω*/*ω*) for three times, the combined organic phase was dried over anhydrous $MgSO_4$. The compound **S2** was purified by flash column chromatography (silica gel, petroleum ether : dichloromethane = 5:1, *v*/*v*) as a colorless solid (580.2 mg, 85% yield). ^{1}H NMR (400 MHz, $CDCl_3$): δ 8.02 (d, *J* = 8.2 Hz, 1H), 7.85 (d, *J* = 7.9 Hz, 1H), 7.47 (t, *J* = 7.6 Hz, 1H), 7.39-7.35 (m, 1H), 7.31 (d, *J* = 4.0 Hz, 1H), 6.46 (d, *J* = 4.0 Hz, 1H), 3.86 (s, 3H). The ^{1}H NMR spectra match the reported data.[10]

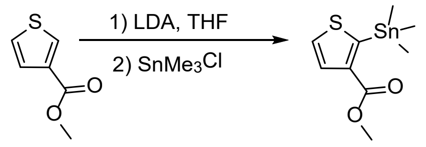

13

Methyl 2-(trimethylstannyl)thiophene-3-carboxylate (13)

To a solution of methyl thiophene-3-carboxylate (500.0 mg, 3.52 mmol) in dry THF (10.0 mL) was added lithium diisopropylamide (2.11 mL, 2.0 M in THF/*n*-heptane, 4.22 mmol) dropwise under N_2 atmosphere at -78 °C. After stirring at -78 °C for 1 h, chlorotrimethylstannane (4.30 mL, 1.0 M in hexane, 4.30 mmol) was added. The mixture was then allowed to warm up to room temperature and stirred for 12 h. After quenching the reaction, the solution was diluted with dichloromethane, and washed with water. Then the organic phase was then dried over anhydrous $MgSO_4$ and filtered. After removing the solvent under reduced pressure, the residue was purified by silica gel chromatography eluting with petroleum ether : dichloromethane = 3:1 (*v*/*v*) to give compound **13** as a light-yellow solid (700.0 mg, 65% yield). ^{1}H NMR (400 MHz, $CDCl_3$): δ 7.68 (d, *J* = 4.8 Hz, 1H), 7.56 (d, *J* = 4.8 Hz, 1H), 3.86 (s, 3H), 0.39 (s, 9H). The ^{1}H NMR spectra match the reported data.[11]

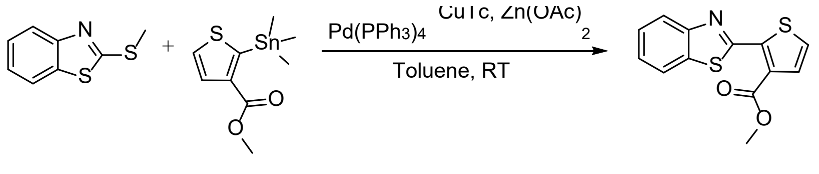

13 **S3**

Methyl 2-(benzo[*d*]thiazol-2-yl)thiophene-3-carboxylate (S3)

Under N_2 atmosphere, a mixture of compound MTBT (500.0 mg, 2.76 mmol), compound **13** (927.0 mg, 3.04 mmol), CuTc (2.63 g, 13.79 mmol), $Zn(OAc)_2$ (50.6 mg, 0.28 mmol) and $Pd(PPh_3)_4$ (323.6 mg, 0.28 mmol) were dissolved in dry toluene (15.0 mL). After 5 minutes, the solution was turned to darkness, and then the mixture was stirred at room temperature for 12 h. After the mixture was diluted with dichloromethane and washed with $NH_3{\cdot}H_2O$ (aq. 25–28%, *ω*/*ω*) three times, the combined organic phase was dried over $MgSO_4$. After filtration, the solvent was removed by the rotavap. The compound **S3** was purified by flash column chromatography as a white solid (380.0 mg, 50% yield). ^1H NMR (400 MHz, $CDCl_3$): δ 8.08 (d, J = 7.1 Hz, 1H), 7.92 (d, J = 8.6 Hz, 1H), 7.56 (d, J = 5.4 Hz, 1H), 7.52-7.49 (m, 1H), 7.44-7.40 (m, 2H), 3.96 (s, 3H). $^{13}C\{^1H\}$ NMR (100 MHz, $CDCl_3$): δ 163.64, 159.04, 152.19, 143.87, 136.91, 130.73, 129.70, 128.04, 126.53, 125.74, 123.28, 121.51, 52.33. Mp, 82 °C. IR (film), ν (cm^{-1}): 3094, 2993, 2946, 2919, 2849, 1705, 1532, 1430, 1409, 1370, 1317, 1259, 1213, 1160, 1095, 1008, 923, 880, 831, 758, 717, 592. HRMS (*m*/*z*): $[M+H]^+$ calcd. for: $C_{13}H_9NO_2S_2$, 276.0148; found, 276.0143.

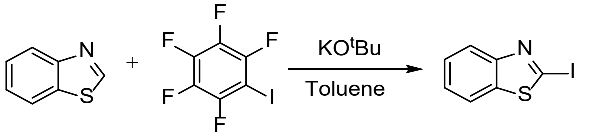

2-iodobenzo[*d*]thiazole (14)

To benzothiazole (1.24 g, 9.17 mmol) solution in toluene (23.0 mL) was added pentafluoroiodobenzene (2.70 g, 9.17 mmol) and potassium *t*-butoxide (206.0 mg, 1.84 mmol). The mixture was stirred at room temperature for 30 min. After the reaction was completed, the solution was filtered through Celite, concentrated, and subjected to chromatography on silica gel with dichloromethane as an eluent. Compound **14** was obtained as a crystalline white solid (2.04 g , 85% yield). ^1H NMR (400 MHz, $CDCl_3$): δ 8.04 (m, 1H), 7.85 (m, 1H), 7.46-7.37 (m, 2H). The ^1H NMR spectra match the previous reference.[4]

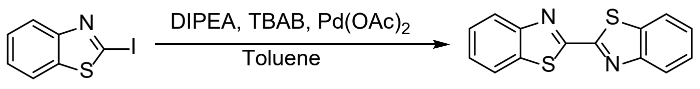

14 **S4**

2,2'-bibenzo[*d*]thiazole (S4)

Compound **14** (1.00 g, 3.83 mmol), N,N'-diisopropylethylamine (0.50 g, 3.83 mmol), *n*-Bu_4NBr (0.62 g, 1.92 mmol), and $Pd(OAc)_2$ (86.0 mg, 0.38 mmol) were dissolved in dry toluene (14.0 mL). The mixture was heated at 110 °C for 20 h. Then distilled water (50.0 mL) was added to the dark-brown solution after cooling the solution to room temperature. Next the solution was extracted with dichloromethane, the organic layers were combined and evaporated to dryness to give a dark-brown oil, diethyl ether (200.0 mL) was added to this oil and the mixture was sonicated for 30 min. The ether solution was then carefully decanted and evaporated to dryness to give compound **S4** as a dark-orange microcrystalline solid (300 mg, 59% yield). ^1H NMR (400 MHz, $CDCl_3$): δ 8.17 (d, J = 8.4 Hz, 2H), 7.99 (d, J = 8.0 Hz, 2H), 7.57 (m, 2H), 7.49 (m, 2H). The ^1H NMR spectra are matching with the previous reference.[12]

II-4. Optimization on the polymerization of P1 (P1-CS)

General synthetic procedure**:** In a Schlenk flask under an N_2 atmosphere, monomer **E1** (14.2 mg, 0.05 mmol) and monomer **N1** (55.4 mg, 0.05 mmol) were dissolved in dry toluene (2.0 mL). CuTc (47.7 mg, 0.25 mmol), and $Pd(PPh_3)_4$ (5.8 mg, 5.0 μmol) were added to the mixture, which was then stirred under N_2 atmosphere. The polymerization solution was washed with ammonium hydroxide. After quenching the reaction with methanol, the reaction mixture was poured into methanol. The crude polymer was collected by filtration and purified by successive soxhlet extraction with acetone, hexane and chloroform. The chloroform fraction was concentrated and precipitated in methanol. A dark-red solid was collected filtration.

Supplementary Table 1. Optimization of C-S cleavage polycondensation conditions for the synthesis of semiconducting polymer **P1** (**P1-CS**) from **E1** + **N1**.[a]

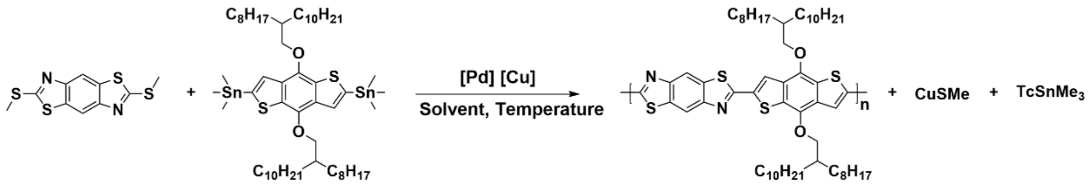

E1 **N1** **P1**

Entry	[Pd] Catalyst	[Cu] Cocatalyst	Solvent	T (°C)	Time (h)	M_n/M_w (kDa)	*Đ*	Yield[b] (%)
1	Pd(^{t}Bu$_3$P)$_2$	CuTc	Toluene	RT	24	-	-	0
2	Pd(PPh$_3$)$_2$Cl$_2$	CuTc	Toluene	RT	24	12.9/47.4	3.67	85
3	Pd(PPh$_3$)$_4$	CuTc	CB	RT	24	17.3/79.5	4.6	81
4	Pd(PPh$_3$)$_4$	CuTc	THF	RT	24	7.0/21.0	2.99	73
5	Pd(PPh$_3$)$_4$	CuTc	dioxane	RT	24	6.3/17.6	2.79	50
6	Pd(PPh$_3$)$_4$	CuTc	DMF	RT	24	-	-	0
7[c]	Pd(PPh$_3$)$_4$	CuTc	Toluene	RT	24	9.5/38.3	4.01	86
8[d]	Pd(PPh$_3$)$_4$	CuTc	Toluene	RT	24	9.9/42.1	4.27	73
9[e]	Pd(PPh$_3$)$_4$	CuTc	Toluene	RT	24	9.2/29.4	3.75	81
10[f]	Pd(PPh$_3$)$_4$	CuTc	Toluene	RT	72	-	-	0

Reaction conditions: [a] Pd catalyst (10 mol%), Cu cocatalyst (5 equiv.), **E1** (1 equiv, 0.025 M) and **N1** (1 equiv, 0.025 M) in solvent under N_2. [b] Yield collected from chloroform fraction. [c] Monomers =0.1 M each. [d] Monomers =0.001 M each. [e] Zn(OAc)$_2$ (10 mol%). [f] CuTc (10 mol%).

Supplementary Table 2. Reproducibility of the polymerization to produce **P1**.[a]

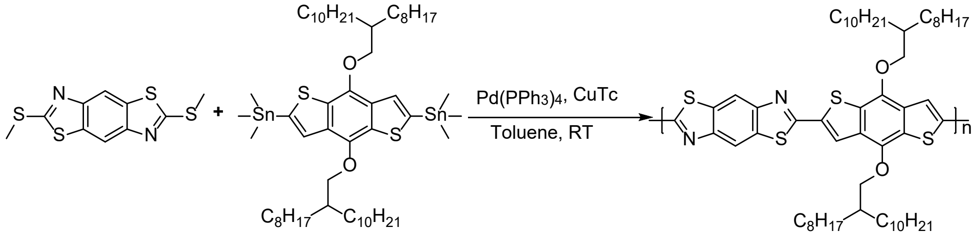

E1 **N1** **P1**

Batch	Yield (%)[b]	M_n (kDa)[c]	M_w (kDa)[c]	*Đ*[c]
1	82	15.9	65.4	4.10
2	80	15.7	67.3	4.30
3	81	16.8	76.5	4.54
4	84	16.0	75.6	4.71
5[d]	85	15.2	58.6	3.85

Reaction conditions: [a] Compound **E1** (14.2 mg, 0.05 mmol), compound **N1** (55.4 mg, 0.05 mmol), CuTc (47.7 mg, 0.25 mmol), Pd(PPh$_3$)$_4$ (5.8 mg, 10 mol%, 5.0 μmol), toluene (2.0 mL), under N_2 atmosphere, room temperature, 24 h. [b] Isolated yield of chloroform fraction. [c] Determined by GPC at 150 °C in 1,2,4-trichlorobenzene against a polystyrene standard. [d] Gram scale synthesis: compound **E1** (284.43 mg, 1.00 mmol),

compound **N1** (1109.0 mg, 1.00 mmol), CuTc (953.5 mg, 5.00 mmol), $Pd(PPh_3)_4$ (115.6 mg, 0.10 mmol), toluene (40.0 mL), at room temperature, under N_2 atmosphere, 24 h.

II-5. Classic Stille coupling

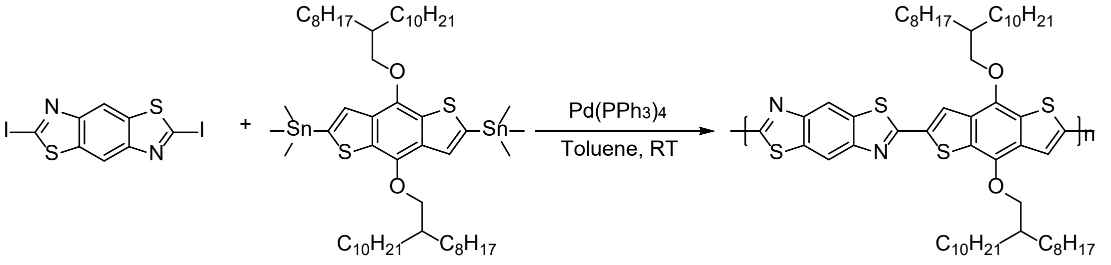

E5 **N1** **P1-Cl**

To a Schlenk flask, **E5** (22.2 mg, 0.05 mmol), **N1** (55.4 mg, 0.05 mmol) $Pd(PPh_3)_4$ (5.8 mg, 10 mol%, 5.0 μmol) and dry toluene were added under N_2 atmosphere. Then the mixture solution was stirred at room temperature for 72 h. The mixture was then poured into methanol and no precipate was collected. Thus, **P1** was not obtained under this condition.

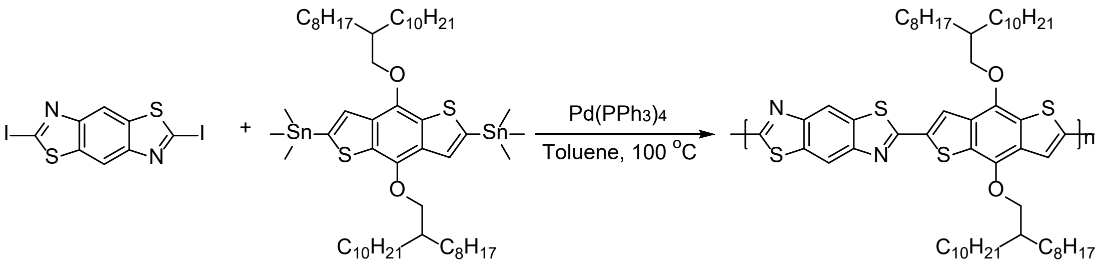

E5 **N1** **P1-Cl**

In a Schlenk flask, **E5** (22.2 mg, 0.05 mmol), **N1** (55.4 mg, 0.05 mmol) and dry toluene were added under N_2 atmosphere. $Pd(PPh_3)_4$ (5.8 mg, 10 mol%, 5.0 μmol) was added to the mixture under N_2. Then the mixture solution was stirred at 100 °C for 72 h. Afterwards, the reaction mixture was poured into methanol. The crude polymer was collected by filtration and purified by soxhlet extraction successively with acetone, hexane, and chloroform. The chloroform solution was concentrated, poured into methanol, and then collected by filtration to afford the copolymers **P1** (39.8 mg, 82% yield) as a red powder. GPC: M_n 11.1 kDa, M_w 46.1 kDa, $Đ$ 4.17. ^{1}H NMR (400 MHz, $CDCl_3$): δ 7.71, 7.49, 4.08, 1.77, 1.63, 1.50, 1.29, 0.88. IR (film), ν (cm^{-1}): 2916, 2850,

1554, 1466, 1438, 1398, 1358, 1311, 1247, 1163, 1037, 881, 854, 828, 720, 684, 607. Analysis (calcd., found for $(C_{58}H_{86}N_2O_2S_4)_n$): C (71.70, 72.45), H (8.92, 9.23), N (2.88, 2.62), S (13.20, 12.28). PLQY, 4%.

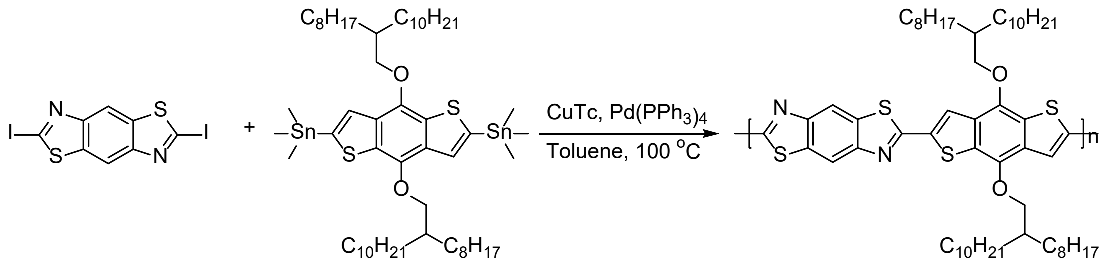

E5 N1 P1-Cl

In a Schlenk flask, **E5** (22.2 mg, 0.05 mmol), **N1** (55.4 mg, 0.05 mmol) and dry toluene were added under N_2 atmosphere. $Pd(PPh_3)_4$ (5.8 mg, 10 mol%, 5.0 μmol) and CuTc (47.7 mg, 0.25 mmol) were added to the mixture. Then the mixture solution was stirred at 100 °C for 72 h. Afterwards, the reaction mixture was poured into methanol. The crude polymer was collected by filtration and purified by soxhlet extraction with acetone, hexane and chloroform. The chloroform solution was concentrated, poured into methanol, and then collected by filtration to afford copolymers **P1** (29.6 mg, 61% yield) as red powders. GPC: M_n 7.5 kDa, M_w 22.6 kDa, $Đ$ 3.04. ^{1}H NMR (400 MHz, $CDCl_3$): δ 7.71, 7.49, 4.08, 1.77, 1.63, 1.50, 1.29, 0.88.

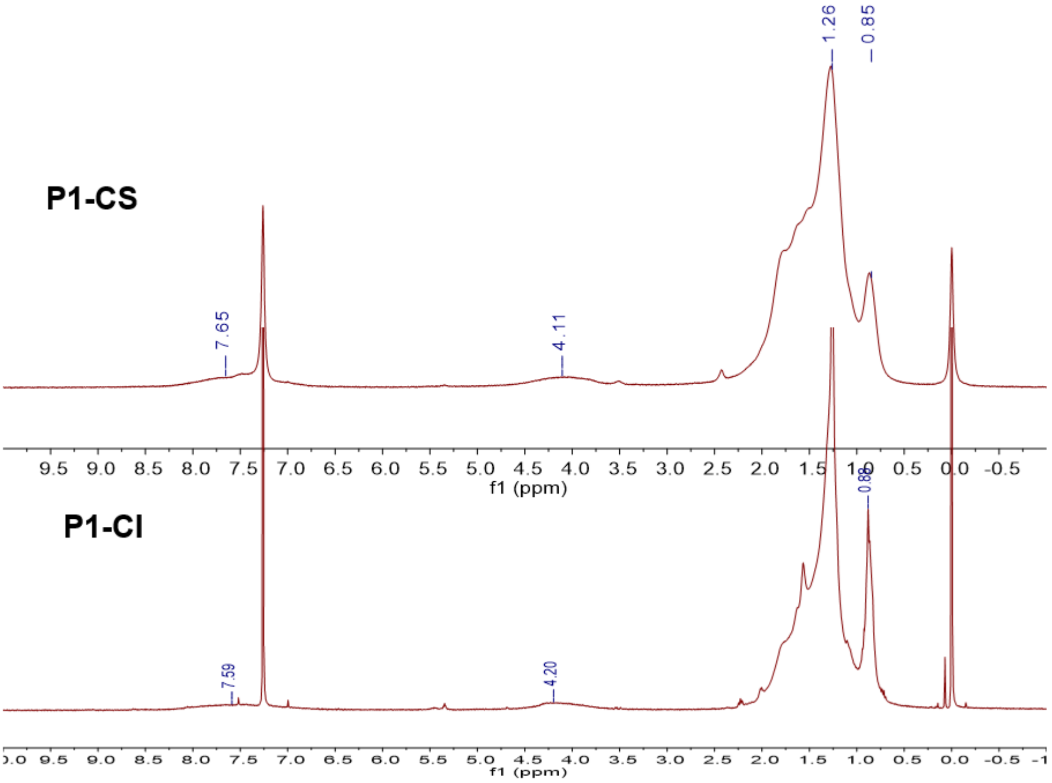

Supplementary Fig. 1. ^{1}H NMR spectra of **P1-CS** and **P1-CI** prepared by polycondensation via C-S cleavage and classic Stille coupling, respectively.

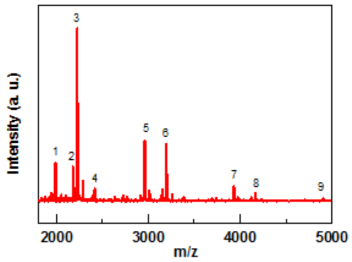

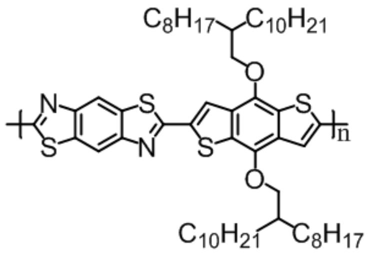

P1

1. (1990, m/z)=[BBTz-BDT]$_2$-SMe
2. (2180, m/z)=[BBTz-BDT]$_2$-BBTz-SMe
3. (2226, m/z)=[BBTz-BDT]$_2$-BBTz-2SMe
4. (2416, m/z)=[BBTz-BDT]$_2$-2BBTz-SMe (Defect)
5. (2961, m/z)=[BBTz-BDT]$_3$-SMe
6. (3197, m/z)=[BBTz-BDT]$_3$-BBTz-2SMe
7. (3931, m/z)=[BBTz-BDT]$_4$-SMe
8. (4167, m/z)=[BBTz-BDT]$_4$-BBTz-2SMe
9. (4902, m/z)=[BBTz-BDT]$_5$-SMe

Supplementary Fig. 2. MALDI–TOF mass spectra of **P1** (expansion from 1800 to 5000 m/z).

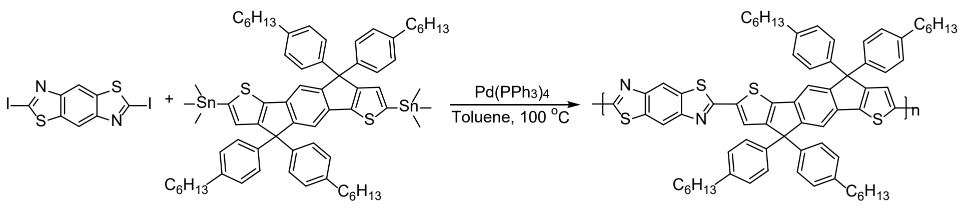

E5 **N2** **P2-CI**

P2-CI: In a Schlenk flask, **E5** (96.5 mg, 0.22 mmol), **N2** (268.0 mg, 0.22 mmol) and dry toluene (1.5 mL) were added under N_2 atmosphere. $Pd(PPh_3)_4$ (12.7 mg, 11.0 μmol) was added to the mixture. Then the mixture solution was stirred at 100 °C for 72 h. Afterwards, the reaction mixture was poured into methanol. The crude polymer was collected by filtration and purified by soxhlet extraction successively with acetone, hexane and chloroform. The chloroform solution was concentrated, poured into methanol, and then collected by filtration to afford a red powder (220.0 mg, 92% yield). GPC: M_n 10.2 kDa, M_w 15.2 kDa, $Đ$ 1.50. ^1H NMR (400 MHz, $CDCl_3$): δ 8.43, 8.27, 7.62, 7.51, 7.23, 7.09, 2.64, 2.53, 1.66, 1.56, 1.36, 1.26, 0.92, 0.84. $^{13}C\{^1H\}$ NMR (126 MHz, $CDCl_3$) δ 162.23, 157.08, 154.38, 151.79, 145.62, 142.11, 141.90, 141.70, 141.44, 141.22, 139.69, 138.88, 135.68, 134.45, 128.71, 128.61, 128.49, 127.98, 124.94, 118.32, 114.70, 63.26, 35.72, 31.86, 31.50, 29.85, 29.29, 22.74, 14.26. IR (film), ν (cm^{-1}): 3726, 3628, 2923, 2853, 1716, 1540, 1507, 1456, 1398, 1356, 1310, 1243, 1161, 1090, 1054, 1020, 886, 863, 807, 701. Analysis (calcd., found for $(C_{72}H_{74}N_2S_4)_n$): C (78.93, 78.89), H (6.86, 7.69), N (2.56, 2.22), S (11.70, 9.21). PLQY 30%.

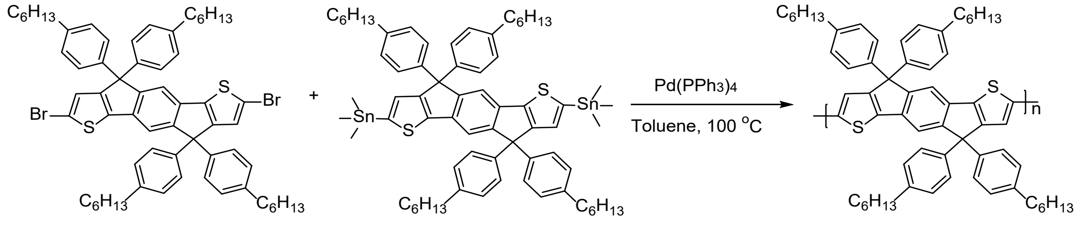

IDT-Br **N2** **PIDT**

PIDT: In a Schlenk flask, **IDT-Br** (53.3 mg, 0.05 mmol), **N2** (61.7 mg, 0.05 mmol), $Pd(PPh_3)_4$ (5.8 mg, 5.0 μmol) and dry toluene (2.0 mL) were added under N_2 atmosphere. Then the mixture solution was stirred at 100 °C for 72 h. After the reaction mixture was poured into methanol, the crude product was collected by filtration and purified by soxhlet extraction successively with acetone, hexane, chloroform and

chlorobenzene. The chloroform solution was concentrated, poured into methanol and then collected by filtration to afford a red solid (80 mg, 88% yield). GPC: M_n 31.0 kDa, M_w 58.3 kDa, $Đ$ 1.82. ^{1}H NMR (400 MHz, $CDCl_3$): δ 7.33, 7.16, 7.14, 7.07, 7.05, 2.56, 1.59, 1.29, 0.89. IR (film), ν (cm^{-1}): 3726, 3628, 2922, 2850, 1507, 1457, 1413, 1375, 1321, 1185, 1119, 1019, 864, 812, 722, 668. Analysis (calcd., found for $(C_{64}H_{72}S_2)_n$): C (84.90, 83.66), H (8.02, 7.86), N (0.00, 0.08), S (7.08, 7.02). PLQY 13%.

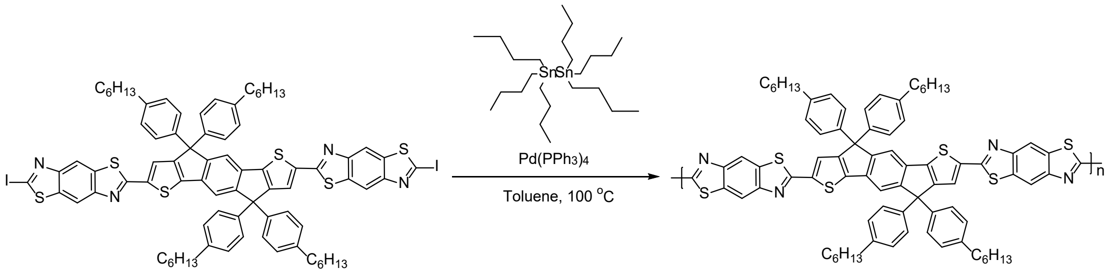

E7 **PBBT**

PBBT: In a Schlenk flask, compound **E6** (68.2 mg, 44.0 μmol), hexabutyldistannane (25.7 mg, 44.0 μmol), $Pd(PPh_3)_4$ (5.1 mg, 4.4 μmol) and dry toluene (2.0 mL) were added under N_2 atmosphere. Then the mixture solution was stirred at 100 °C for 72 h. Next, the reaction mixture was poured into methanol, the crude polymer was collected by filtration, and purified by successive soxhlet extractions with acetone, hexane, chloroform and chlorobenzene. The chloroform solution was concentrated, poured into methanol, which was collected by filtration to afford a red solid (57 mg, 49% yield). GPC: M_n 9.0 kDa, M_w 19.3 kDa, $Đ$ 2.15. ^{1}H NMR (400 MHz, $CDCl_3$): δ 8.57, 7.92, 7.61, 7.50, 7.21, 7.12, 7.11, 2.58, 1.50, 1.29, 0.85. IR (film), ν (cm^{-1}): 3735, 2921, 2855, 2160, 2032, 1533, 1507, 1461, 1413, 1396, 1363, 1309, 1260, 1160, 1054, 1019, 891, 861, 809, 702. Analysis (calcd., found for $(C_{80}H_{76}N_4S_6)_n$): C (74.73, 70.17), H (5.96, 5.96), N (4.36, 3.78), S (14.96, 12.94). PLQY 18%.

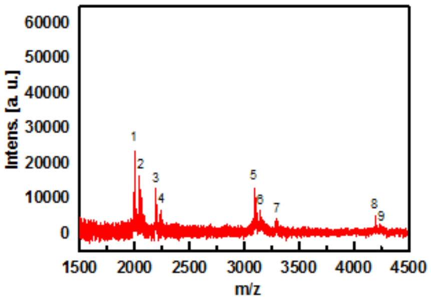

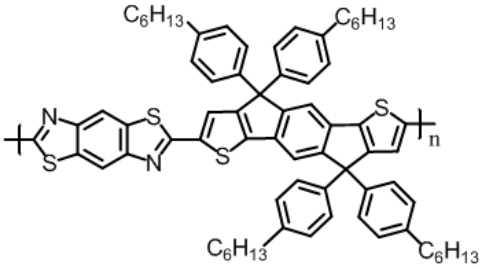

P2

1. (2002, m/z)=[BBTz-IDT]$_1$-IDT
2. (2048, m/z)=[BBTz-IDT]$_1$-IDT-SMe (Defect)
3. (2192, m/z)=[BBTz-IDT]$_2$
4. (2238, m/z)=[BBTz-IDT]$_2$-SMe
5. (3097, m/z)=[BBTz-IDT]$_2$-IDT
6. (3143, m/z)=[BBTz-IDT]$_2$-IDT-SMe (Defect)
7. (3286, m/z)=[BBTz-IDT]$_3$
8. (4191, m/z)=[BBTz-IDT]$_3$-IDT
9. (4237, m/z)=[BBTz-IDT]$_3$-IDT-SMe (Defect)

Supplementary Fig. 3. MALDI–TOF mass spectra of **P2** (expansion from 1500 to 4500 m/z)

II-6. Substrates scope on polymerization

General synthetic procedure: In a Schlenk flask, **E1-E4** (0.05 mmol), **N1-N9** (0.05 mmol), CuTc (0.25 mmol) and $Pd(PPh_3)_4$ (5.0 μmol) were added in dry toluene (2.0 mL) under N_2 atmosphere. Then the mixture solution was stirred at room temperature for 24 h (**P8** for 72 h). After the reaction mixture was washed with ammonium hydroxide and water, the organic phase was concentrated and poured into methanol. The crude polymer was then collected by filtration and purified by soxhlet extraction with acetone, hexane, chloroform and chlorobenzene, successively. The chloroform and chlorobenzene solution was concentrated, poured into methanol and collected by filtration to afford copolymers, respectively.

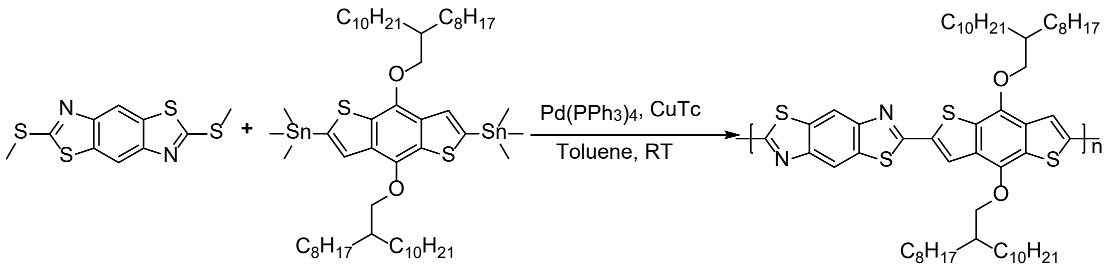

E1 **N1** **P1 (P1-CS)**

P1 (P1-CS): The chloroform solution was concentrated, poured into methanol and the resulting precipitate then collected by filtration to afford a dark-red solid (41.0 mg, 84% yield). ^{1}H NMR (400 MHz, $CDCl_3$): δ 7.71, 7.49, 4.08, 1.77, 1.63, 1.50, 1.29, 0.88. IR (film), ν (cm^{-1}): 2920, 2849, 1553, 1465, 1426, 1386, 1358, 1310, 1262, 1163, 1034, 880, 855, 829, 715, 690, 607. Analysis (calcd., found for $(C_{58}H_{88}N_2O_2S_4)_n$): C (71.70, 72.29), H (8.92, 9.18), N (2.88, 2.60), S (13.20, 12.21). PLQY 1%.

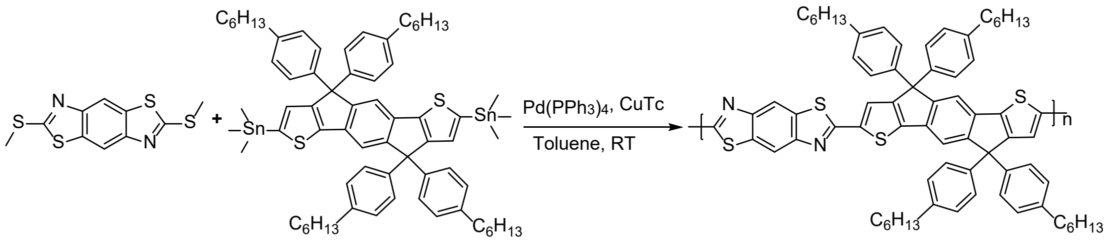

E1 **N2** **P2 (P2-CS)**

P2 (P2-CS): The chloroform solution was concentrated, poured into methanol and the resulting precipitate then collected by filtration to afford a dark-red solid (45.0 mg, 82% yield). GPC: M_n 13.4 kDa, M_w 44.2 kDa, $Đ$ 3.29. ^{1}H NMR (400 MHz, $CDCl_3$): δ 8.43, 8.27, 7.62, 7.51, 7.23, 7.09, 2.64, 2.53, 1.66, 1.56, 1.36, 1.26, 0.92, 0.84. $^{13}C\{^1H\}$NMR (126 MHz, $CDCl_3$) δ 162.23, 157.08, 154.38, 151.79, 145.62, 142.11, 141.90, 141.70, 141.44, 141.22, 139.69, 138.88, 135.68, 134.45, 128.71, 128.61, 128.49, 127.98, 124.94, 118.32, 114.70, 63.26, 35.72, 31.86, 31.50, 29.85, 29.29, 22.74, 14.26. IR (film), ν (cm^{-1}): 3853, 3735, 3649, 2921, 2852, 1717, 1540, 1507, 1457, 1398, 1362, 1309, 1244, 1162, 1054, 1020, 887, 861, 808, 701. Analysis (calcd., found for $(C_{72}H_{74}N_2S_4)_n$): C (78.93, 78.66), H (6.86, 7.25), N (2.56, 2.09), S (11.70, 10.75). PLQY 36%.

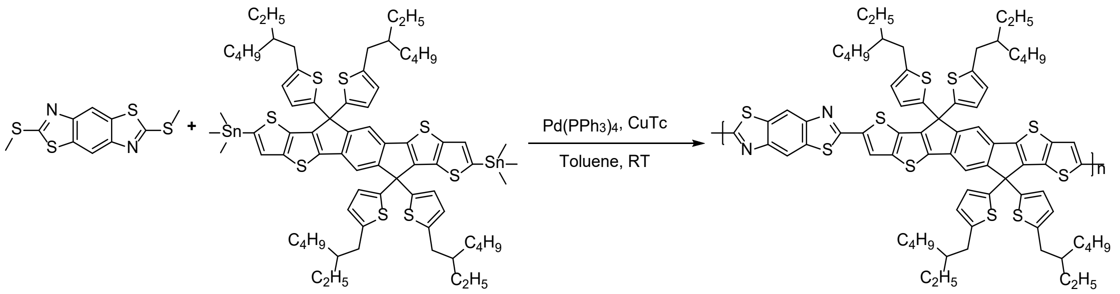

E1 N3 P3

P3: The chloroform solution and the chlorobenzene solution were concentrated, poured into methanol and the resulting precipitate then collected by filtration to afford a black solid.

Chloroform fraction: (43.7 mg, 65% yield). GPC: M_n 16.4 kDa, M_w 64.6 kDa, $Đ$ 3.94. ^{1}H NMR (400 MHz, $CDCl_3$): δ 8.41, 7.90, 7.78, 6.84, 6.57, 2.67, 1.60, 1.25, 0.85. ^{13}C{^{1}H} NMR (176 MHz, C_2D2Cl_4) δ 161.86, 154.19, 151.84, 146.44, 144.68, 144.30, 142.30, 138.20, 136.57, 135.14, 134.51, 131.89, 131.50, 128.24, 125.77, 124.15, 121.79, 117.14, 114.71, 41.29, 34.48, 32.59, 28.81, 25.97, 22.67, 13.68, 10.70. IR (film), ν (cm^{-1}): 3073, 2923, 2852, 1529, 1458, 1426, 1366, 1306, 1241, 1149, 1025, 920, 859, 797, 712, 658, 604. Analysis (calcd., found for $(C_{76}H_{82}N_2S_{10})_n$): C (67.91, 66.60), H (6.15, 6.21), N (2.08, 2.12), S (23.85, 23.37). PLQY 26%

Chlorobenzene fraction: (14.8 mg, 22% yield). GPC: M_n 34.7 kDa, M_w 109.2 kDa, $Đ$ 3.15. ^{1}H NMR (400 MHz, CD_5Cl): δ 8.33, 8.17, 7.38, 6.72, 6.54, 2.56, 1.46, 1.16, 0.82. IR (film), ν (cm^{-1}): 3072, 2954, 2919, 2853, 1529, 1456, 1425, 1366, 1307, 1241, 1149, 1053, 1028, 920, 860, 796, 714, 660, 604. Analysis (calcd., found for $(C_{76}H_{82}N_2S_{10})_n$): C (67.91, 66.44), H (6.15, 6.18), N (2.08, 2.12), S (23.85, 23.45).

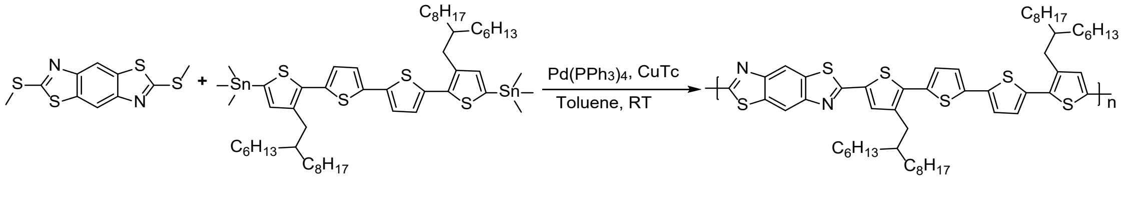

E1 N4 P4

P4: The chlorobenzene solution was concentrated, poured into methanol and the resulting precipitate was then collected by filtration to afford a glossy black solid (29.1

mg, 60% yield). GPC: M_n 30.5 kDa, M_w 64.8 kDa, $Đ$ 2.13. ^{1}H NMR (400 MHz, CD_5Cl): δ 8.18, 7.53, 2.86, 1.71, 1.31, 0.90. IR (film), ν (cm^{-1}): 2920, 2850, 1560, 1510, 1490, 1420, 1380, 1310, 1250, 1180, 1050, 881, 851, 778, 719, 689, 602. Analysis (calcd., found for $(C_{56}H_{74}N_2S_6)_n$): C (69.52, 69.12), H (7.71, 7.44), N (2.90, 3.03), S (19.88, 19.26). PLQY 15%.

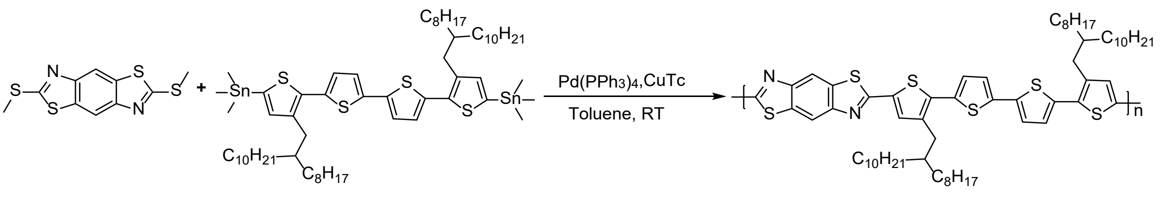

E1 N5 P5

P5: The chloroform solution and the chlorobenzene solution were concentrated, the resulting precipitate poured into methanol and then collected by filtration to afford a glossy-black solid.

Chloroform fraction: (8.1 mg, 15% yield). GPC: M_n 13.1 kDa, M_w 52.2 kDa, $Đ$ 3.97. ^{1}H NMR (400 MHz, $CDCl_3$): δ 8.35, 7.17, 2.75, 1.74, 1.59, 1.25, 0.87. IR (film), ν (cm^{-1}): 2921, 2849, 1553, 1512, 1488, 1457, 1420, 1375, 1308, 1256, 1185, 1053, 881, 852, 783, 720, 690, 602. Analysis (calcd., found for $(C_{64}H_{90}N_2S_6)_n$): C (71.19, 69.29), H (8.40, 8.01), N (2.59, 2.71), S (17.81, 17.37). PLQY 7%.

Chlorobenzene fraction: (44.9 mg, 83% yield). GPC: M_n 45.0 kDa, M_w 150.9 kDa, $Đ$ 3.35. ^{1}H NMR (400 MHz, CD_5Cl): δ 8.31, 7.56, 2.86, 2.53, 1.30, 0.91. IR (film), ν (cm^{-1}): 2920, 2849, 1557, 1512, 1488, 1455, 1420, 1376, 1308, 1256, 1189, 980, 881, 852, 785, 718, 678, 603. Analysis (calcd., found for $(C_{64}H_{90}N_2S_6)_n$): C (71.19, 68.38), H (8.40, 8.04), N (2.59, 2.72), S (17.81, 17.40).

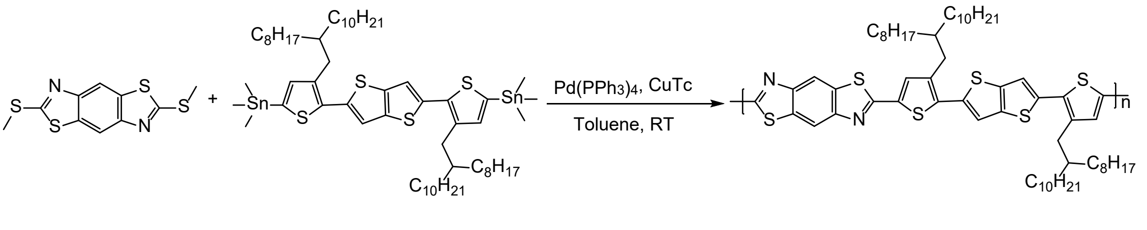

E1 N6 P6

P6: The chlorobenzene solution was contentrated, poured into methanol, and the

resulting precipitate then collected by filtration to afford a glossy black solid (26.4 mg, 50% yield). GPC: M_n 18.5 kDa, M_w 44.9 kDa, $Đ$ 2.43. ^{1}H NMR (400 MHz, $CDCl_3$): δ 8.38, 7.46, 2.79, 1.79, 1.25, 0.87. IR (film), ν (cm^{-1}): 2918, 2847, 1557, 1508, 1463, 1417, 1373, 1307, 1258, 1182, 1096, 1054, 1020, 978, 884, 857, 721, 684, 607. Analysis (calcd., found for $(C_{62}H_{88}N_2S_6)_n$): C (70.67, 66.82), H (8.42, 7.84), N (2.66, 2.44), S (18.25, 16.00). PLQY 5%

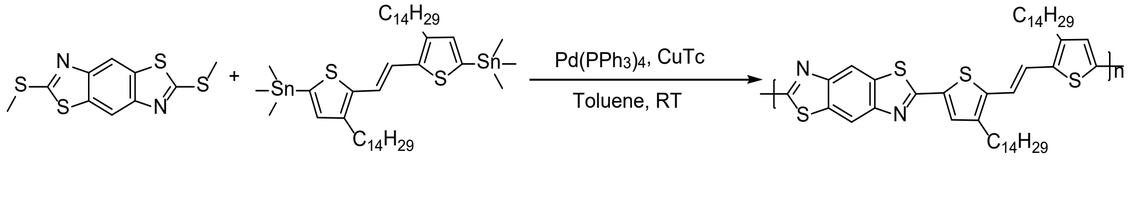

E1 N7 P7

P7: The chlorobenzene solution and chloroform solution were poured into methanol and the the resulting precipitates then collected by filtration to afford black solids, respectively.

Chloroform fraction: (23.3 mg, 60% yield). GPC: M_n 9.2 kDa, M_w 15.4 kDa, $Đ$ 1.67. ^{1}H NMR (400 MHz, $CDCl_3$): δ 7.49, 7.01, 3.30, 3.06, 2.68, 2.43, 1.25, 0.87. IR (film), ν (cm^{-1}): 2916, 2849, 1547, 1499, 1464, 1423, 1385, 1308, 1255, 1179, 1095, 1055, 1024, 923, 882, 853, 748, 718, 605. Analysis (calcd., found for $(C_{46}H_{64}N_2S_4)_n$): C (71.45, 67.34), H (8.34, 7.86), N (3.62, 3.17), S (16.58, 15.54). PLQY: 1%.

Chlorobenzene fraction:: (6.6 mg, 17% yield). GPC: M_n 12.9 kDa, M_w 21.6 kDa, $Đ$ 1.68. ^{1}H NMR (400 MHz, CD_5Cl): δ 7.85, 6.74, 3.27, 2.62, 1.31, 0.91. IR (film), ν (cm^{-1}): 2919, 2848, 1551, 1501, 1464, 1422, 1385, 1308, 1255, 1178, 1096, 1054, 1025, 923, 880, 853, 718, 605. Analysis (calcd., found for $(C_{46}H_{64}N_2S_4)_n$): C (71.45, 67.74), H (8.34, 7.91), N (3.62, 3.20), S (16.58, 15.66).

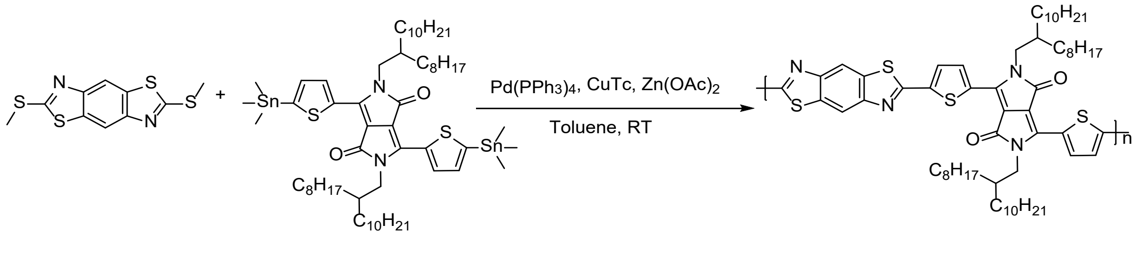

E1 N8 P8

P8: The chloroform solution was contentrated and poured into methanol and the

resulting precipitate then collected by filtration to afford a deep blue solid (47.9 mg, 91% yield). GPC: M_n 11.9 kDa, M_w 53.6 kDa, $Đ$ 4.52. ^{1}H NMR (500 MHz, $C_2D_2Cl_4$): δ 8.90, 7.55, 4.03, 2.43, 1.24, 0.82. IR (film), ν (cm^{-1}): 2918, 2850, 1664, 1544,1478, 1456, 1404, 1310, 1246, 1221, 1103, 1054, 1025, 893, 857, 811, 731, 699, 638, 609. Analysis (calcd., found for $(C_{62}H_{88}N_4O_2S_4)_n$): C (70.95, 68.89), H (8.45, 8.25), N (5.34, 4.67), S (12.22, 11.40).

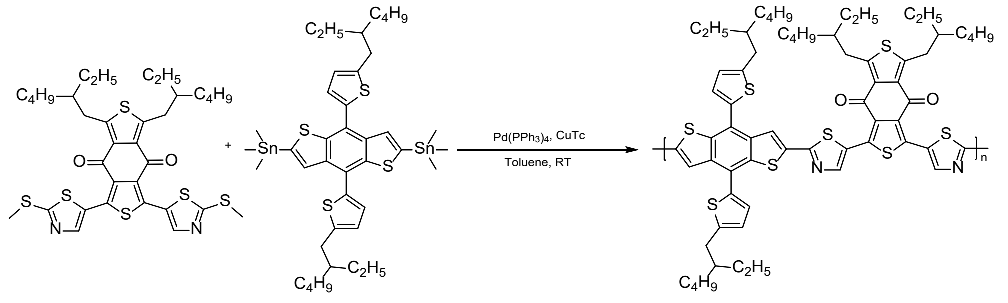

E2 N9 P9

P9: The chlorobenzene solution and chloroform solution were poured into methanol and the the resulting precipitates then collected by filtration to afford black solids, respectively.

Chloroform fraction: (23.3 mg, 60% yield). GPC: M_n 9.2 kDa, M_w 15.4 kDa, $Đ$ 1.67. ^{1}H NMR (400 MHz, $CDCl_3$): δ 7.67, 7.50, 7.05, 6.95, 2.91, 1.61, 1.39, 0.97. IR (film), ν (cm^{-1}): 2953, 2919, 2854, 1648, 1487, 1458, 1363, 1282, 1145, 1073, 850, 797, 707, 665, 634, 543. Analysis (calcd., found for $(C_{46}H_{64}N_2S_4)_n$): C (66.85, 66.04), H (6.46, 6.29), N (2.36, 2.19), S (21.63, 21.03). PLQY 1%.

Chlorobenzene fraction:: (6.6 mg, 17% yield). GPC: M_n 12.9 kDa, M_w 21.6 kDa, $Đ$ 1.68. ^{1}H NMR (400 MHz, CD_5Cl): δ 7.77, 2.95, 1.44, 1.06. IR (film), ν (cm^{-1}): 2955, 2920, 2854, 1648, 1486, 1457, 1363, 1281, 1146, 1075, 850, 800, 708, 636, 548. Analysis (calcd., found for $(C_{46}H_{64}N_2S_4)_n$): C (66.85, 66.05), H (6.46, 6.31), N (2.36, 2.23), S (21.63, 21.18).

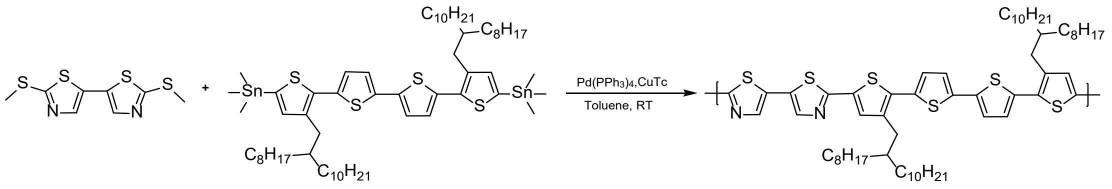

E3 **N5** **P10**

P10: The chloroform solution was contentrated and poured into methanol and the resulting precipitate then collected by filtration to afford a glossy-black solid (47.0 mg, 89% yield). GPC: M_n 17.2 kDa, M_w 28.8 kDa, $Đ$ 1.67. ^{1}H NMR (500 MHz, $CDCl_3$): δ 7.72, 7.26, 7.08, 2.68, 1.24, 0.88. IR (film), ν (cm^{-1}): 2920, 2850, 1610, 1553, 1463, 1374, 1260, 1140, 1071, 1023, 926, 835, 782, 719, 623, 545. Analysis (calcd., found for $(C_{62}H_{90}N_2S_6)_n$): C (70.53, 67.45), H (8.59, 8.46), N (2.65, 2.53), S (18.22, 17.06). PLQY 6%.

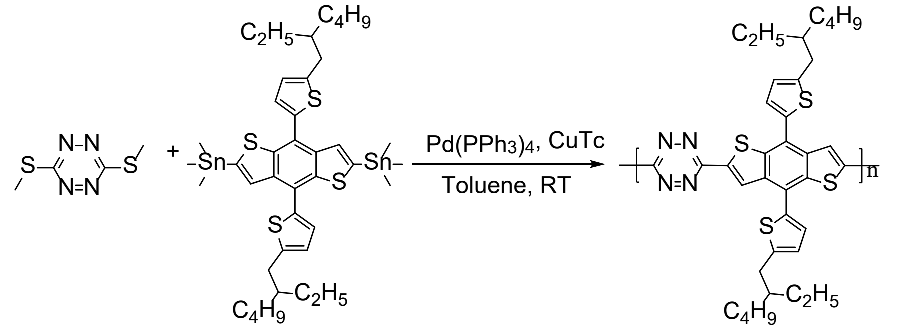

E4 **N9** **P11**

P11: The chloroform solution was contentrated and poured into methanol and then collected by filtration to afford a gloss black solid (21.0 mg, 64% yield). GPC: M_n 9.7 kDa, M_w 16.4 kDa, $Đ$ 1.69. ^{1}H NMR (500 MHz, $CDCl_3$): δ 7.33, 7.24, 6.89, 2.95, 1.81, 1.60, 1.51, 1.43, 1.05, 0.96. IR (film), ν (cm^{-1}): 2955, 2920, 2853, 1457, 1377, 1309, 1257, 1175, 1071, 1019, 887, 820, 797, 710, 662, 608. Analysis (calcd., found for $(C_{36}H_{40}N_4S_4)_n$): C (65.82, 69.84), H (6.14, 6.87), N (8.53, 0.88), S (19.52, 20.35). PLQY 1%.

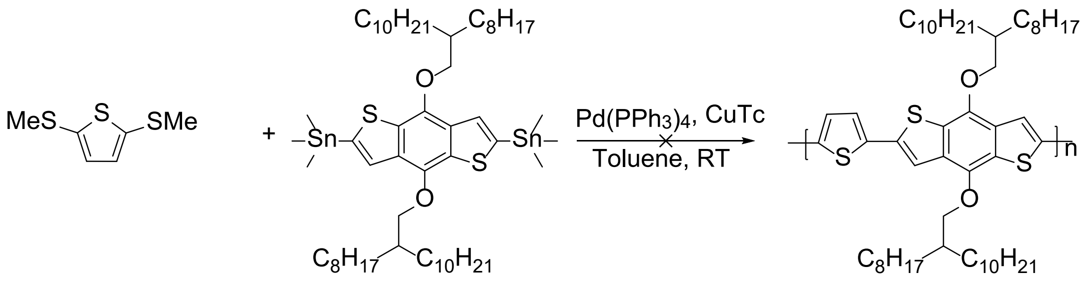

2,5-bis(methylsulfanyl)thiophene N1

In a Schlenk flask under an N_2 atmosphere, monomer 2,5-bis(methylsulfanyl)thiophene (8.7 mg, 0.05 mmol) and monomer **N1** (55.4 mg, 0.05 mmol) were dissolved in dry toluene (2.0 mL). CuTc (47.7 mg, 0.25 mmol), and $Pd(PPh_3)_4$ (5.8 mg, 5.0 μmol) were added to the mixture, which was then stirred at room temperature under N_2 atmosphere. After 3 days, the solution remain colorless. After quenching the reaction with methanol, the reaction mixture was poured into methanol, and no precipitation is formed.

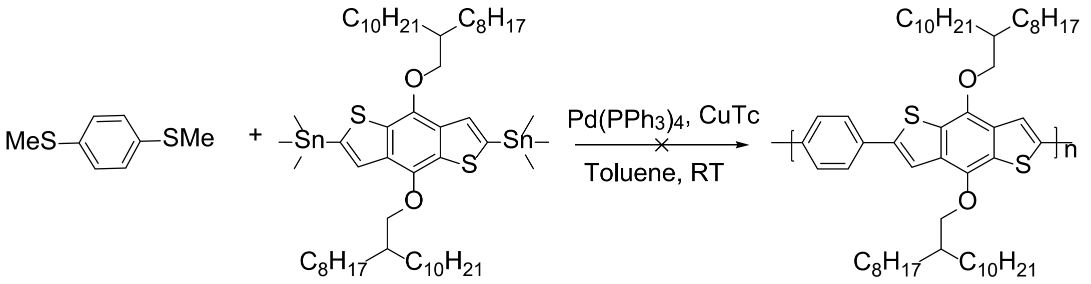

1,4-bis(methylsulfanyl)benzene N1

In a Schlenk flask under an N_2 atmosphere, monomer 1,4-bis(methylsulfanyl)benzene (8.5 mg, 0.05 mmol) and monomer **N1** (55.4 mg, 0.05 mmol) were dissolved in dry toluene (2.0 mL). CuTc (47.7 mg, 0.25 mmol), and $Pd(PPh_3)_4$ (5.8 mg, 5.0 μmol) were added to the mixture, which was then stirred at room temperature under N_2 atmosphere. After 3 days, the solution remain colorless. After quenching the reaction with methanol, the reaction mixture was poured into methanol, and no precipitation is formed.

III. Mechanistic studies

GC-MS Assay Parameters and Reaction Times:

2,2'-bithiophene, retention time 7.20 min

Anthracene, retention time 12.20 min;

2-(thiophen-2-yl)benzo[*d*]thiazole, retention time 14.43 min;

2-(3-methoxythiophen-2-yl)benzo[*d*]thiazole, retention time 17.37 min;

methyl 2-(benzo[*d*]thiazol-2-yl)thiophene-3-carboxylate, retention time 18.09 min.

2,2'-bibenzo[*d*]thiazole, retention time 19.93 min.

III-1. Competition reactions

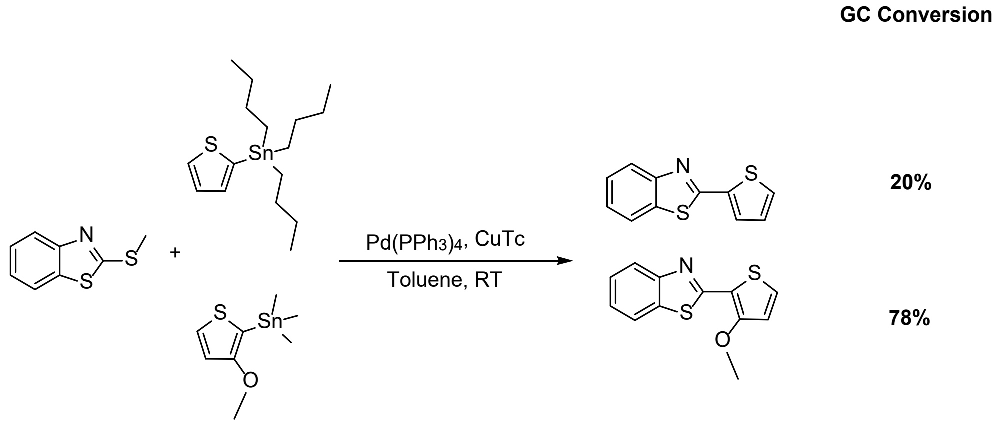

Under an N_2 atmosphere, a mixture of compound MTBT (18.1 mg, 0.10 mmol), tributyl(thiophen-2-yl)stannane (37.3 mg, 0.10 mmol), (3-methoxythiophen-2-yl)trimethylstannane (33.2 mg, 0.10 mmol), CuTc (47.7 mg, 0.25 mmol) and $Pd(PPh_3)_4$ (5.8 mg, 5.0 μmol) were dissolved in dry toluene (10.0 mL). And then the mixture was stirred at room temperature for 24 h. Afterwards, anthracene (17.8 mg, 0.10 mmol) in toluene (10 mL) was added to the reaction solution. The solution was filtered through Celite, washed with DCM and ether (1:1 *v/v*) and analyzed via GC versus anthracene which served as an internal standard.

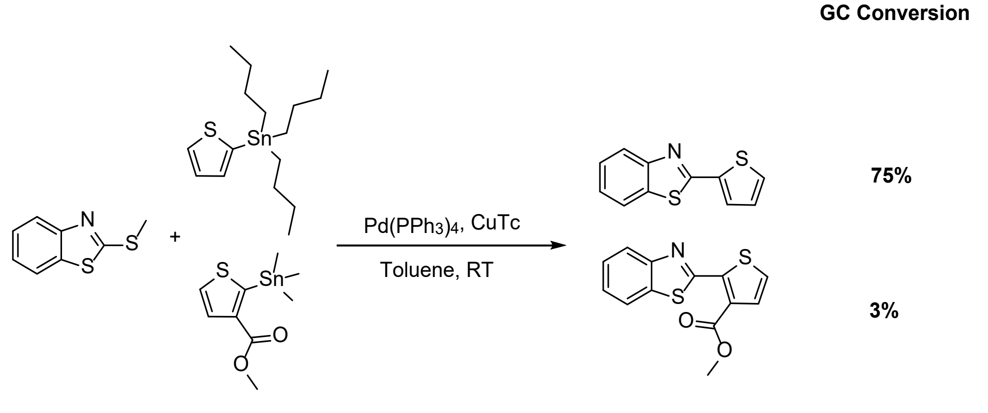

Under an N_2 atmosphere, a mixture of compound MTBT (18.1 mg, 0.10 mmol), tributyl(thiophen-2-yl)stannane (37.3 mg, 0.10 mmol), methyl 2-(trimethylstannyl)thiophene-3-carboxylate (30.5 mg, 0.10 mmol), CuTc (47.7 mg, 0.25

mmol) and Pd(PPh_3)$_4$ (5.8 mg, 5.0 μmol) were dissolved in dry toluene (10.0 mL). And then the mixture was stirred at room temperature for 24 h. Afterwards, anthracene (17.8 mg, 0.10 mmol) in toluene (10 mL) was added to the solution. The solution was filtered through Celite, washed with DCM and ether (1:1 *v/v*) and analyzed via GC versus anthracene which served as an internal standard.

III-2. Control experiments

GC Conversion

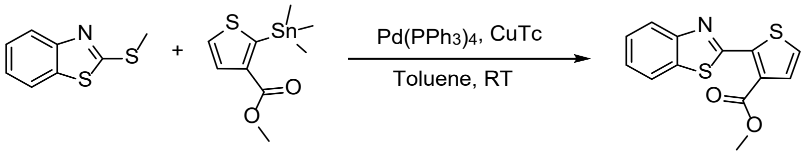

28%

Under an N_2 atmosphere, a mixture of compound MTBT (65.4 mg, 0.36 mmol), methyl 2-(trimethylstannyl)thiophene-3-carboxylate (110.0 mg, 0.36 mmol), CuTc (171.6 mg, 0.90 mmol) and Pd(PPh_3)$_4$ (20.8 mg, 18.0 μmol) were dissolved in dry toluene (15.0 mL). And then the mixture was stirred at room temperature for 24 h. After the reaction was complete, anthracene (64.2 mg, 0.36 mmol) in 30 mL of toluene was added to the solution. The solution was filtered through Celite, washed with DCM and ether (1:1 *v/v*) and analyzed via GC versus anthracene which served as an internal standard.

GC Conversion

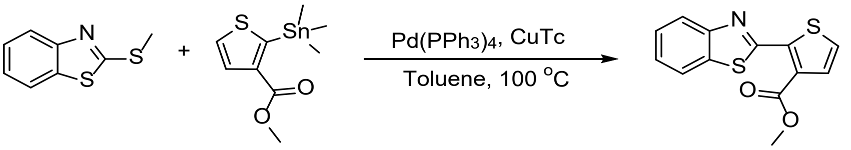

33%

Under N_2 atmosphere, a mixture of compound MTBT (63.6 mg, 0.35 mmol), methyl 2-(trimethylstannyl)thiophene-3-carboxylate (107.0 mg, 0.35 mmol), CuTc (167.8 mg, 0.88 mmol) and Pd(PPh_3)$_4$ (20.3 mg, 18.0 μmol) were dissolved in dry toluene (15.0 mL). And then the mixture was stirred at 100 °C for 24 h. After cooling to room temperature, anthracene (62.4 mg, 0.35 mmol) in toluene (10 mL) was added into the solution. The solution was filtered through Celite, washed with DCM and ether (1:1 *v/v*)

and analyzed via GC versus anthracene which served as an internal standard.

GC Conversion

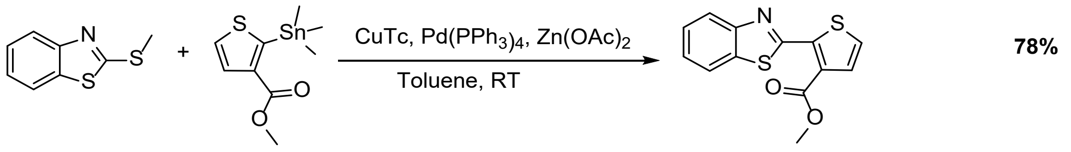

Under N_2 atmosphere, a mixture of compound MTBT (210.0 mg, 1.16 mmol), methyl 2-(trimethylstannyl)thiophene-3-carboxylate (353.8 mg, 1.16 mmol), CuTc (552.0 mg, 2.89 mmol)，$Zn(OAc)_2$ (21.3 mg, 0.12 mmol) and $Pd(PPh_3)_4$ (67.0 mg, 58.0 μmol) were dissolved in dry toluene (20.0 mL). And then the mixture was stirred at room temperature for 24 h. Afterwards, anthracene (206.7 mg, 1.16 mmol) in toluene (10 mL) was added into the solution. The solution was filtered through Celite, washed with DCM and ether (1:1 *v/v*) and analyzed via GC versus anthracene which served as an internal standard.

III-3. Defect analysis

GC Convertion

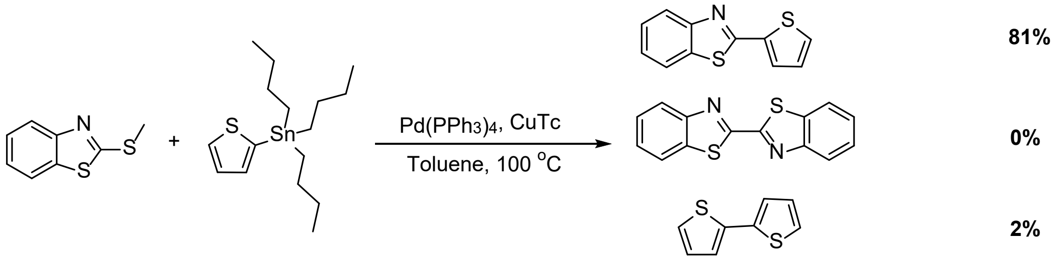

Under an N_2 atmosphere, a mixture of compound MTBT (139.0 mg, 0.37 mmol), tributyl(thiophen-2-yl)stannane (286.2 mg, 0.37 mmol), CuTc (365.6 mg, 1.92 mmol) and $Pd(PPh_3)_4$ (44.3 mg, 37.0 μmol) were dissolved in dry toluene (15.0 mL). And then the mixture was stirred at 100 °C for 24 h. Afterwards, anthracene (65.9 mg, 0.37 mmol) in toluene (10 mL) was added into the solution. The solution was filtered through Celite, washed with DCM and ether (1:1 *v/v*) and analyzed via GC versus anthracene which served as an internal standard.

GC Conversion

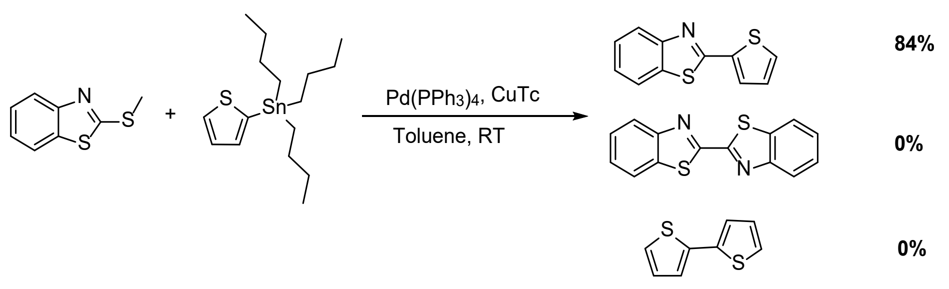

Under N_2 atmosphere, a mixture of MTBT (185.2 mg, 1.02 mmol), tributyl(thiophen-2-yl)stannane (381.3 mg, 1.02 mmol), CuTc (487.1 mg, 2.55 mmol) and $Pd(PPh_3)_4$ (115.6 mg, 0.10 mmol) were dissolved in dry toluene (20.0 mL). And then the mixture was stirred at room temperature for 24 h. Afterwards, anthracene (210.8 mg, 1.02 mmol) in toluene (10 mL) was added into the solution. The solution was filtered through Celite, washed with DCM and ether (1:1 *v/v*) and analyzed via GC versus anthracene which served as an internal standard.

GC Conversion

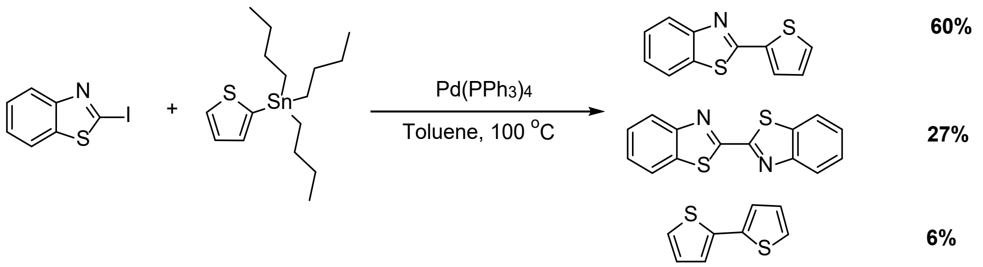

Under N_2 atmosphere, a mixture of 2-iodobenzo[*d*]thiazole (342.1 mg, 1.31 mmol), tributyl(thiophen-2-yl)stannane (489.0 mg, 1.31 mmol) and $Pd(PPh_3)_4$ (75.7 mg, 65 μmol) were dissolved in dry toluene (15.0 mL). And then the mixture was stirred at 100 °C for 24 h. Afterwards, anthracene (233.5 mg, 1.31 mmol) in toluene (10 mL) was added into the solution. The solution was filtered through Celite, washed with DCM and ether (1:1 *v/v*) and analyzed via GC versus anthracene which served as an internal standard.

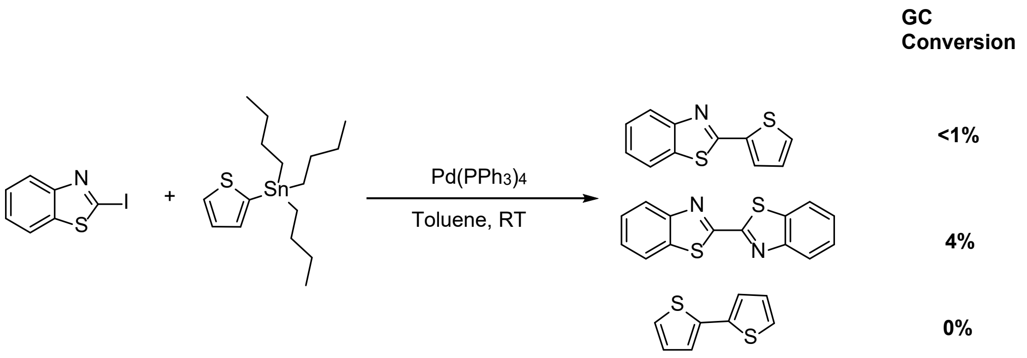

Under an N_2 atmosphere, a mixture of 2-iodobenzo[d]thiazole (354.3 mg, 1.36 mmol), tributyl(thiophen-2-yl)stannane (506.5 mg, 1.36 mmol) and $Pd(PPh_3)_4$ (78.4 mg, 68.0 μmol) were dissolved in dry toluene (20.0 mL). And then the mixture was stirred at room temperature for 24 h. Afterwards, anthracene (242.4 mg, 1.36 mmol) in toluene (10 mL) was added into the solution. The solution was filtered through Celite, washed with DCM and ether (1:1 *v/v*) and analyzed via GC versus anthracene which served as an internal standard.

IV. Characterization

IV-1 Optical and electrochemical properties

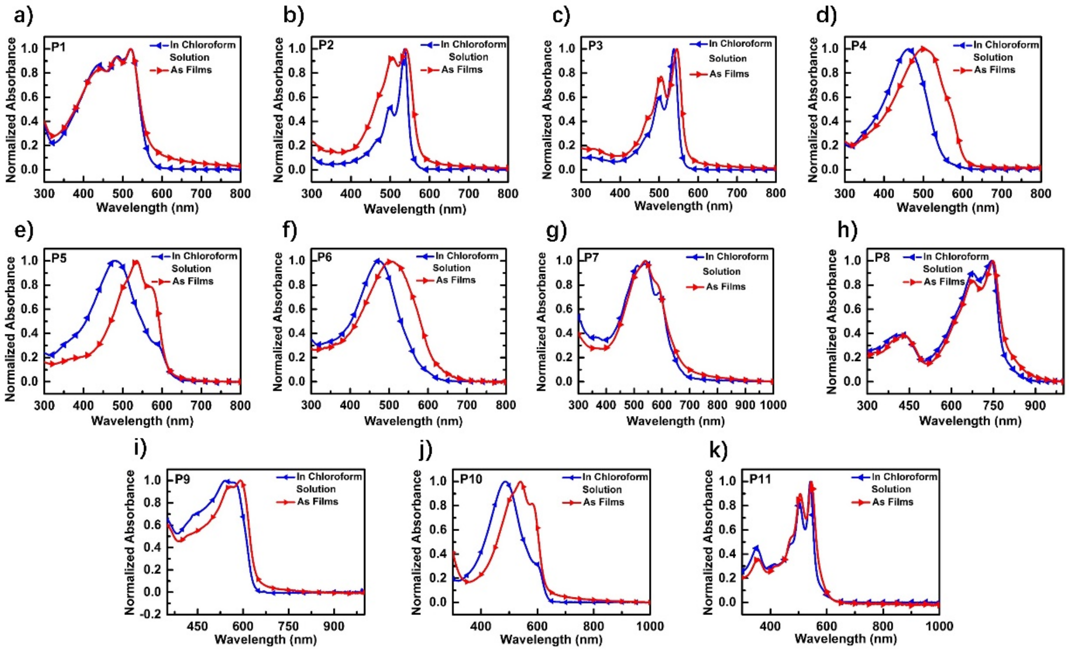

Supplementary Fig. 4. UV–vis–NIR absorption spectra of **P1-P11** in chloroform solution and as films.

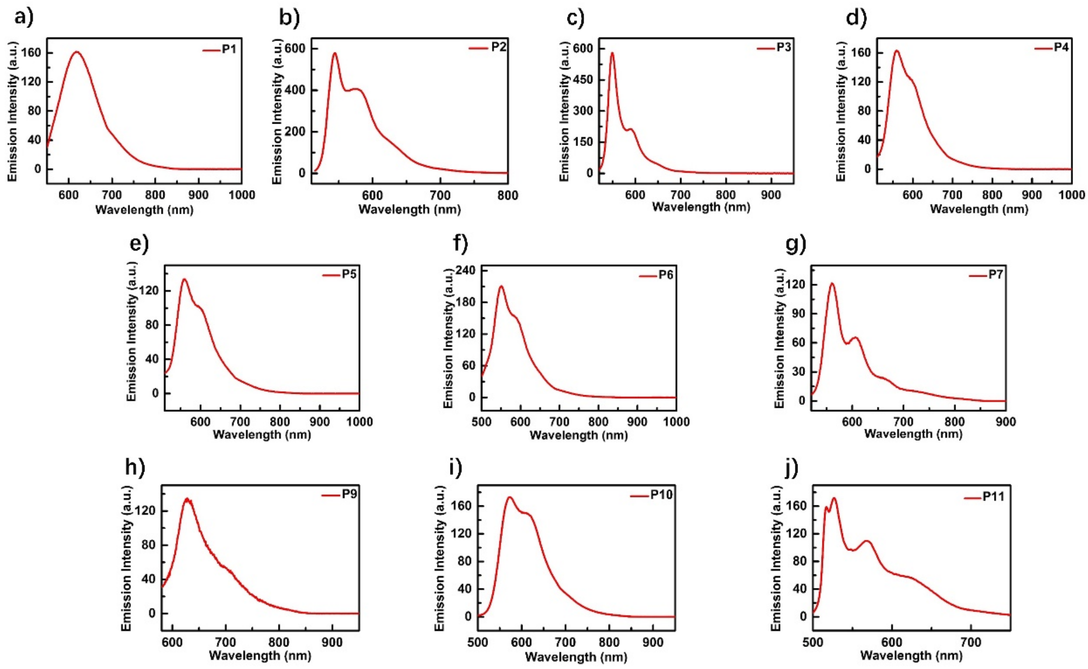

Supplementary Fig. 5. Steady-state photo-luminescence (PL) spectra of **P1-P7, P9 -P11** in chloroform solution (λ_{ex} = 527 nm , 498 nm, 498 nm, 482 nm, 482 nm, 473 nm, 500 nm, 540 nm , 483 nm and 514 nm for **P1-P7, P9 -P11**, respectively).

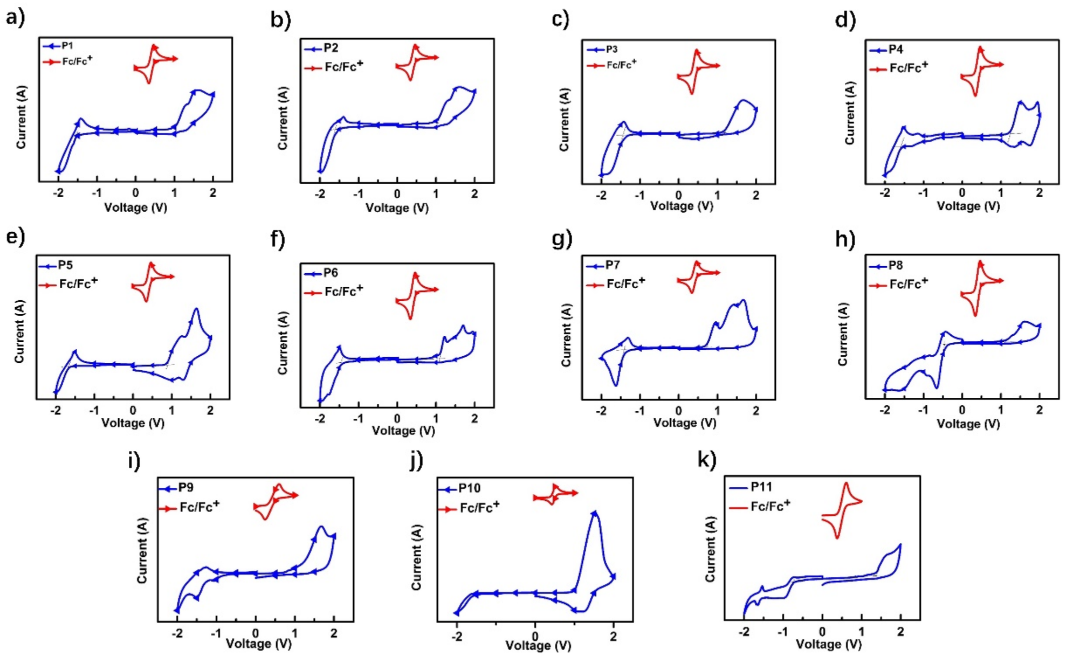

Supplementary Fig. 6 Cyclic voltammograms of **P1-P11** thin films in CH_3CN/0.1 M $[n\text{-}Bu_4N]^+[PF_6]^-$ with ferrocenium/ferrocene as an internal standard at 50 mV s^{-1}. The horizontal scale refers to an anodized Ag wire pseudoreference electrode.

IV-2. Thermal properties

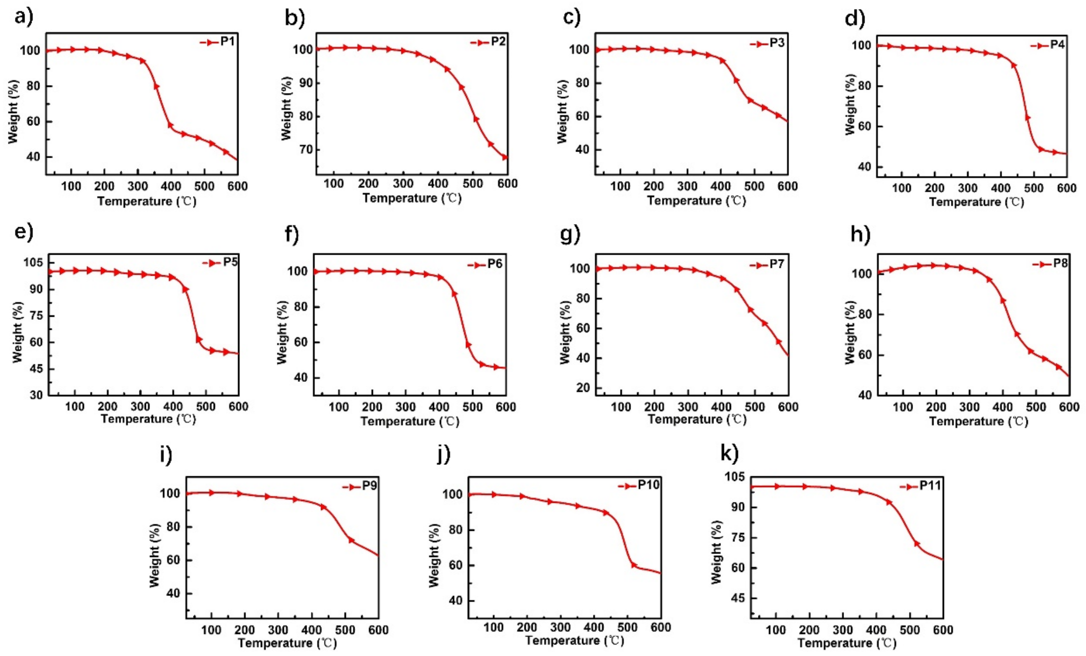

Supplementary Fig. 7. Thermogravimetric analysis (TGA) of polymers **P1-P11**.

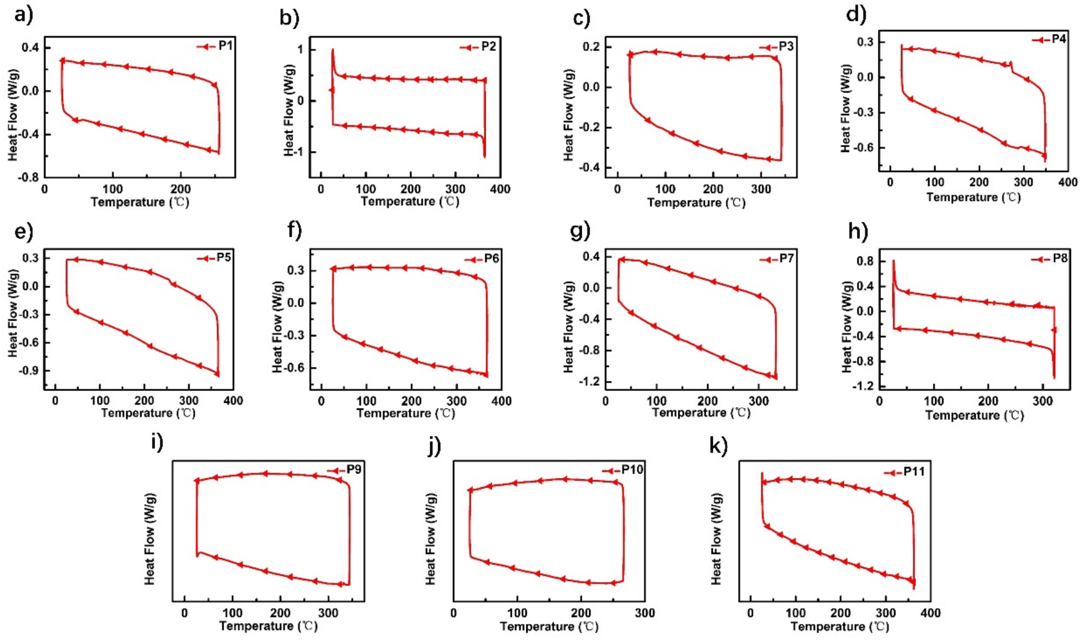

Supplementary Fig. 8. Differential scanning calorimetry (DSC) of polymers **P1-P11**.

Supplementary Table 3. Optical and electrochemical properties.

	Absorbance[a]		Emission[b]		CV[c] Versus ferrocene				TGA[d]
	λ_{sol} (nm)	λ_{film} (nm)	λ_{sol} (nm)	Φ (%)	E_{red} (V)	E_{ox} (eV)	LUMO (eV)	HOMO (eV)	T_d (°C)
**P1**	520	520	616	1	-1.93	0.62	-2.87	-5.42	328
**P2**	536	539	545	36	-2.00	0.64	-2.80	-5.44	416
**P3**	538	545	549	26	-1.84	0.78	-2.96	-5.58	393
**P4**	462	498	559	15	-1.99	0.57	-2.81	-5.37	399
**P5**	481	535	559	7	-2.07	0.48	-2.73	-5.28	415
**P6**	473	501	551	5	-1.86	0.70	-2.94	-5.50	418
**P7**	540	545	580	1	-1.81	0.33	-2.99	-5.13	384
**P8**	743	748	-	-	-1.18	0.45	-3.62	-5.25	373
**P9**	542	590	627	1	-0.88	1.17	-3.50	-5.55	395
**P10**	487	540	572	6	-1.58	1.04	-2.80	-5.37	317
**P11**	542	545	527	1	-0.75	1.37	-3.56	-5.68	413

[a]Absorption maxima. [b]Emission maxima in solution. [c]Polymer thin films on a glassy-carbon electrode in CH_3CN/0.1 M $[^nBu_4N]^+[PF_6]^-$, versus ferrocenium/ferrocene at 50 mV s^{-1}. E_{ox} is the onset potential corresponding to oxidations, whereas E_{red} is the onset potential corresponding to reductions. HOMOs and LUMOs are estimated from the onset oxidation and reduction potentials, respectively, assuming the absolute energy level of ferrocene/ferrocenium to be 4.8 eV below vacuum. [d]Temperature at 5% weight loss measured by TGA at a heating rate of 20 °C min^{-1} under N_2 atmosphere.

IV-3. Trap studies on P2-CS and P2-CI

IV-3-1. The trap density of states (tDOS) measurement

Trap density of states (tDOS) were performed on Keysight 4980A and analyzed by using the thermal admittance spectroscopy (TAS) method.[13] The configuration of the tested device was ITO/PEDOT:PSS/active layer/PDINN/Ag.

The thermal admittance spectroscopy (TAS) method was employed to check the trap density at varied energy depth. The angular frequency dependent capacitance can derive the energetic profile of trap density of states (tDOS) of solar cells according to the following equation:

$$N_T(E_\omega) = -\frac{V_{bi}}{qW}\frac{dC}{d\omega}\frac{\omega}{k_B T}$$

where k_B is the Boltzmann's constant, q is the elementary charge, ω is the angular frequency, C is the capacitance, T is the absolute temperature (using the room temperature), W is the depletion width (using the thickness of the active layer), and V_{bi}

is the built-in potential (extracted from the Mott-Schottky analysis). The applied angular frequency ω defined an energetic demarcation by the following formula:

$$E_{\omega} = k_B T \ln\left(\frac{\omega_0}{\omega}\right)$$

where ω_0 is the attempt-to-escape frequency. The trap states below the energy demarcation can capture or emit charges with the given ω and contribute to the capacitance.

ITO substrates were cleaned in deionized water, acetone, and isopropyl alcohol by sonication subsequently, and dried them finally. The PEDOT:PSS (Clevios P VP AI 4083) layer was spin-coated onto precleaned ITO glass substrates at 4000 rpm for 30 s, and subsequently dried at 150°C for 15 min in air. The active layer was dissolved in chloroform at 12 mg mL^{-1}, then deposited onto PEDOT:PSS layer through spin-coating at 3000 rpm for 30 s in nitrogen glove box. The PDINN was dissolved in methanol at 1 mg mL^{-1}, then spin-coated on the active layer at 4000 rpm for 30 s. Ag electrode (ca. 120 nm) was slowly deposited onto the surface of the PDINN layer by the thermal evaporation method under vacuum (*ca.* 10^{-5} Pa). The thickness of the film (ca. 50 nm) was measured *via* the Bruker Dektak-XT.

IV-3-2. OFETs fabrication

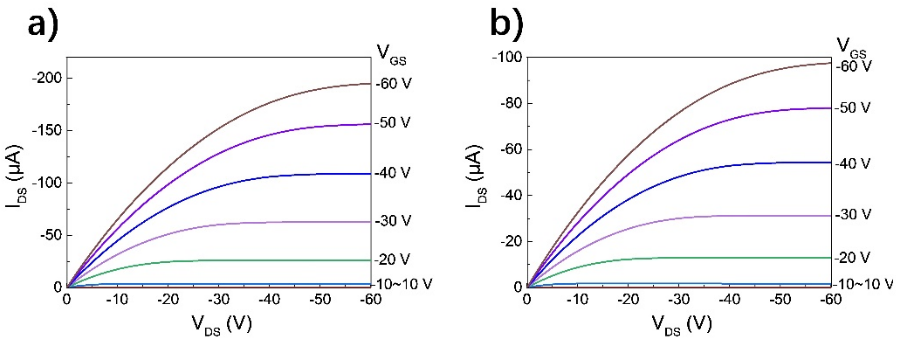

Supplementary Fig. 9. Output characteristics for (a) **P2-CS** and (b) **P2-CI** of OFETs devices.

Supplementary Table 5. Electrical Properties of **P2-CS** and **P2-CI** in OFETs.

Material	μ_h ($cm^2V^{-1}s^{-1}$) [a] av (max)	V_T (V) av (min)	Log (I_{on}/I_{off}) av (max)
**P2-CS**	0.112 (0.115)	3.1 (0.1)	5.8 (6.1)
**P2-CI**	0.057 (0.060)	-3.4 (-1.3)	5.5 (5.7)

[a]The average characteristics were obtained from more than 5 devices for each material.

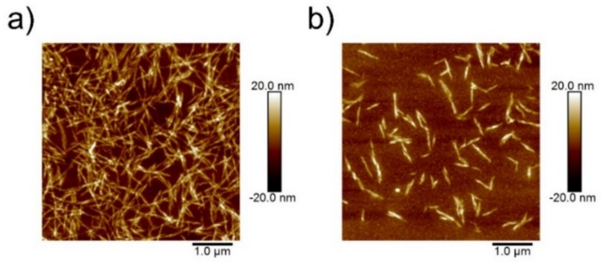

Supplementary Fig. 10. AFM height images of polymer (a) **P2-CS** and (b) **P2-CI** thin films.

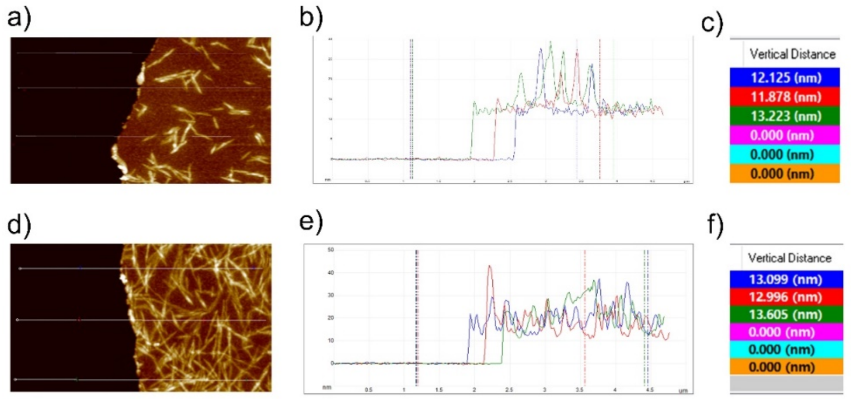

Supplementary Fig. 11. Part of AFM height images and vertical distance demonstration for **P2-CS** (a, b and c) and **P2-CI** (d, e and f) thin films.

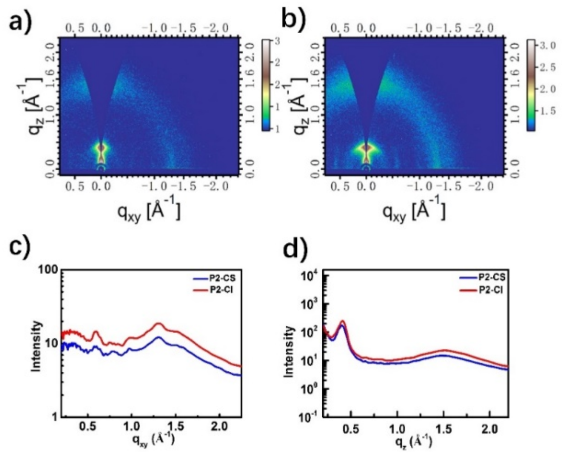

Supplementary Fig. 12. 2D GIWAXS patterns of **P2-CS** (a), **P2-CI** (b) showing in-plane and out-of-plane profiles of 2D-GIWAXS patterns of the polymer films for **P2-**

CS (c) and **P2-CI** (d) .

IV-4. Applications of high quality materials based on P2-CS, P4, and P5.

IV-4-1. Cell imaging applications

1. Methods

1.1 The preration of P2-CS NPs

The polymer **P2-CS** (5 mg, M_n = 13.4 kDa) and PF127 (50 mg) were dissolved in THF (1 mL). The above THF solution was sonicated with an ultrasonic cell disruptor (160 W) for 5 min. While sonicating, deionized (DI) water (5 mL) was added dropwise to the THF solution and sonicated for 10 min. The obtained NPs mixed solution was stirred at room temperature for 48 h to completely remove THF. The NPs solution was dialyzed in DI water with a dialysis tube with a molecular weight cut-off of 3.5 kDa for 2 days to remove excess emulsifier. The obtained NP aqueous suspension was stored in the refrigerator for future use.

1.2 Cell culture

HeLa cells were selected for cell imaging. HeLa cells were provided by the Functional Materials Laboratory of the School of Materials Science and Opto-electronic Technology, University of Chinese Academy of Sciences. HeLa cells were cultured in 90% PRMI-1640 medium, 10% FBS and 1% penicillin and streptomycin complete medium in a 5% CO_2 cell incubator at 37 °C.

1.3 Cell viability

Hela cells were cultured in complete PRMI-1640 medium for 24 h, then were seeded in 96-well cell culture plates at a density of 10,000 cells per well (n = 6). After 24 h, Hela cells were co-cultured with different concentrations of **P2-CS** NPs (5 μg mL^{-1}, 10 μg mL^{-1}, 25 μg mL^{-1}, and 50 μg mL^{-1}) diluted with complete PRMI-1640 medium. After 24 hours of incubation, the cells were washed three times with PBS to remove excess NPs. The CCK-8 assay was used to determine the cytotoxicity of **P2-CS** NPs to

Hela.

1.4 Cell imaging

HeLa cells were seeded in a 35 mm diameter confocal culture dish at a density of 100,000 cells per well. After cultured in Roswell Park Memorial Institute (RPMI) 1640 for 24 h, HeLa cells were co-incubated with P2 NPs (10 μg mL-1). After 24 h, the HeLa cells were washed with phosphate buffer saline (PBS) three times. Afterwards, the HeLa cells were stained with LysoTracker green (10 nM) for 1 h at 37 °C. After the HeLa cells were washed with PBS three times, they were imaged with a confocal laser scanning microscope (LSM880) for cell imaging.

2. Results and discussion

Since **P2-CS** exhibits the strongest fluorescence emission of the current polymers (Supplementary Fig. 13a), it was next selected as a potential dye for cell imaging. Through nanoprecipitation,[14] the **P2-CS** nanoparticles (NPs) were prepared with Pluronic® F127 (PF127) as a neutral emulsifier. The size, zeta potential, and morphology of the **P2-CS** NPs were characterized by dynamic light scattering (DLS) and transmission electron microscopy (TEM). The DLS results show that the average particle size and polydispersity index (PDI) of the **P2-CS** NPs to be ~167 nm and 0.114, respectively, implying significant uniformity (Supplementary Fig. 13b), consistent with the TEM images (Supplementary Fig. 14). The negative zeta potentials of **P2-CS** NPs (-17.3 mV) suggest that the NPs are stable in aqueous solution (Supplementary Fig. 15). Moreover, a Cell Counting Kit-8 (CCK8) assay was used to assess the cytotoxicity of the **P2-CS** NPs after incubation with HeLa cells for 24 h. at NP concentrations as high as 10 μg mL^{-1}. It is found that the survival rate of HeLa cells is near 96%, indicating low toxicity (Supplementary Fig. 13c). Cell imaging experiments were also performed with HeLa cells. Supplementary Fig. 13c-III shows that **P2-CS** NPs can be endocytosed

by cells and emit strong red fluorescence (Supplementary Fig. 16). In lysosome co-localization experiments, Supplementary Fig. 13c-II shows that lysosomes can be stained to emit green fluorescence. After the two graphs overlap (Supplementary Fig. 13-IV), the co-localization coefficient between **P2-CS** NPs and lysosome is 0.79 (Supplementary Fig. 17). This high value[15,16] for the **P2-CS** NPs suggests promise in cell imaging for the BBT-based polymers.

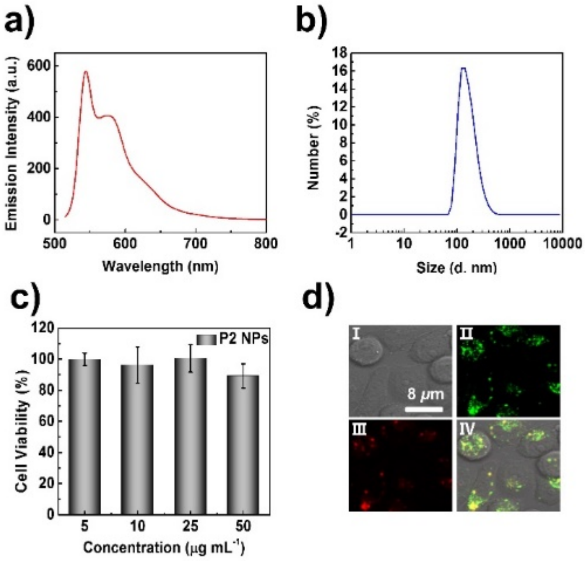

Supplementary Fig. 13. (a) Emission spectra of **P2-CS** in chloroform solution. (b) Size distribution analysis of **P2-CS** NPs by DLS. (c) Cell viability of HeLa cells incubated with different concentration of **P2-CS** NPs. (d) Confocal laser scanning microscope images of **I**) the bright-field image; **II**) MitoTracker Green (Ex, 488 nm, Em, 493-622 nm); **III**) **P2-CS** (Ex, 561 nm, Em, 566-685 nm); and **IV**) merged image of bright-field, MitoTracker Green, and **P2-CS**.

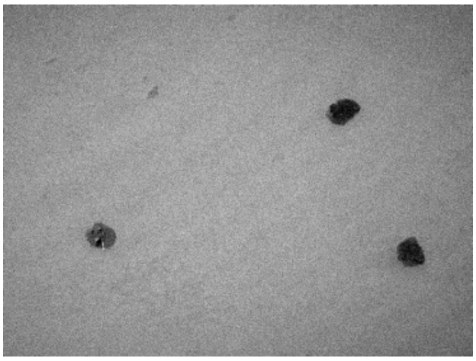

Supplementary Fig. 14. TEM images of **P2-CS** NPs.

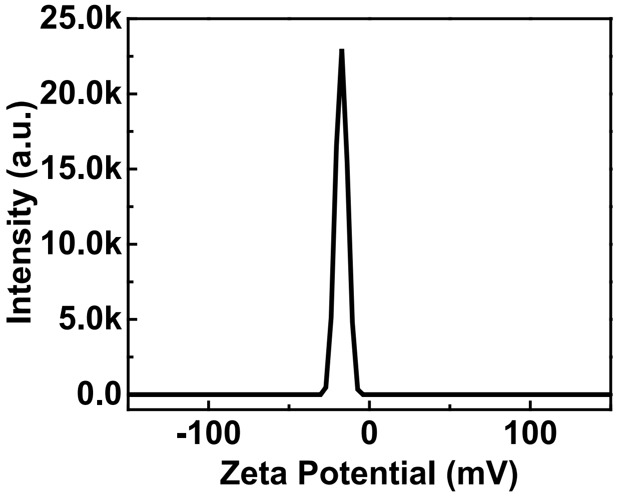

Supplementary Fig. 15. Zeta potential analysis of **P2-CS** NPs.

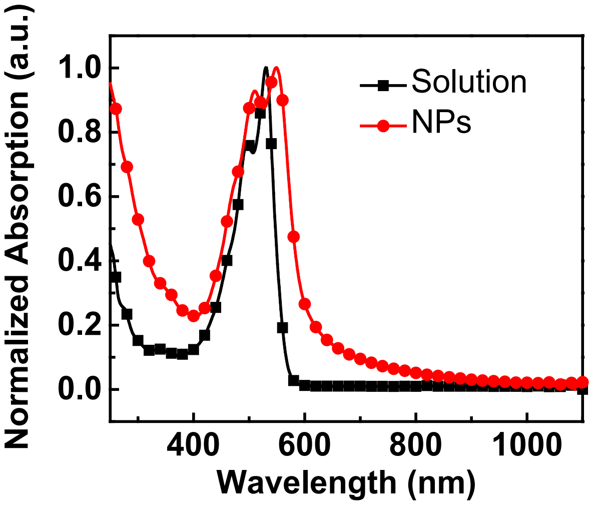

Supplementary Fig. 16. The UV-vis absorption spectra of **P2-CS** in dichloromethane solution and as NPs.

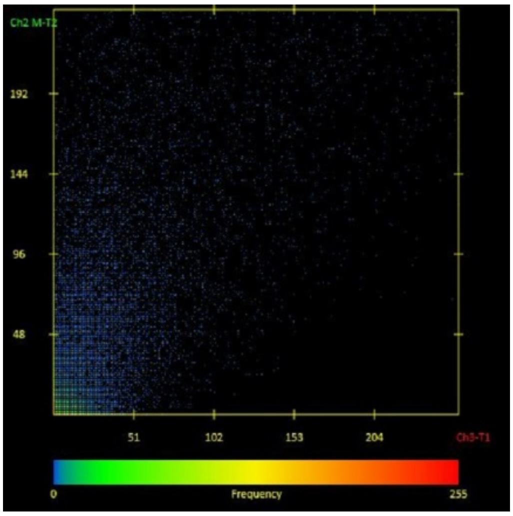

Supplementary Fig. 17. Colocalization scatter plot showing a Pearson's correlation

(PC) coefficient of 0.79.

IV-4-2. OFETs fabrication for P4 and P5

1. Method

The thin polymer films were deposited by spin-coating of a 20 mg mL^{-1} polymer solution in 1,2-dichlorobenzene in a nitrogen-filled glovebox onto octadecyltrichlorosilane(OTS)-treated p-doped Si (001) wafers with a 300 nm thermally grown SiO_2 dielectric layer. The capacitance of the 300 nm SiO_2 gate insulator was 10 $nF.cm^{-2}$. Prior to substrate treatment with OTS, the wafers were first cleaned by piranha solution and then cleaned by sonicating (in two beakers, sequentially, for 15 min each) in EtOH and were then dried with a filtered stream of N_2, followed by 20 min UVO zone cleaning. After semiconductor deposition, the films were annealed at different temperatures under vacuum for 30 min. Top contact OFETs were fabricated by vapor deposition of gold electrodes (~10^{-5} Torr, 0.1 Å s^{-1}, ~40 nm thick) onto the semiconductor thin films through a shadow mask to obtain devices with channel widths and lengths of 1400 μm and 40 μm, respectively.

IV plots of device performance were measured in ambient or under vacuum; at least five transfer and output plots were recorded for each device. The current-voltage (*I*-*V*) characteristics of the devices were measured using Keysight B1500A source meter, operated by a local Labview program and GPIB communication. Key device parameters, such as charge carrier mobility (μ) and on-to-off current ratio ($I_{on/off}$) were extracted from the source-drain current (I_{SD}) versus gate voltage (V_G) characteristics employing standard procedures. Mobilities were obtained from the formula defined by the saturation regime in transfer plots, $\mu = 2I_{SD}L/[C_iW(V_G\text{-}V_T)^2]$, where I_{SD} is the source-drain current, V_G is gate voltage, and V_T is the threshold voltage. Threshold voltage was obtained from the x intercept of V_G vs $I_{SD}^{1/2}$ plots.

2. Results and discussion

Since polymer **P4** synthesized by classic Stille coupling has excellent charge transport (mobility = 0.12 to 0.26 cm^2/Vs),[17] organic thin film transistors (OFETs) were fabricated with polymers **P4** and **P5** for comparison. As shown in Supplementary Fig. 18. a and b, the **P4** based OFETs exhibit a good hole mobility of 0.12 cm^2/Vs with a high $I_{on/off}$ (on-to-off current ratio) of *ca.* 10^7, comparable to the literature data.[14] Moreover, **P5** OFETs have a higher hole mobility of 0.23 cm^2/Vs with a $I_{on/off}$ of *ca.* 10^6, which may reflect the higher molecular weight, more ordered crystalline (Supplementary Fig. 19) and fibrous morphology (Supplementary Fig. 18. c and d). Again, the results argue that C-S activation based cross-coupling polymerization at room temperature can produce conjugated polymers with comparable or superior microstructural and electronic properties to those from classic Stille coupling at high temperature.

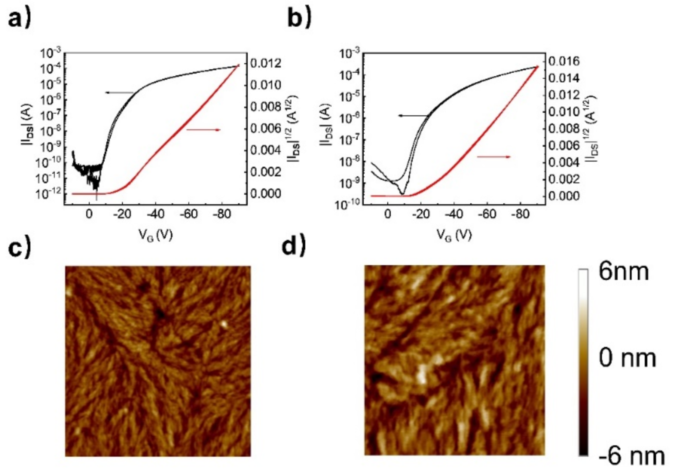

Supplementary Fig. 18. OFETs transfer curves of polymer (**a**) **P4** and (**b**) **P5** thin films. AFM height images of polymer (**c**) **P4** and (**d**) **P5** thin films.

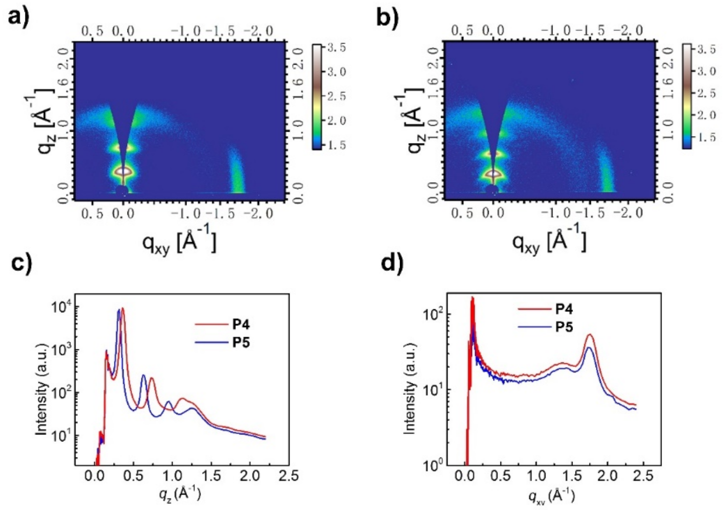

Supplementary Fig. 19. 2D GIWAXS patterns of **P4** (a)**, P5** (b) showing out-of-plane and in-plane profiles of 2D-GIWAXS patterns of the polymer films for **P4** (c) and **P5** (d) .

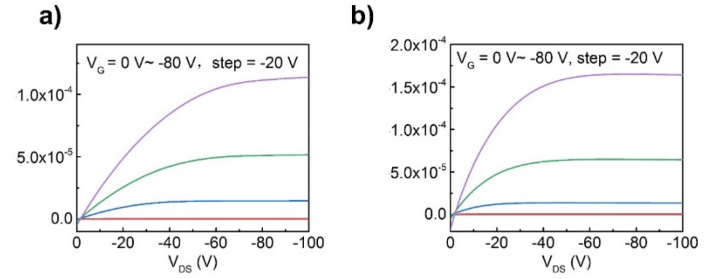

Supplementary Fig. 20. Output characteristics for (a) **P4** and (b) **P5** of OFETs devices.

V. References

1. Mike, J. F., Inteman, J. J., Ellern, A. & Jeffries-El, M., Facile Synthesis of 2,6-Disubstituted Benzobisthiazoles: Functional Monomers for the Design of Organic Semiconductors. *J. Org. Chem.* **75**，495-497 (2010).
2. Kumar, N. S., Braun, M. P., Chaudhary, A. G. & Young, R. N. Synthesis of a tritium-labeled photo-affinity probe based on an atypical leukotriene biosynthesis inhibitor. *J. Label Compd. Radiopharm* **54,** 43-50 (2011).
3. Zhu, Z., Glinkerman, C. M. & Boger, D. L. Selective N1/N4 1,4-Cycloaddition of 1,2,4,5-Tetrazines Enabled by Solvent Hydrogen Bonding. *J. Am. Chem. Soc.* **142,** 20778-20787 (2020).

4. Shi, Q., et al. KO*t*Bu-Initiated Aryl C–H Iodination: A Powerful Tool for the Synthesis of High Electron Affinity Compounds. *J. Am. Chem. Soc.* **138,** 3946−3949 (2016).

5. Wang, X., et al. Effects of π-Conjugated Bridges on Photovoltaic Properties of Donor-π-Acceptor Conjugated Copolymers. *Macromolecules,* **46,** 2521-2521 (2013).

6. Tsuji, M. et al. Benzobisthiazole as Weak Donor for Improved Photovoltaic Performance: Microwave Conductivity Technique Assisted Molecular Engineering. *Adv. Funct. Mater.*, **24,** 28-36 (2014).

7. Nakanishi, T., Shirai, Y. & Han, L. Synthesis and optical properties of photovoltaic materials based on the ambipolar dithienonaphthothiadiazole unit. *J. Mater. Chem. A*, **3,** 4229-4238 (2015).

8. Efrem, A., Lim, C. J., Lu, Y. & Ng, S. C. Synthesis and characterization of dithienobenzothiadiazole-based donor–acceptor conjugated polymers for organic solar cell applications. *Tetrahedron Lett.* **55,** 4849–4852 (2014).

9. Matsushita K., Takise R., Hisada T., Suzuki S., Isshiki R., Itami K., Muto K., Yamaguchi J. Pd-Catalyzed Decarbonylative C-H Coupling of Azoles and Aromatic Esters. *Chem. Asian J.* **13,** 2393-2396 (2018).

10. Arora, A. & Weaver, J. D. Photocatalytic Generation of 2-Azolyl Radicals: Intermediates for the Azolylation of Arenes and Heteroarenes via C–H Functionalization. *Org. Lett.* **18,** 3996−3999 (2016).

11. Saito, M., Osaka, I., Suda, Y., Yoshida, H. & Takimiya, K. Dithienylthienothiophenebisimide, a Versatile Electron-Deficient Unit for Semiconducting Polymers. *Adv. Mater.* **28,** 6921–6925 (2016)

12. Chen G.-Y., et al. Cationic Ir(III) Emitters with Near-Infrared Emission Beyond 800 nm and Their Use in Light-Emitting Electrochemical Cells. *Chem. Eur. J.* **25,** 5489 – 5497 (2019).

13. J. A. Carr, S. Chaudhary. The identification, characterization and mitigation of defect states in organic photovoltaic devices: a review and outlook. *Energy Environ. Sci.* ***6*****,** 3414 (2013)

14. Geng, J.*, et al.* Biocompatible Conjugated Polymer Nanoparticles for Efficient Photothermal Tumor Therapy. *Small* **11**, 1603-1610 (2015).

15. Chen, Z., Yuan, H., Liang, H., Lu, C. & Liu, X. Synthesis of a cationic poly(p-phenylenevinylene) derivative for lysosome-specific and long-term imaging. *Chinese*

Chemical Letters **29** (2018).

16. Han, T., *et al.* Facile Multicomponent Polymerizations toward Unconventional Luminescent Polymers with Readily Openable Small Heterocycles. *Journal of the American Chemical Society* **140**, 5588-5598 (2018).
17. Osaka, I., Takimiya, K. & McCullough, R.D. Benzobisthiazole-based semiconducting copolymers showing excellent environmental stability in high-humidity air. *Advanced Materials* **22**, 4993-4997 (2010)

VI. NMR spectra and GPC traces

Monomers

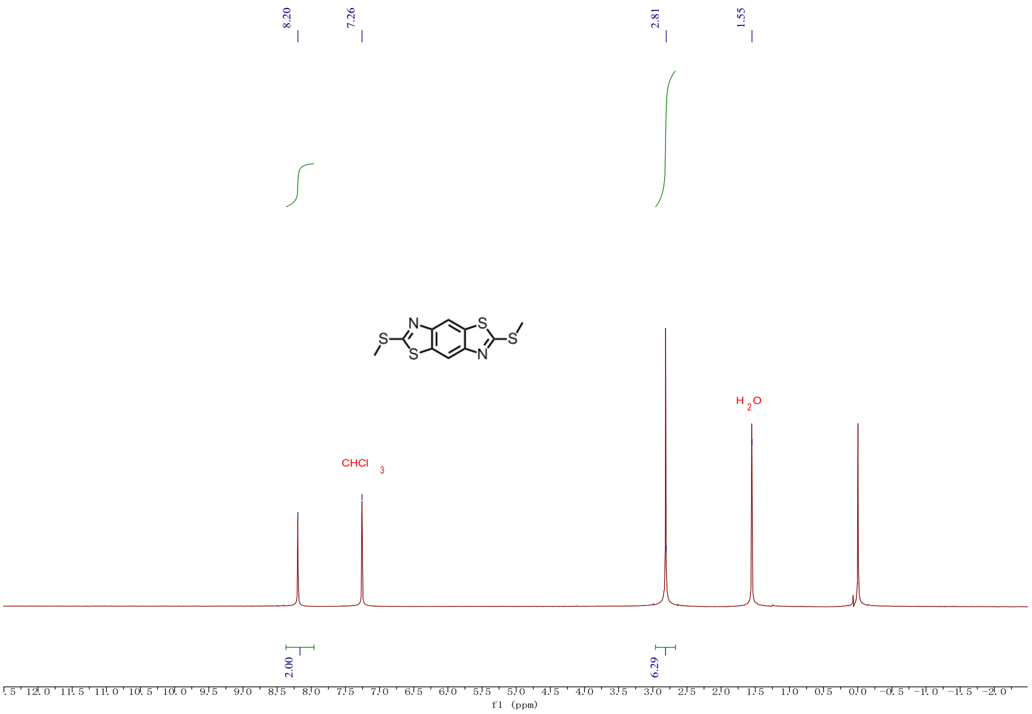

Supplementary Fig. 21. ^{1}H NMR spectrum of compound **E1** in $CDCl_3$ at 298 K.

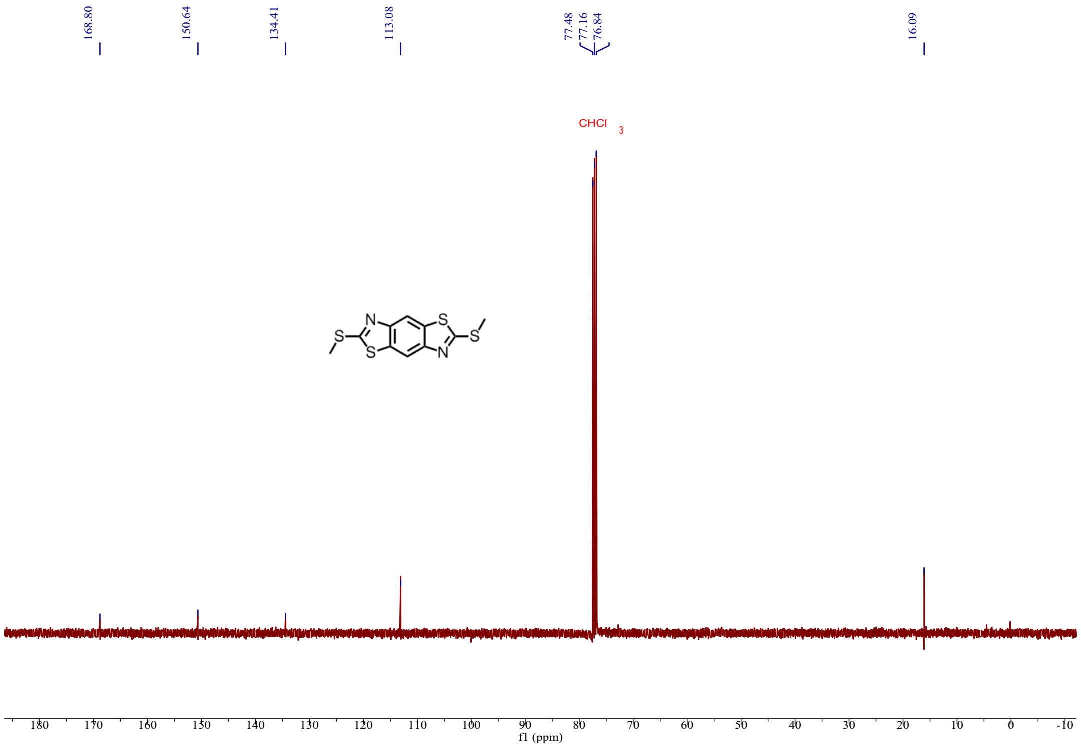

Supplementary Fig. 22. ^{13}C NMR spectrum of compound **E1** in $CDCl_3$ at 298 K.

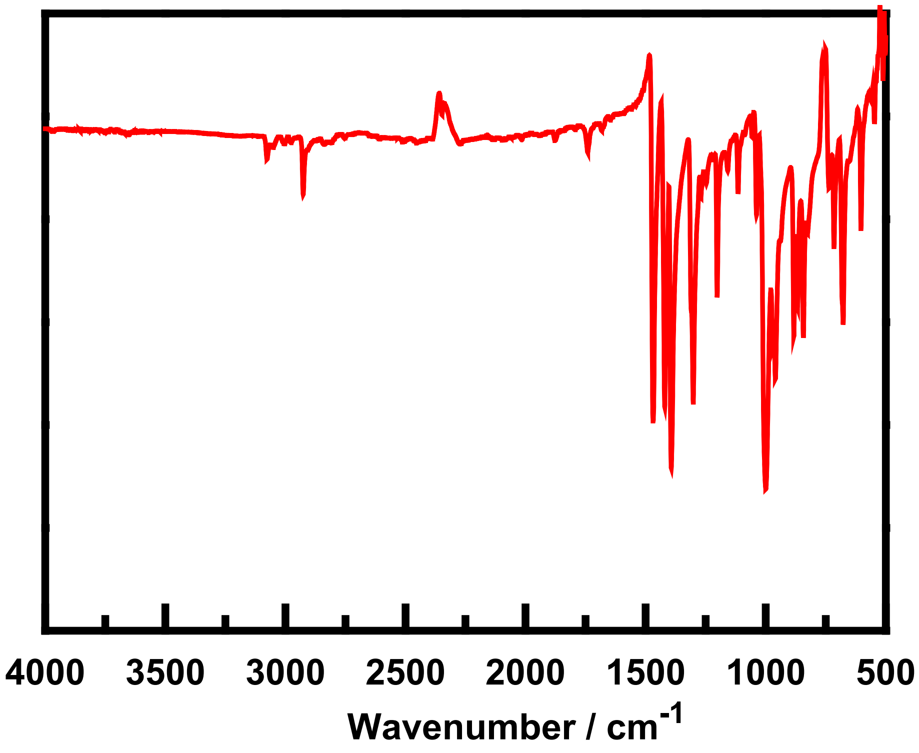

Supplementary Fig. 23. FT-IR spectrum of compound **E1** in thin film at 298 K.

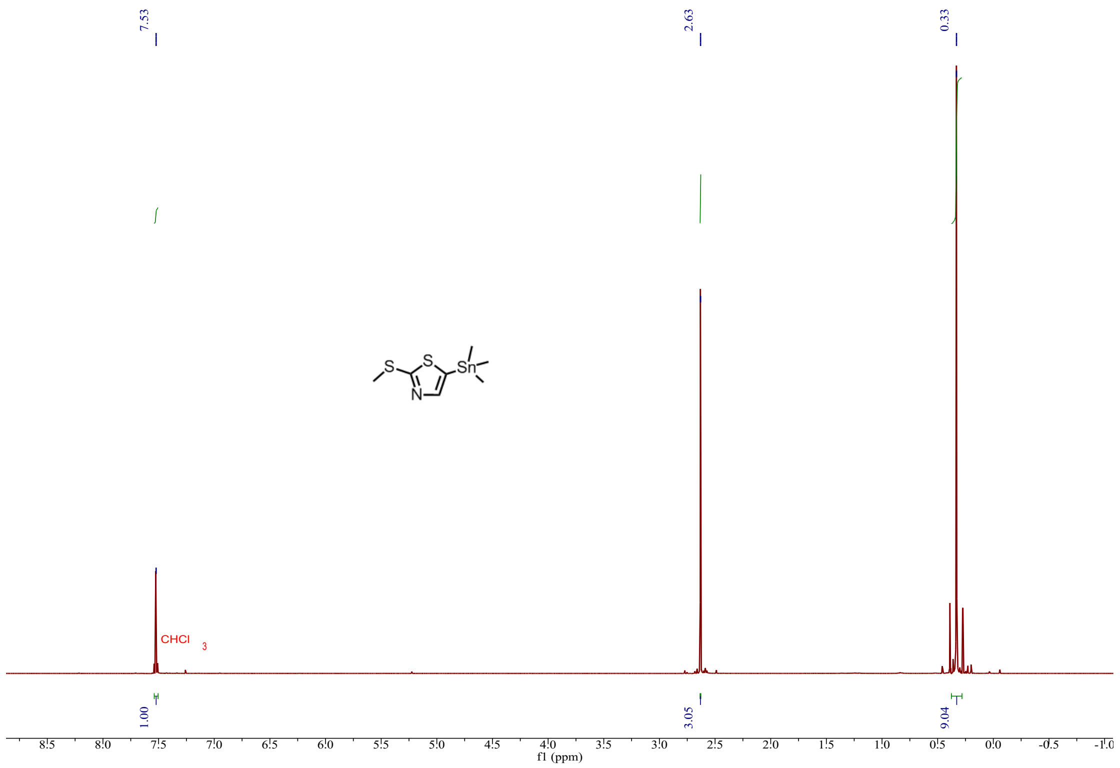

Supplementary Fig. 24. ^{1}H NMR spectrum of compound **2** in $CDCl_3$ at 298 K.

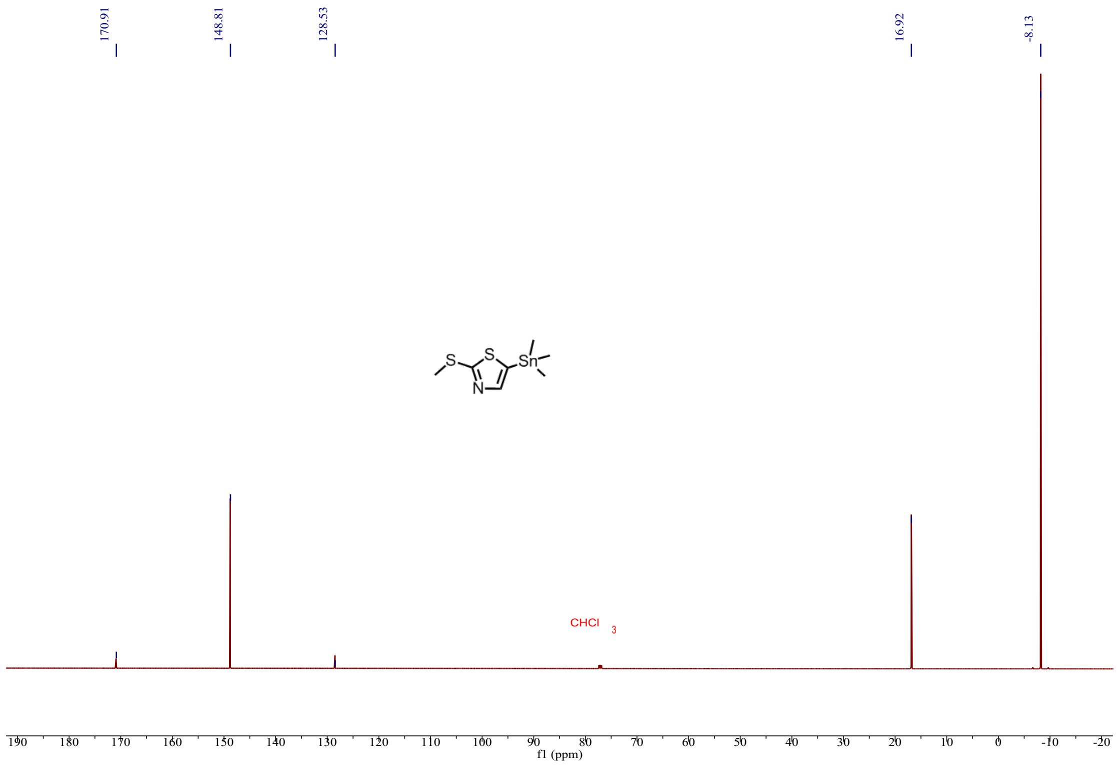

Supplementary Fig. 25. $^{13}C\{^{1}H\}$ NMR spectrum of compound **2** in $CDCl_3$ at 298 K.

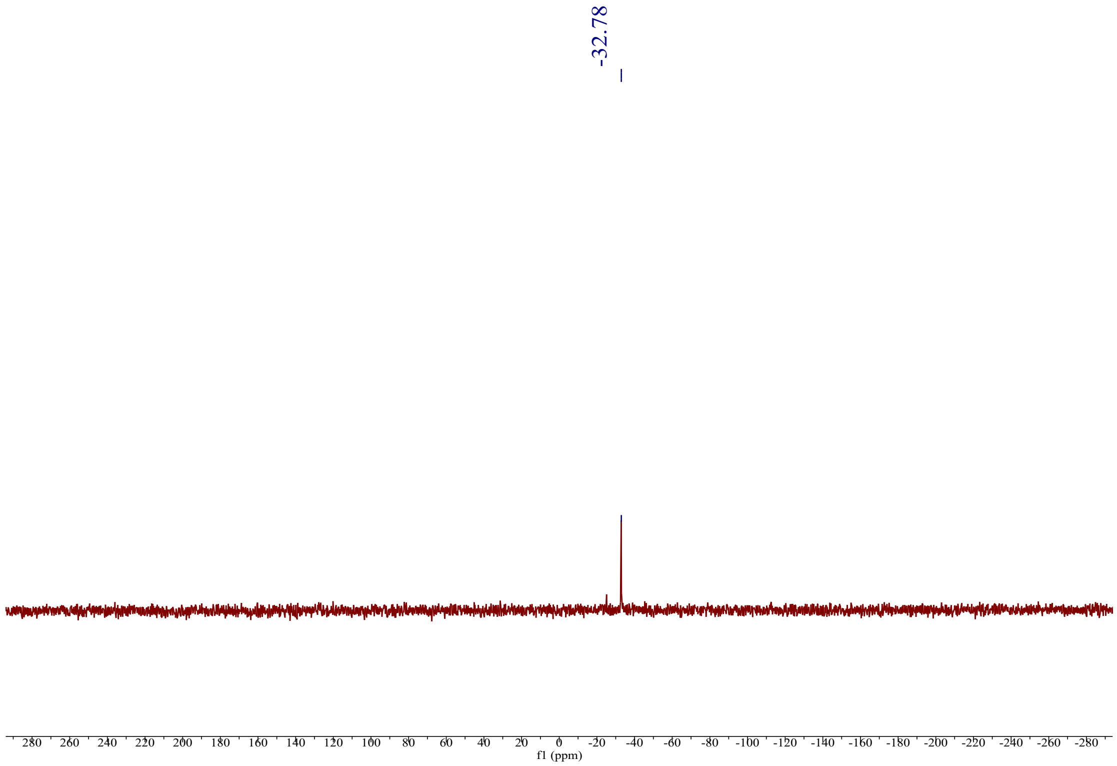

Supplementary Fig. 26. ^{119}Sn NMR spectrum of compound **2** in $CDCl_3$ at 298 K.

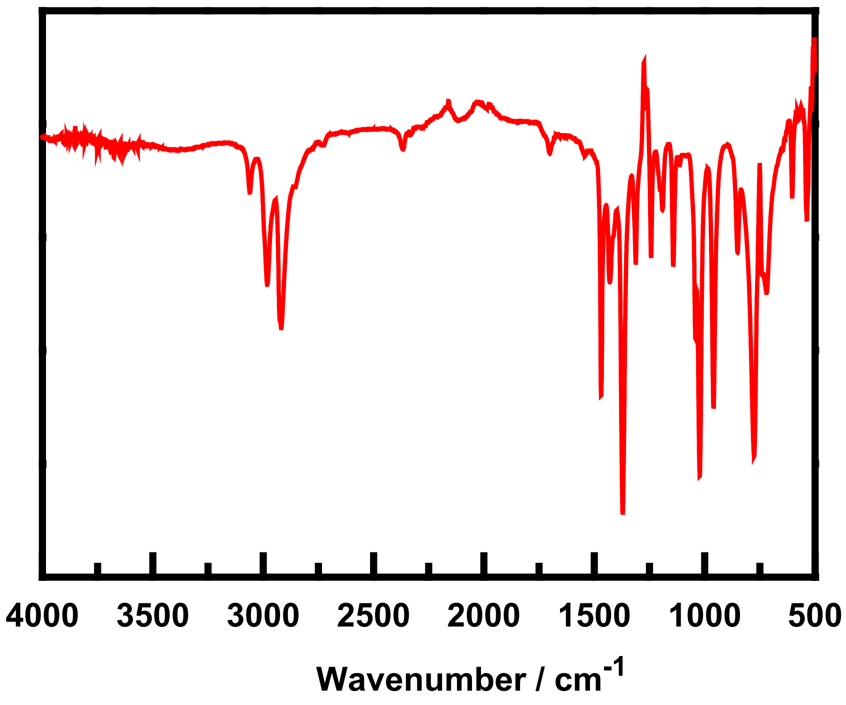

Supplementary Fig. 27. FT-IR spectrum of compound **2** in thin film at 298 K.

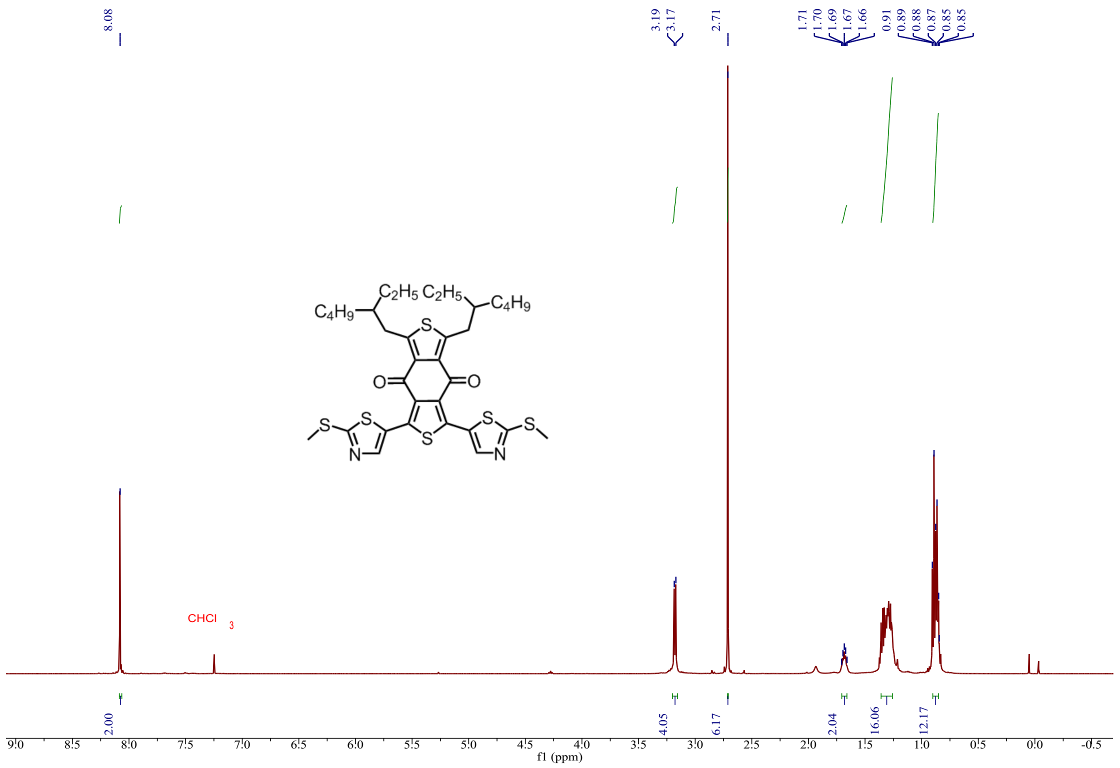

Supplementary Fig. 28. ^{1}H NMR spectrum of compound **E2** in $CDCl_3$ at 298 K.

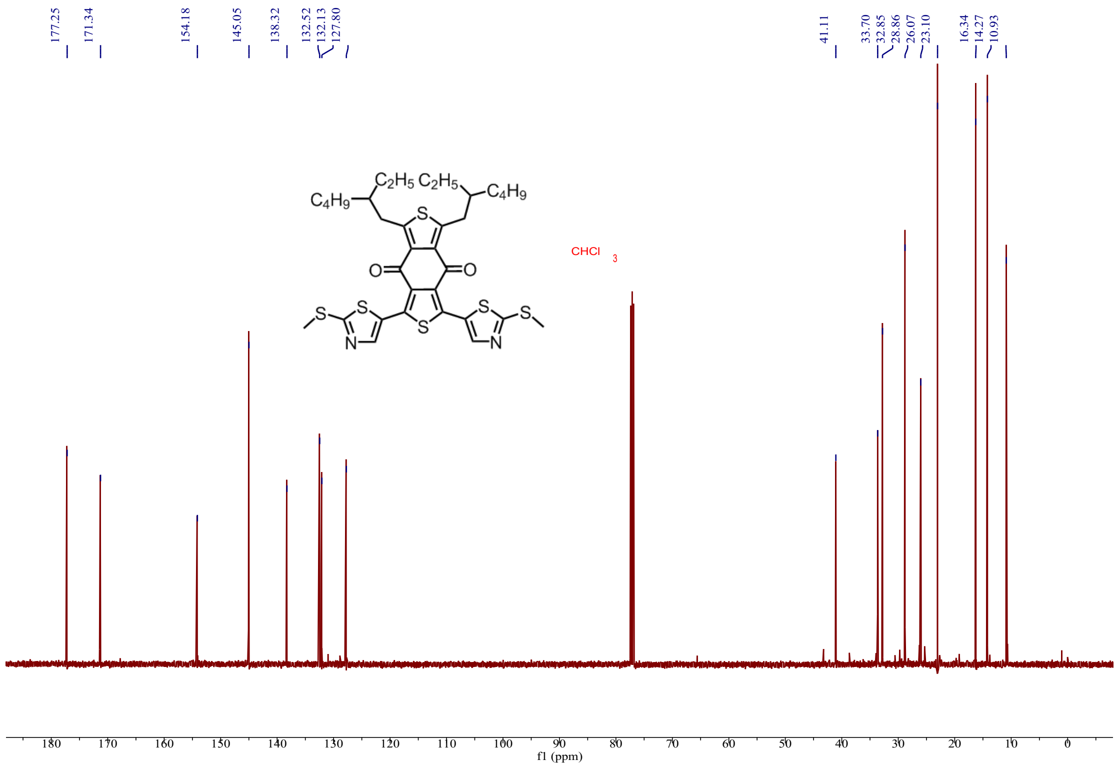

Supplementary Fig. 29. $^{13}C\{^{1}H\}$ NMR spectrum of compound **E2** in $CDCl_3$ at 298 K.

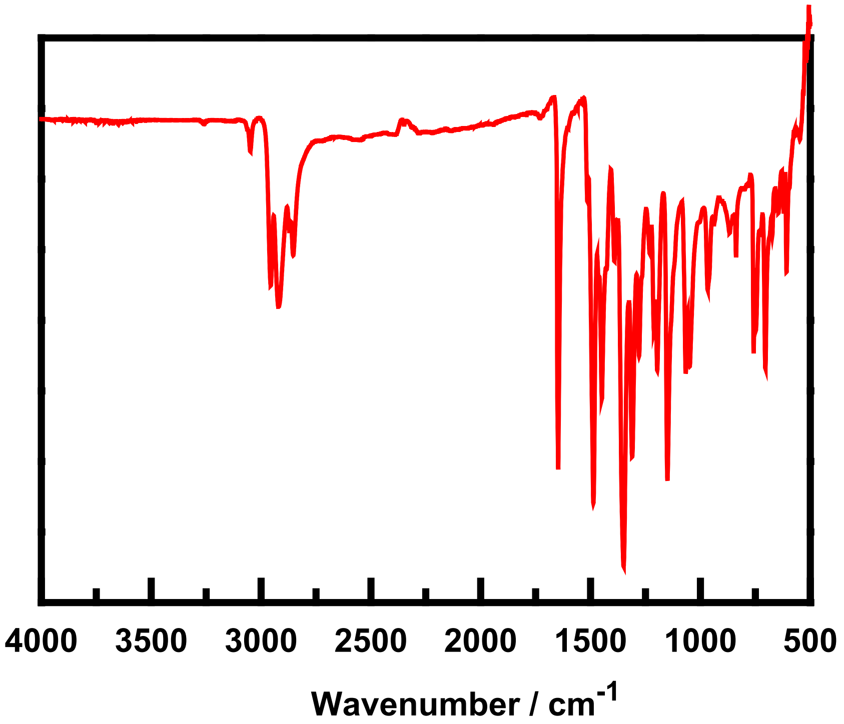

Supplementary Fig. 30. FT-IR spectrum of compound **E2** in thin film at 298 K.

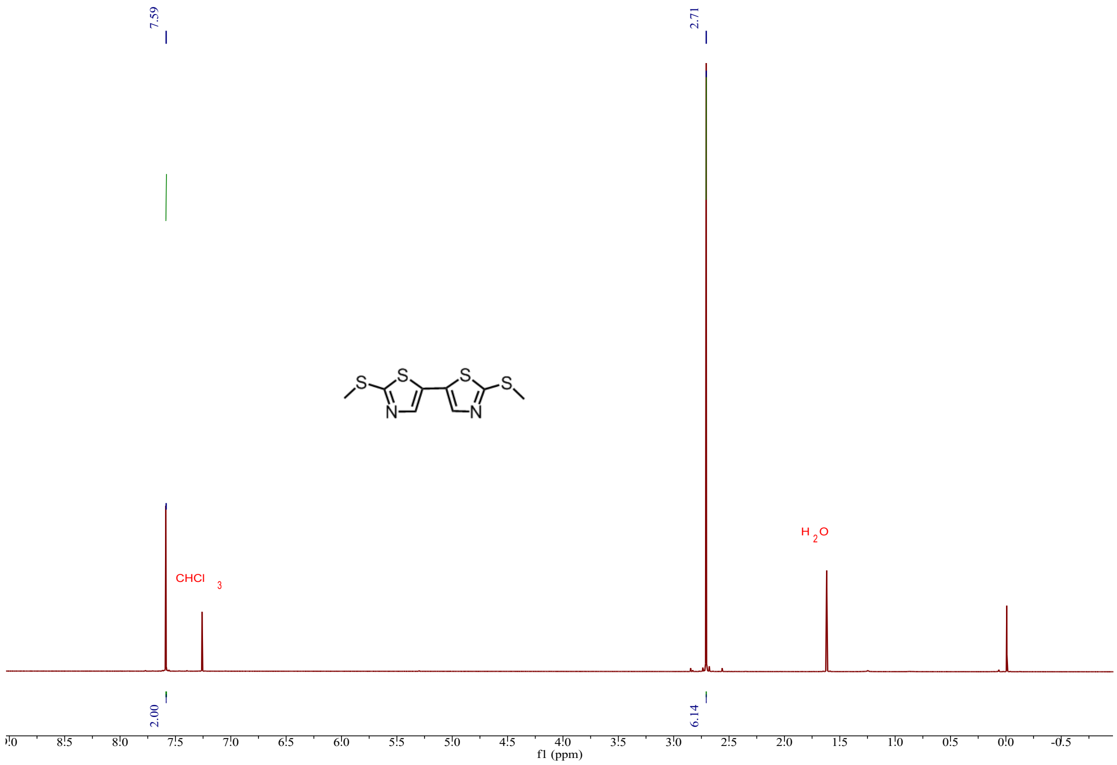

Supplementary Fig. 31. ^{1}H NMR spectrum of compound **E3** in $CDCl_3$ at 298 K.

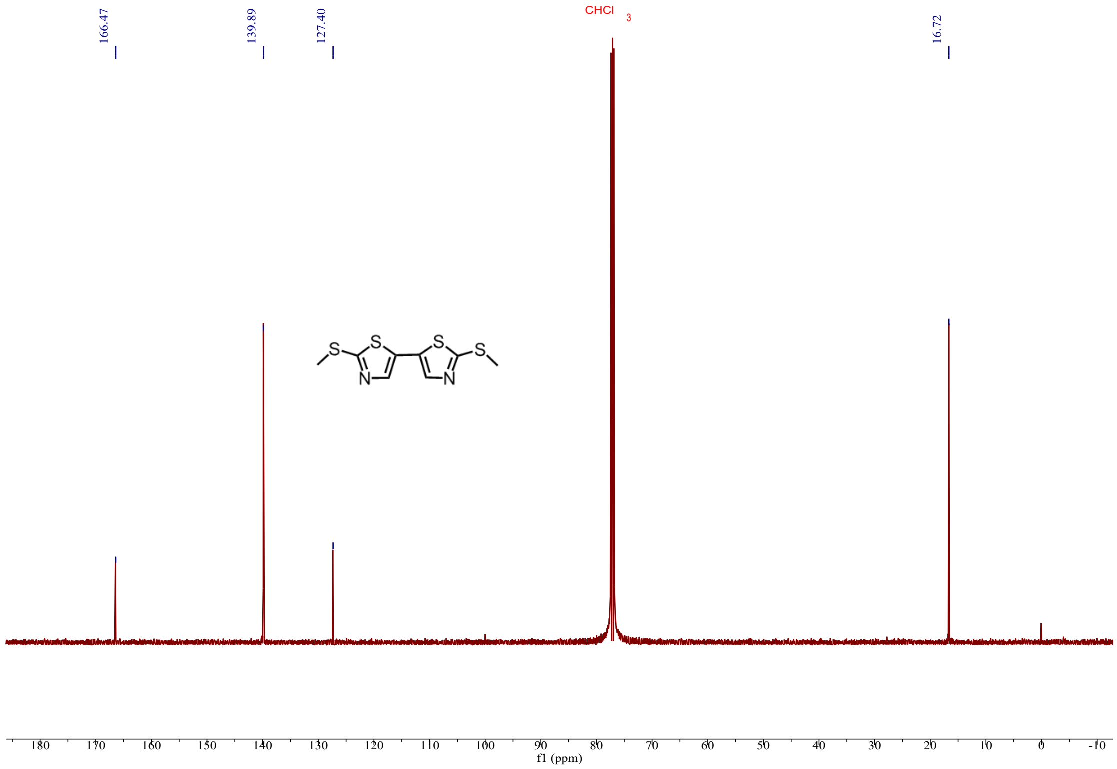

Supplementary Fig. 32. $^{13}C\{^{1}H\}$ NMR spectrum of compound **E3** in $CDCl_3$ at 298 K.

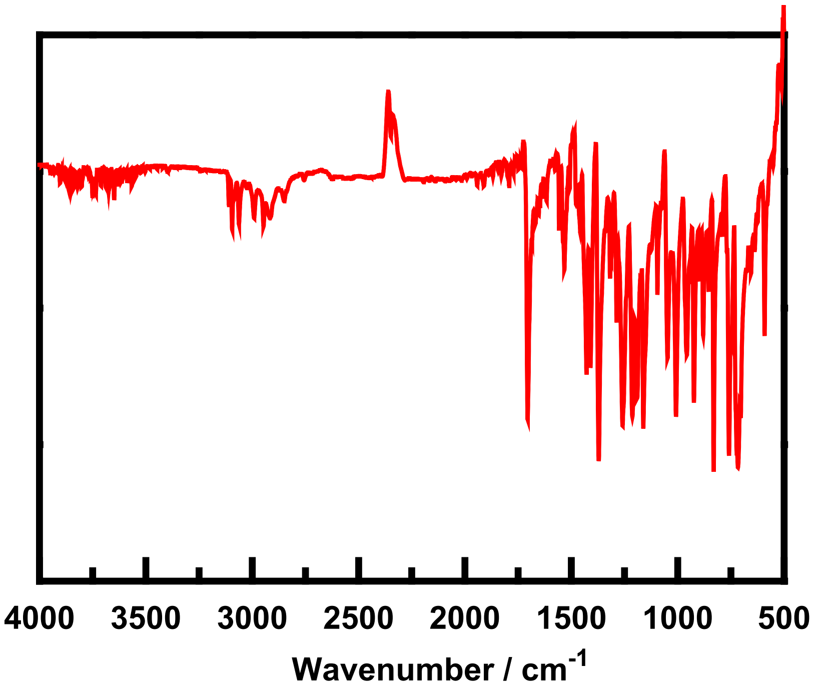

Supplementary Fig. 33. FT-IR spectrum of compound **E3** in thin film at 298 K.

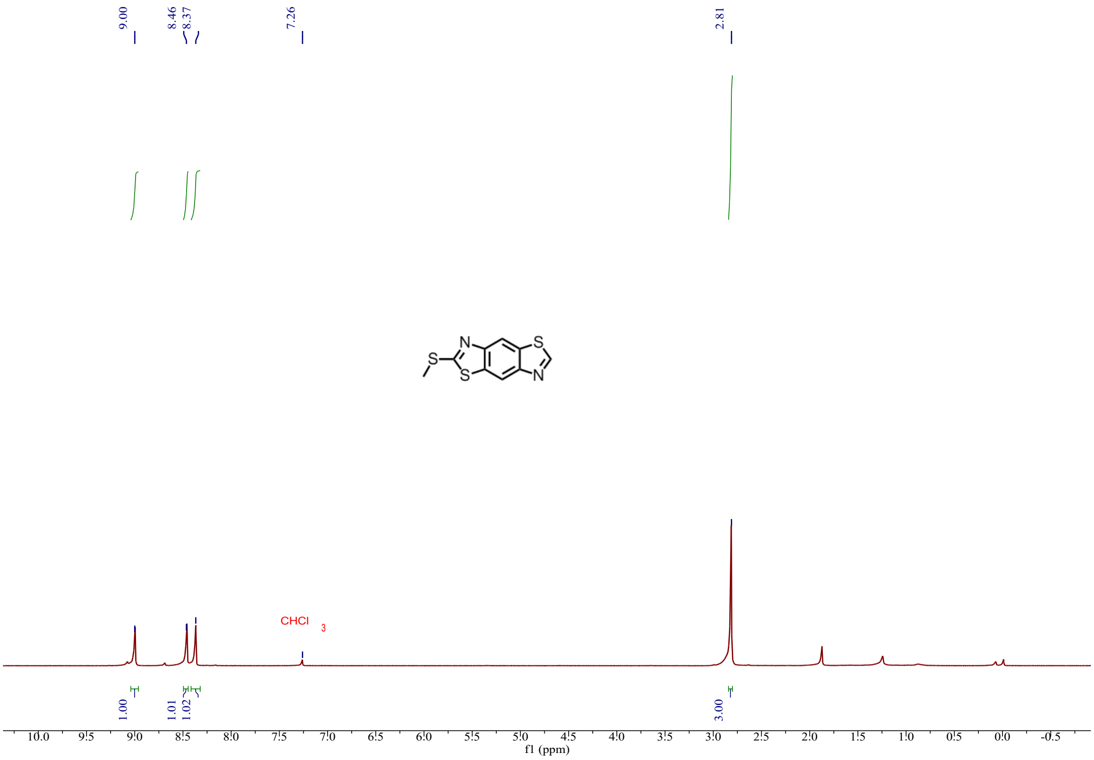

Supplementary Fig. 34. ^{1}H NMR spectrum of compound **E6** in $CDCl_3$ at 298 K.

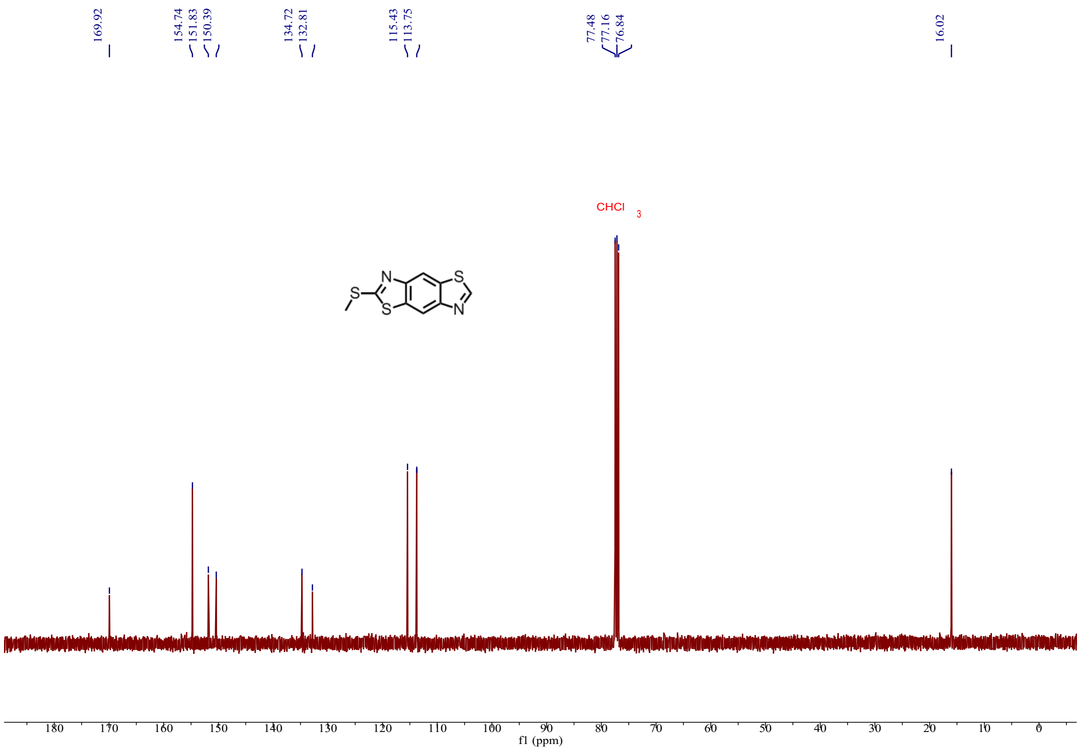

Supplementary Fig. 35. $^{13}C\{^1H\}$NMR spectrum of compound **E6** in $CDCl_3$ at 298 K.

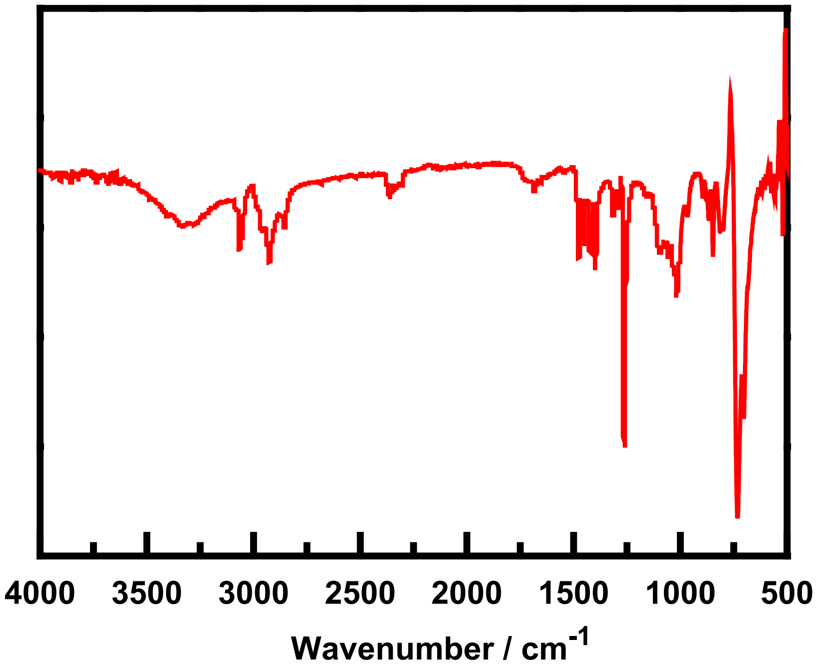

Supplementary Fig. 36. FT-IR spectrum of compound **E6** in thin film at 298 K.

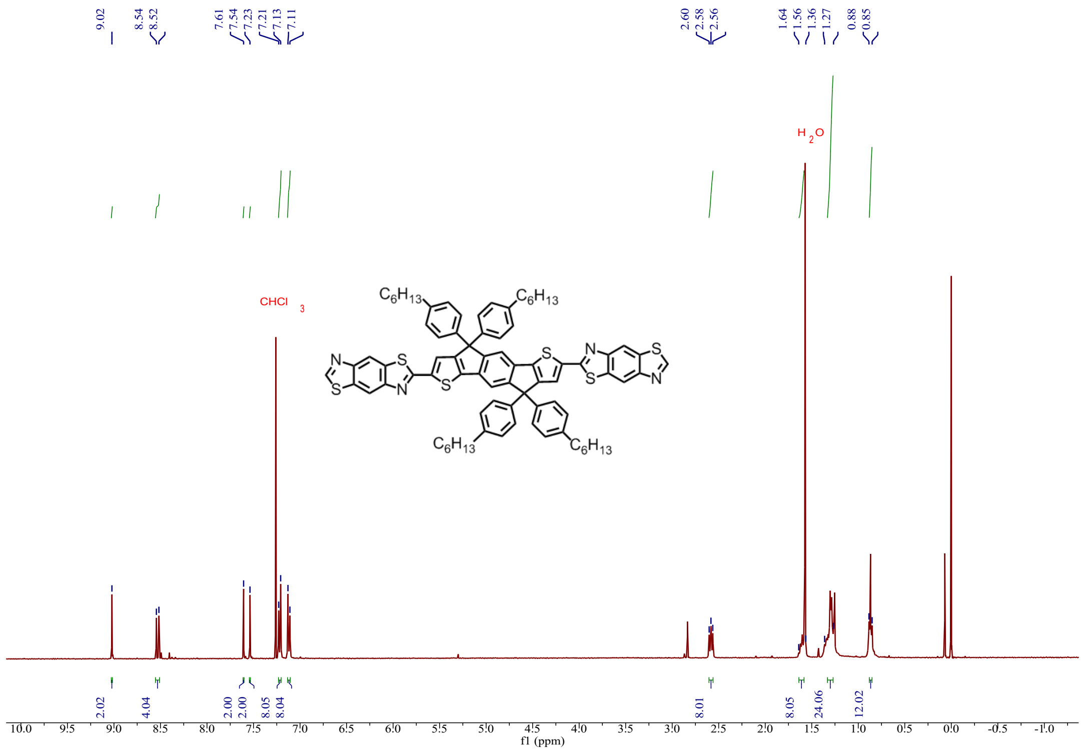

Supplementary Fig. 37. ^{1}H NMR spectrum of compound **5** in $CDCl_3$ at 298 K.

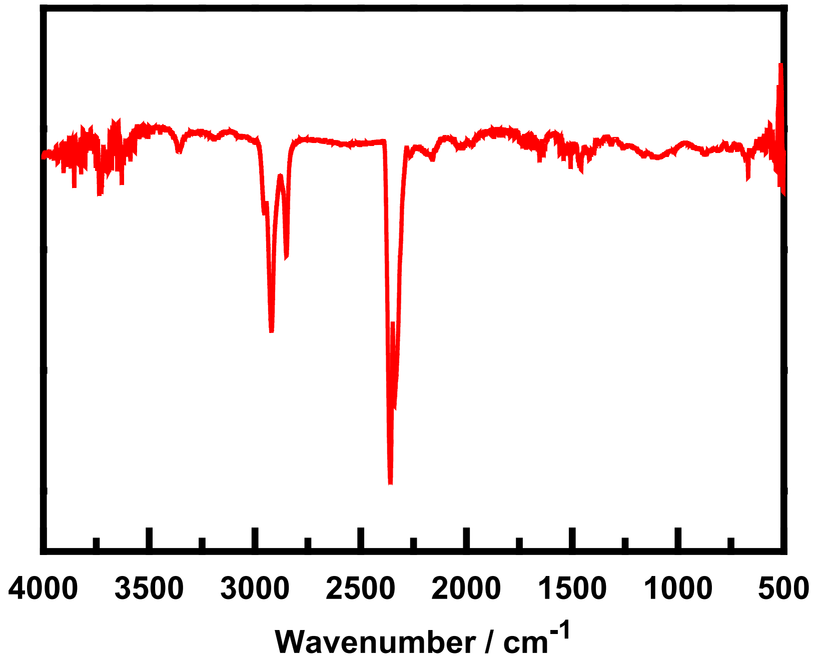

Supplementary Fig. 38. FT-IR spectrum of compound **5** in thin film at 298 K.

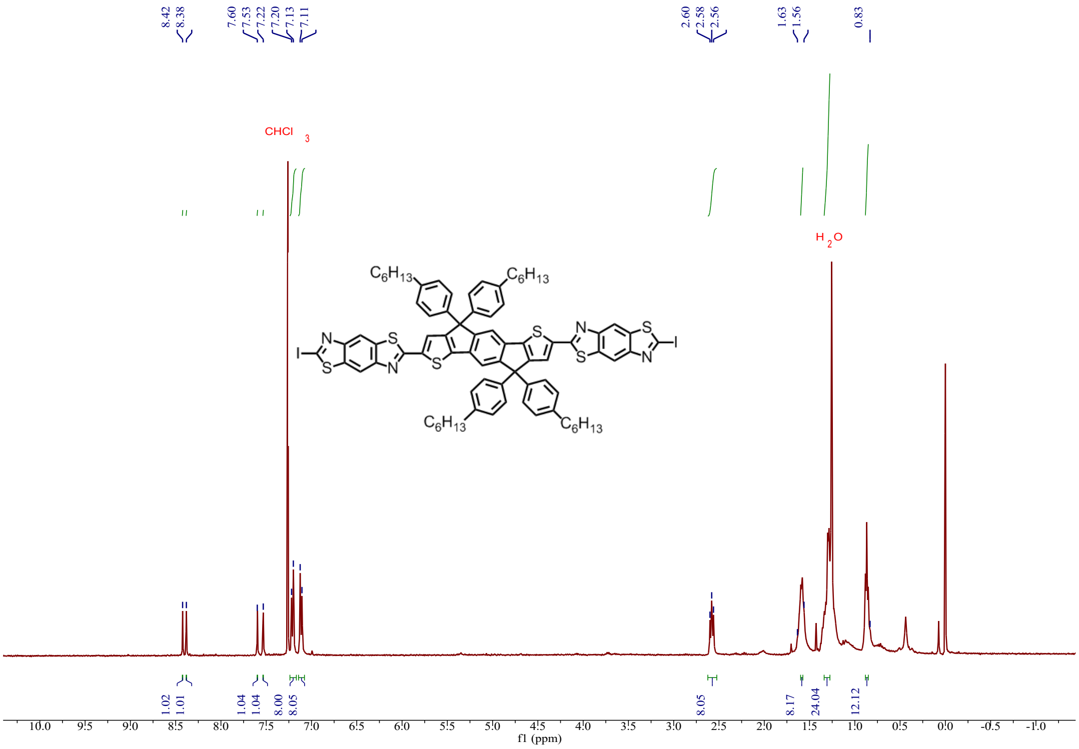

Supplementary Fig. 39. ^{1}H NMR spectrum of compound **E7** in CDCl3 at 298 K.

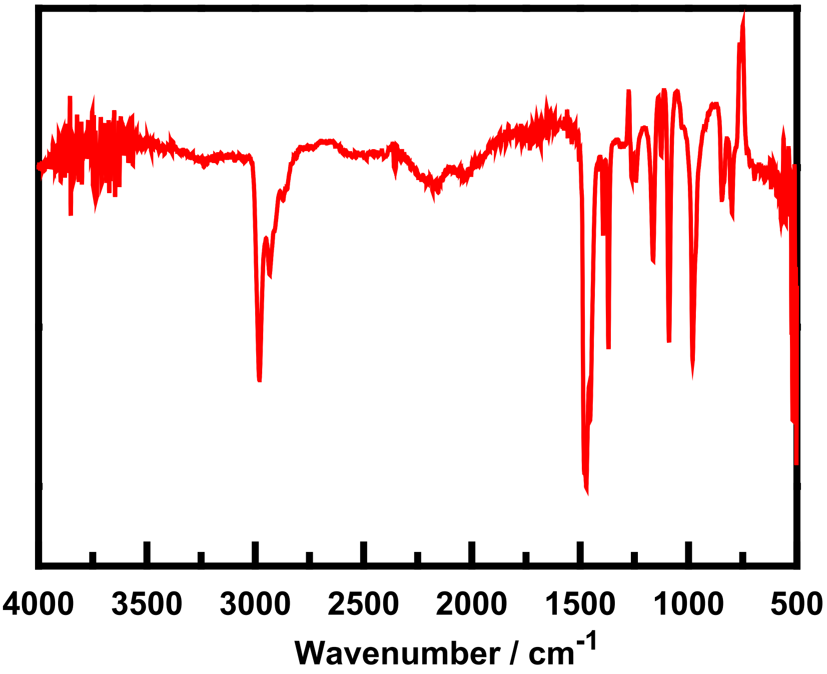

Supplementary Fig. 40. FT-IR spectrum of compound **E7** in thin film at 298 K.

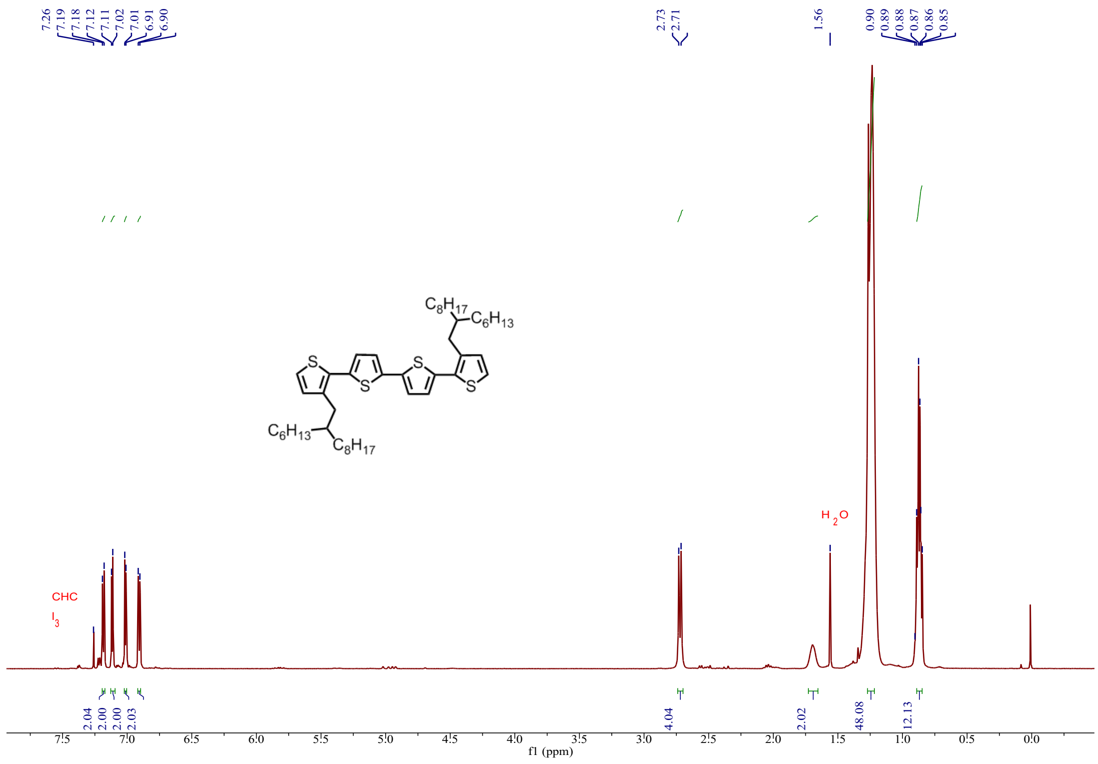

Supplementary Fig. 41. ^{1}H NMR spectrum of compound **8** in $CDCl_3$ at 298 K

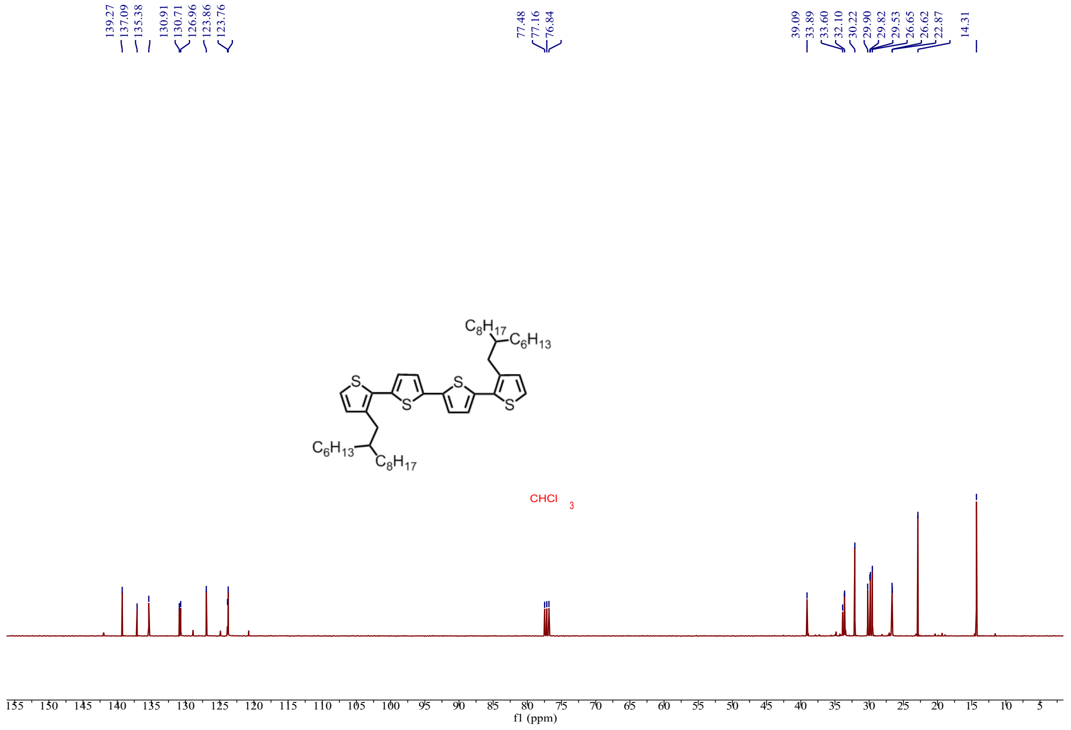

Supplementary Fig. 42. $^{13}C\{^{1}H\}$ NMR spectrum of compound **8** in $CDCl_3$ at 298 K

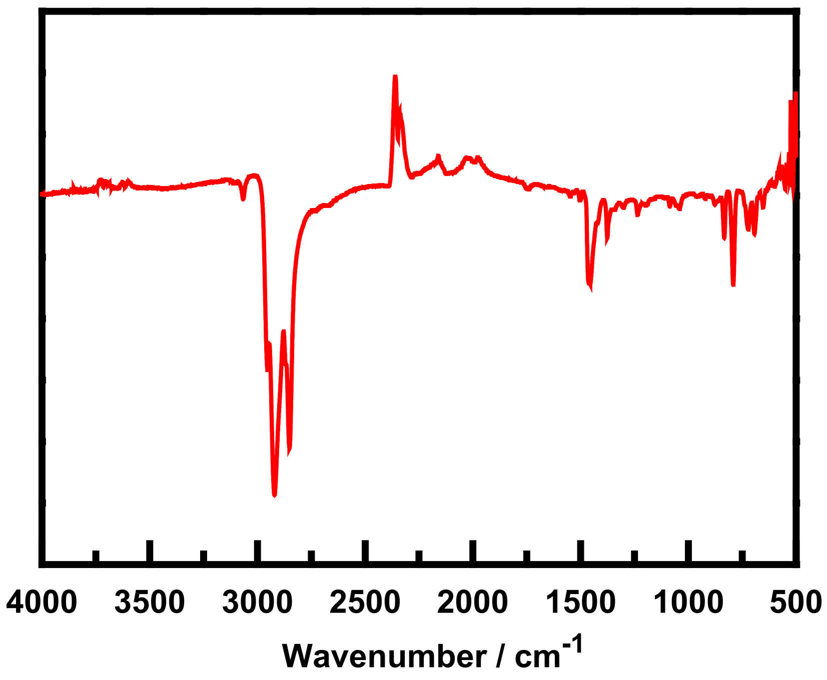

Supplementary Fig. 43. FT-IR spectrum of compound **8** in thin film at 298 K

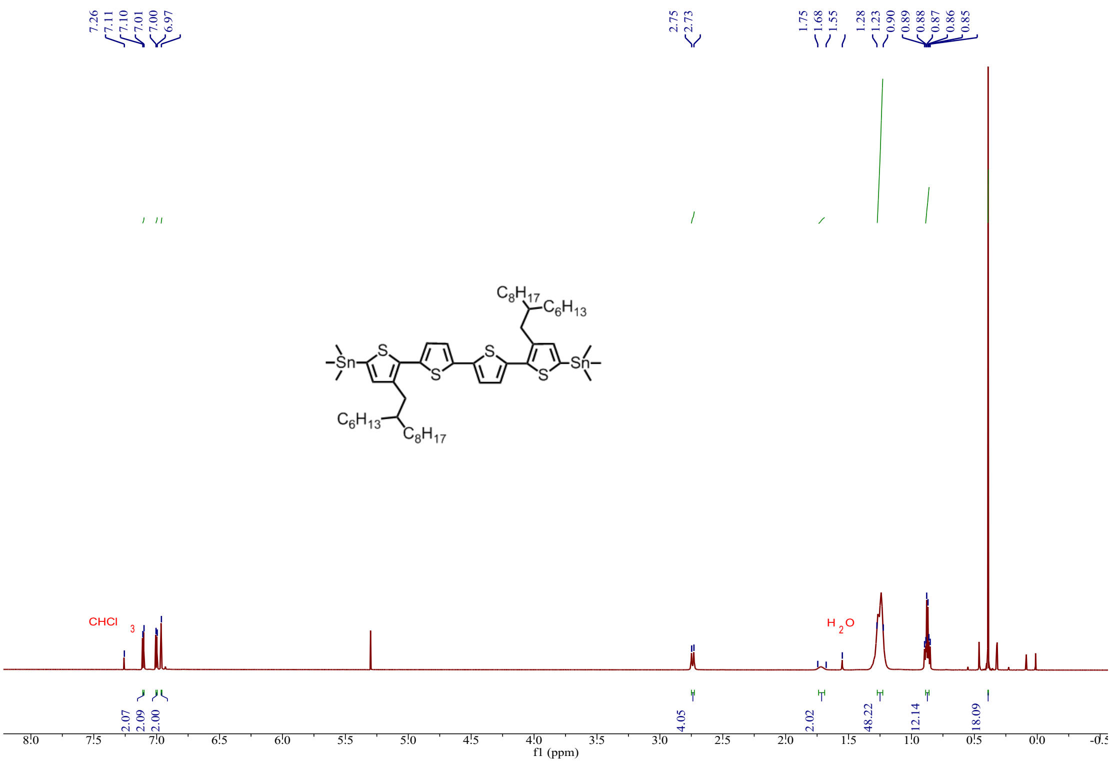

Supplementary Fig. 44. ^{1}H NMR spectrum of compound **N4** in $CDCl_3$ at 298 K

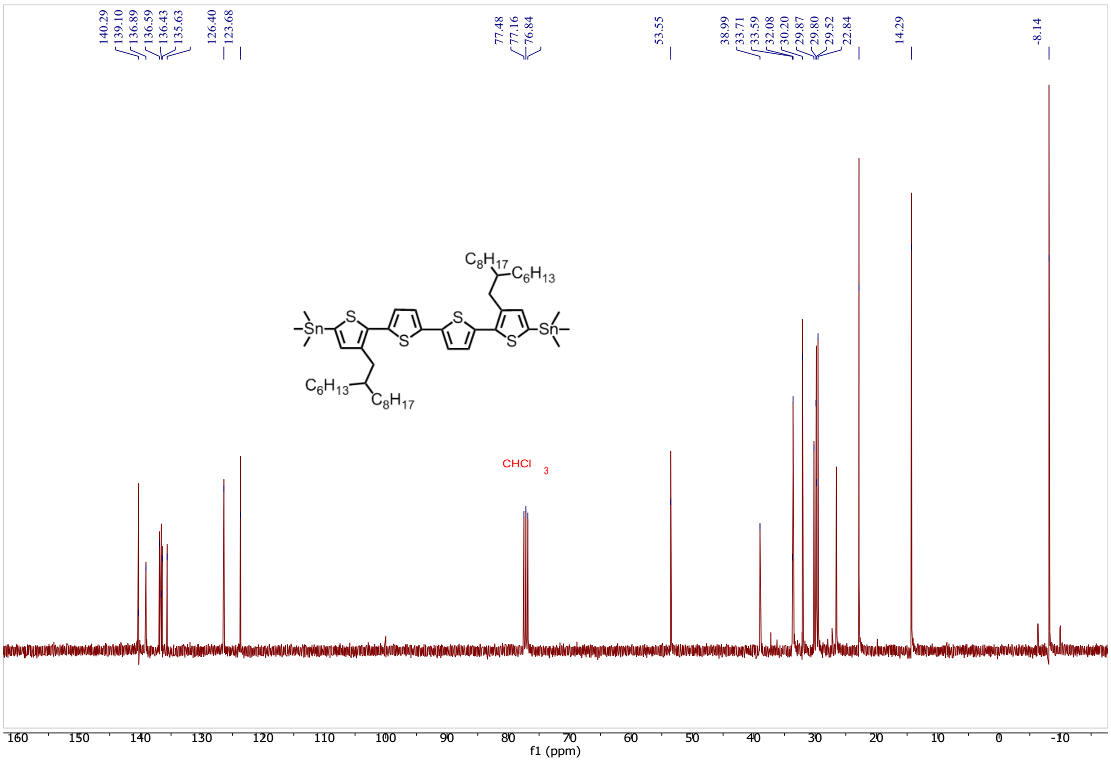

Supplementary Fig. 45. $^{13}C\{^{1}H\}$ NMR spectrum of compound **N4** in $CDCl_3$ at 298 K

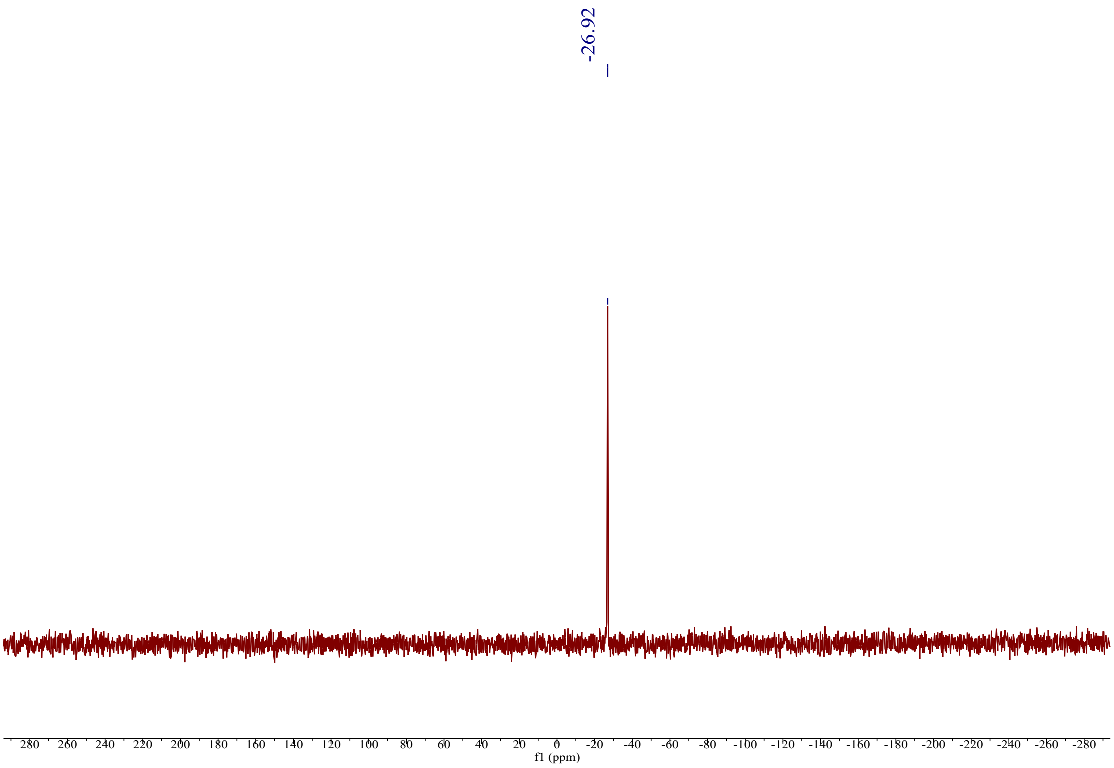

Supplementary Fig. 46. ^{119}Sn NMR spectrum of compound **N4** in $CDCl_3$ at 298 K.

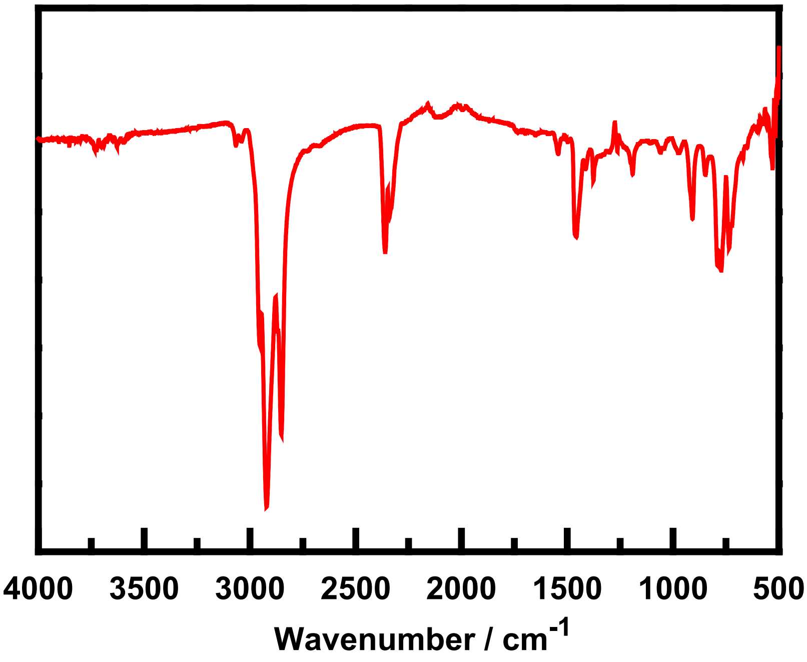

Supplementary Fig. 47. FT-IR spectrum of compound **N4** in thin film at 298 K.

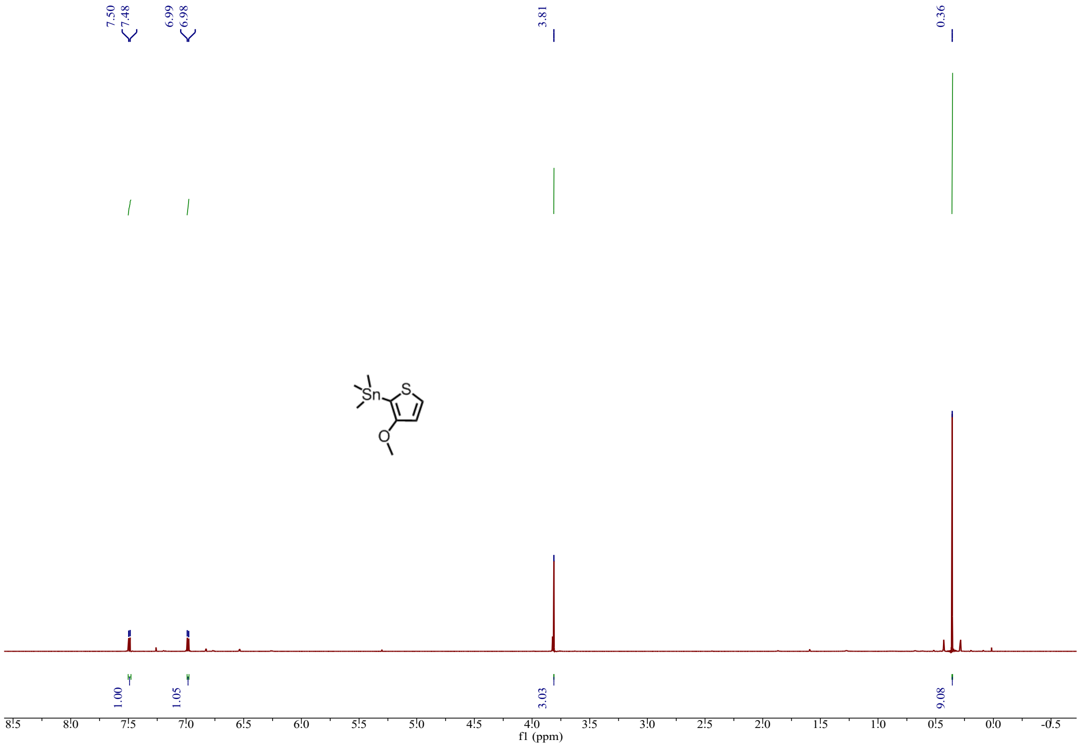

Supplementary Fig. 48. ^{1}H NMR spectrum of compound **12** in $CDCl_3$ at 298 K.

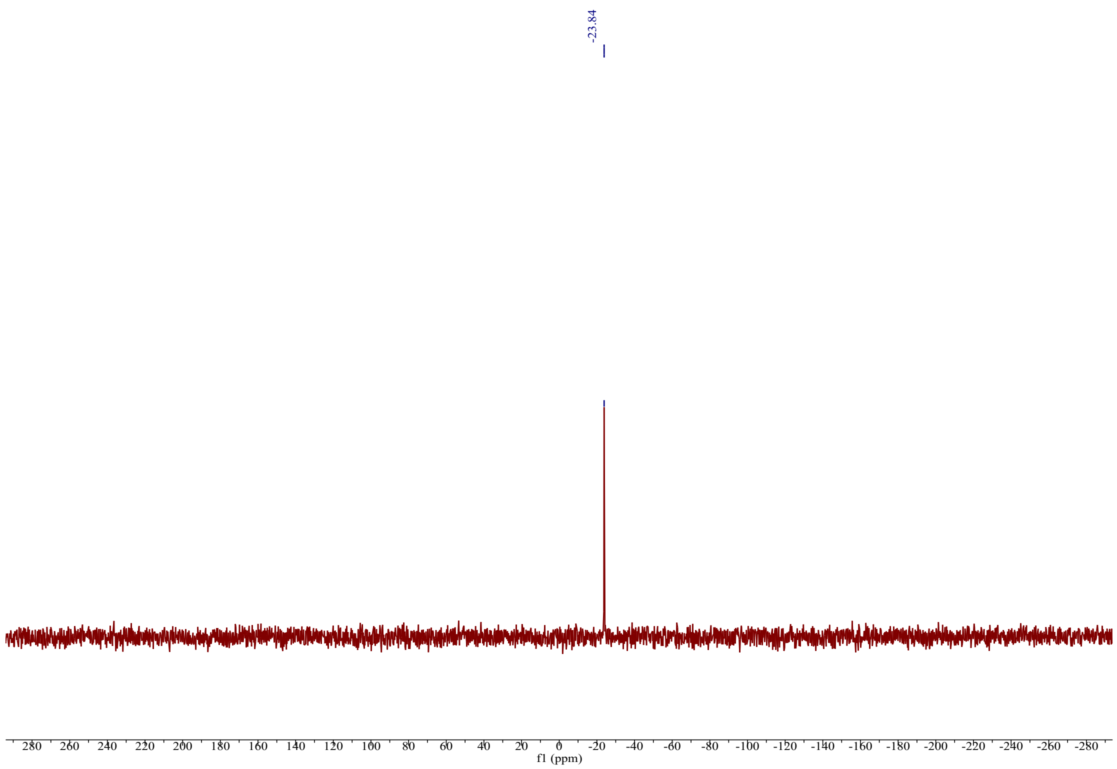

Supplementary Fig. 49. ^{119}Sn NMR spectrum of compound **12** in $CDCl_3$ at 298 K.

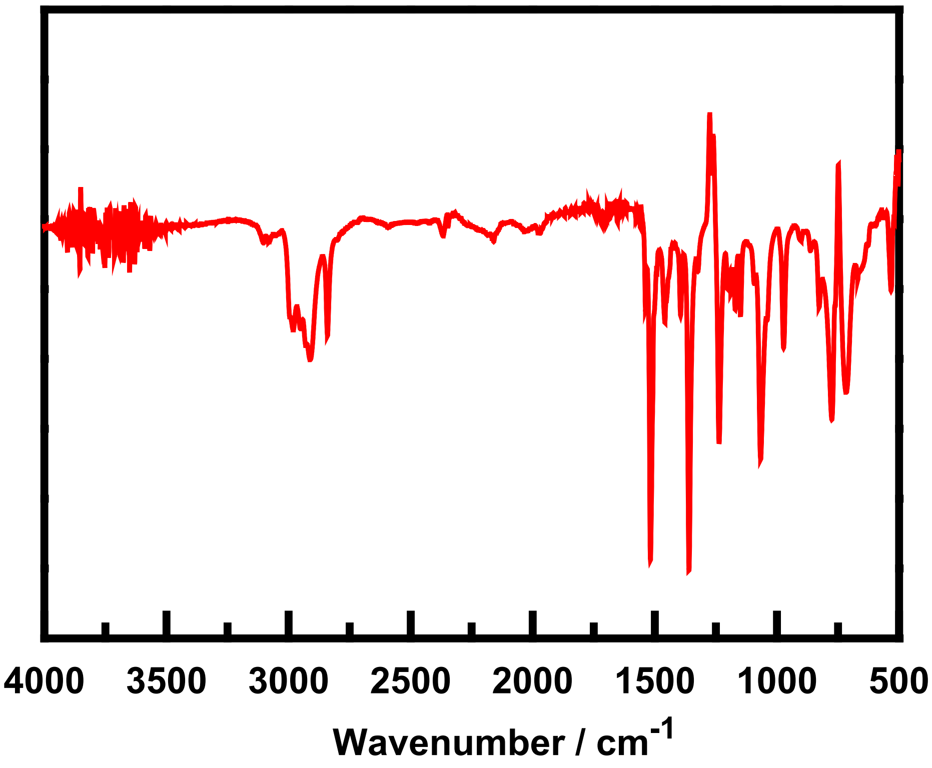

Supplementary Fig. 50. FT-IR spectrum of compound **12** in thin film at 298 K.

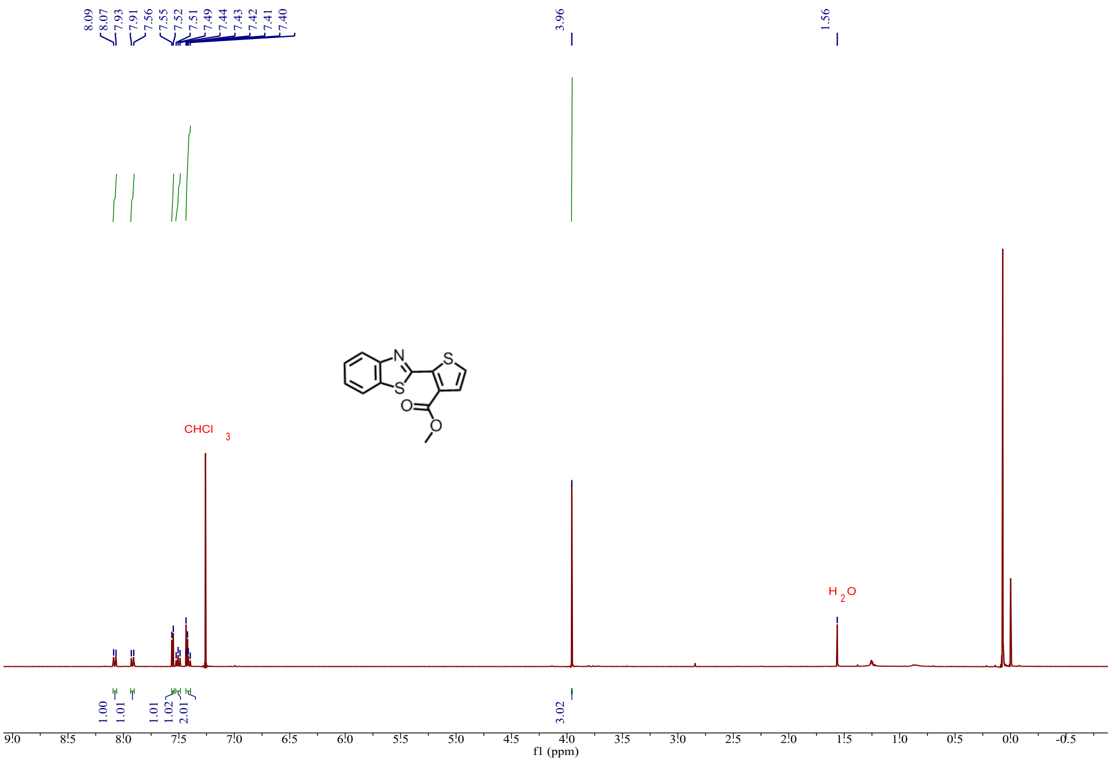

Supplementary Fig. 51. ^{1}H NMR spectrum of compound **S3** in $CDCl_3$ at 298 K

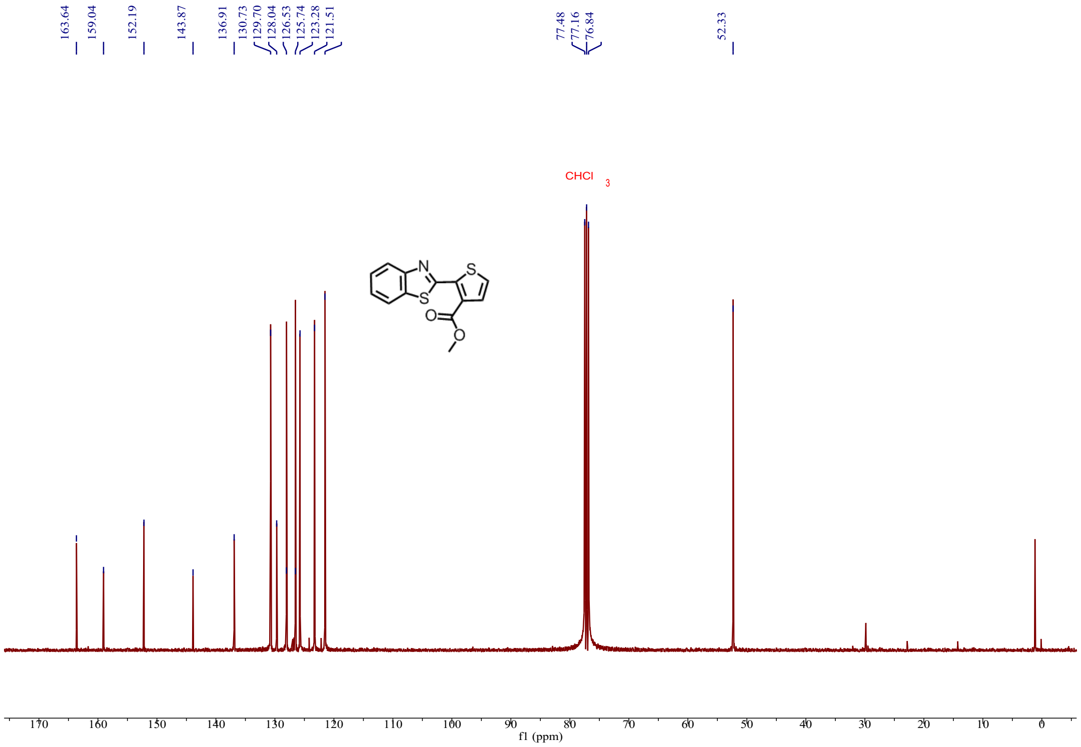

Supplementary Fig. 52. $^{13}C\{^{1}H\}$ NMR spectrum of compound **S3** in $CDCl_3$ at 298 K

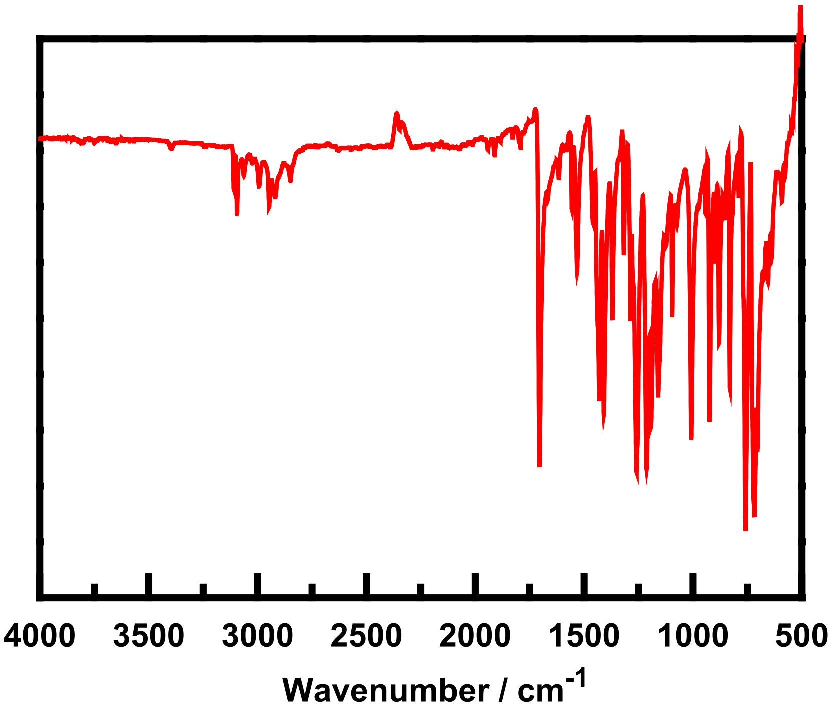

Supplementary Fig. 53. FT-IR spectrum of compound **S3** in thin film at 298 K

Optimization of the polymerization to produce P1

(a)

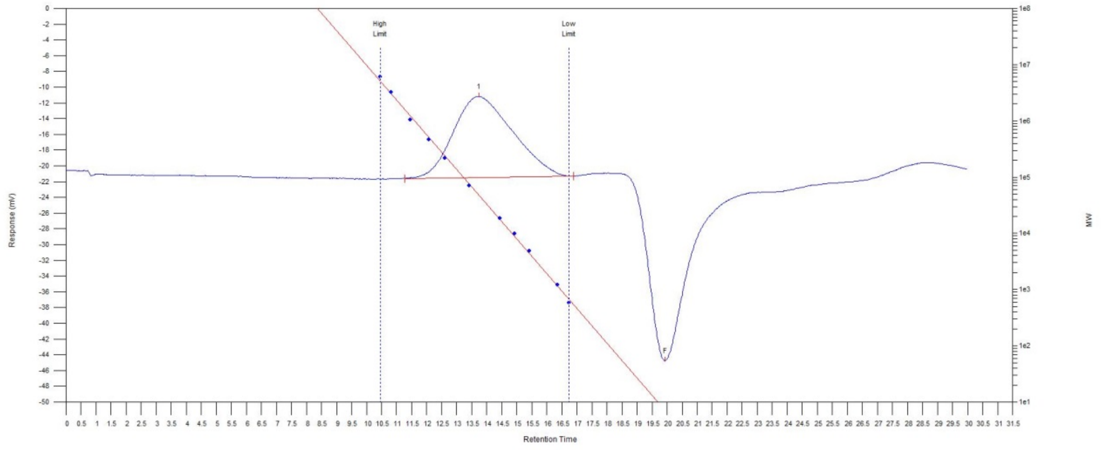

(b)

MW Averages

Mp: 48230 Mn: 14529 Mv: 57850 Mw: 69982

Mz: 206429 Mz+1: 415965 PD: 4.8167

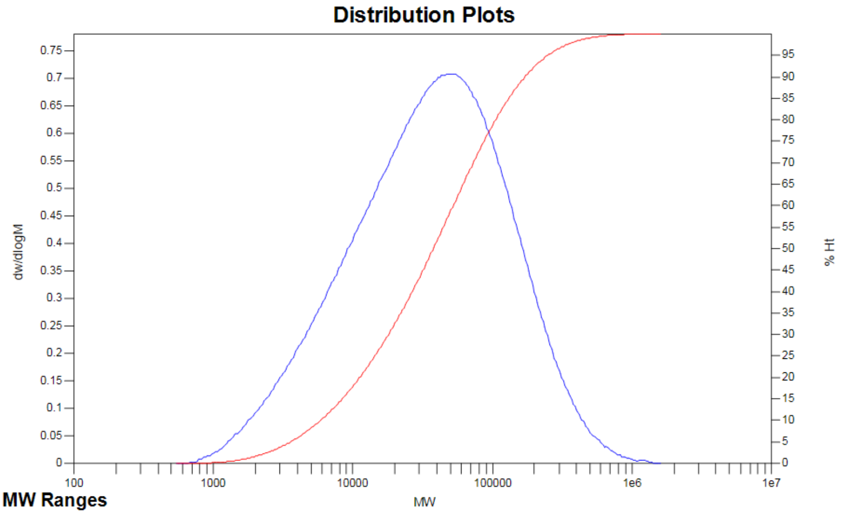

Supplementary Fig. 54. Gel Permeation Chromatography (GPC) trace of **Entry 1** of optimized condition . (a) Trace data from refractive index (RI) detector. (b) Molecular weight distribution plots of **Entry 1** of optimized condition **(Table 1, Entry 1)**.

(a)

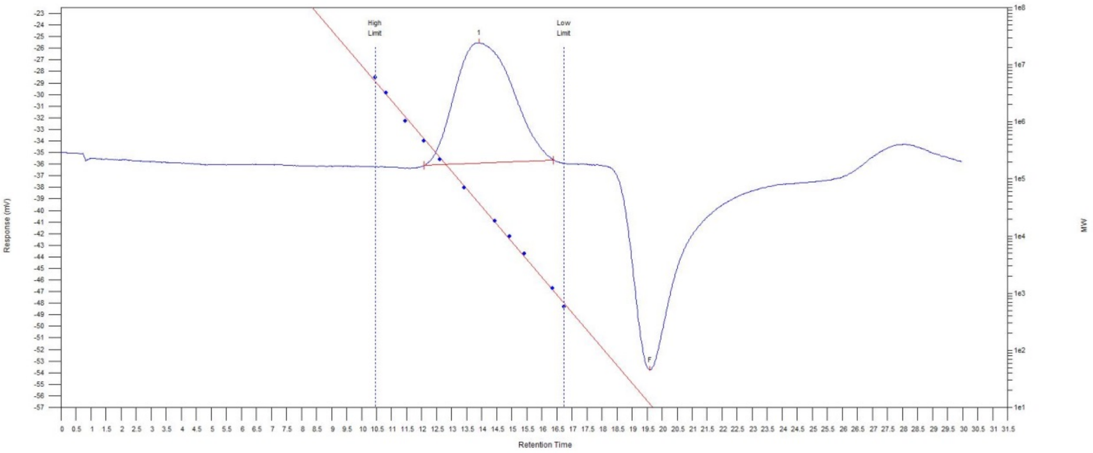

(b)

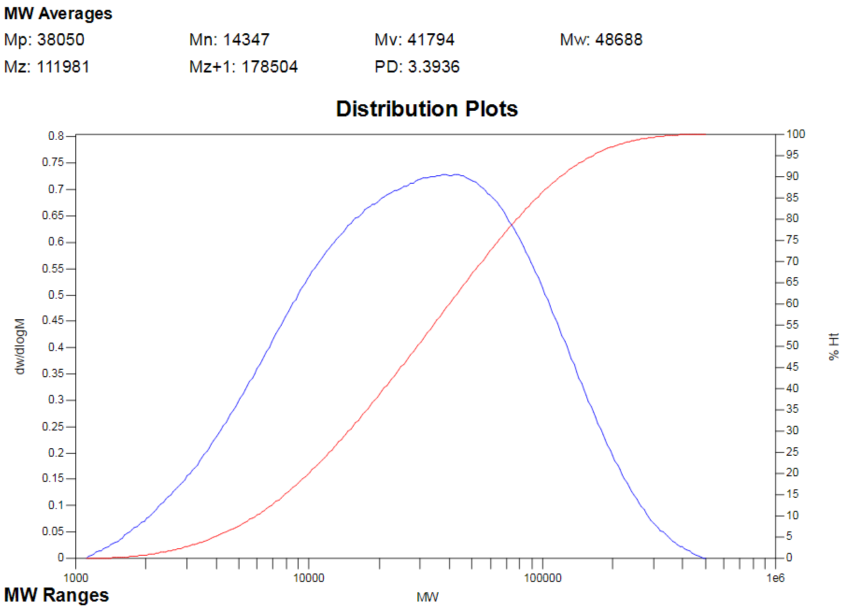

Supplementary Fig. 55. Gel Permeation Chromatography (GPC) trace of **Entry 2** of optimized condition. (a) Trace data from refractive index (RI) detector. (b) Molecular weight distribution plots of **Entry 2** of optimized condition **(Table 1, Entry 2)**.

(a)

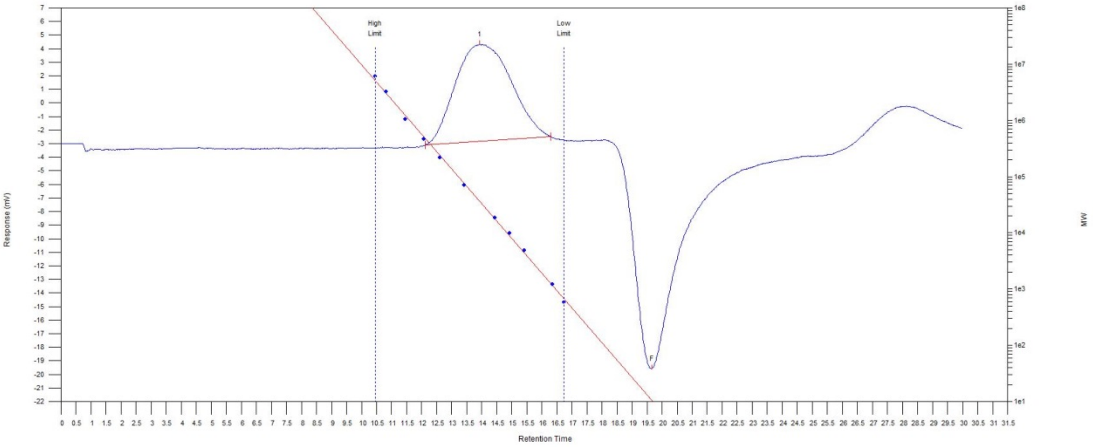

(b)

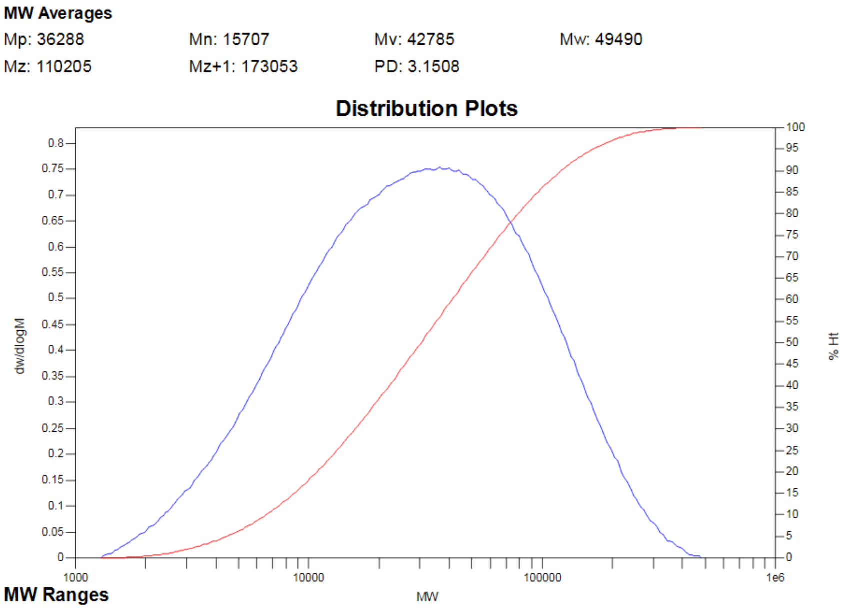

Supplementary Fig. 56. Gel Permeation Chromatography (GPC) trace of **Entry 3** of optimized condition. (a) Trace data from refractive index (RI) detector. (b) Molecular weight distribution plots of **Entry 3** of optimized condition **(Table 1, Entry 3)**.

(a)

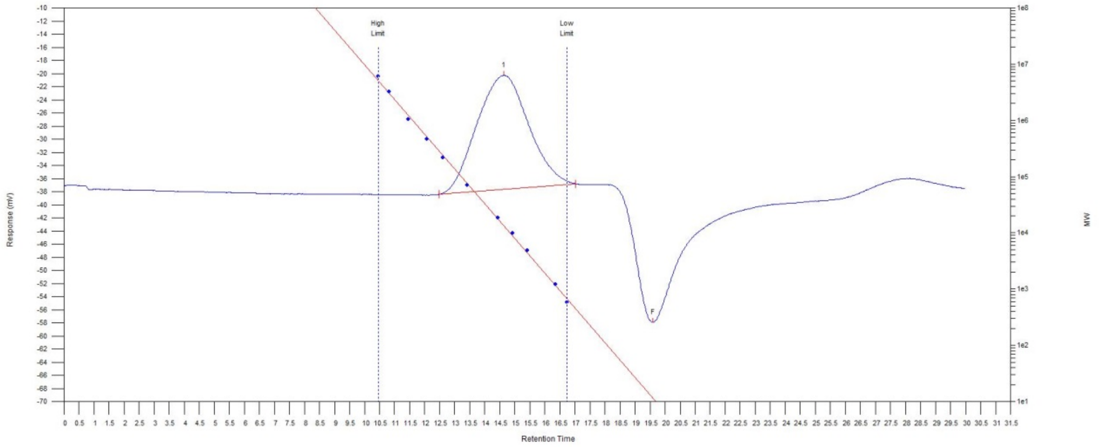

(b)

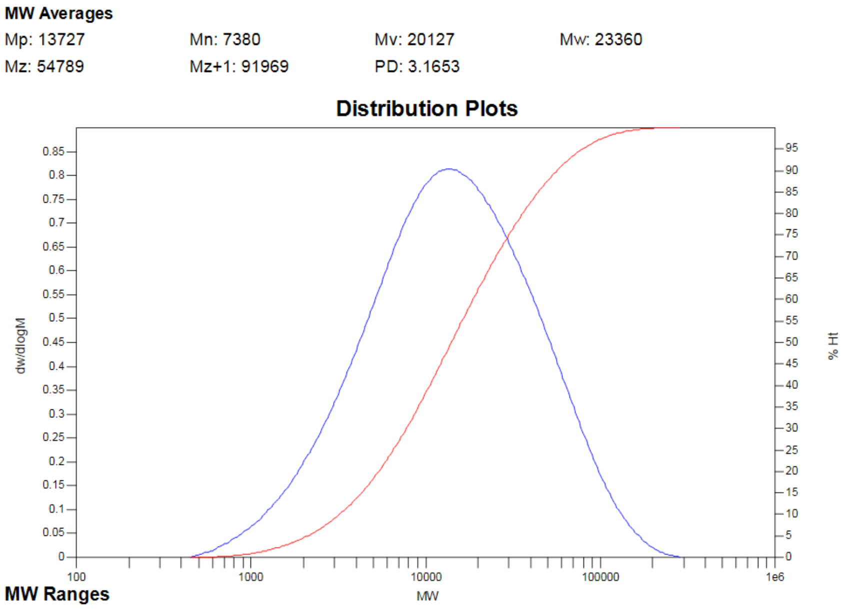

Supplementary Fig. 57. Gel Permeation Chromatography (GPC) trace of **Entry 4** of optimized condition. (a) Trace data from refractive index (RI) detector. (b) Molecular weight distribution plots of **Entry 4** of optimized condition **(Table 1, Entry 4).**

(a)

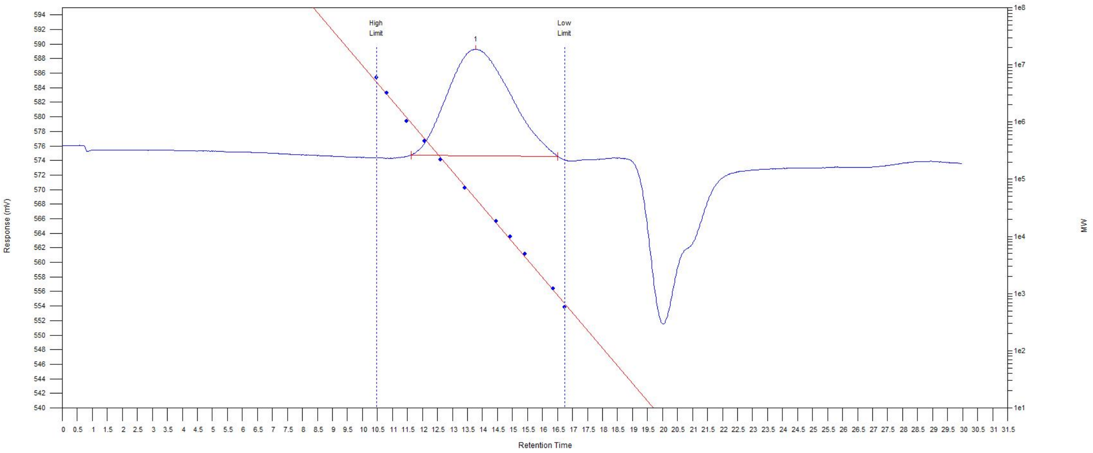

(b)

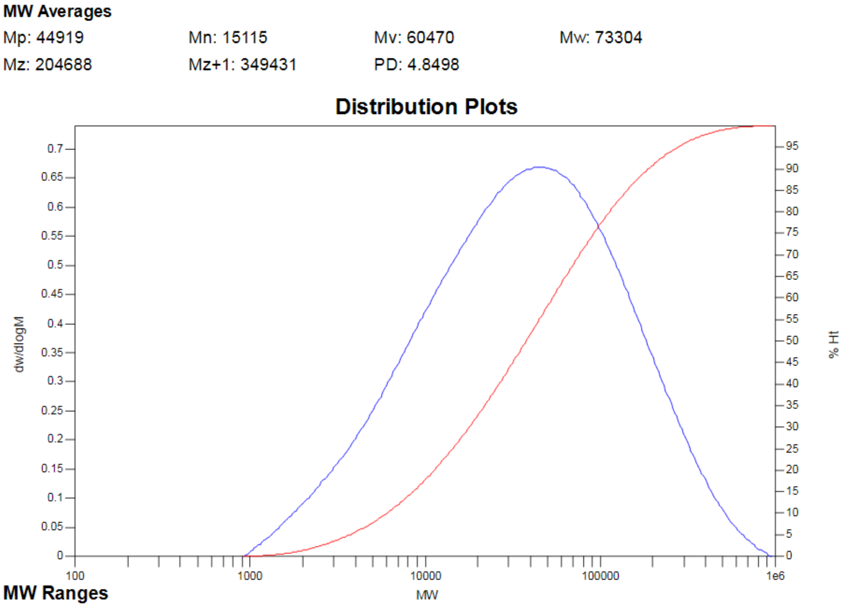

Supplementary Fig. 58. Gel Permeation Chromatography (GPC) trace of **Entry 5** of optimized condition. (a) Trace data from refractive index (RI) detector. (b) Molecular weight distribution plots of **Entry 5** of optimized condition **(Table 1, Entry 5)**.

(a)

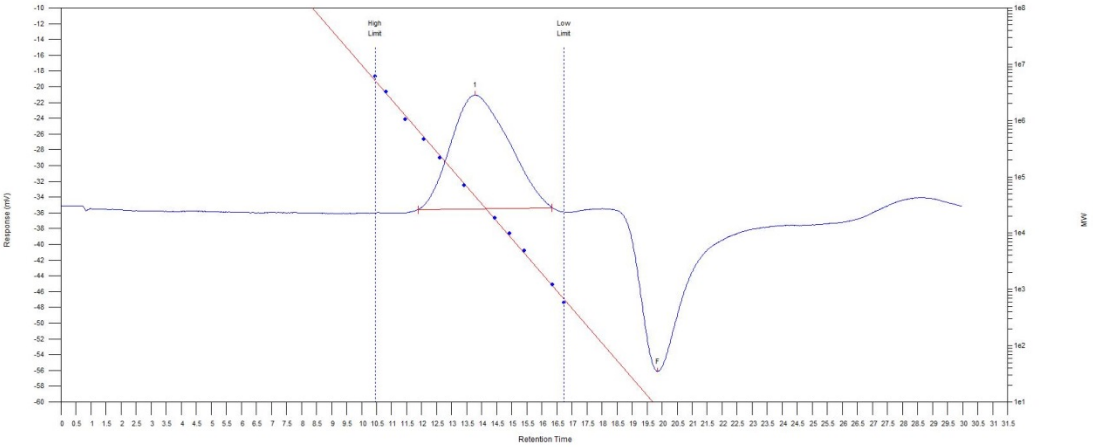

(b)

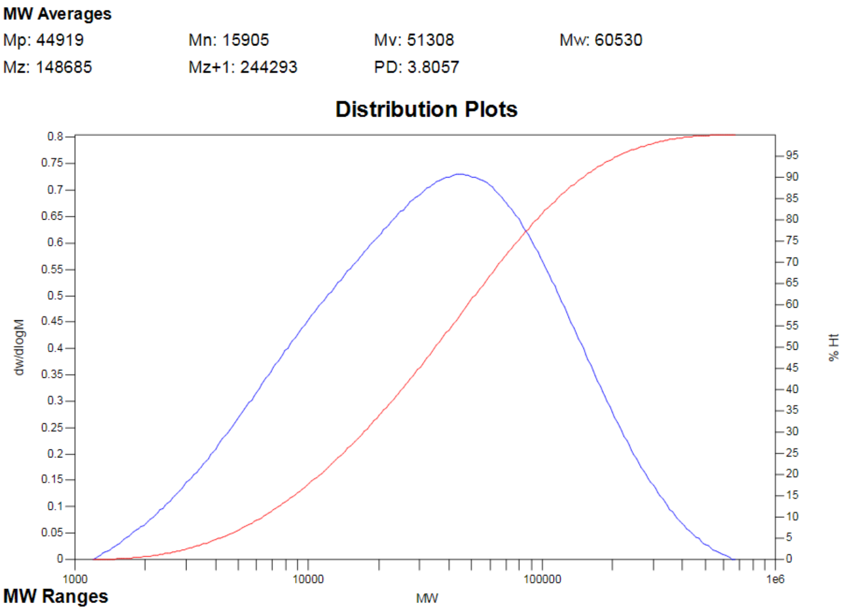

Supplementary Fig. 59. Gel Permeation Chromatography (GPC) trace of **Entry 6** of optimized condition. (a) Trace data from refractive index (RI) detector. (b) Molecular weight distribution plots of **Entry 6** of optimized condition **(Table 1, Entry 6)**.

(a)

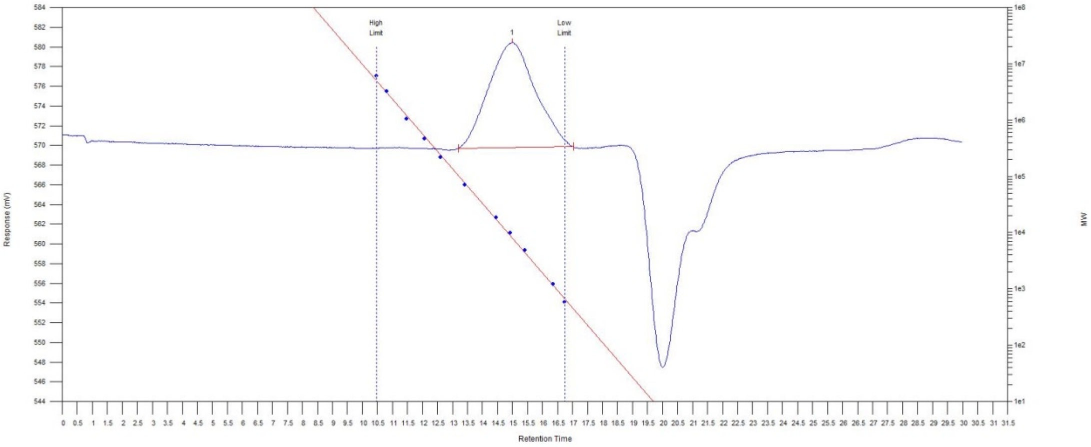

(b)

MW Averages

Mp: 8148	Mn: 4661	Mv: 10767	Mw: 12213
Mz: 25213	Mz+1: 39312	PD: 2.6203	

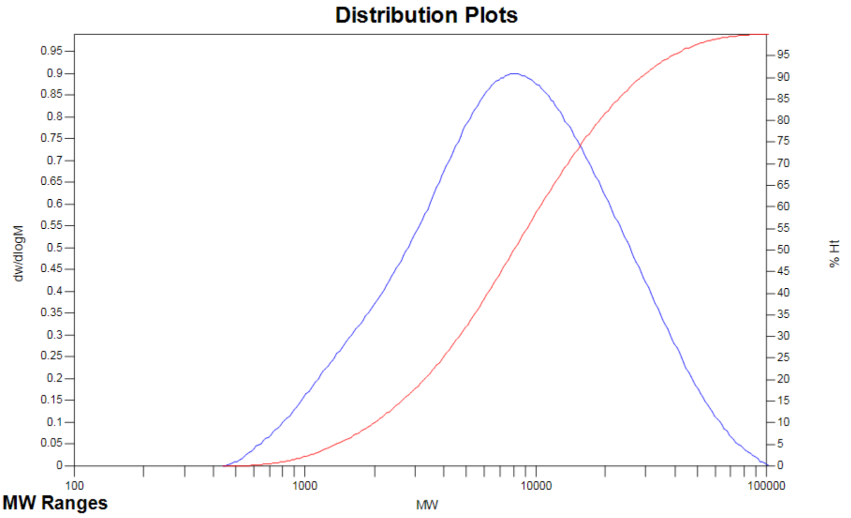

Supplementary Fig. 60. Gel Permeation Chromatography (GPC) trace of **Entry 7** of optimized condition. (a) Trace data from refractive index (RI) detector. (b) Molecular weight distribution plots of **Entry 7** of optimized condition **(Table 1, Entry 7)**.

(a)

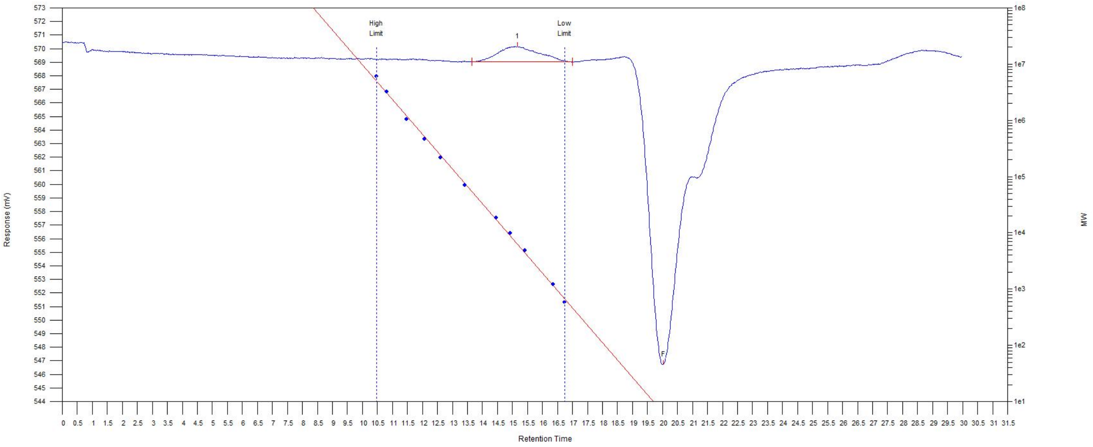

(b)

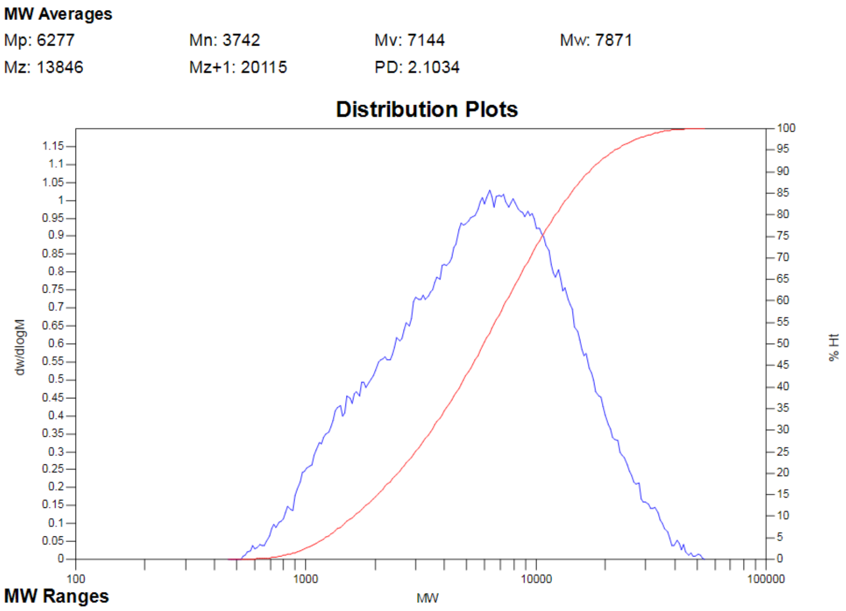

Supplementary Fig. 61. Gel Permeation Chromatography (GPC) trace of **Entry 8** of optimized condition. (a) Trace data from refractive index (RI) detector. (b) Molecular weight distribution plots of **Entry 8** of optimized condition **(Table 1, Entry 8)**.

(a)

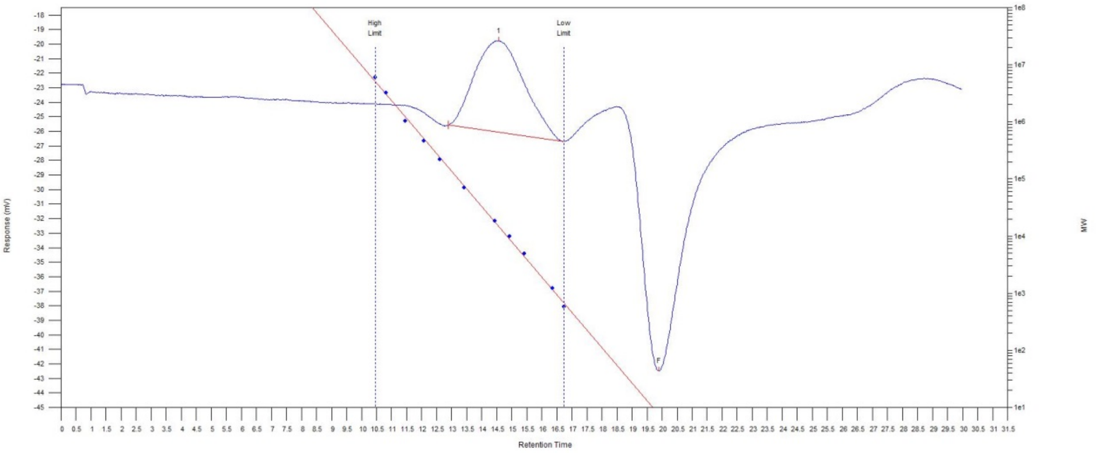

(b)

MW Averages

Mp: 14739	Mn: 7821	Mv: 18408	Mw: 20851
Mz: 41869	Mz+1: 62905	PD: 2.6660	

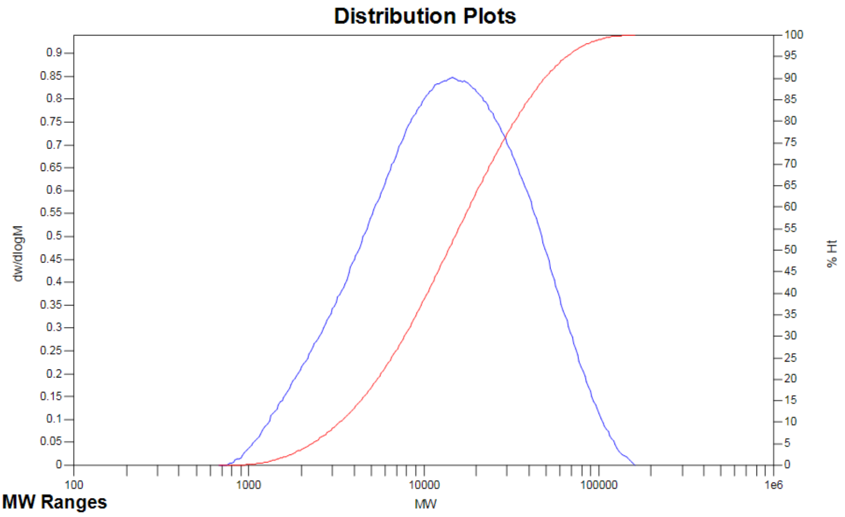

Supplementary Fig. 62. Gel Permeation Chromatography (GPC) trace of **Entry 9** of optimized condition. (a) Trace data from refractive index (RI) detector. (b) Molecular weight distribution plots of **Entry 9** of optimized condition **(Table 1, Entry 9)**.

(a)

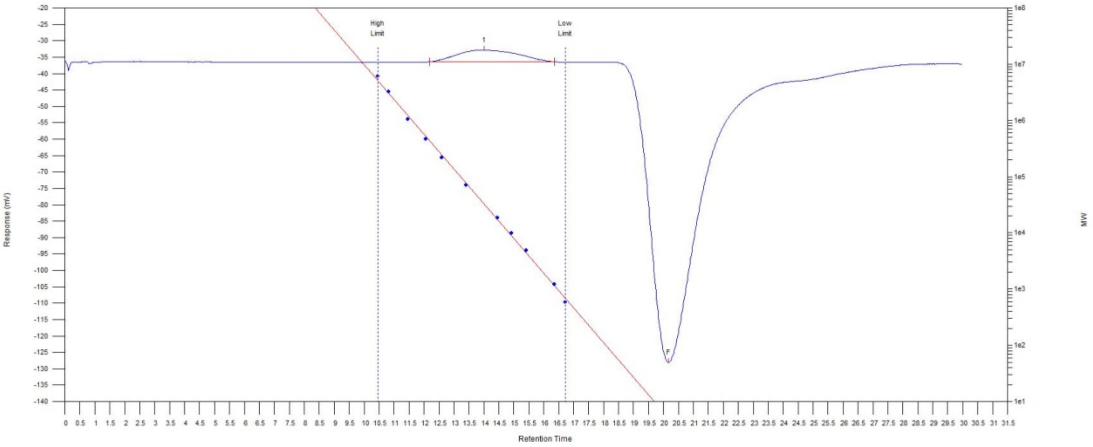

(b)

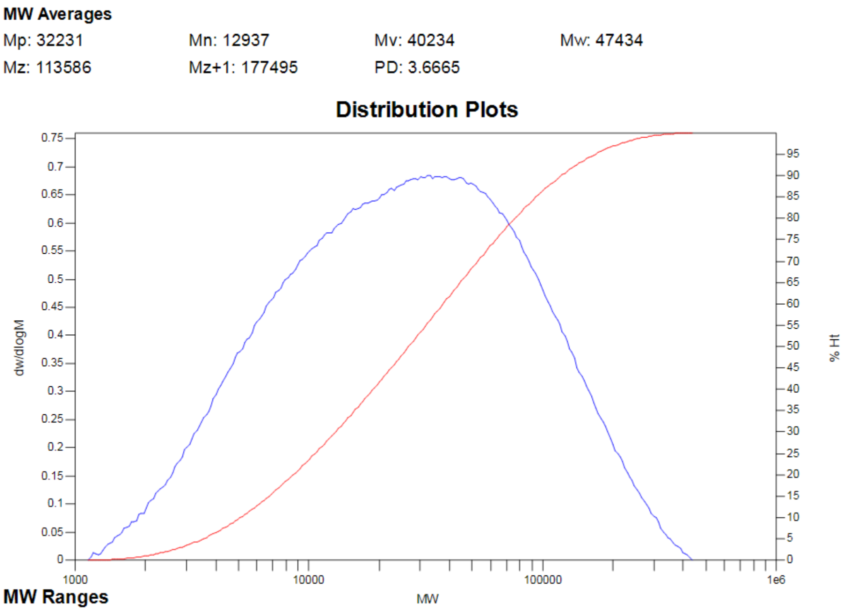

Supplementary Fig. 63. Gel Permeation Chromatography (GPC) trace of **Entry 2** of optimized condition. (a) Trace data from refractive index (RI) detector. (b) Molecular weight distribution plots of **Entry 2** of optimized condition **(Supplementart Table 1, Entry 2)**.

(a)

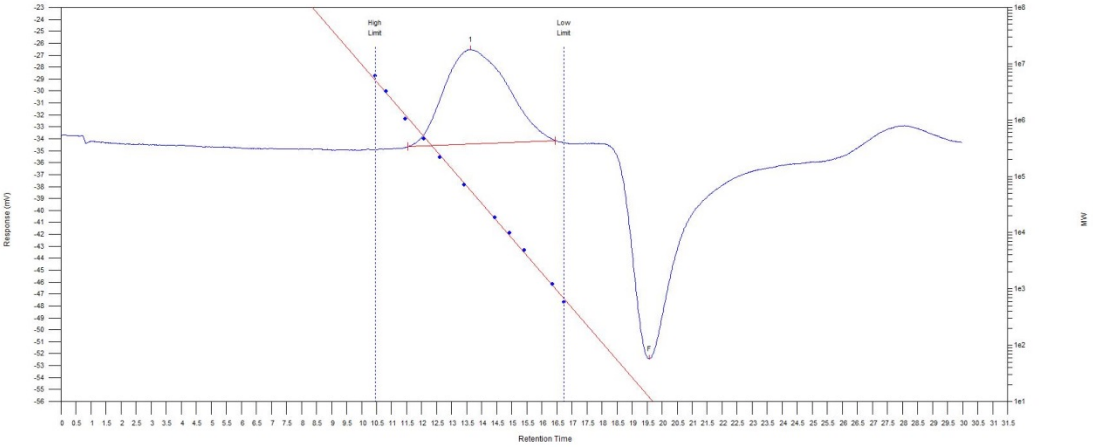

(b)

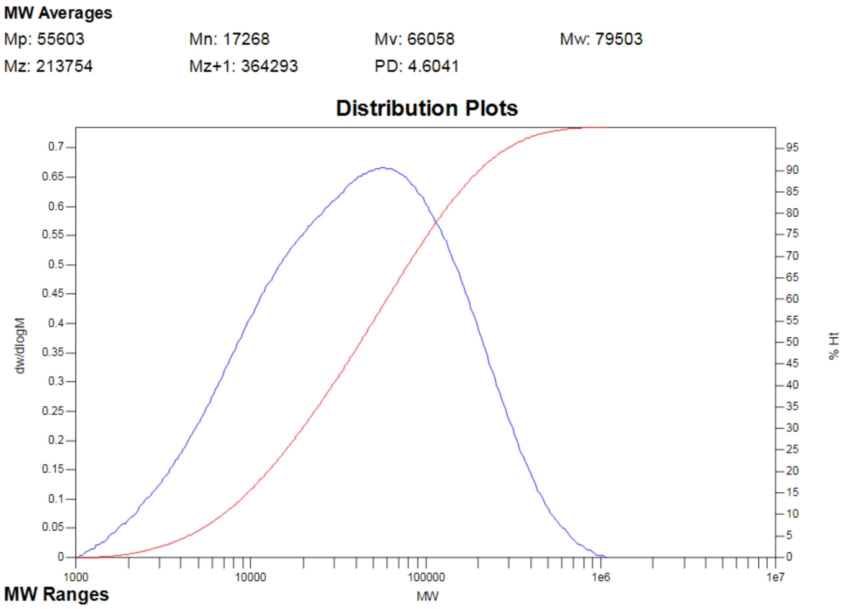

Supplementary Fig. 64. Gel Permeation Chromatography (GPC) trace of **Entry 3** of optimized condition. (a) Trace data from refractive index (RI) detector. (b) Molecular weight distribution plots of **Entry 3** of optimized condition **(Supplementary Table 1, Entry 3)**.

(a)

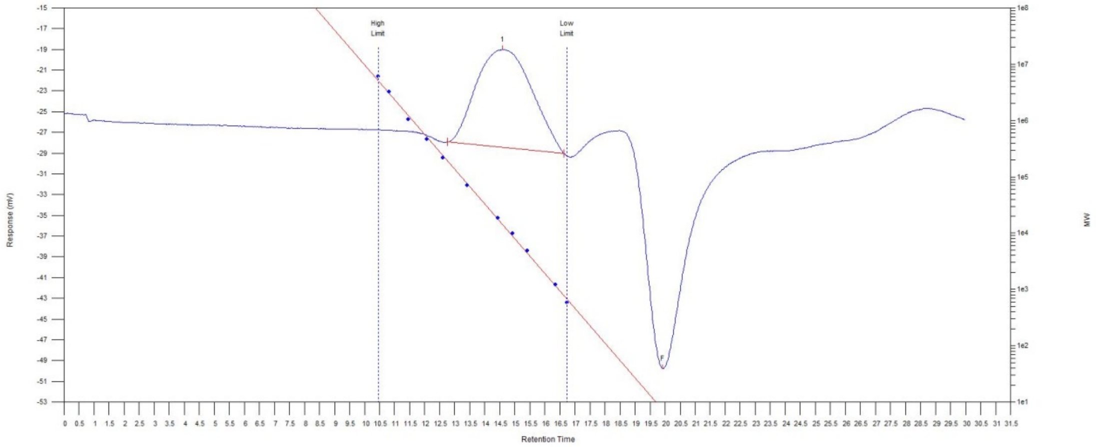

(b)

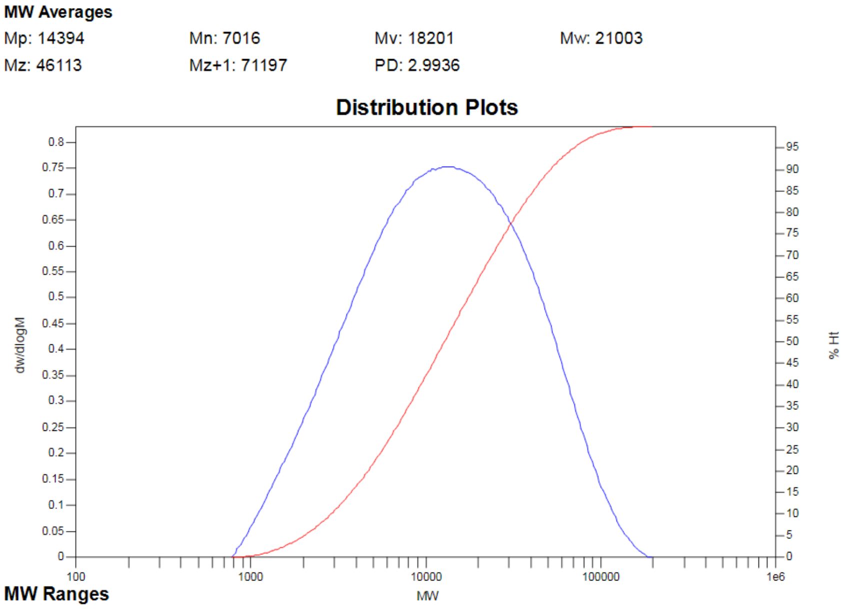

Supplementary Fig. 65. Gel Permeation Chromatography (GPC) trace of **Entry 4** of optimized condition. (a) Trace data from refractive index (RI) detector. (b) Molecular weight distribution plots of **Entry 4** of optimized condition **(Supplementary Table 1, Entry 4)**.

(a)

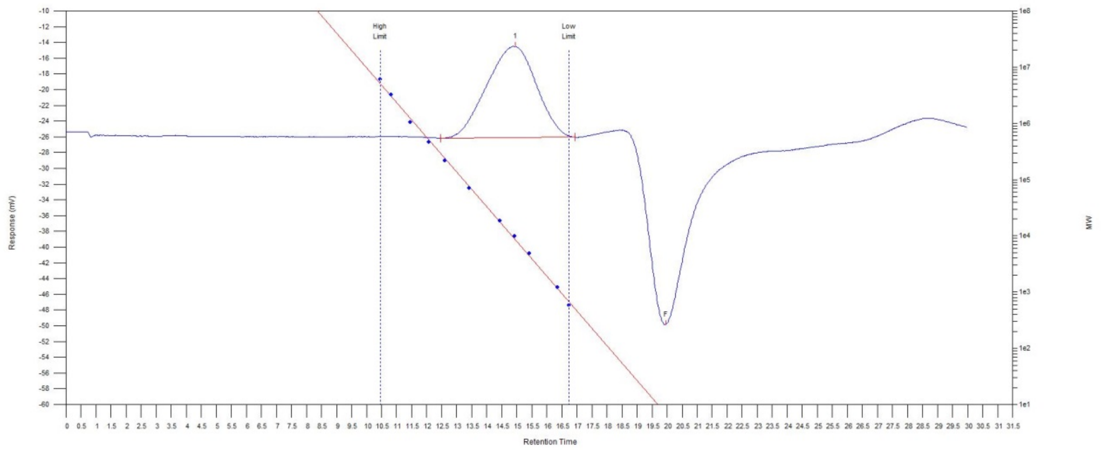

(b)

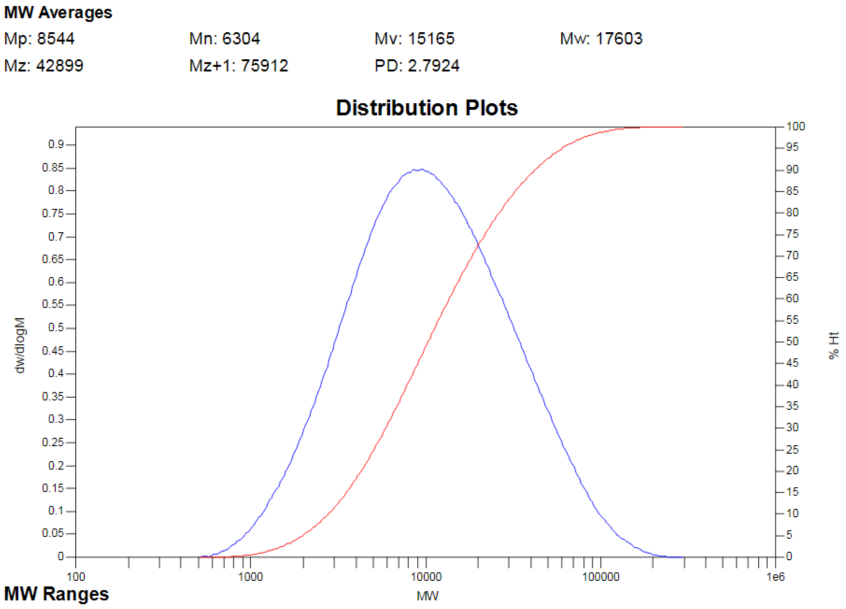

Supplementary Fig. 66. Gel Permeation Chromatography (GPC) trace of **Entry 5** of optimized condition. (a) Trace data from refractive index (RI) detector. (b) Molecular weight distribution plots of **Entry 5** of optimized condition **(Supplementary Table 1, Entry 5)**.

(a)

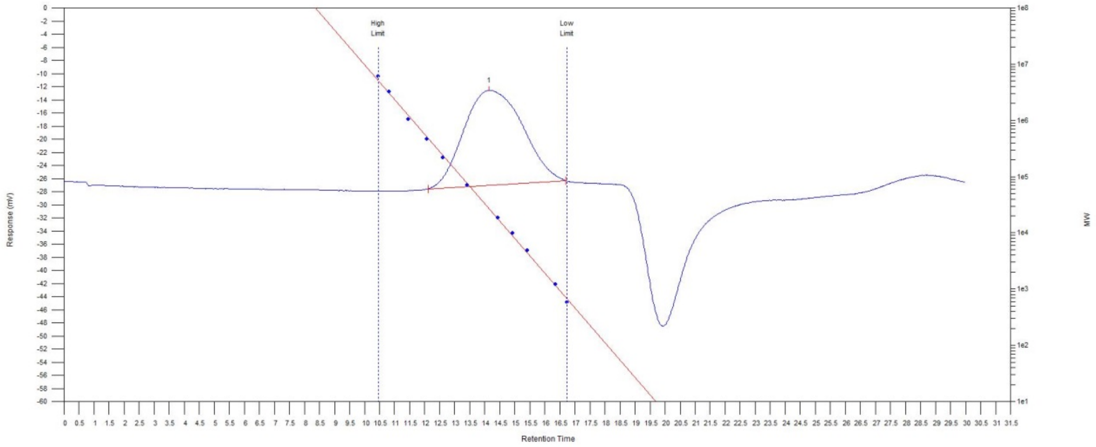

(b)

MW Averages

Mp: 27302	Mn: 9544	Mv: 32189	Mw: 38259
Mz: 99062	Mz+1: 170661	PD: 4.0087	

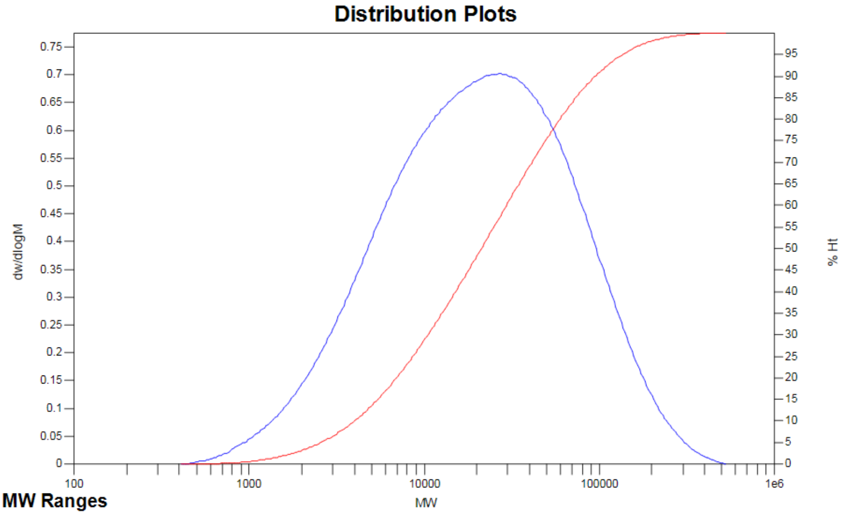

Supplementary Fig. 67. Gel Permeation Chromatography (GPC) trace of **Entry 7** of optimized condition. (a) Trace data from refractive index (RI) detector. (b) Molecular weight distribution plots of **Entry 7** of optimized condition **(Supplementary Table 1, Entry 7)**.

(a)

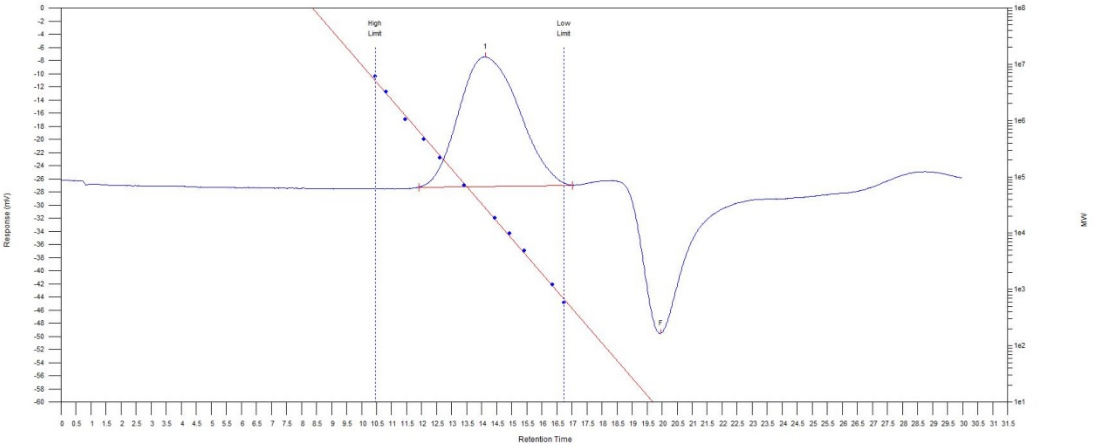

(b)

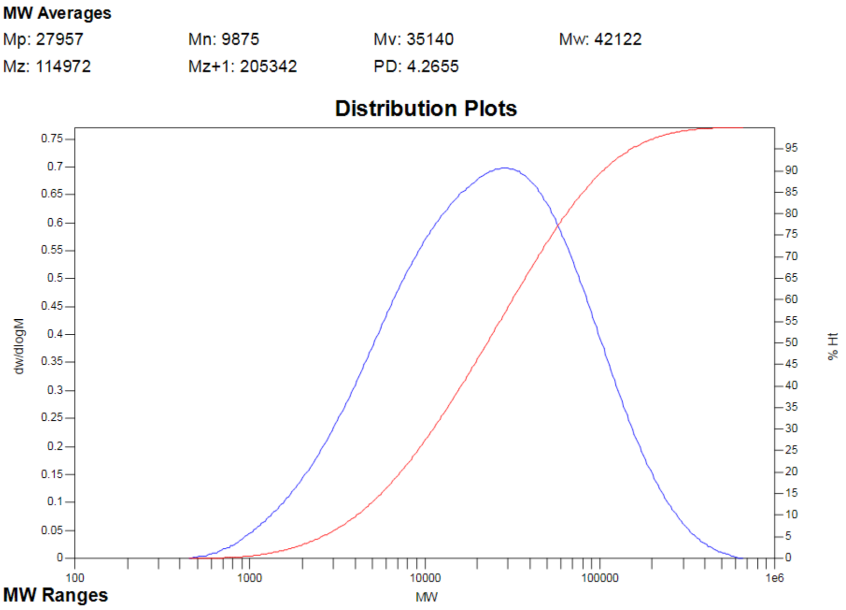

Supplementary Fig. 68. Gel Permeation Chromatography (GPC) trace of **Entry 8** of optimized condition. (a) Trace data from refractive index (RI) detector. (b) Molecular weight distribution plots of **Entry 8** of optimized condition **(Supplementary Table 1, Entry 8)**.

(a)

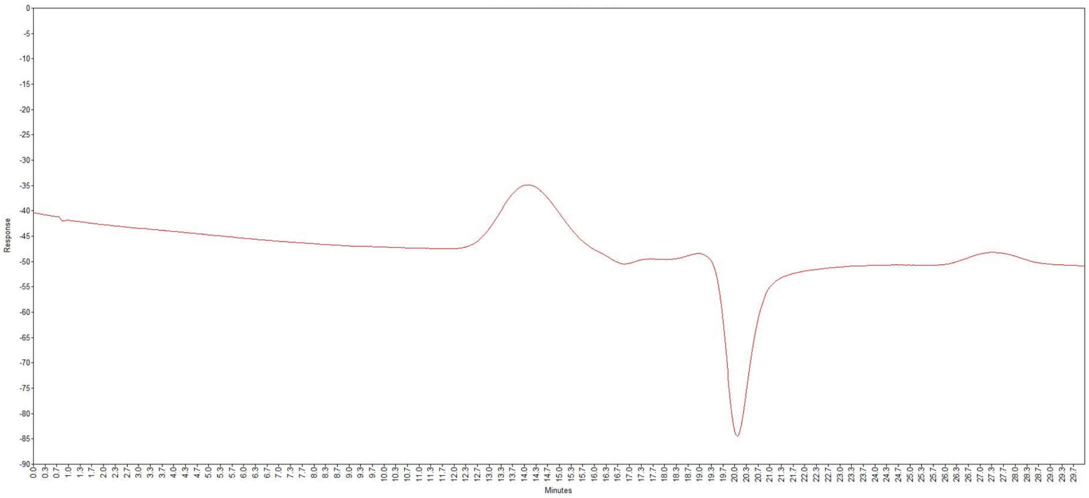

(b)

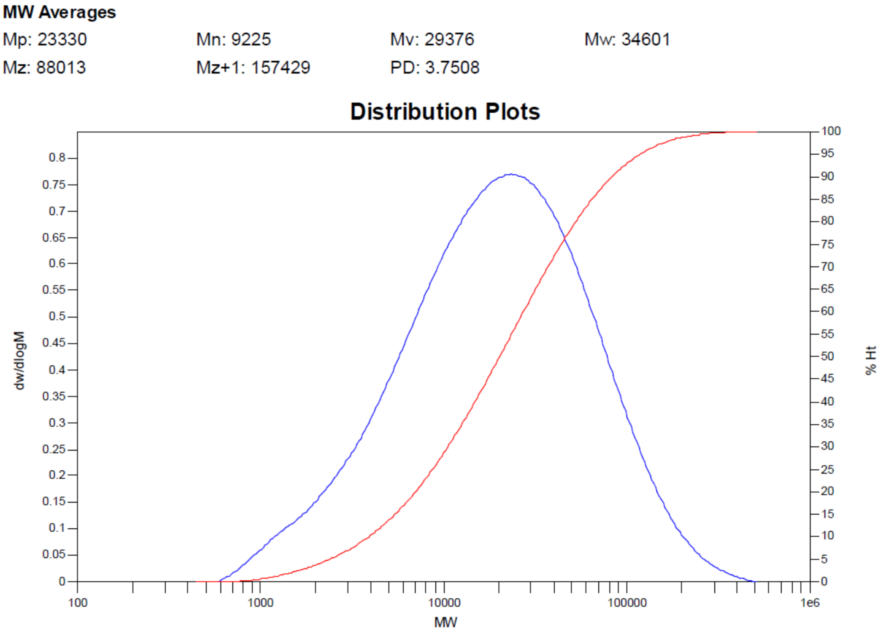

Supplementary Fig. 69. Gel Permeation Chromatography (GPC) trace of **Entry 9** of optimized condition. (a) Trace data from refractive index (RI) detector. (b) Molecular weight distribution plots of **Entry 9** of optimized condition **(Supplementary Table 1, Entry 9)**.

Reproducibility Experiments

(a)

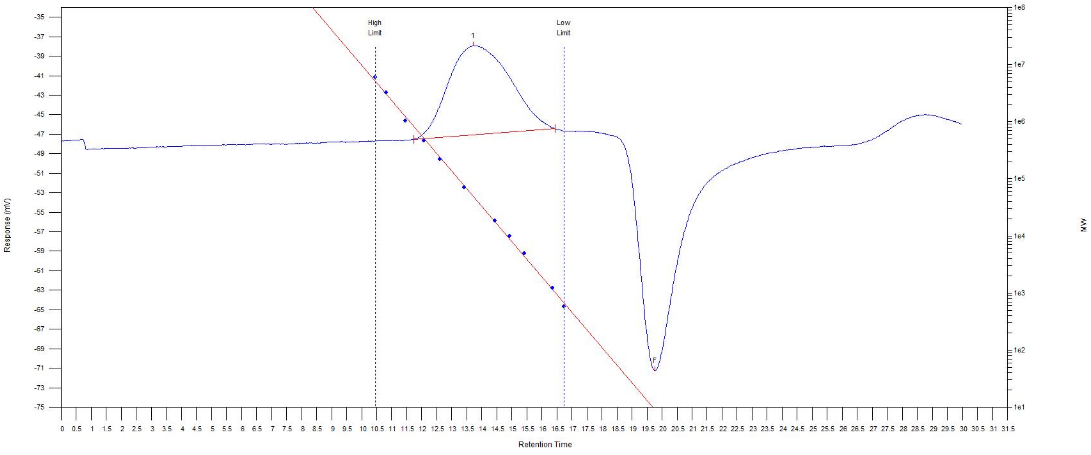

(b)

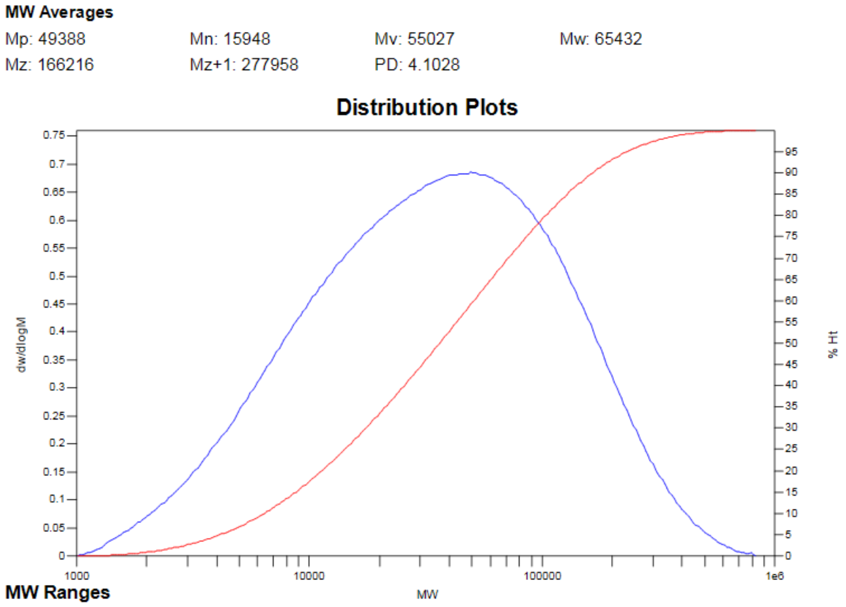

Supplementary Fig. 70. Gel Permeation Chromatography (GPC) trace of **Entry 1** for reproducibility experiment. (a) Trace data from refractive index (RI) detector. (b) Molecular weight distribution plots of **Entry 1** of reproducibility experiments **(Table S1, Entry 1)**.

(a)

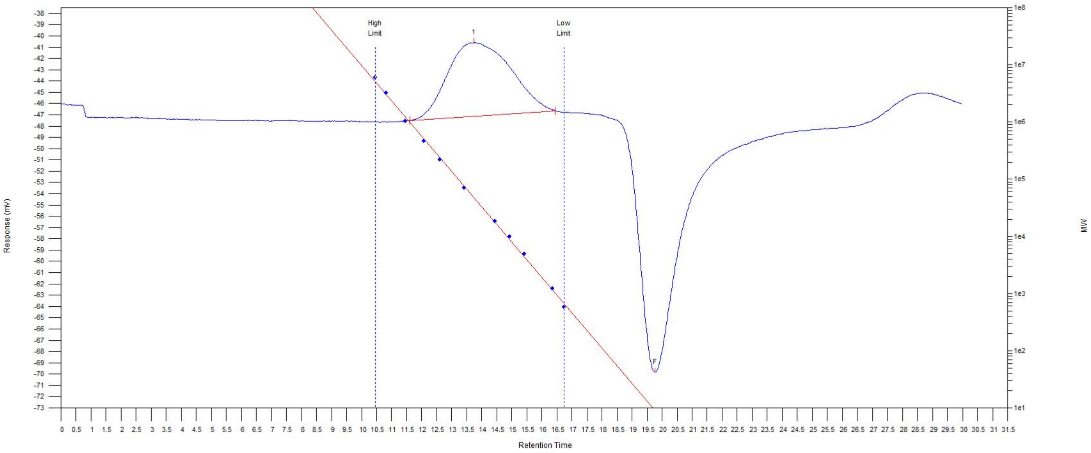

(b)

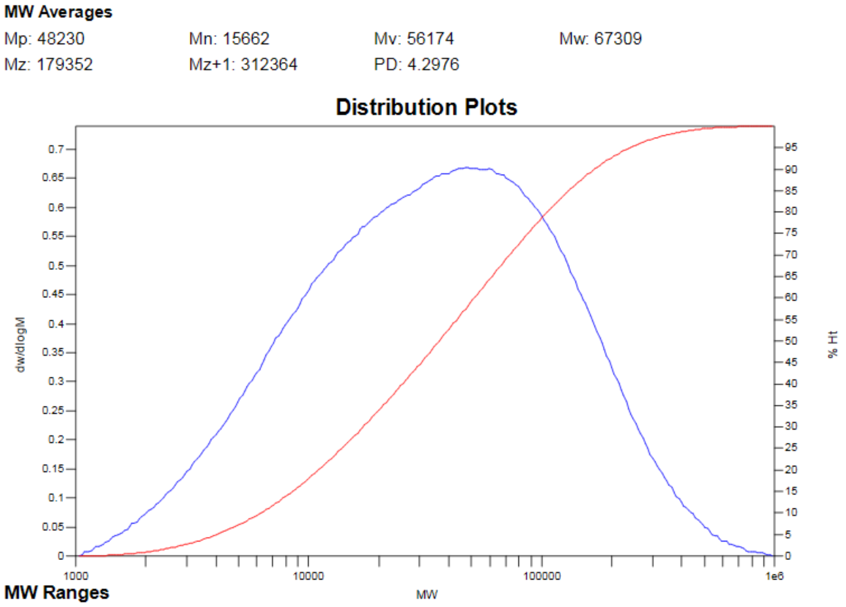

Supplementary Fig. 71. Gel Permeation Chromatography (GPC) trace of **Entry 2** for reproducibility experiment. (a) Trace data from refractive index (RI) detector. (b) Molecular weight distribution plots of **Entry 2** of reproducibility experiments **(Table S1, Entry 2)**.

(a)

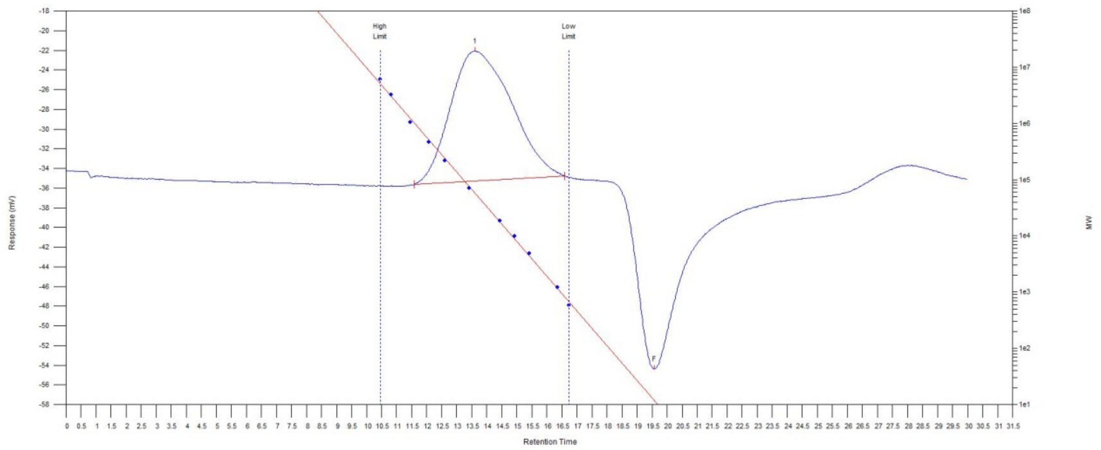

(b)

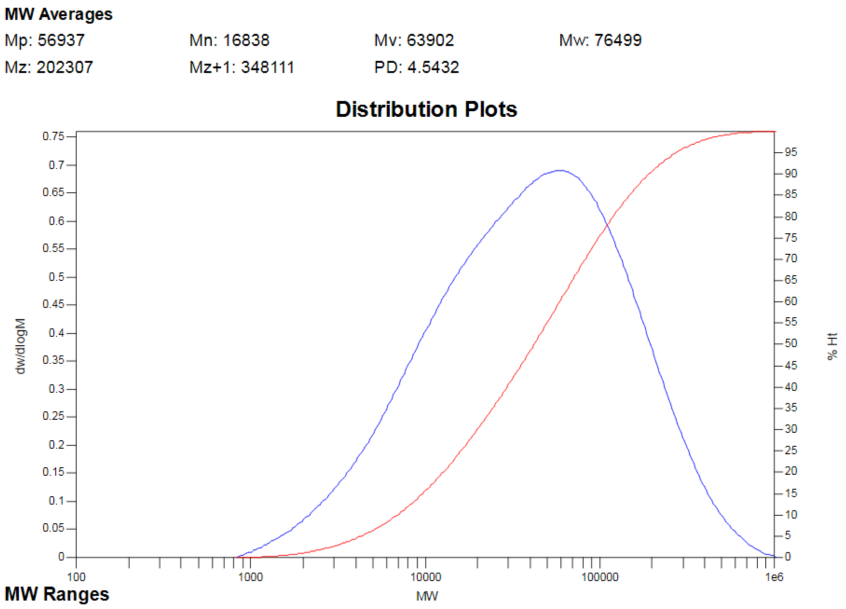

Supplementary Fig. 72. Gel Permeation Chromatography (GPC) trace of **Entry 3** for reproducibility experiment. (a) Trace data from refractive index (RI) detector. (b) Molecular weight distribution plots of **Entry 3** of reproducibility experiments **(Table S1, Entry 3)**.

(a)

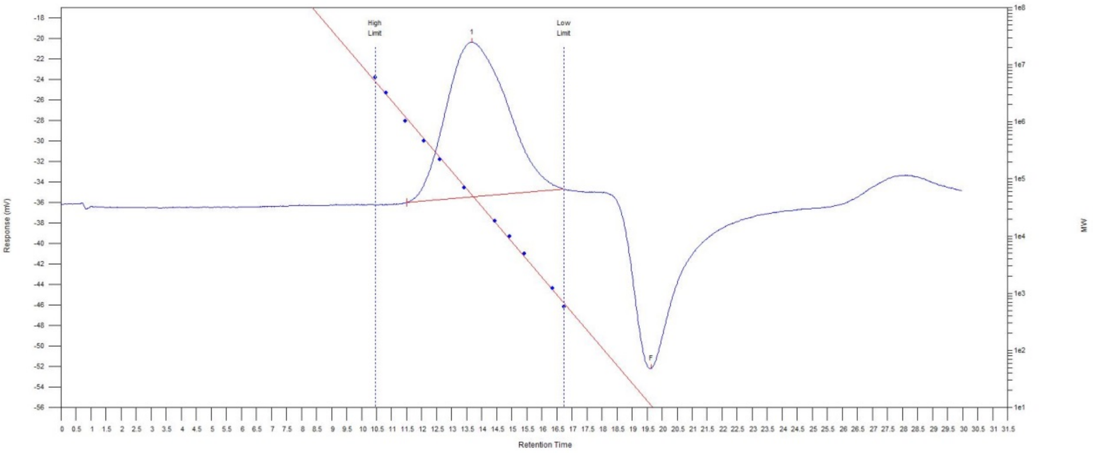

(b)

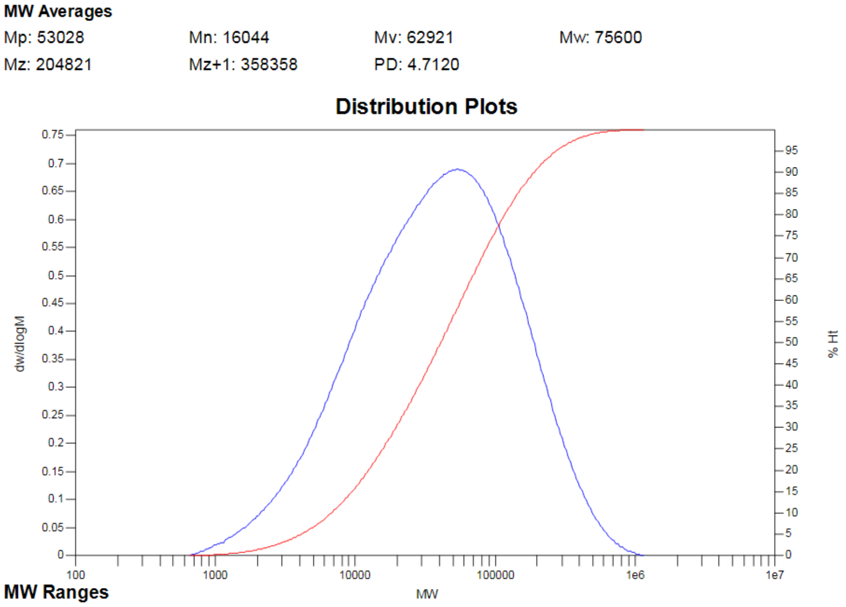

Supplementary Fig. 73. Gel Permeation Chromatography (GPC) trace of **Entry 4** for reproducibility experiment. (a) Trace data from refractive index (RI) detector. (b) Molecular weight distribution plots of **Entry 4** of reproducibility experiments **(Table S1, Entry 4)**.

(a)

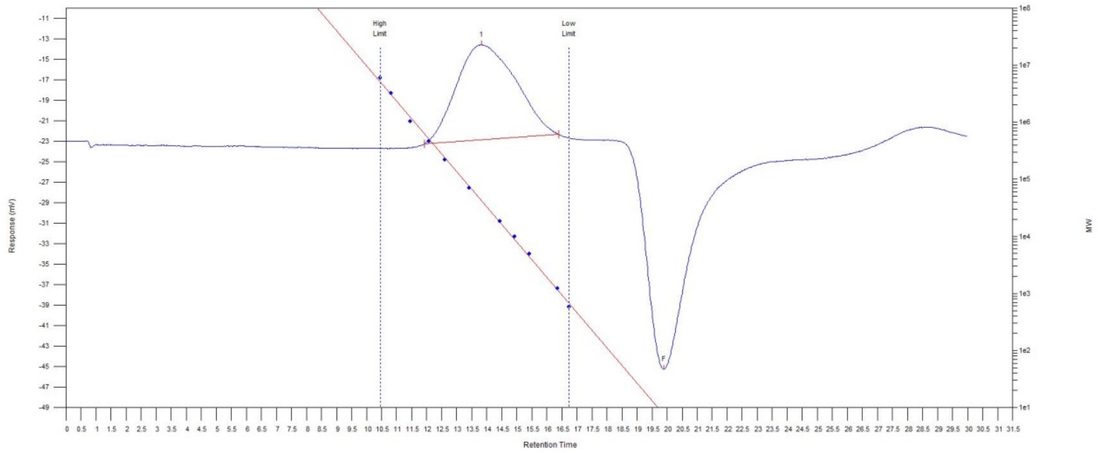

(b)

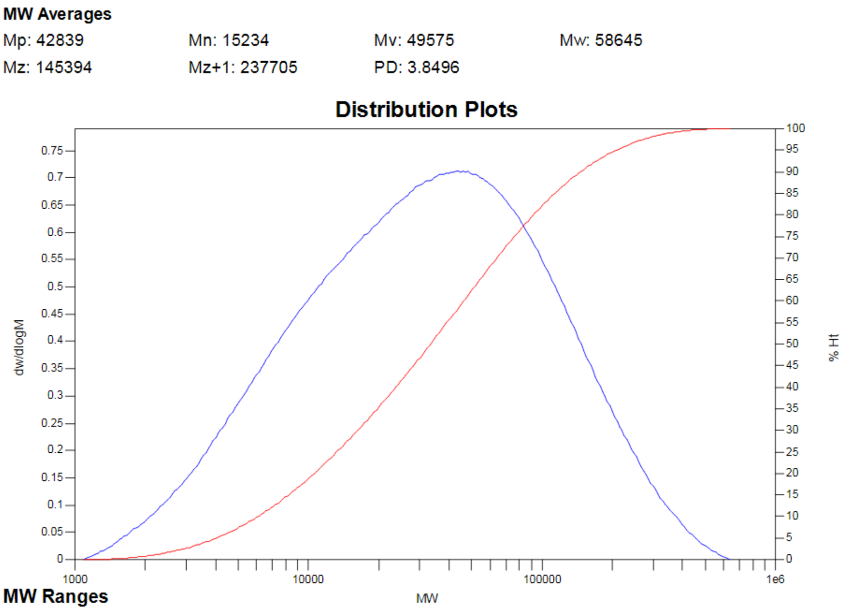

Supplementary Fig. 74. Gel Permeation Chromatography (GPC) trace of **Entry 5** for reproducibility experiment. (a) Trace data from refractive index (RI) detector. (b) Molecular weight distribution plots of **Entry 5** of reproducibility experiments **(Table S1, Entry 5)**.

Stille Coupling Polymerizations

(a)

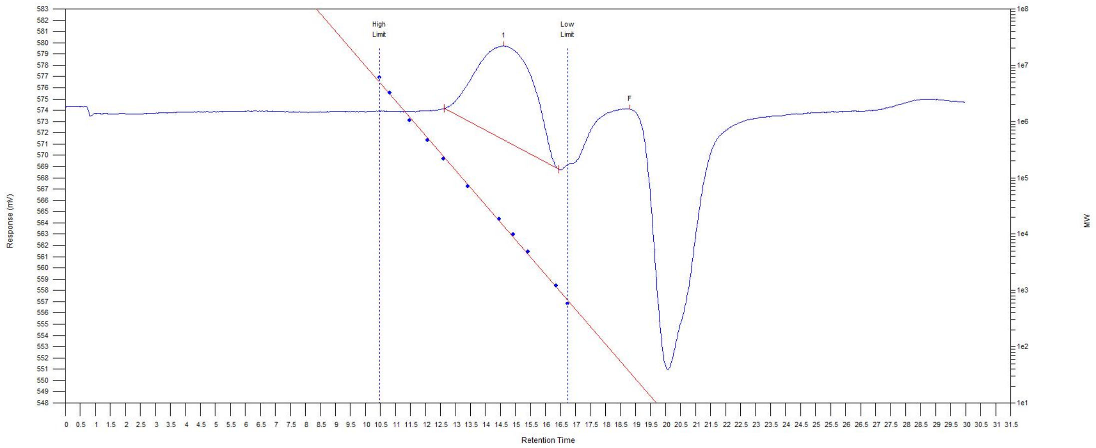

(b)

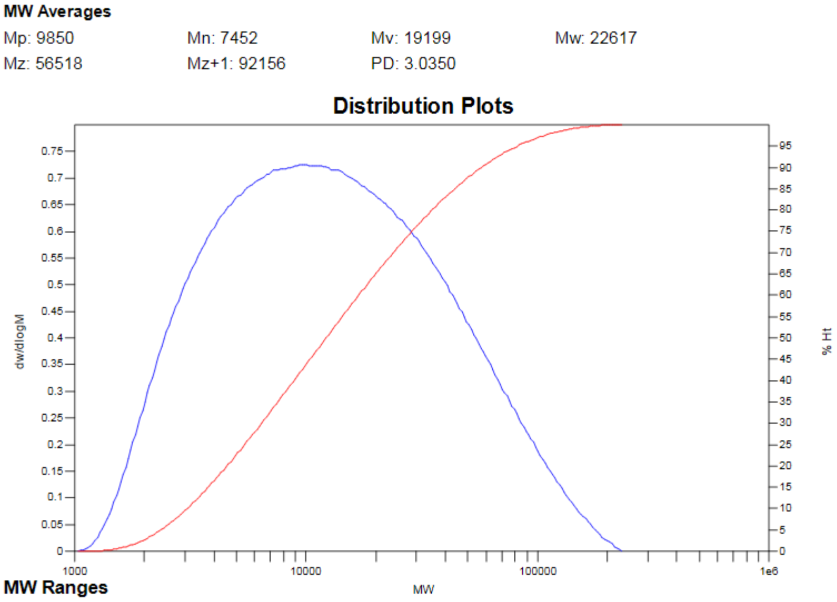

Supplementary Fig. 75. Gel Permeation Chromatography (GPC) trace of **Entry 3** by Stille coupling polymerization. (a) Trace data from refractive index (RI) detector. (b) Molecular weight distribution plots of **Entry 3** of Stille coupling polymerizations **(Table 2, Entry 3)**.

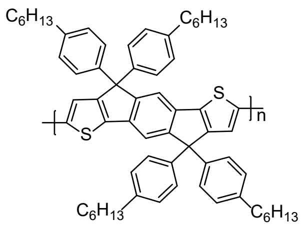

(a)

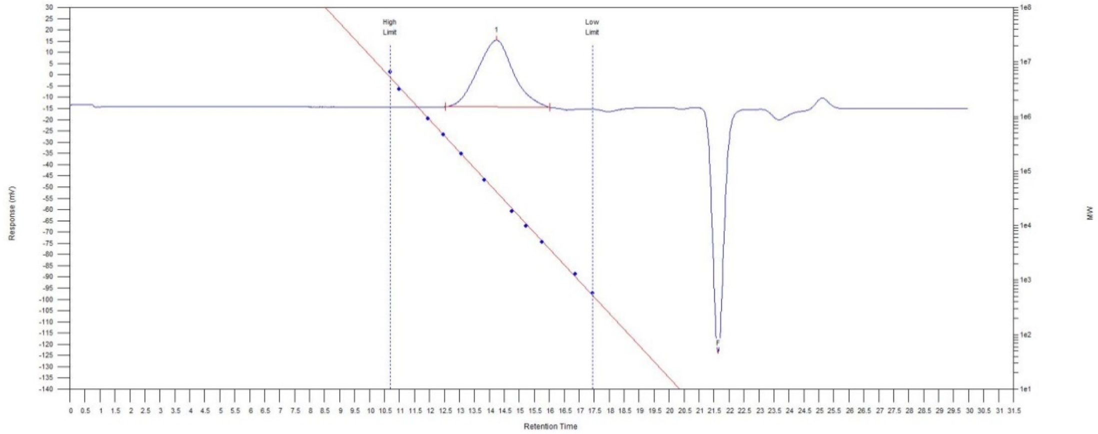

(b)

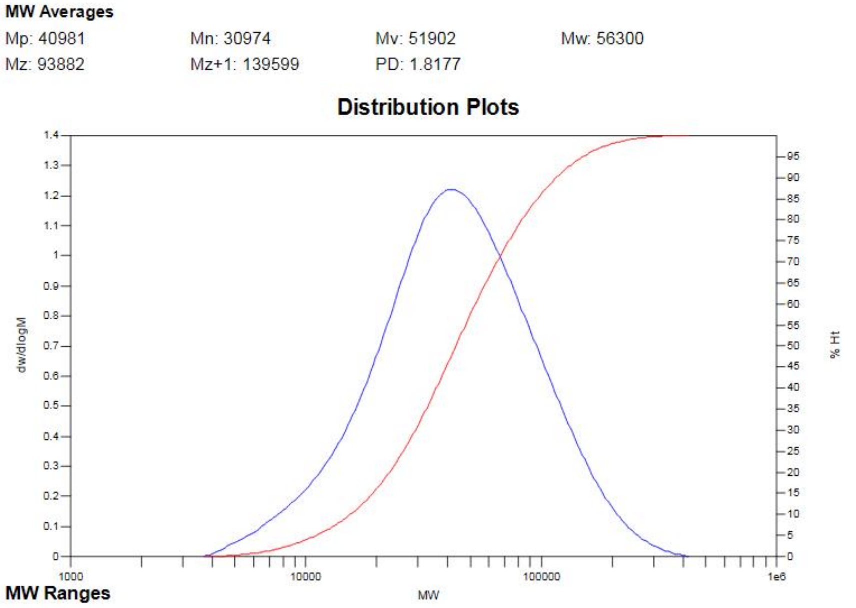

Supplementary Fig. 76. Gel Permeation Chromatography (GPC) trace of **PIDT**. (a) Trace data from refractive index (RI) detector. (b) Molecular weight distribution plots of **PIDT**.

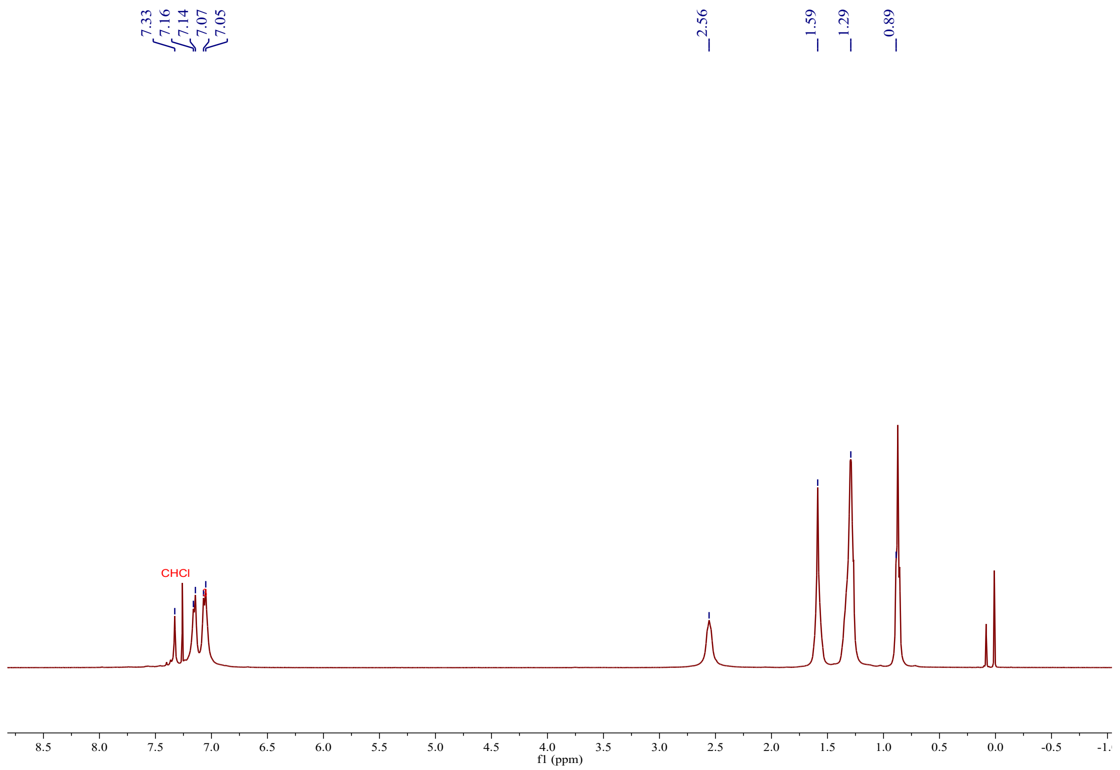

Supplementary Fig. 77. ^{1}H NMR spectrum of **PIDT** in $CDCl_3$ at 298 K.

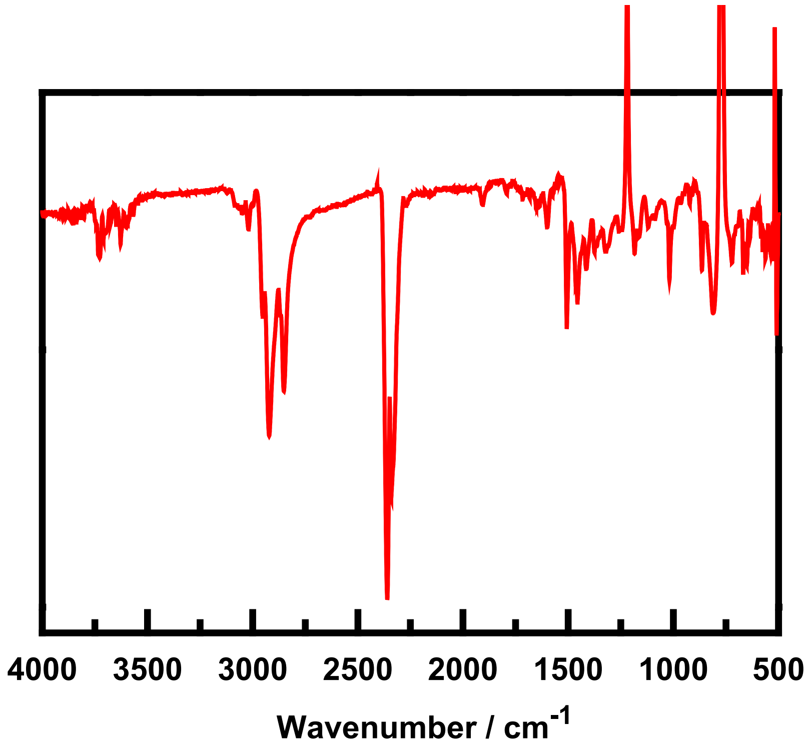

Supplementary Fig. 78. FT-IR spectrum of compound **PIDT** in thin film at 298 K.

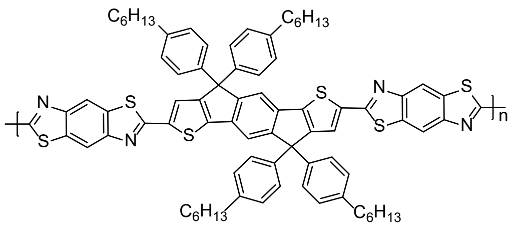

(a)

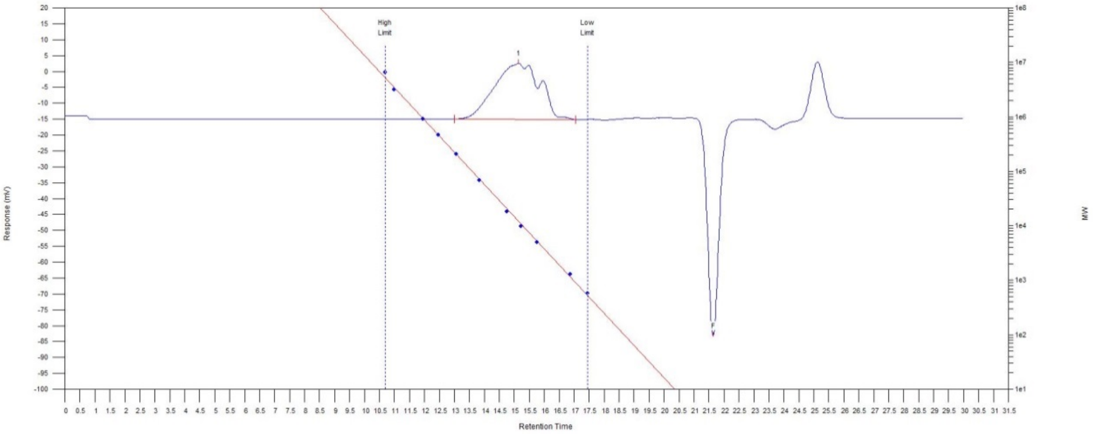

(b)

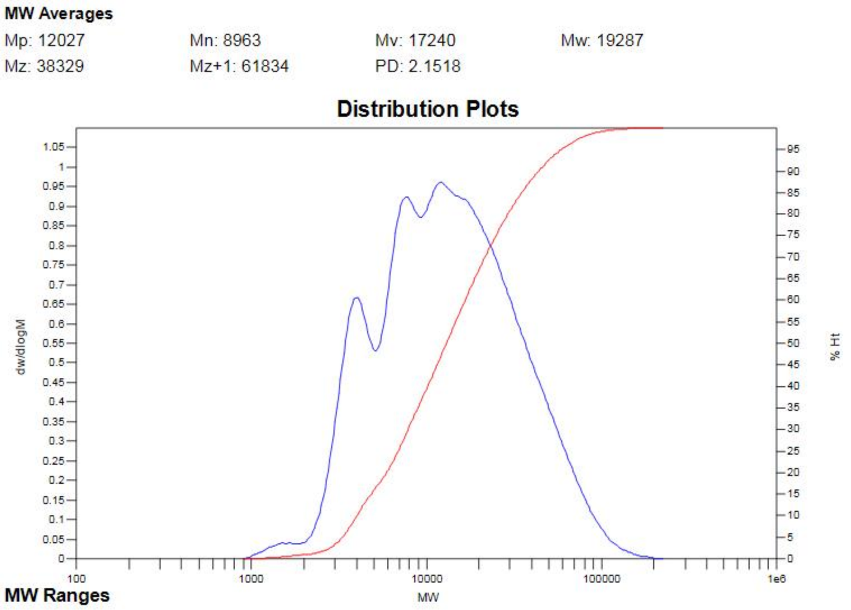

Supplementary Fig. 79. Gel Permeation Chromatography (GPC) trace of **PBBT**. (a) Trace data from refractive index (RI) detector. (b) Molecular weight distribution plots of **PBBT**.

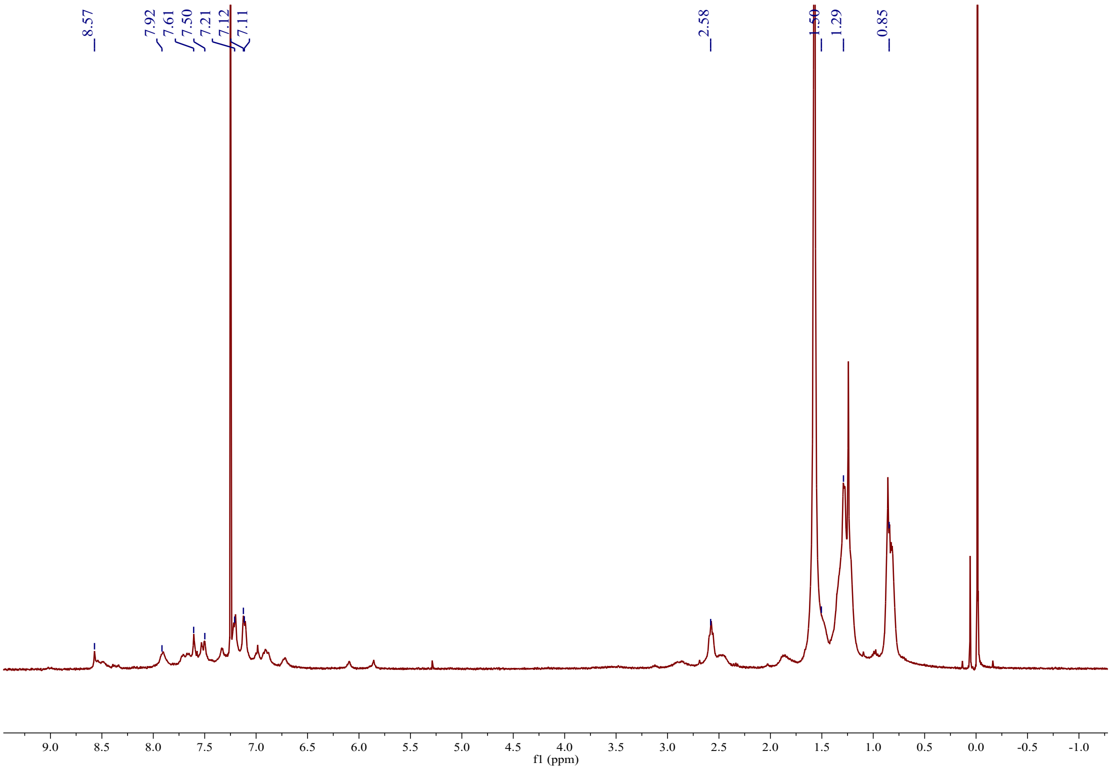

Supplementary Fig. 80. ^{1}H NMR spectrum of **PBBT** in $CDCl_3$ at 298 K.

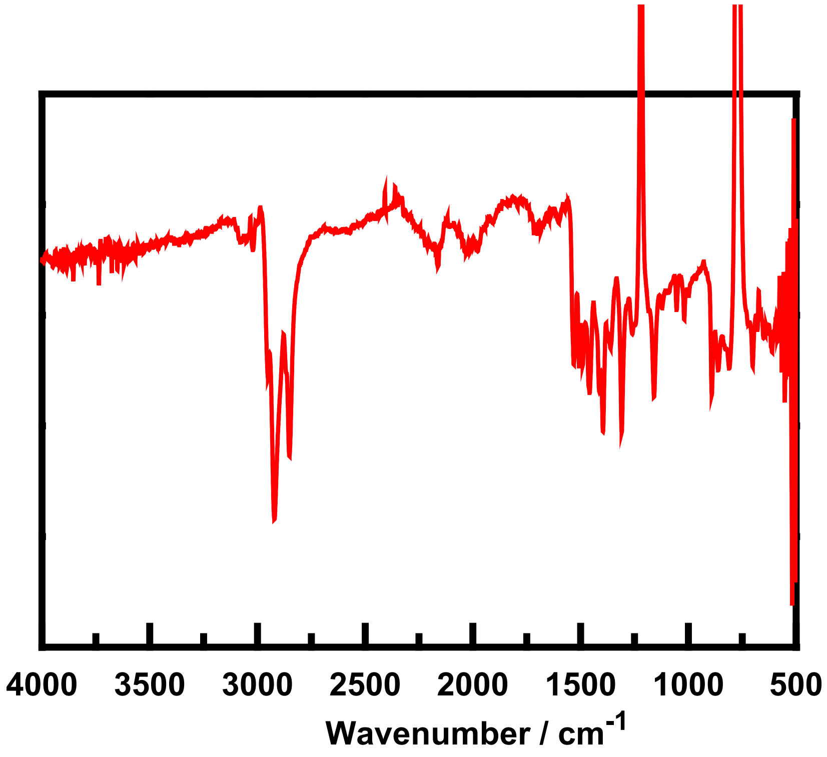

Supplementary Fig. 81. FT-IR spectrum of compound **PBBT** in thin film at 298 K.

(a)

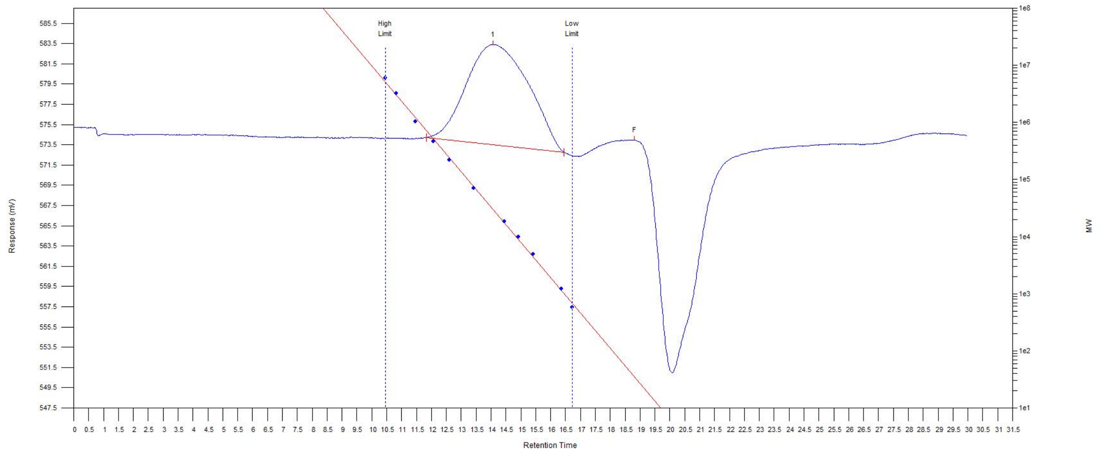

(b)

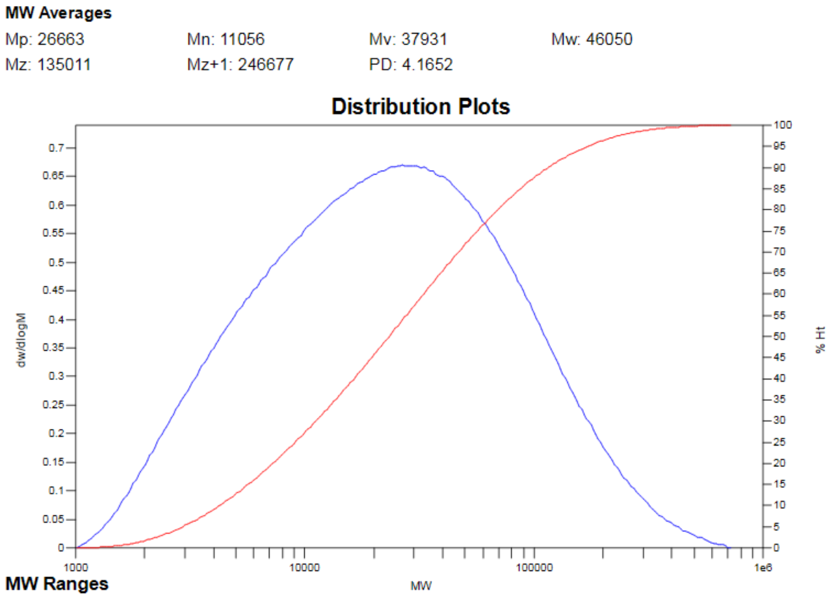

Supplementary Fig. 82. Gel Permeation Chromatography (GPC) trace of **P1-Cl** in **Entry 2** by Stille coupling polymerization. (a) Trace data from refractive index (RI) detector. (b) Molecular weight distribution plots of **Entry 2** of Stille coupling polymerizations **(Table 2, Entry 2)**.

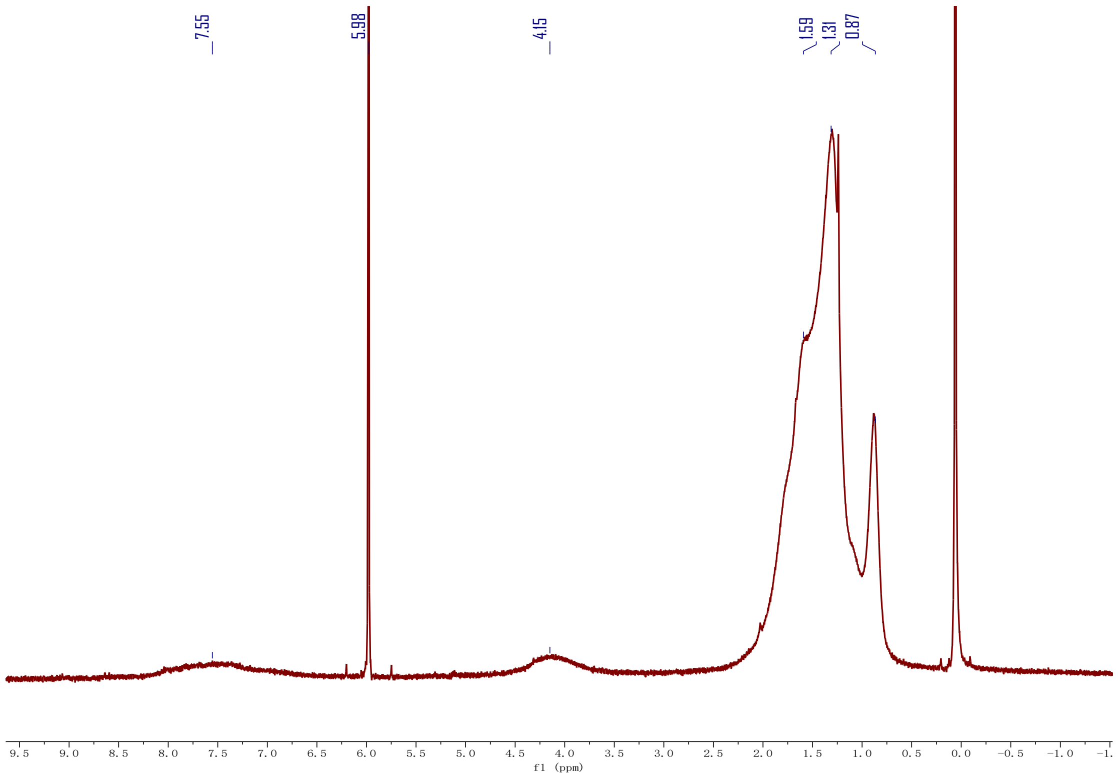

Supplementary Fig. 83. ^{1}H NMR spectrum of **P1-CI** in $C_2D_2Cl_4$ at 298 K.

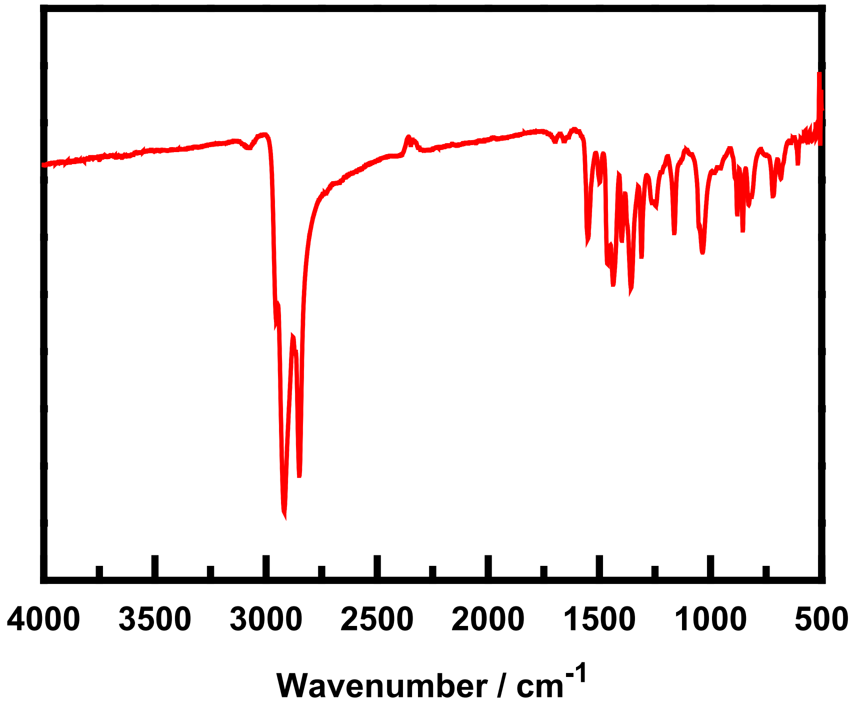

Supplementary Fig. 84. FT-IR spectrum of compound **P1-CI** in thin film at 298 K.

(a)

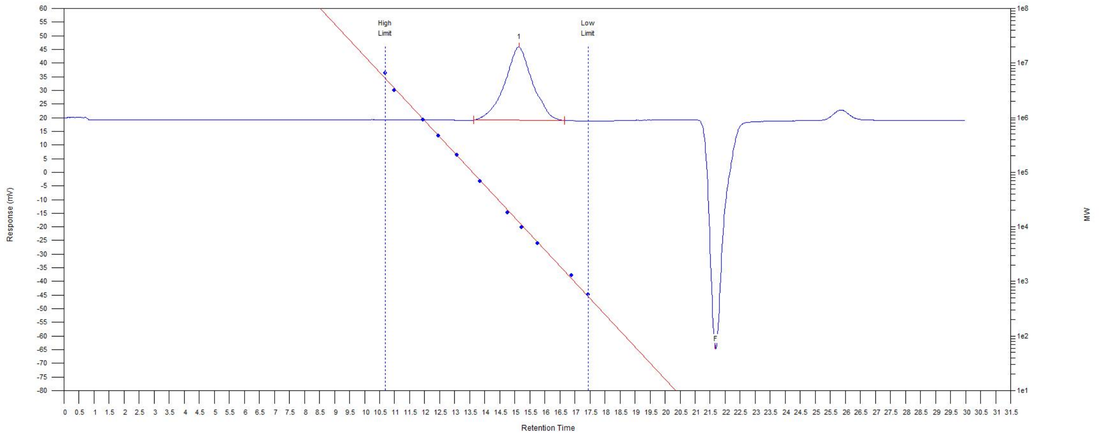

(b)

MW Averages

Mp: 12303	Mn: 10165	Mv: 14314	Mw: 15201
Mz: 22851	Mz+1: 33044	PD: 1.4954	

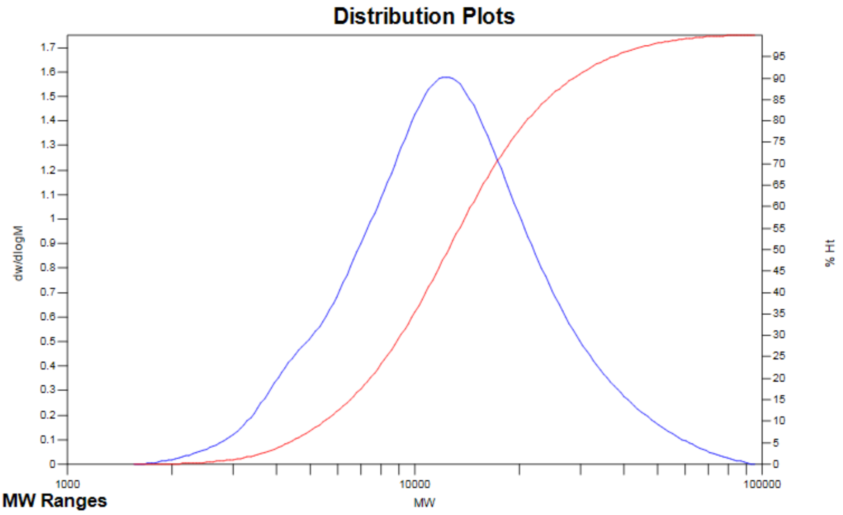

Supplementary Fig. 85. Gel Permeation Chromatography (GPC) trace of **P2-Cl** by Stille coupling polymerization. (a) Trace data from refractive index (RI) detector. (b) Molecular weight distribution plots of **P2-Cl** of Stille coupling polymerizations.

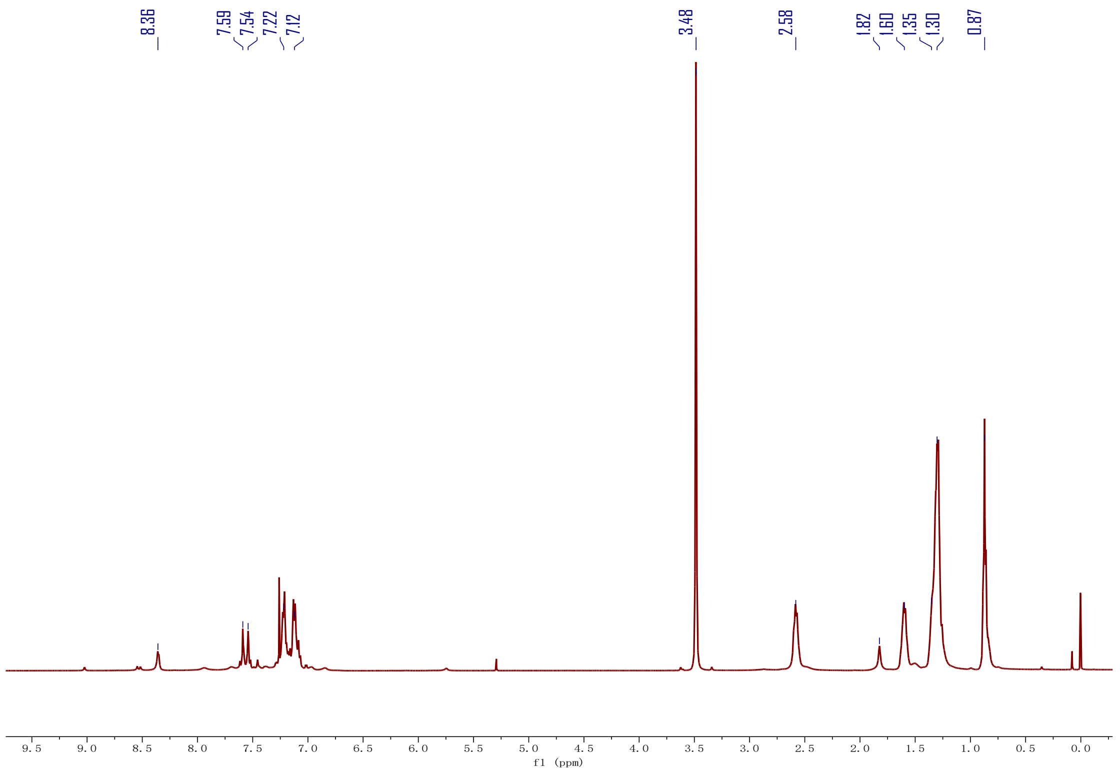

Supplementary Fig. 86. ^{1}H NMR spectrum of **P2-CI** in $CDCl_3$ at 298 K.

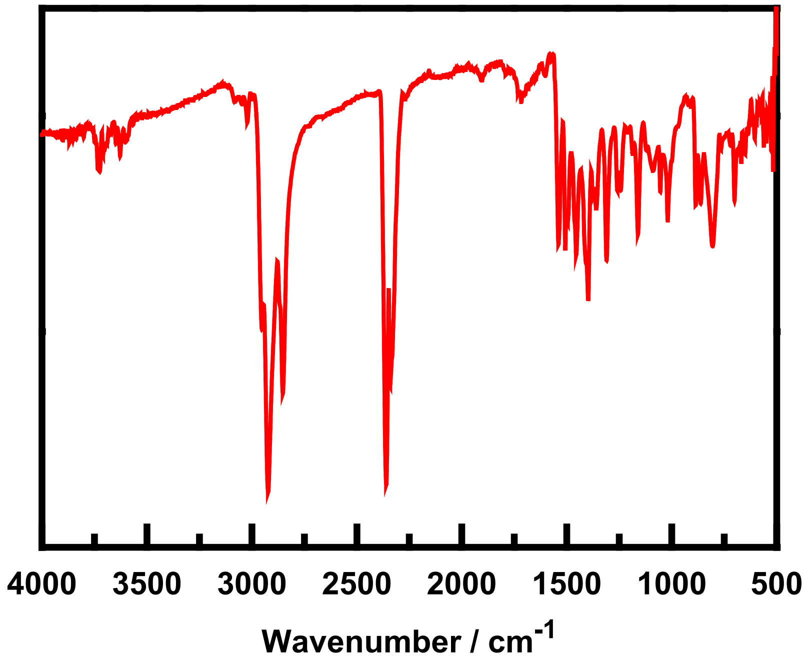

Supplementary Fig. 87. FT-IR spectrum of compound **P2-CI** in thin film at 298 K.

Polymer Characterization Data

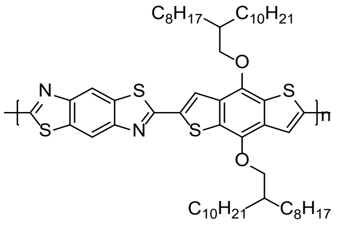

(a)

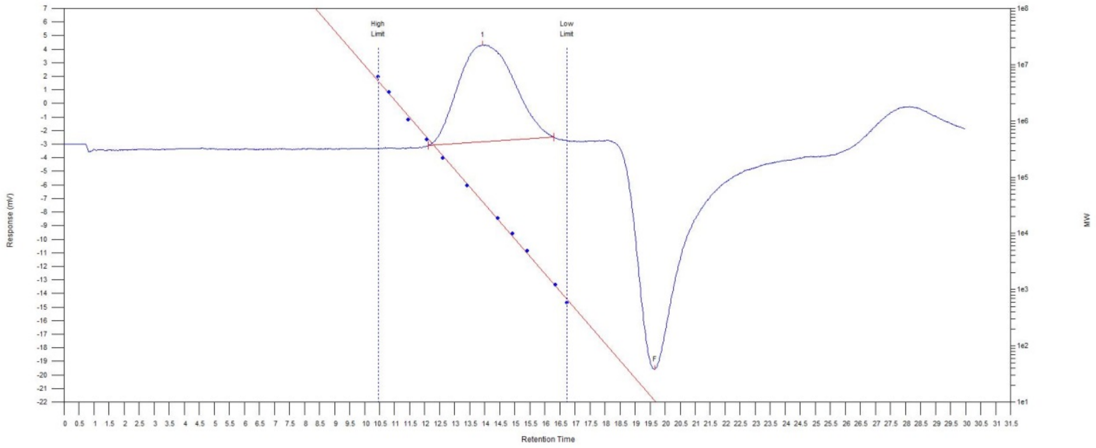

(b)

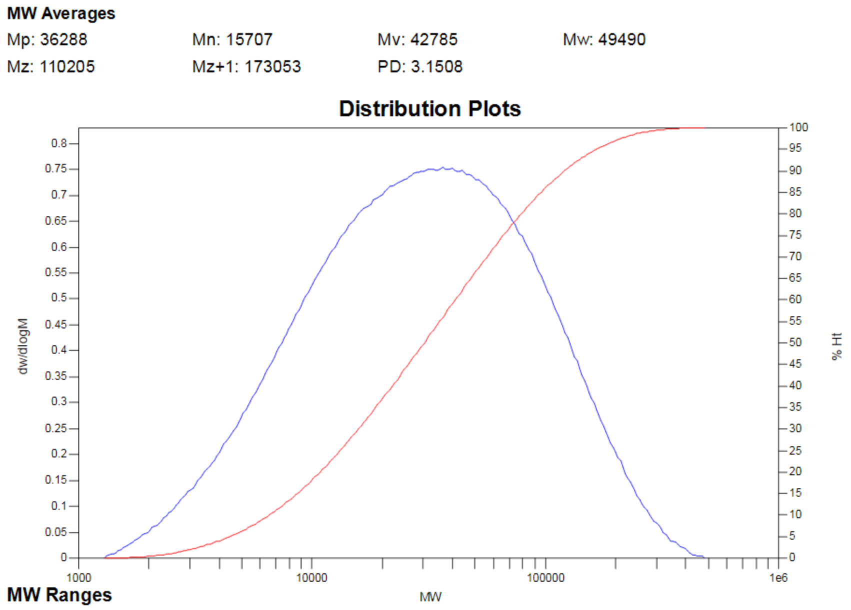

Supplementary Fig. 88. Gel Permeation Chromatography (GPC) trace of **P1 (P1-CS)**. (a) Trace data from refractive index (RI) detector. (b) Molecular weight distribution plots of **P1 (Table 3, Entry 1)**.

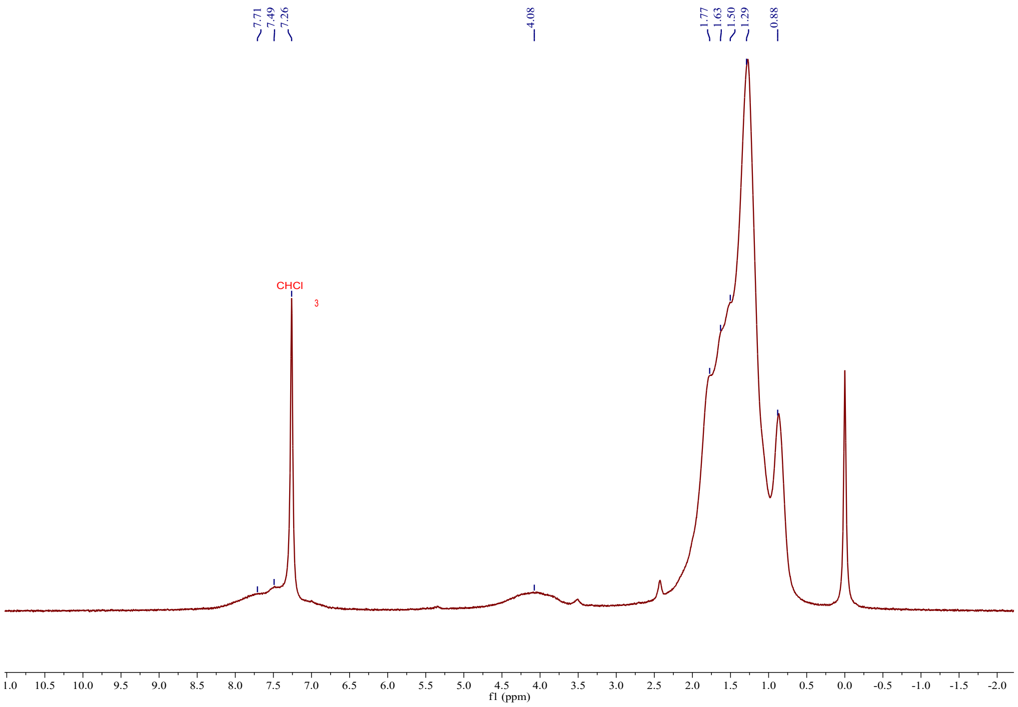

Supplementary Fig. 89. ^{1}H NMR spectrum of **P1 (P1-CS)** in $CDCl_3$ at 298 K.

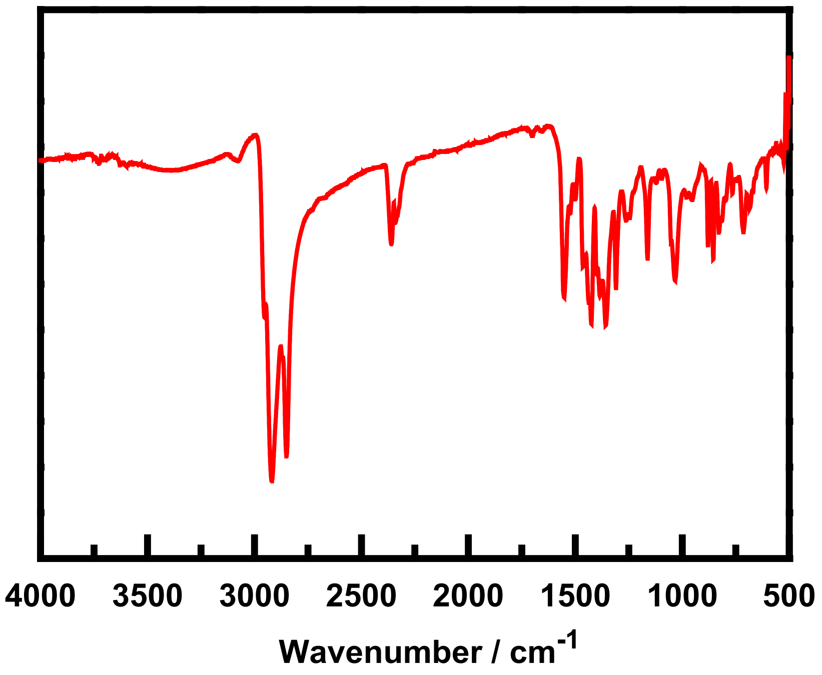

Supplementary Fig. 90. FT-IR spectrum of compound **P1 (P1-CS)** in thin film at 298 K.

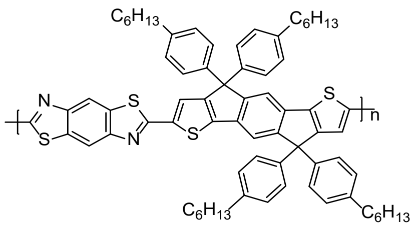

(a)

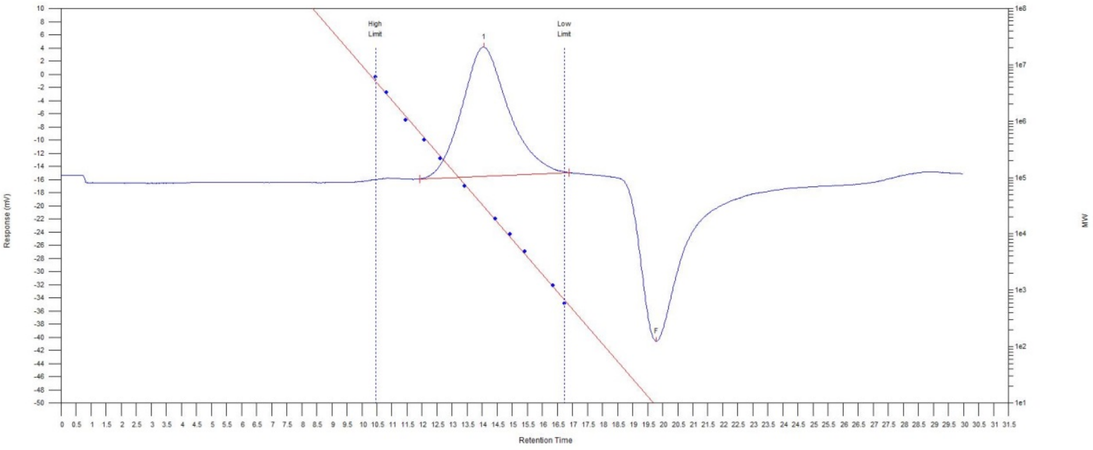

(b)

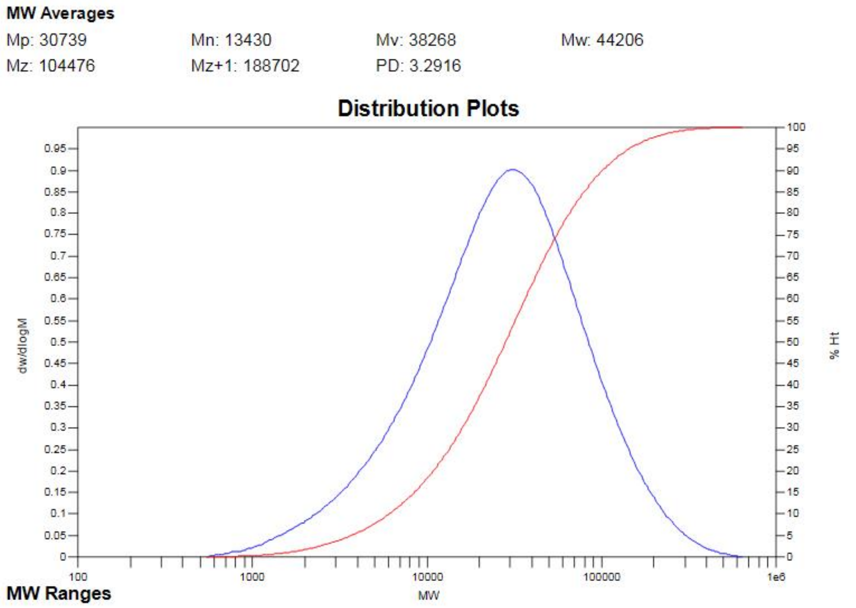

Supplementary Fig. 91. Gel Permeation Chromatography (GPC) trace of **P2 (P2-CS)**. (a) Trace data from refractive index (RI) detector. (b) Molecular weight distribution plots of **P2 (P2-CS) (Table 3, Entry 2)**.

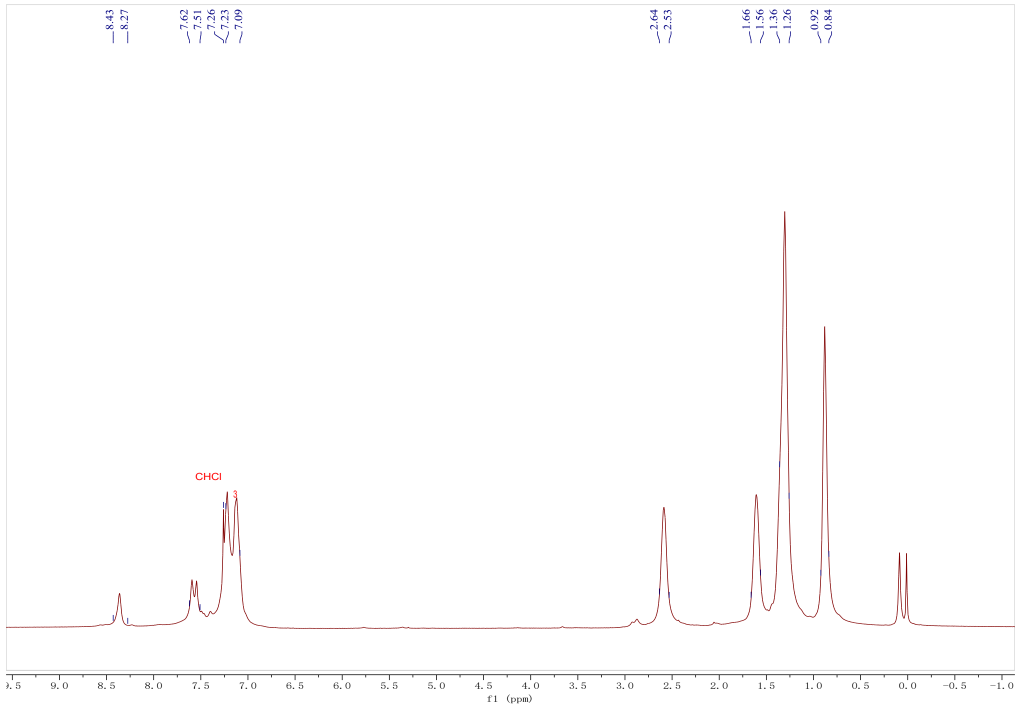

Supplementary Fig. 92. ^{1}H NMR spectrum of **P2 (P2-CS)** in $CDCl_3$ at 298 K.

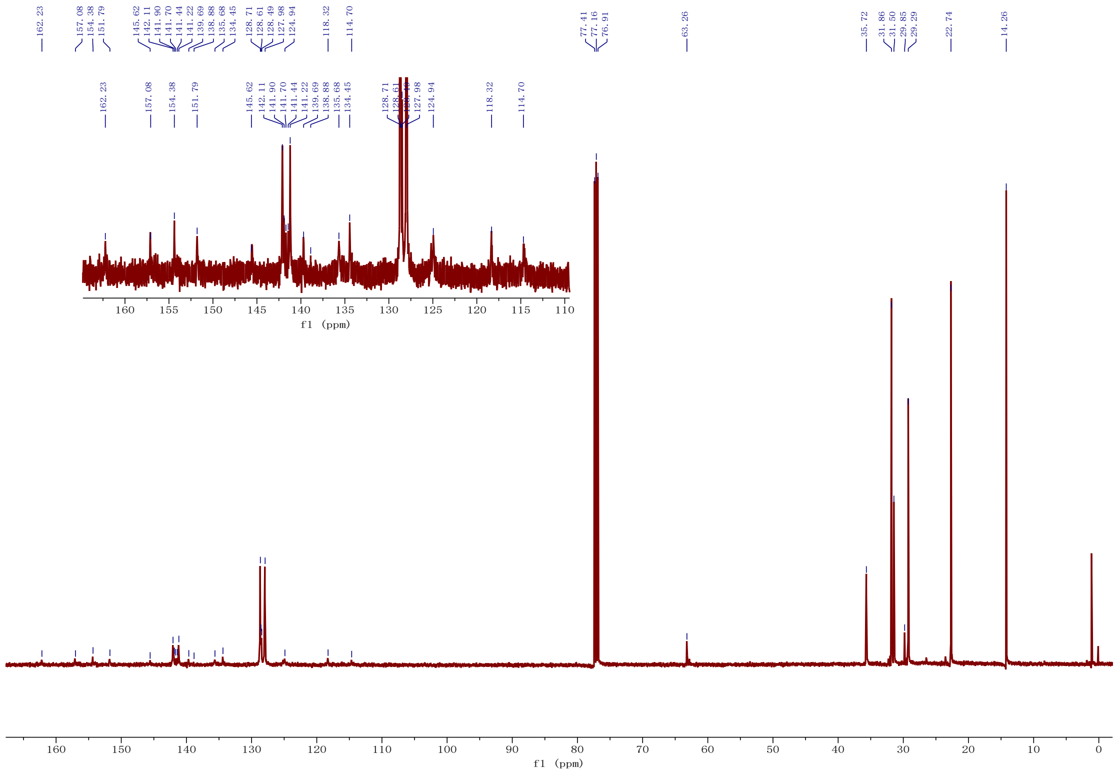

Supplementary Fig. 93. ^{1}H NMR spectrum of **P2 (P2-CS)** in $CDCl_3$ at 298 K.

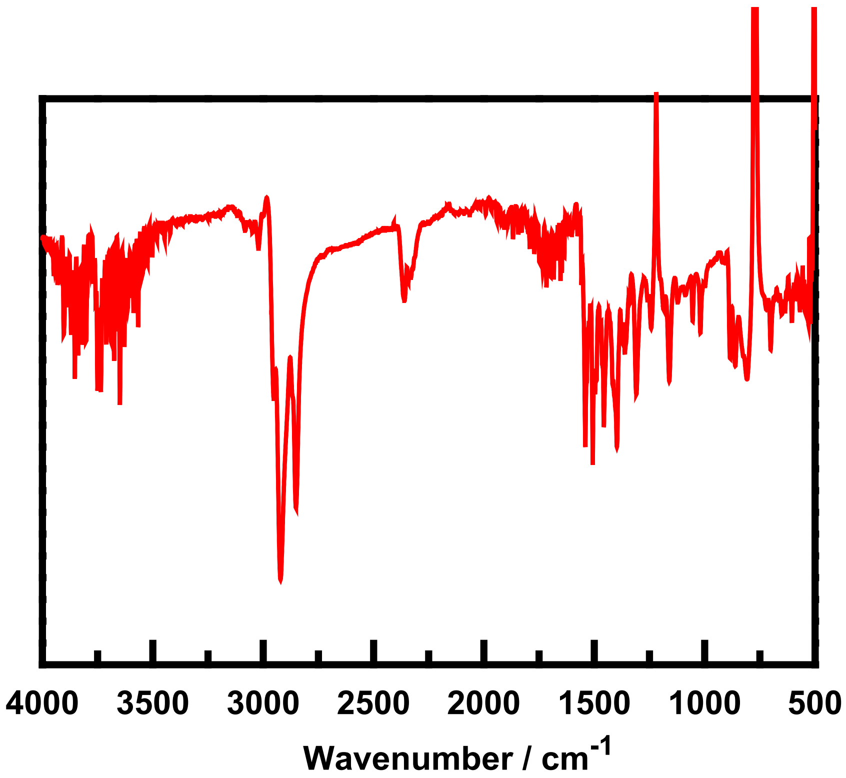

Supplementary Fig. 94. FT-IR spectrum of compound **P2 (P2-CS)** in thin film at 298 K.

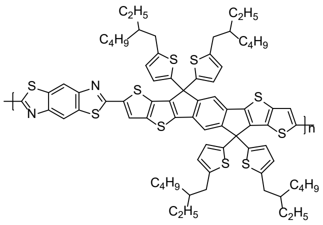

Chloroform fraction:

(a)

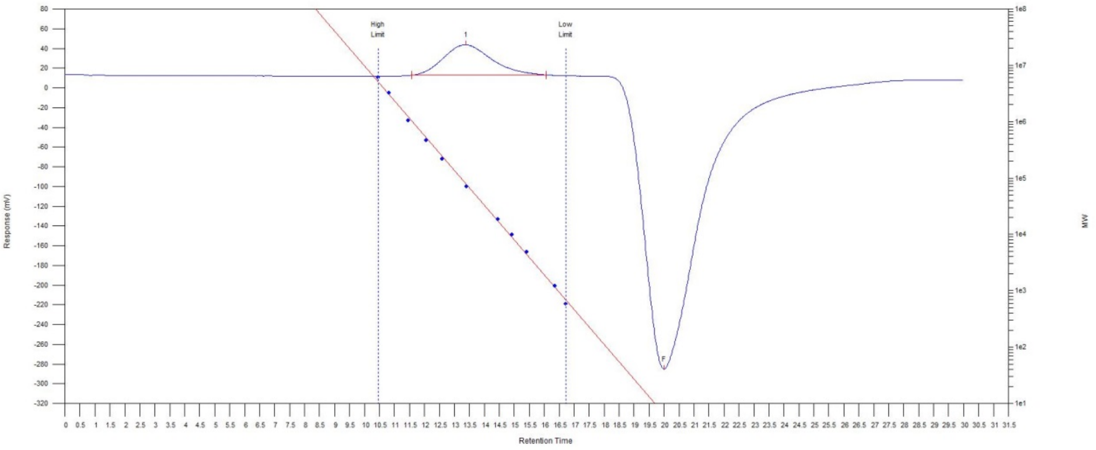

(b)

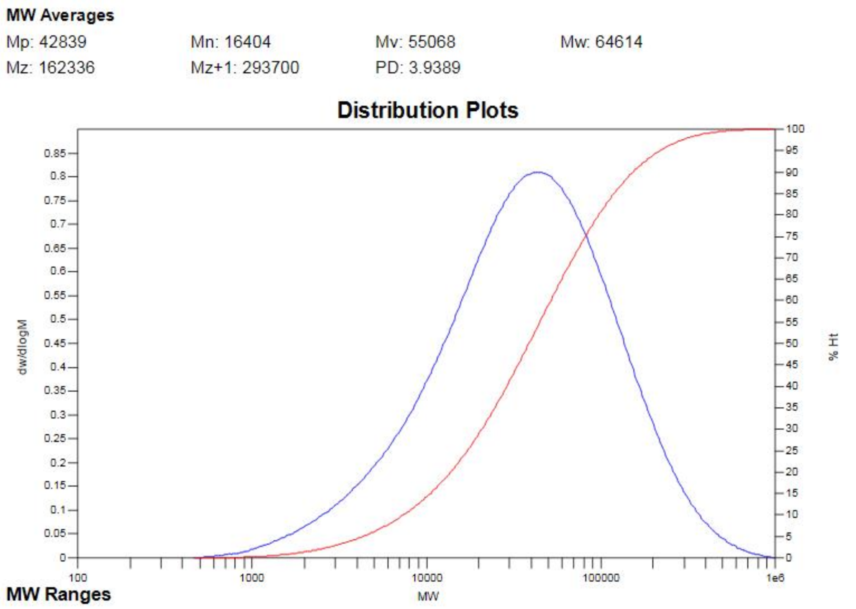

Supplementary Fig. 95. Gel Permeation Chromatography (GPC) trace of **P3** chloroform fraction. (a) Trace data from refractive index (RI) detector. (b) Molecular weight distribution plots of **P3** chloroform fraction **(Table 3, Entry 3)**.

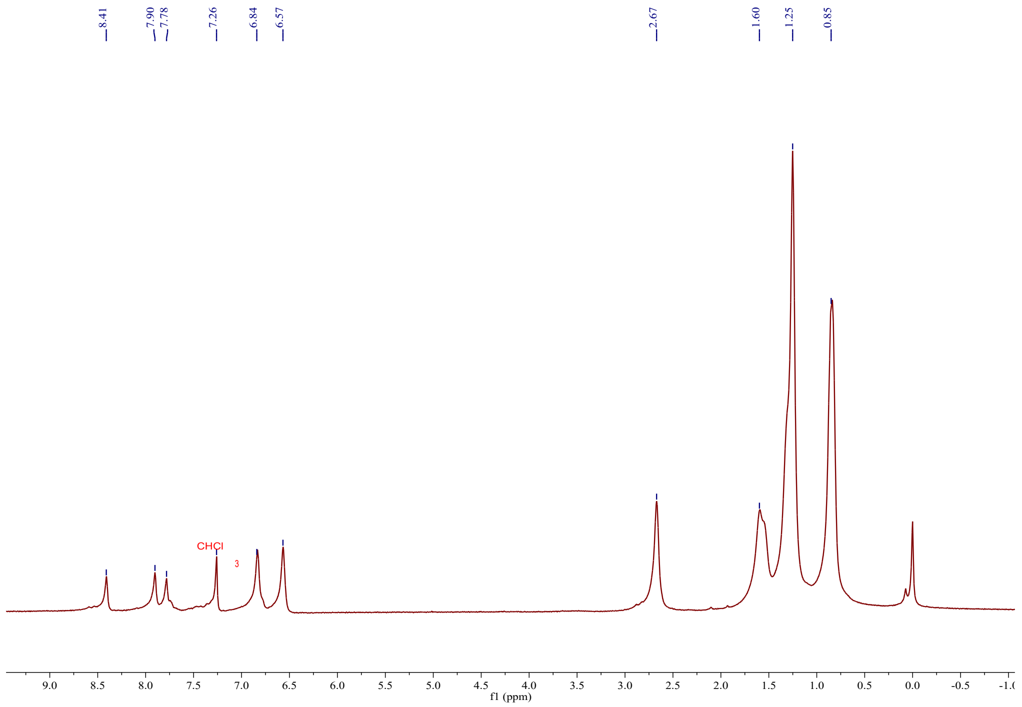

Supplementary Fig. 96. ^{1}H NMR spectrum of **P3** chloroform fraction in $CDCl_3$ at 298 K.

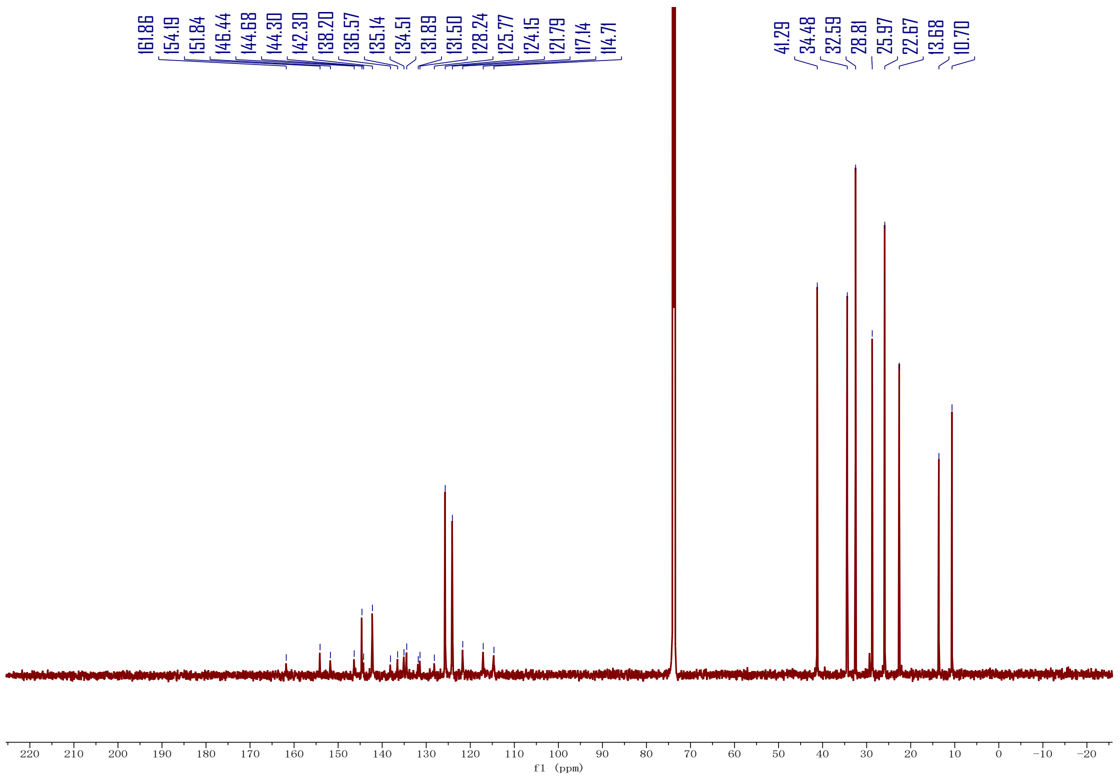

Supplementary Fig. 97. ^{1}H NMR spectrum of **P3** in $CDCl_3$ at 298 K.

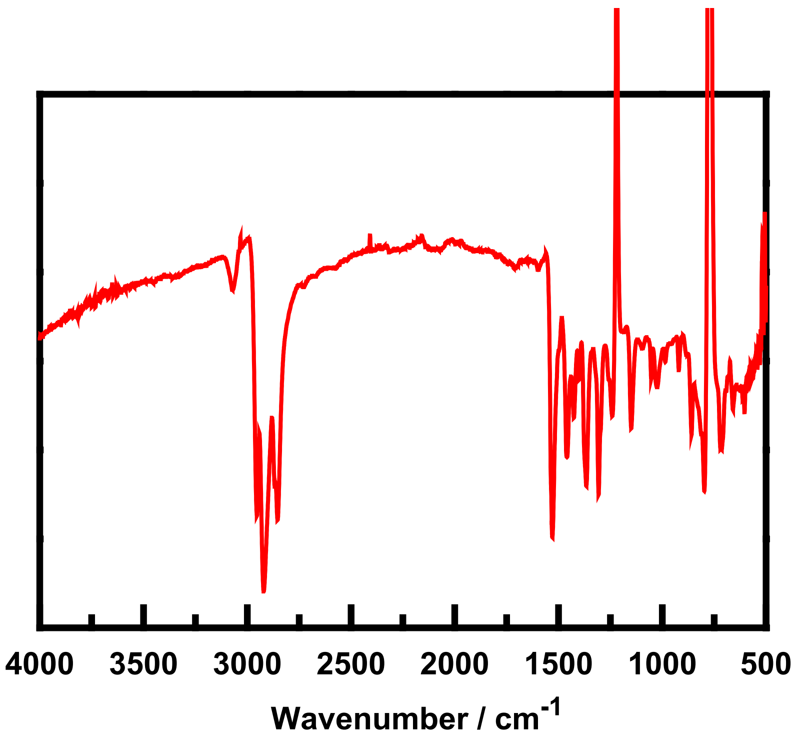

Supplementary Fig. 98. FT-IR spectrum of compound **P3** in thin film at 298 K

Chlorobenzene fraction

(a)

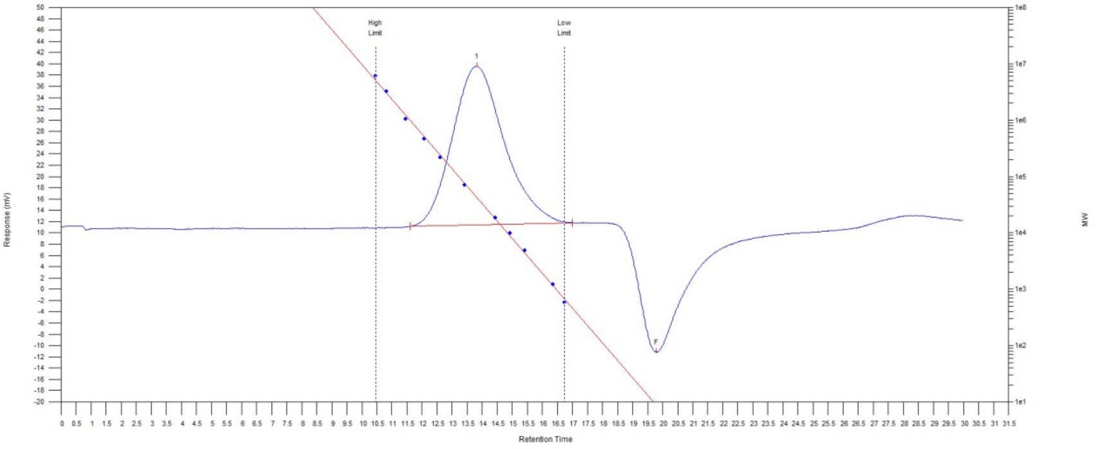

(b)

MW Averages

Mp: 79351 Mn: 34701 Mv: 95522 Mw: 109152

Mz: 232948 Mz+1: 374250 PD: 3.1455

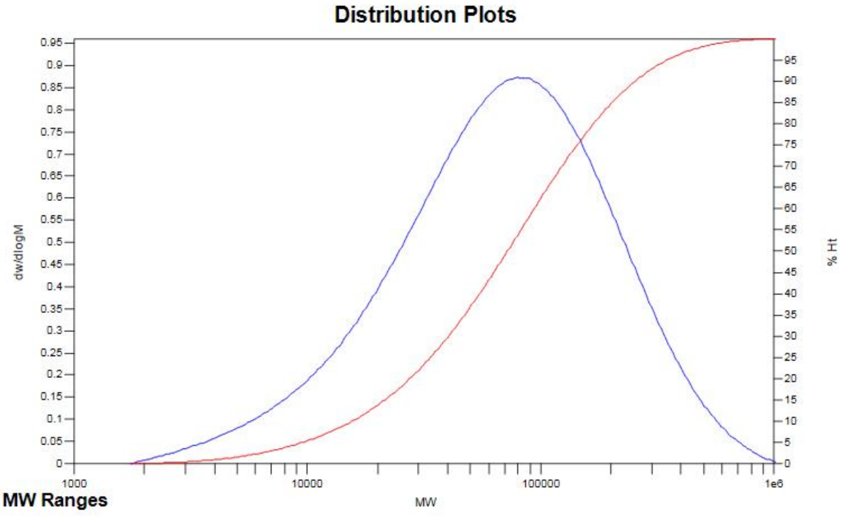

Supplementary Fig. 99. Gel Permeation Chromatography (GPC) trace of **P3** chloroform fraction. (a) Trace data from refractive index (RI) detector. (b) Molecular weight distribution plots of **P3** chlorobenzene fraction **(Table 3, Entry 3)**.

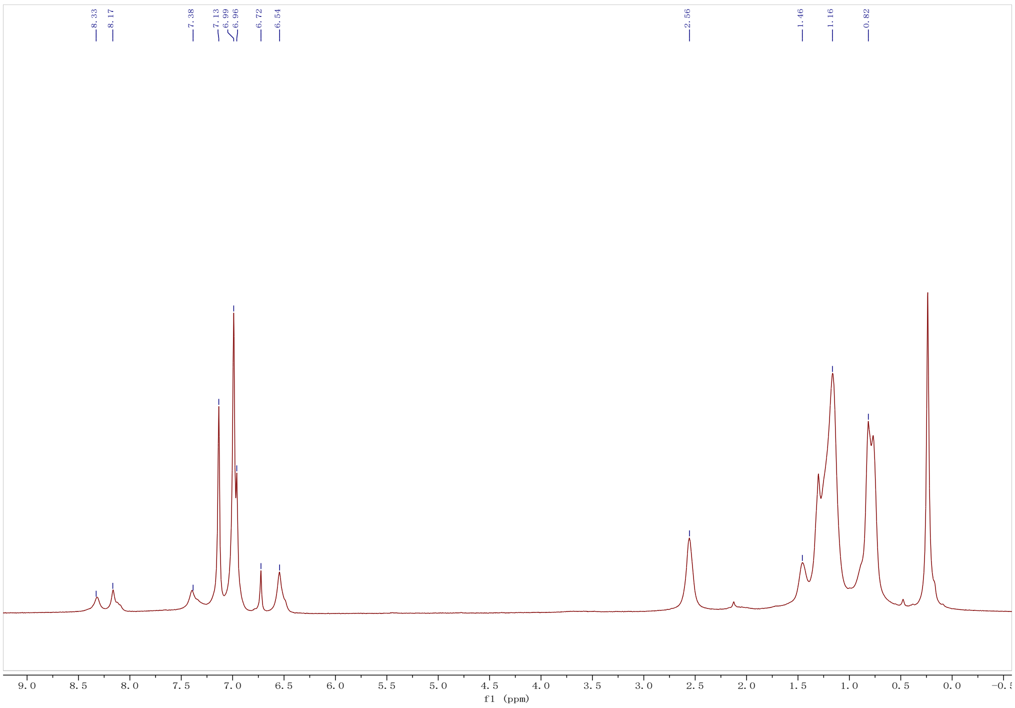

Supplementary Fig. 100. ^{1}H NMR spectrum of **P3** chlorobenzene fraction in C_6D_5Cl at 298 K.

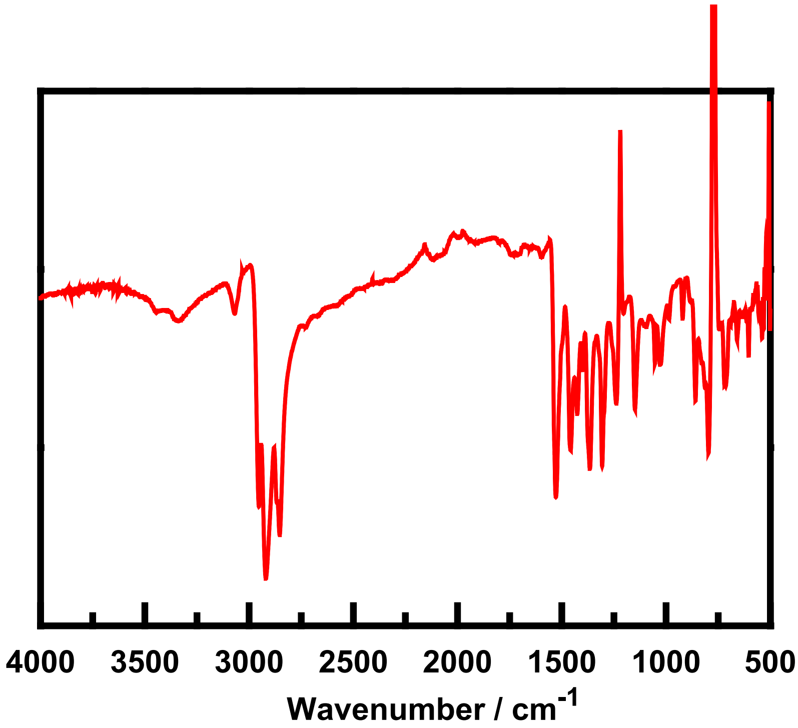

Supplementary Fig. 101. FT-IR spectrum of compound **P3** chlorobenzene fraction in thin film at 298 K.

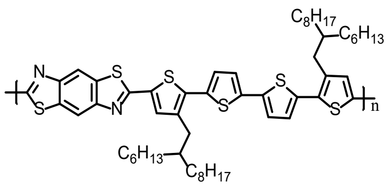

(a)

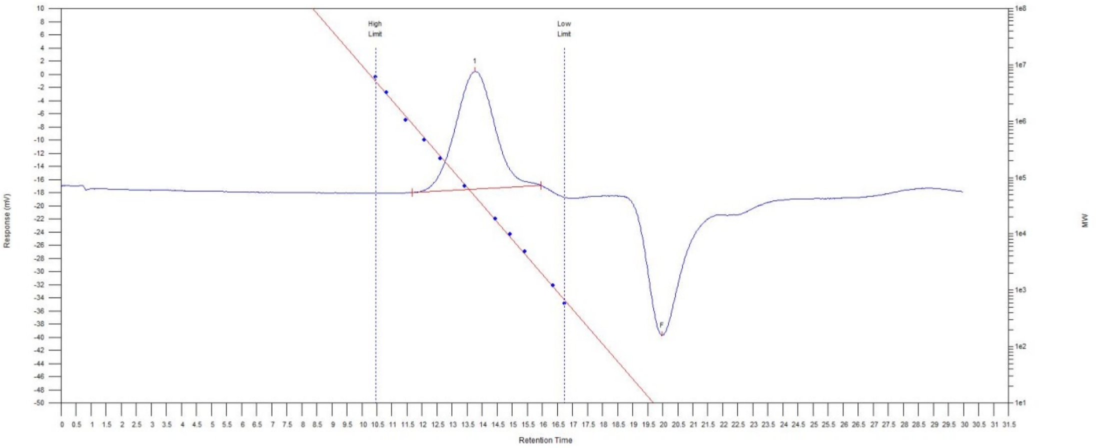

(b)

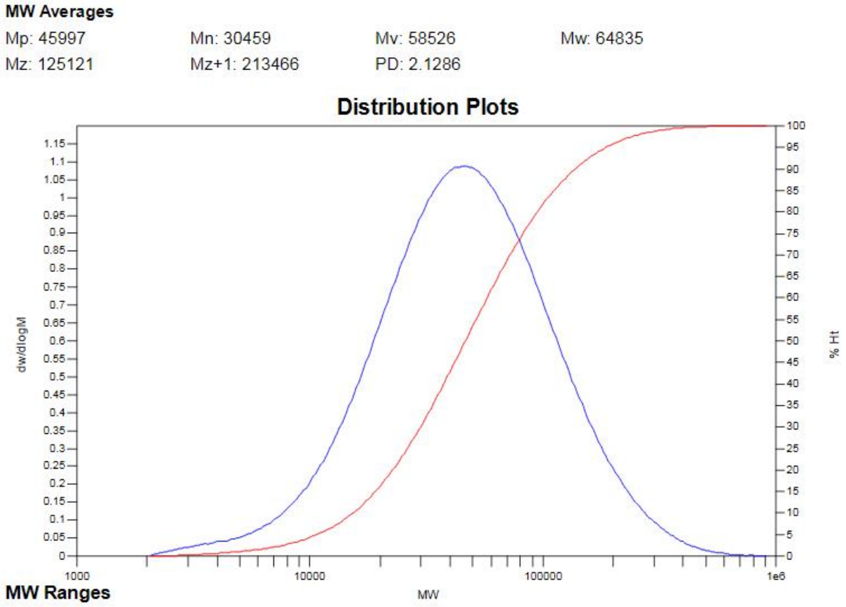

Supplementary Fig. 102. Gel Permeation Chromatography (GPC) trace of **P4**. (a) Trace data from refractive index (RI) detector. (b) Molecular weight distribution plots of **P4 (Table 3, Entry 4)**.

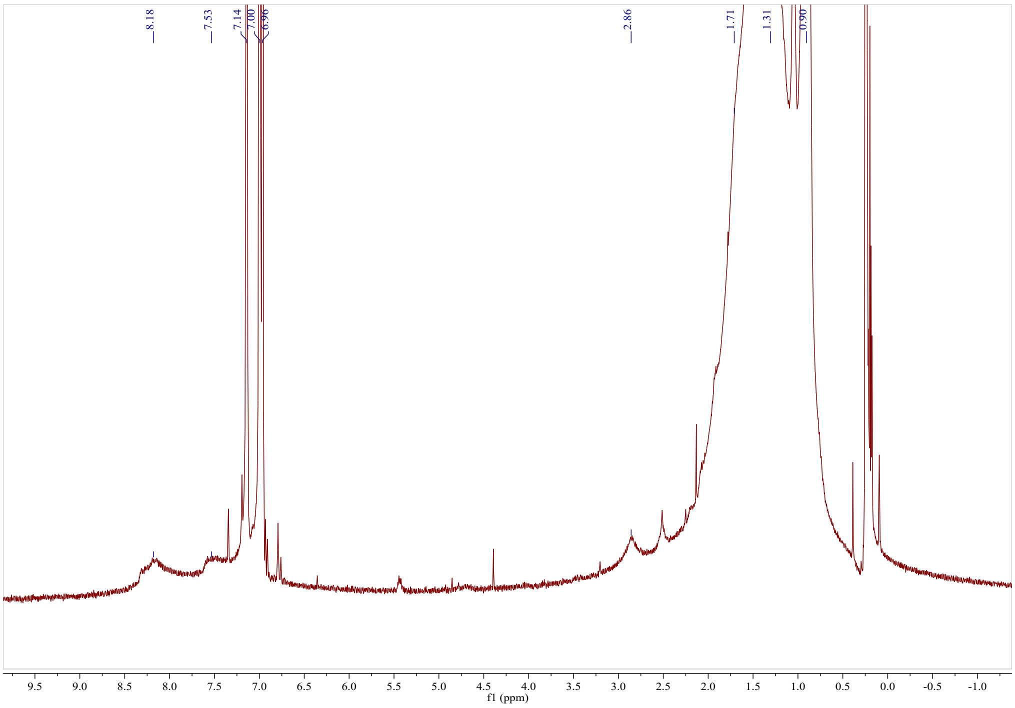

Supplementary Fig. 103. ^{1}H NMR spectrum of **P4** in C_6D_5Cl at 298 K.

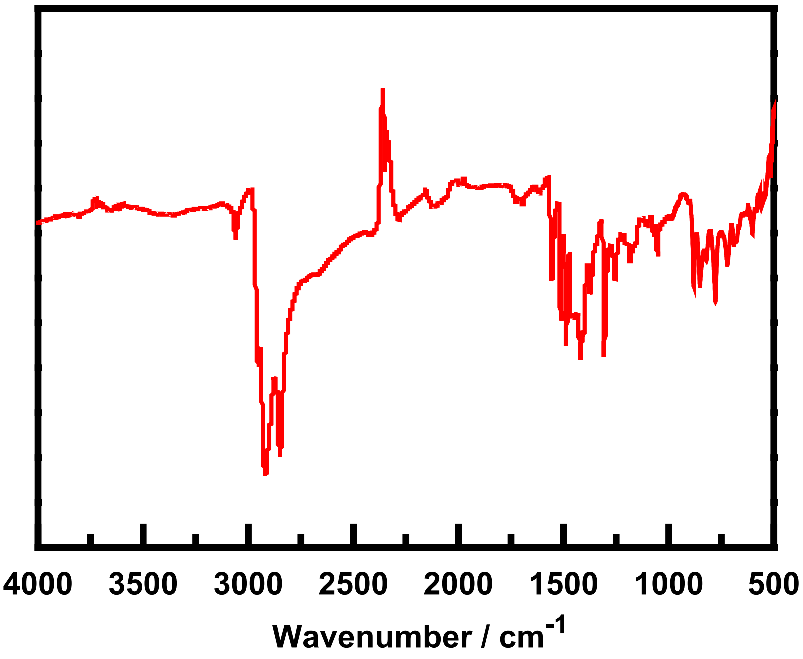

Supplementary Fig. 104. FT-IR spectrum of compound **P4** in thin film at 298 K.

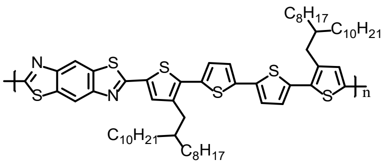

Chloroform fraction:

(a)

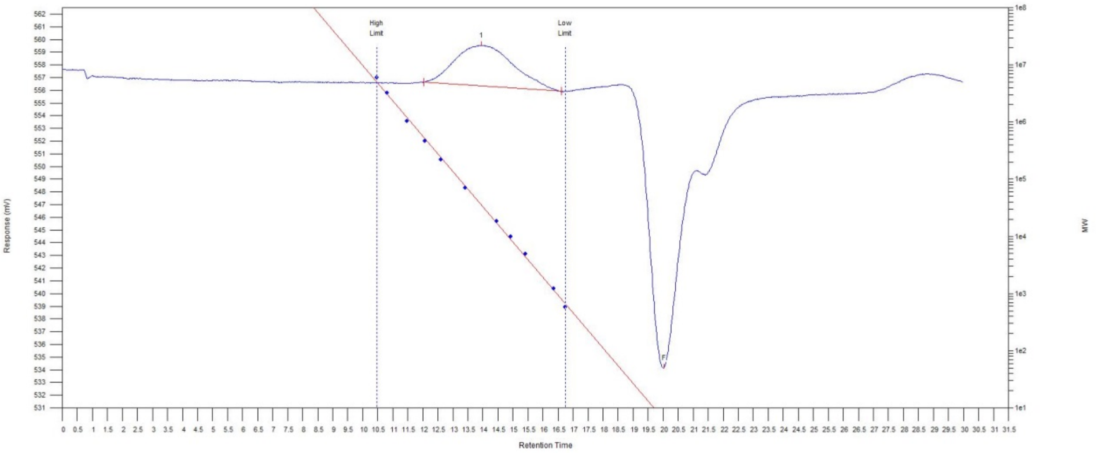

(b)

MW Averages

Mp: 35438	Mn: 13135	Mv: 44180	Mw: 52181
Mz: 127835	Mz+1: 207227	PD: 3.9727	

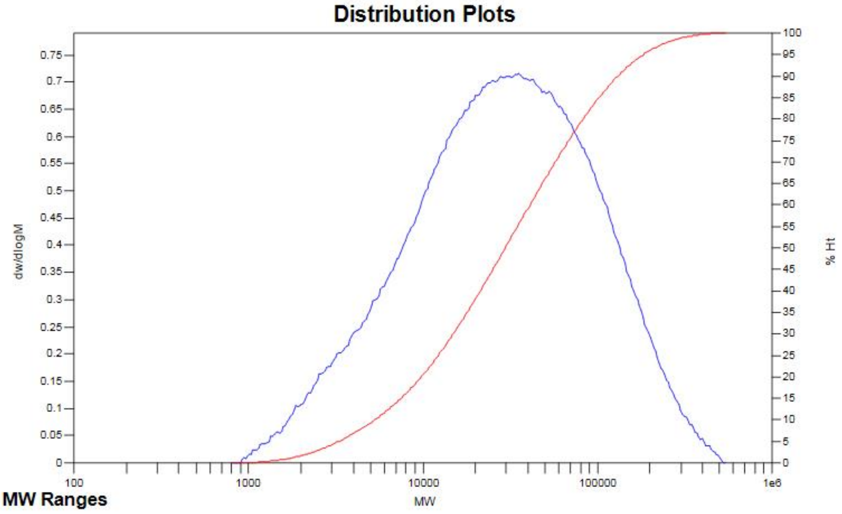

Supplementary Fig. 105. Gel Permeation Chromatography (GPC) trace of **P5** chloroform fraction. (a) Trace data from refractive index (RI) detector. (b) Molecular weight distribution plots of **P5** chloroform fraction **(Table 3, Entry 4)**.

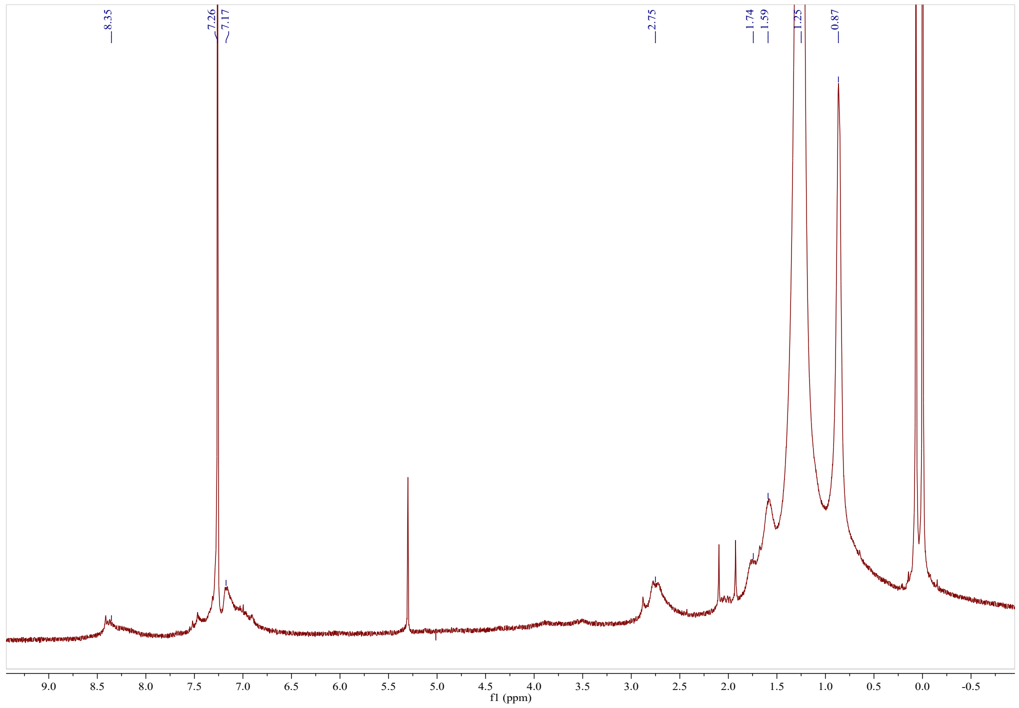

Supplementary Fig. 106. ^{1}H NMR spectrum of **P5** chloroform fraction in $CDCl_3$ at 298 K

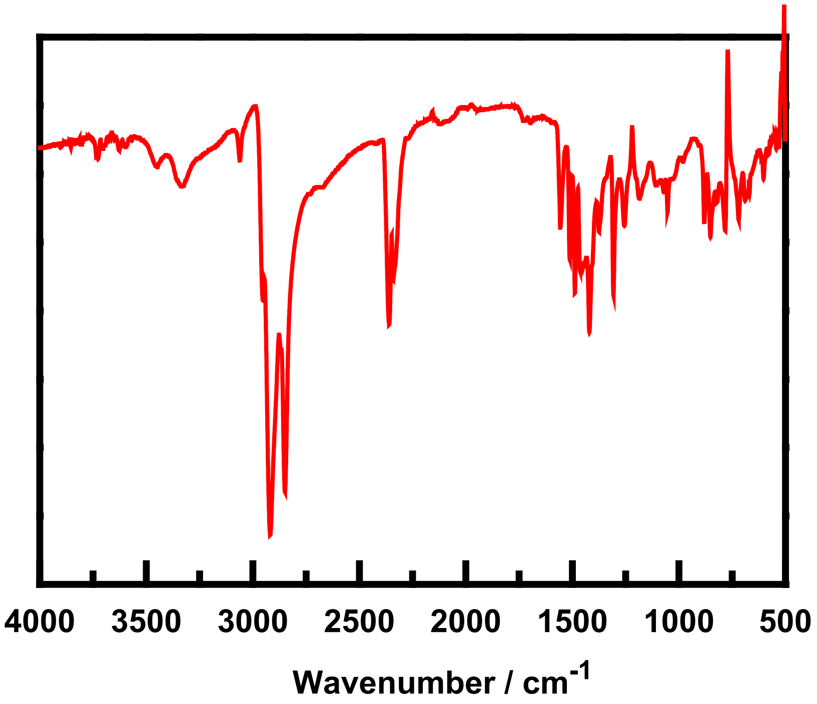

Supplementary Fig. 107. FT-IR spectrum of compound **P5** chloroform fraction in thin film at 298 K.

Chlorobenzene fraction:

(a)

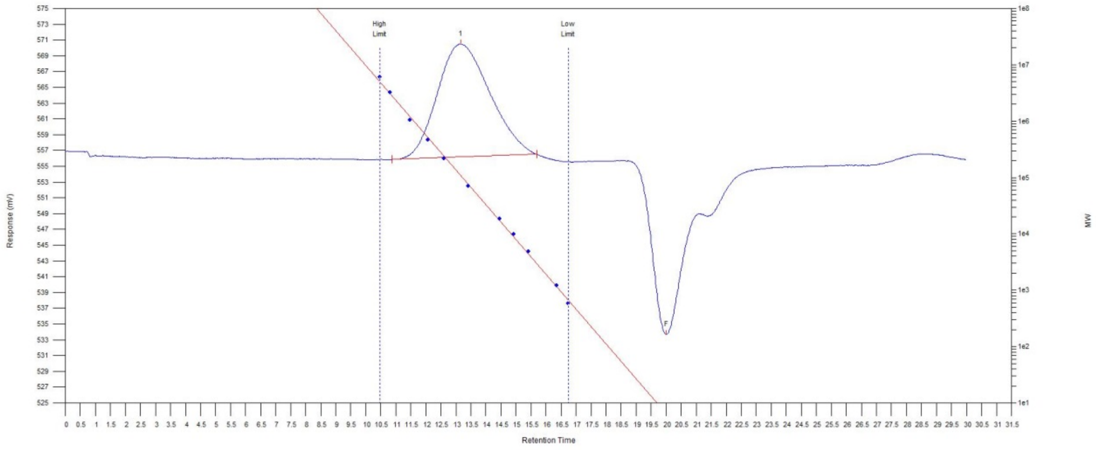

(b)

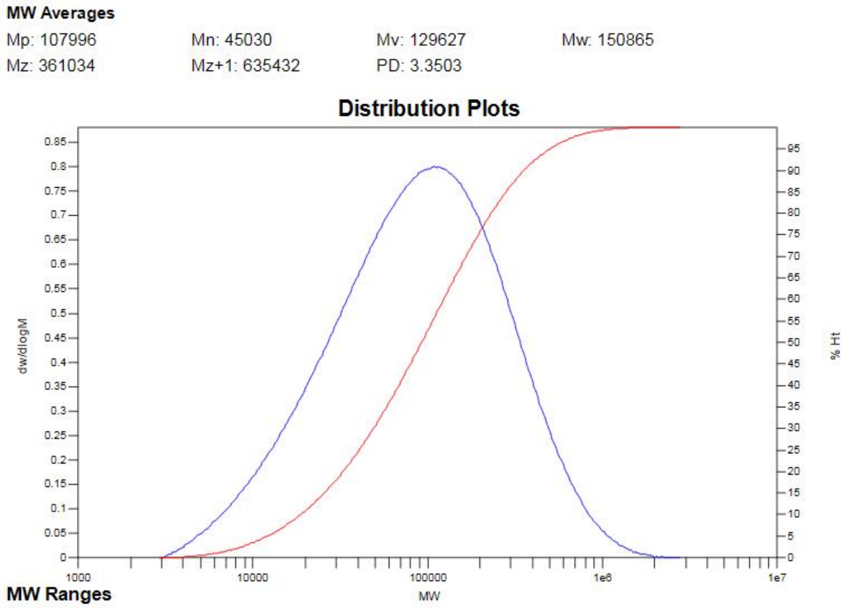

Supplementary Fig. 108. Gel Permeation Chromatography (GPC) trace of **P5** chlorobenzene fraction. (a) Trace data from refractive index (RI) detector. (b) Molecular weight distribution plots of **P5** chlorobenzene fraction **(Table 3, Entry 5)**.

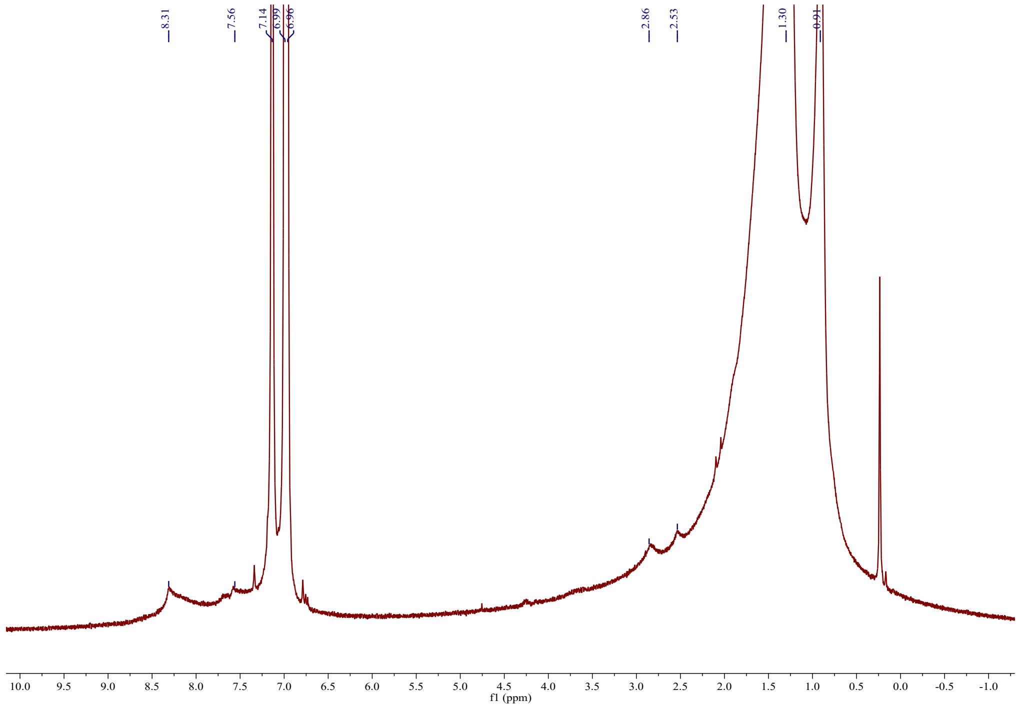

Supplementary Fig. 109. ^{1}H NMR spectrum of **P5** chlorobenzene fraction in C_6D_5Cl at 298 K

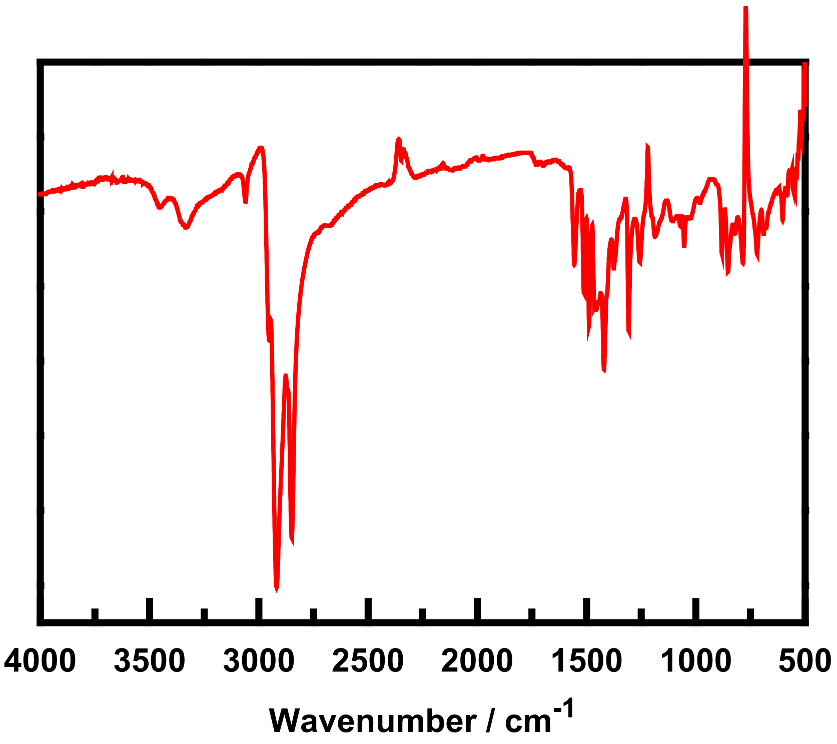

Supplementary Fig. 110. FT-IR spectrum of compound **P5** chloroform fraction in thin film at 298 K

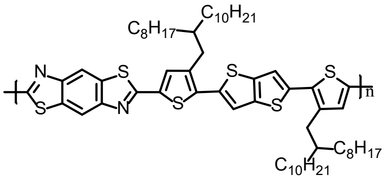

(a)

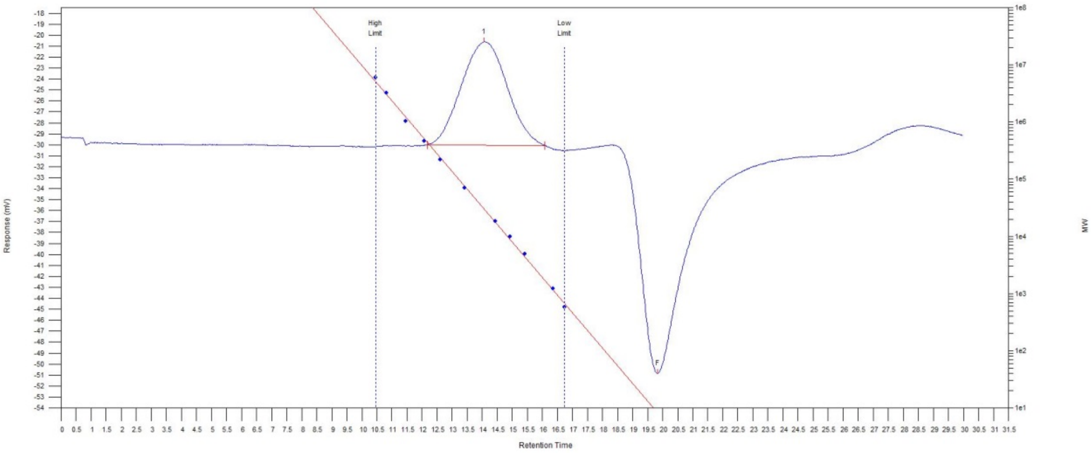

(b)

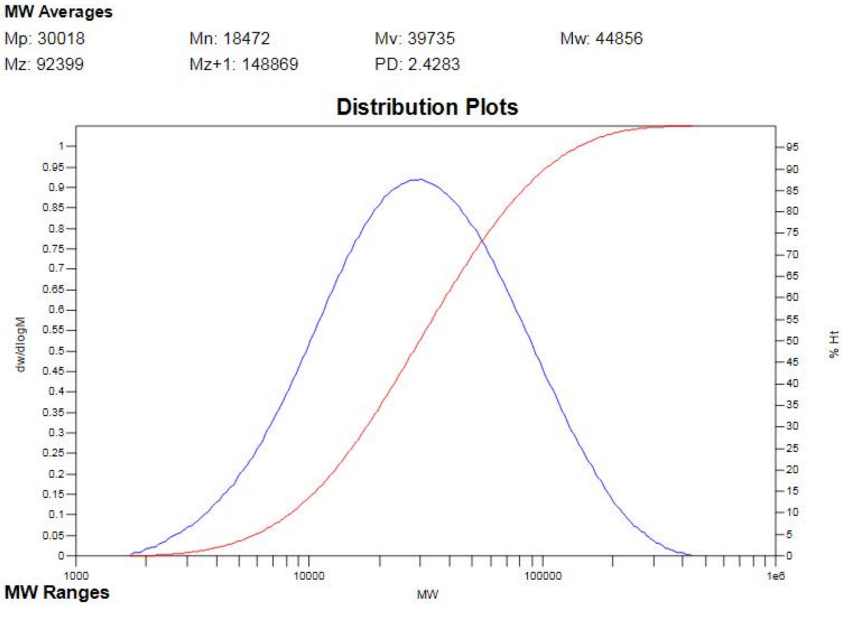

Supplementary Fig. 111. Gel Permeation Chromatography (GPC) trace of **P6**. (a) Trace data from refractive index (RI) detector. (b) Molecular weight distribution plots of **P6 (Table 3, Entry 6)**.

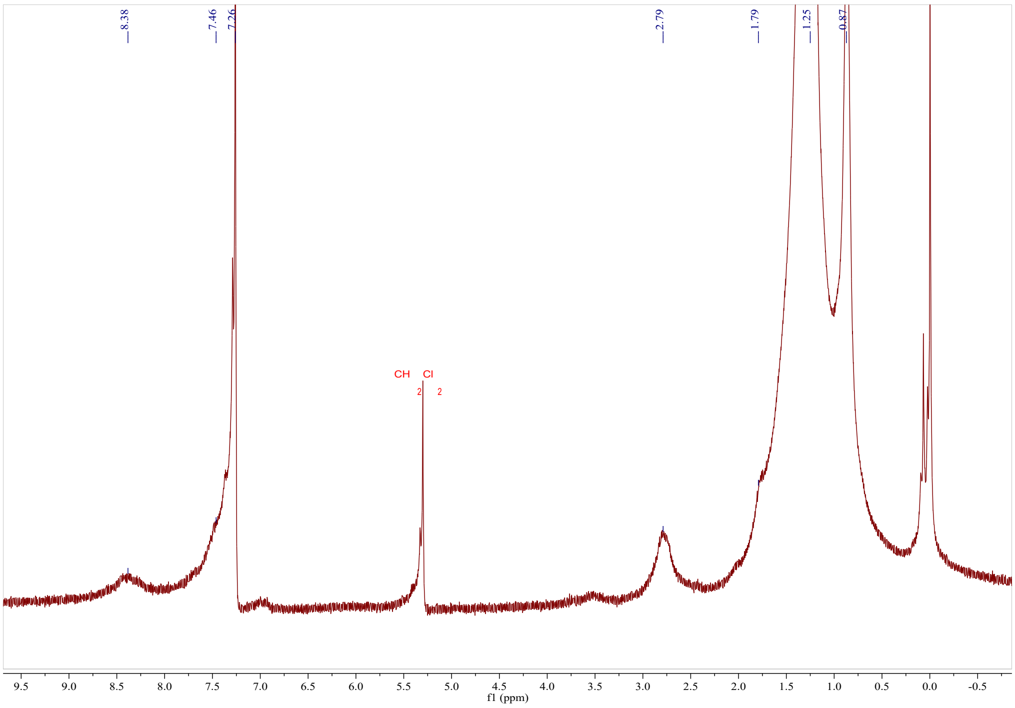

Supplementary Fig. 112. ^{1}H NMR spectrum of **P6** in C_6D_5Cl at 298 K.

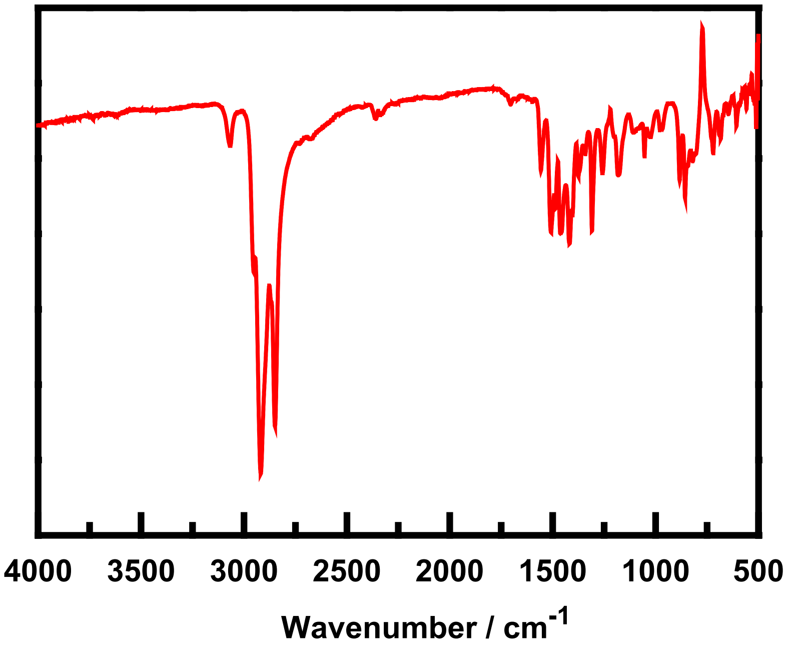

Supplementary Fig. 113. FT-IR spectrum of compound **P6** in thin film at 298 K.

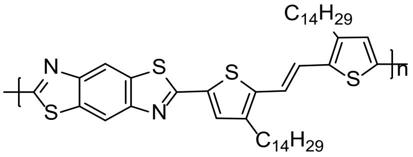

Chloroform fraction:

(a)

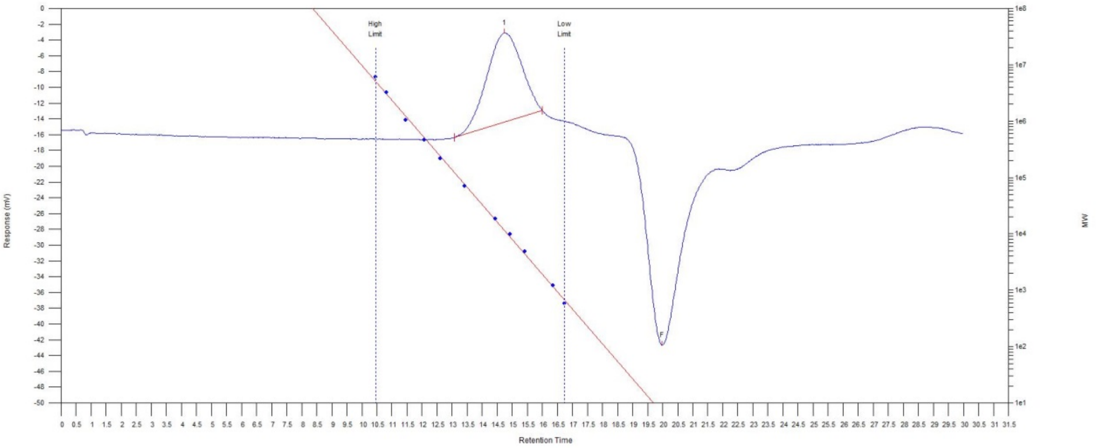

(b)

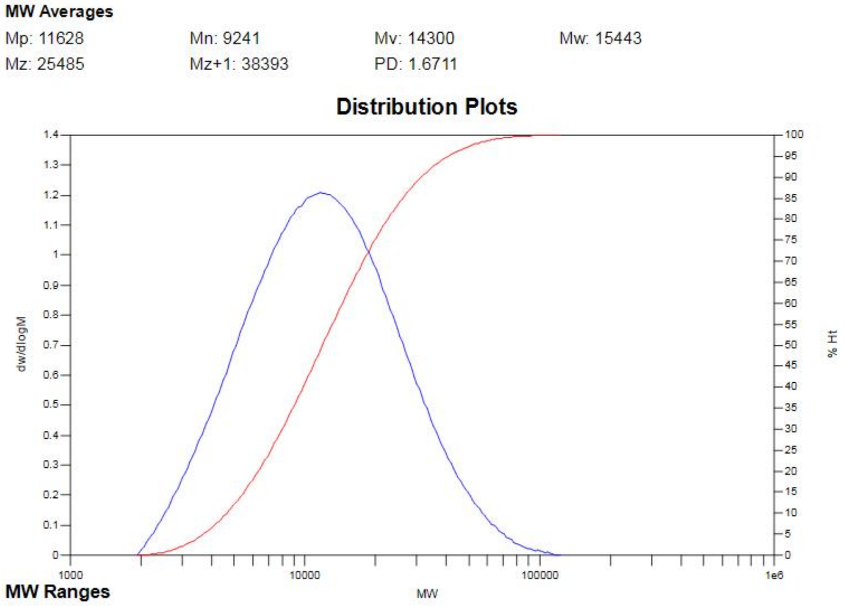

Supplementary Fig. 114. Gel Permeation Chromatography (GPC) trace of **P7** chloroform fraction. (a) Trace data from refractive index (RI) detector. (b) Molecular weight distribution plots of **P7** chloroform fraction **(Table 3, Entry 7)**.

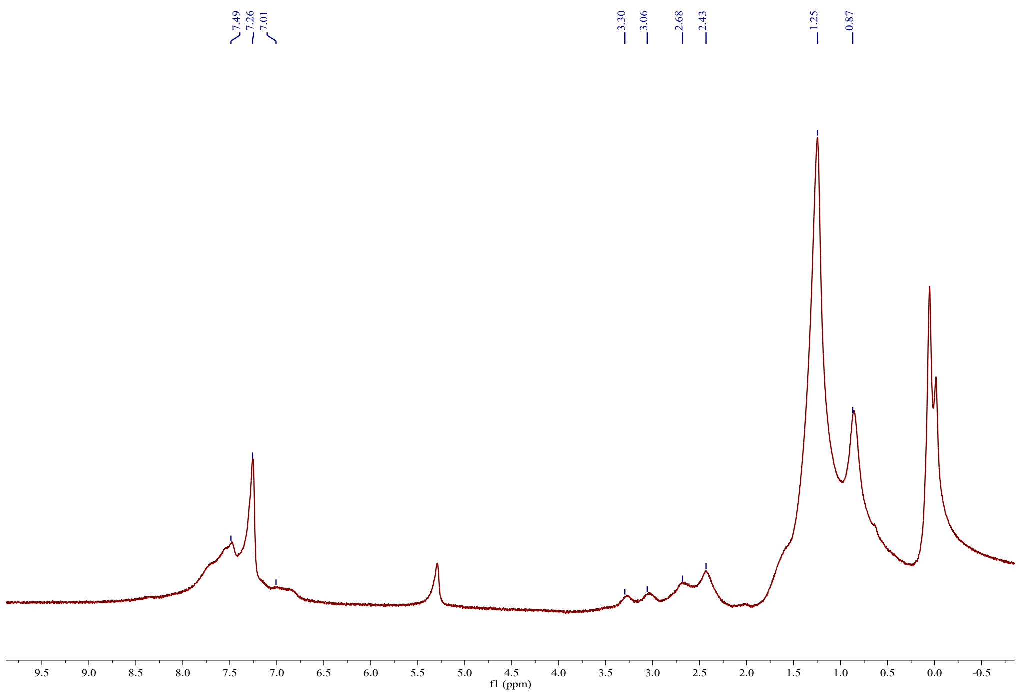

Supplementary Fig. 115. ^{1}H NMR spectrum of **P7** chloroform fraction in $CDCl_3$ at 298 K.

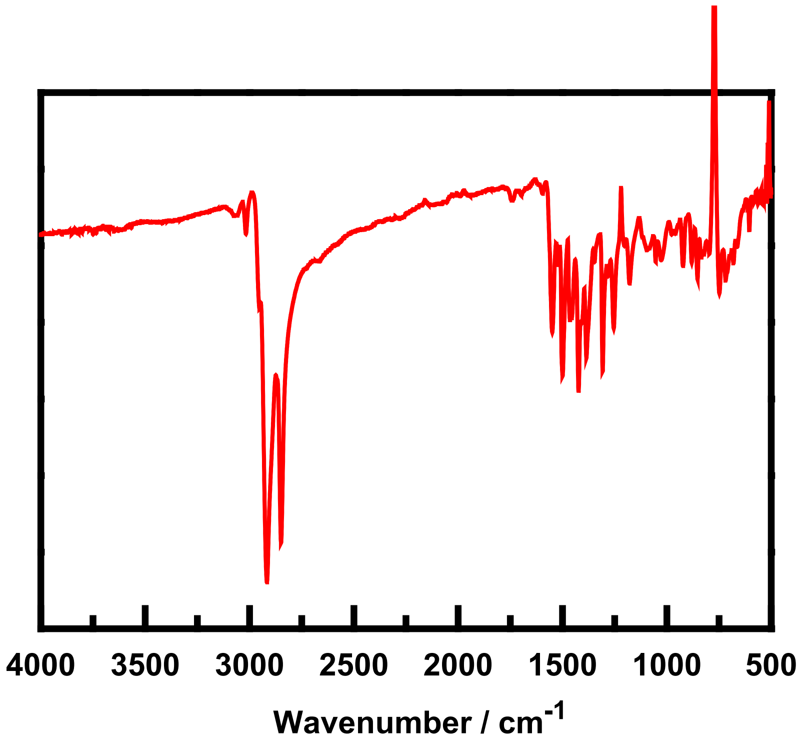

Supplementary Fig. 116. FT-IR spectrum of compound **P7** chloroform fraction in thin film at 298 K.

Chlorobenzene fraction:

(a)

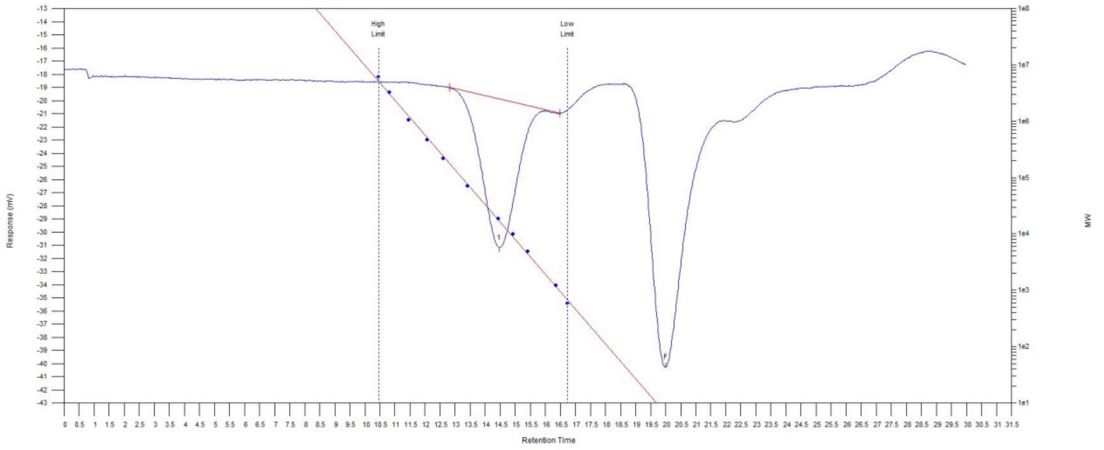

(b)

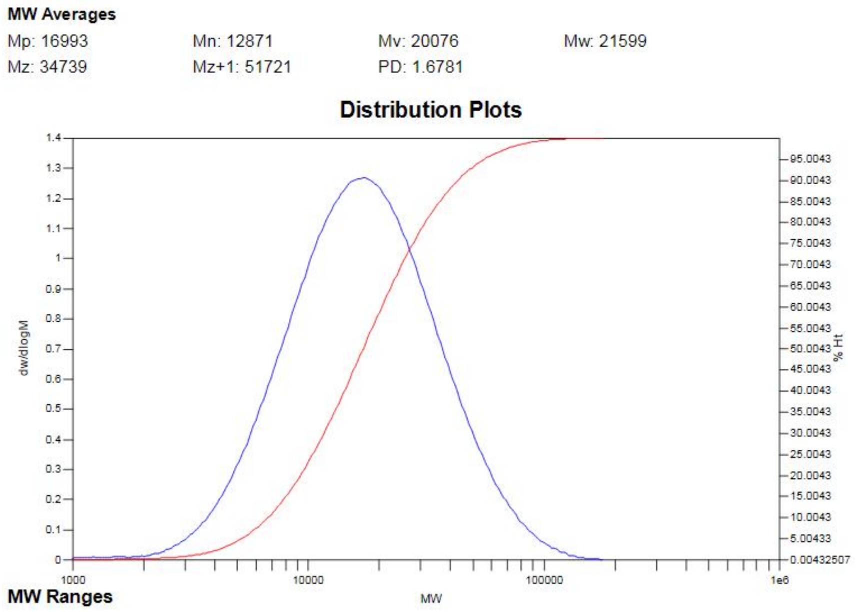

Supplementary Fig. 117. Gel Permeation Chromatography (GPC) trace of **P7** chlorobenzene fraction. (a) Trace data from refractive index (RI) detector. (b) Molecular weight distribution plots of **P7** chlorobenzene fraction **(Table 3, Entry 7)**.

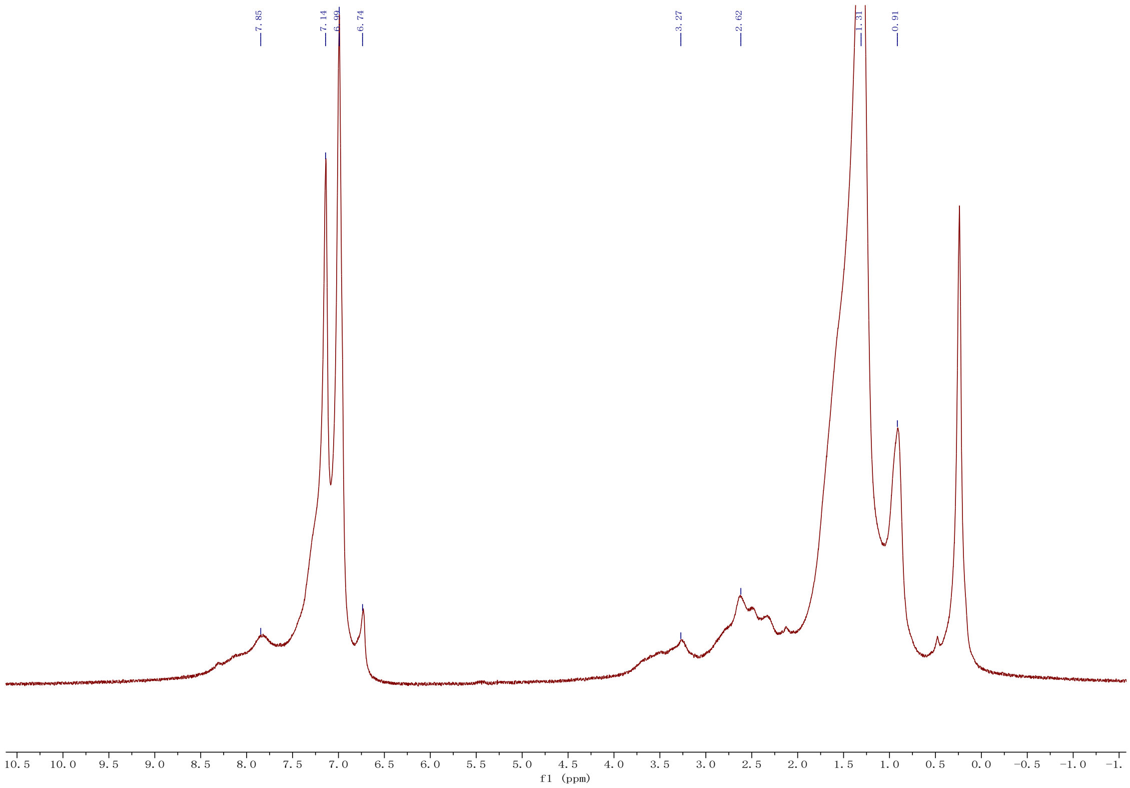

Supplementary Fig. 118. ^{1}H NMR spectrum of **P7** chlorobenzene fraction in C_6D_5Cl at 298 K.

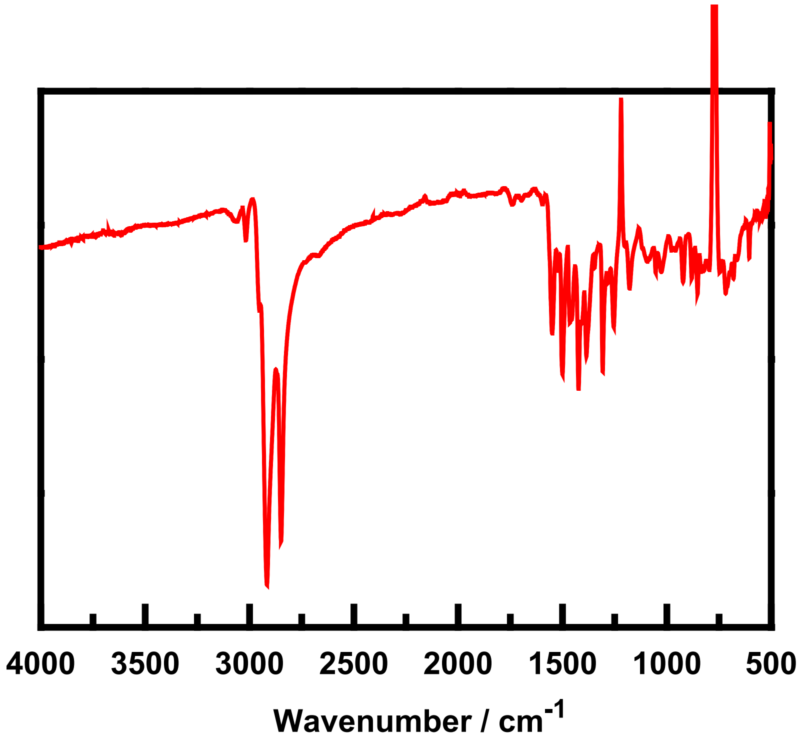

Supplementary Fig. 119. FT-IR spectrum of compound **P7** chlorobenzene fraction in thin film at 298 K.

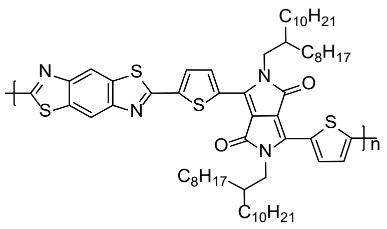

(a)

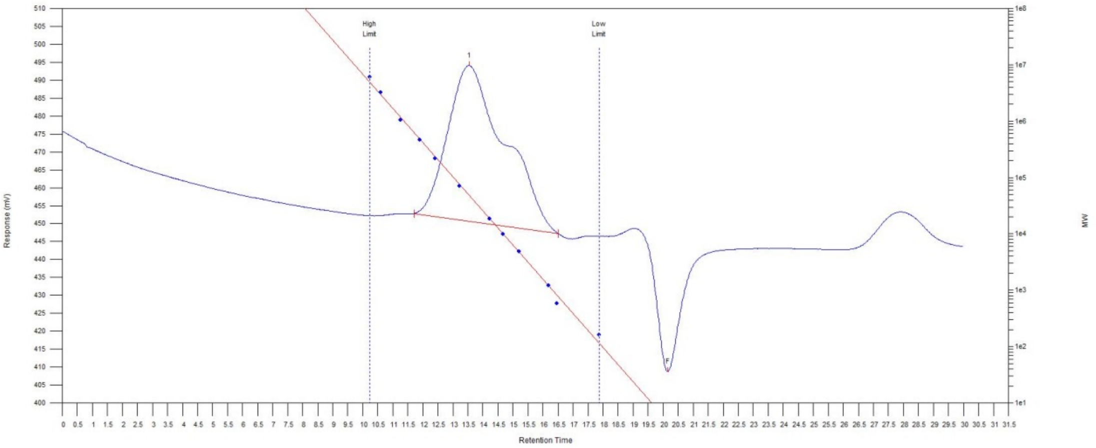

(b)

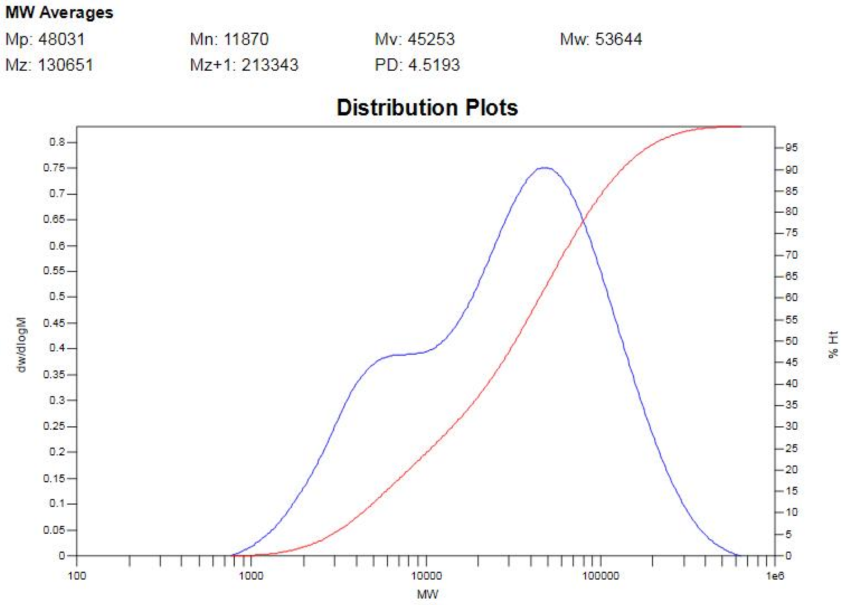

Supplementary Fig. 120. Gel Permeation Chromatography (GPC) trace of **P8**. (a) Trace data from refractive index (RI) detector. (b) Molecular weight distribution plots of **P8 (Table 3, Entry 8)**.

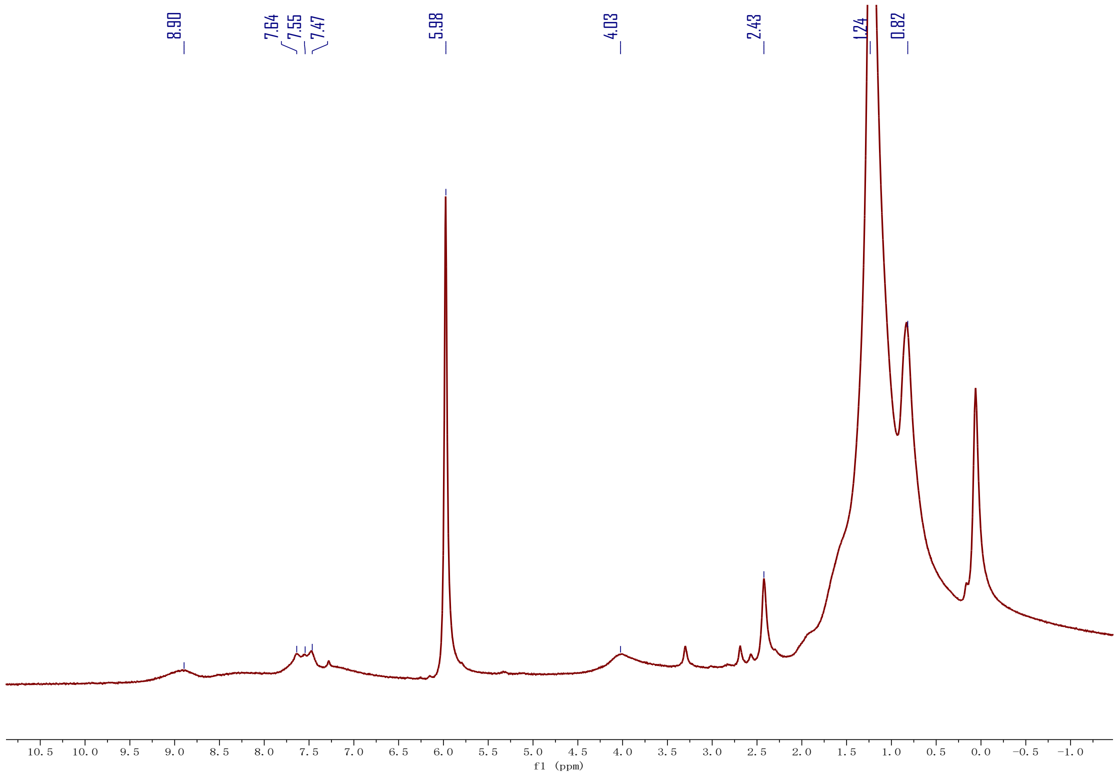

Supplementary Fig. 121. ^{1}H NMR spectrum of **P8** in $C_2D_2Cl_4$ at 298 K.

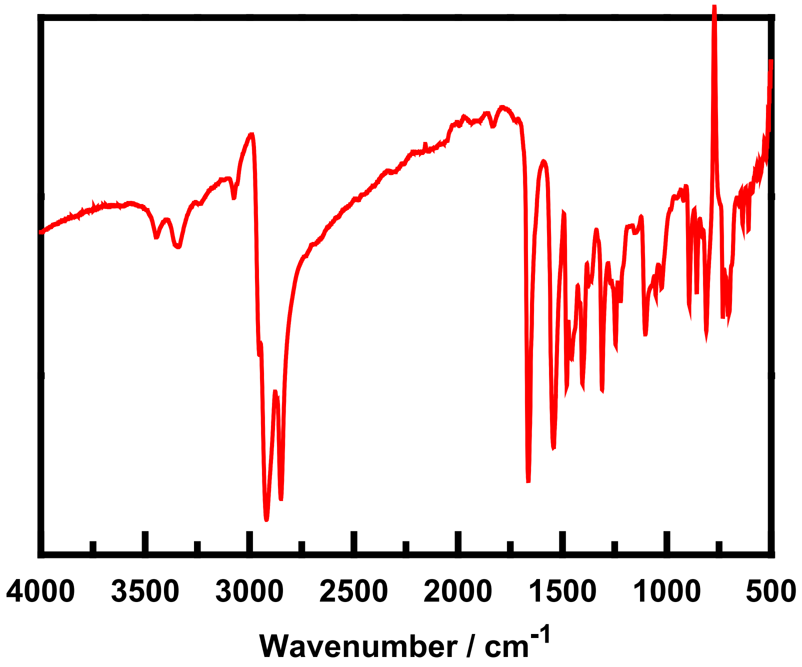

Supplementary Fig. 122. FT-IR spectrum of compound **P8** in thin film at 298 K.

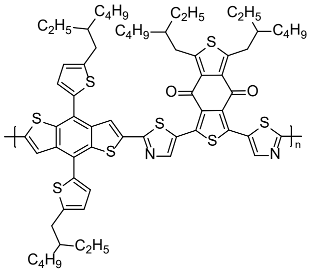

Chloroform fraction:

(a)

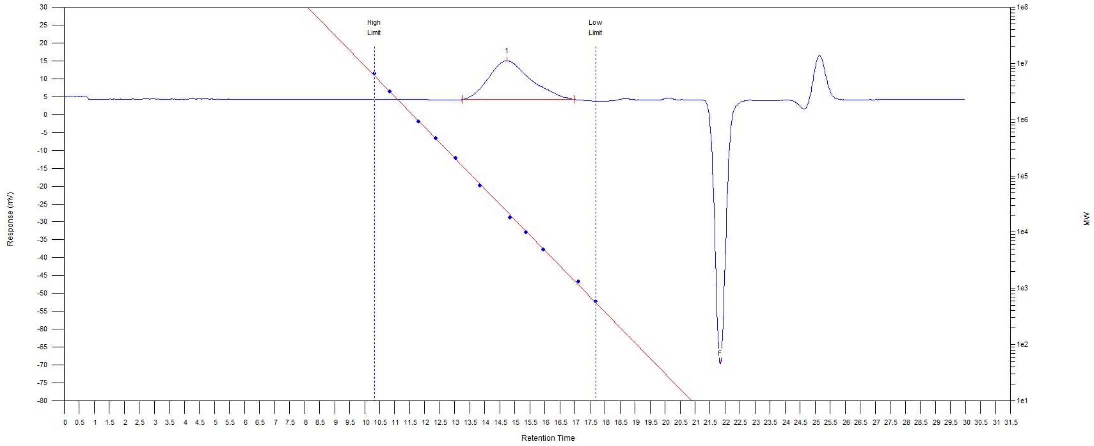

(b)

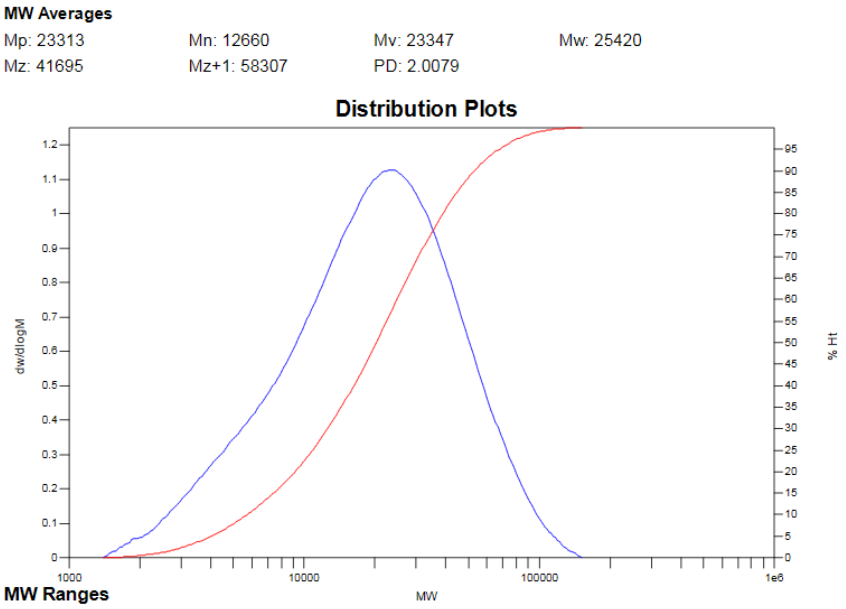

Supplementary Fig. 123. Gel Permeation Chromatography (GPC) trace of **P9** chloroform fraction. (a) Trace data from refractive index (RI) detector. (b) Molecular weight distribution plots of **P9** chloroform fraction **(Table 3, Entry 9)**.

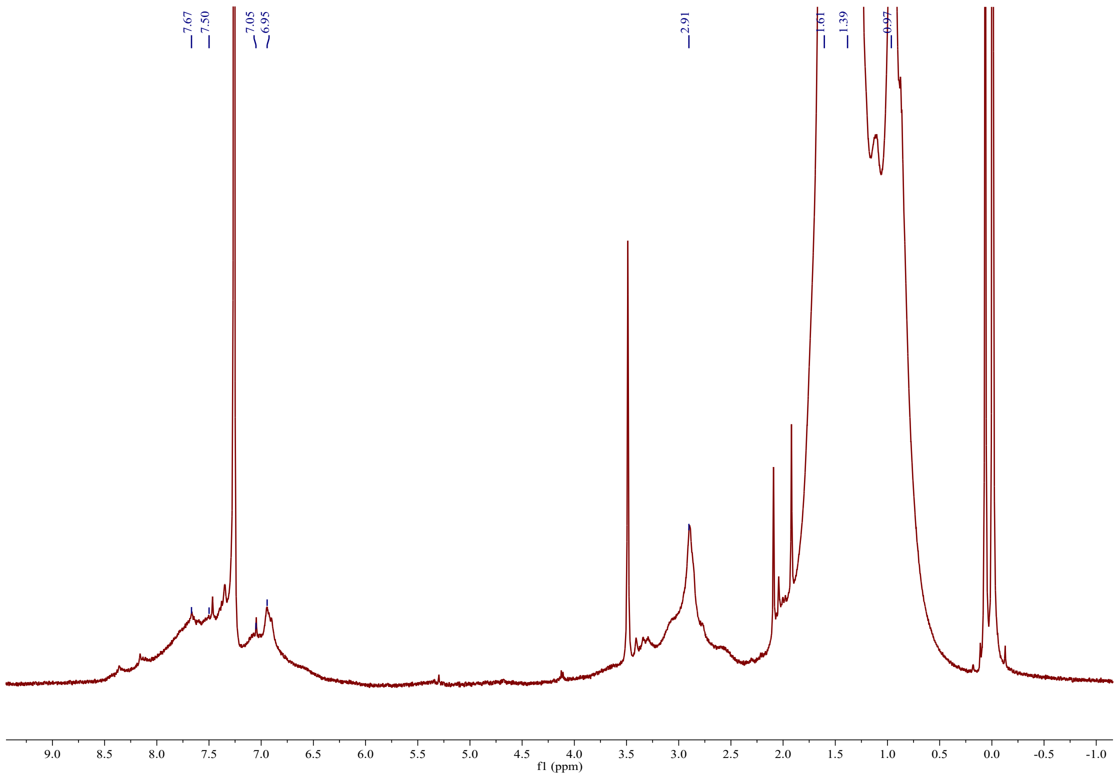

Supplementary Fig. 124. ^{1}H NMR spectrum of **P9** chloroform fraction in $CDCl_3$ at 298 K.

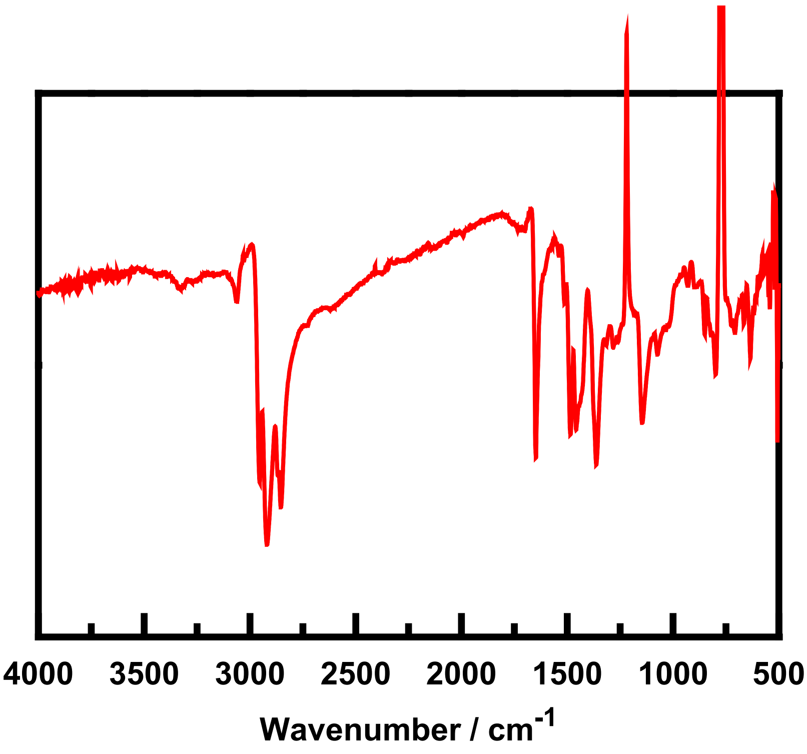

Supplementary Fig. 125. FT-IR spectrum of compound **P9** chloroform fraction in thin film at 298 K.

Chlorobenzene fraction:

(a)

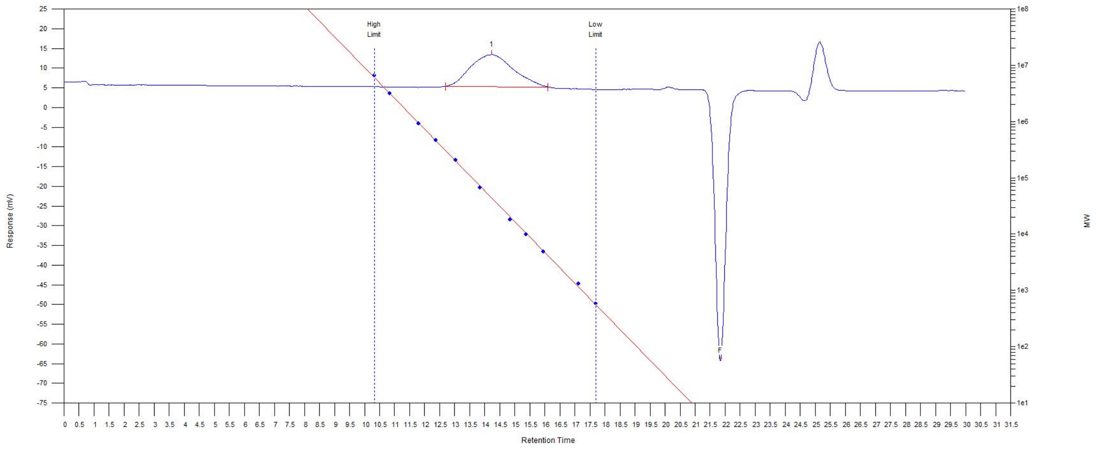

(b)

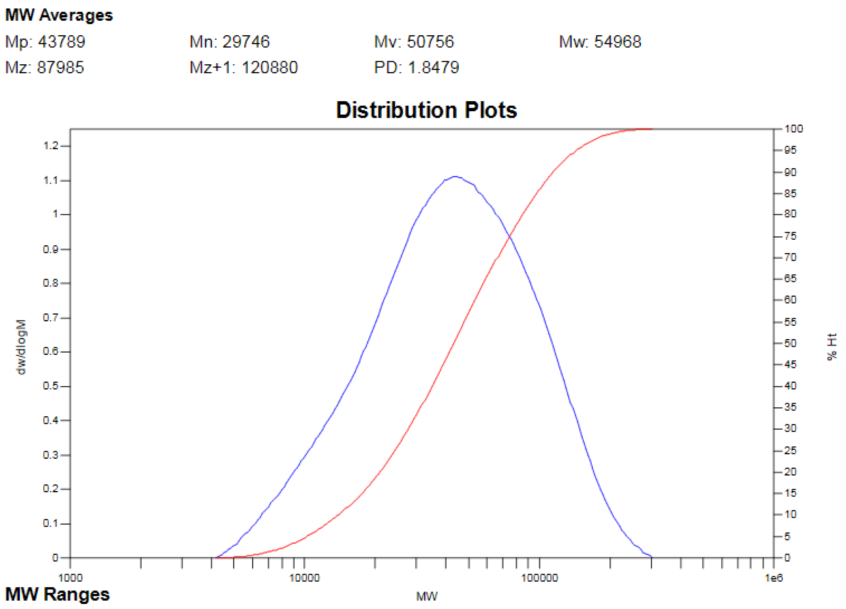

Supplementary Fig. 126. Gel Permeation Chromatography (GPC) trace of **P9** chlorobenzene fraction. (a) Trace data from refractive index (RI) detector. (b) Molecular weight distribution plots of **P9** chlorobenzene fraction **(Table 3, Entry 9)**.

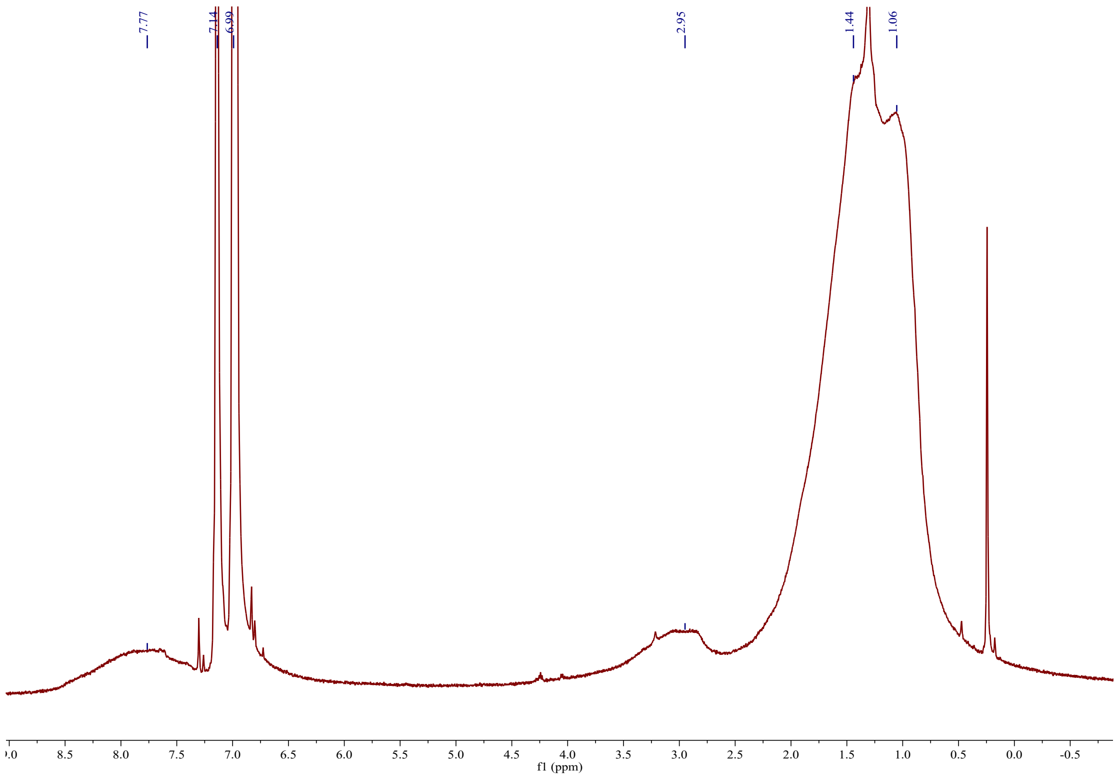

Supplementary Fig. 127. ^{1}H NMR spectrum of **P9** chlorobenzene fraction in C_6D_5Cl at 298 K.

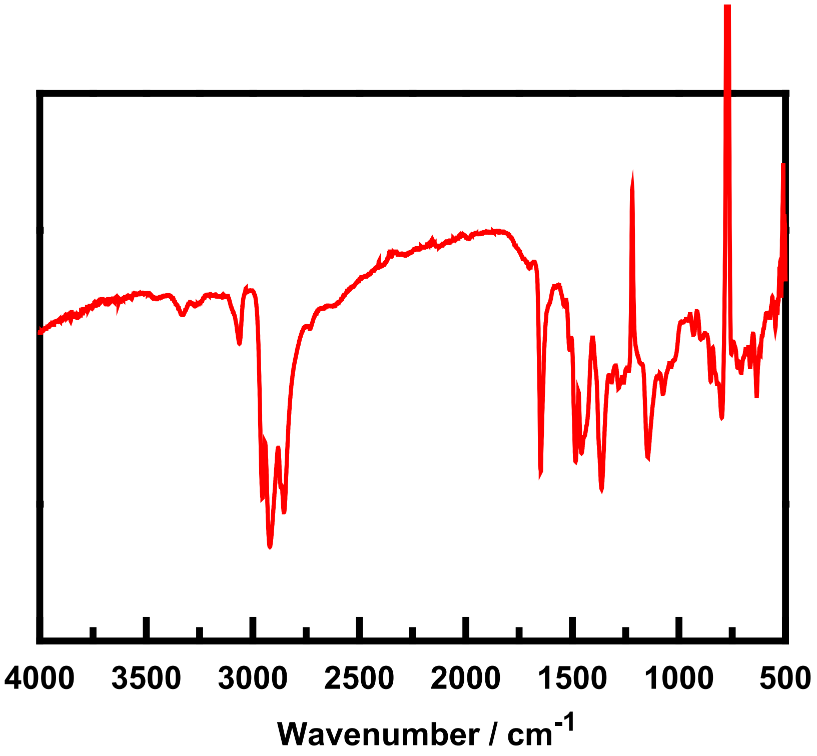

Supplementary Fig. 128. FT-IR spectrum of compound **P9** chlorobenzene fraction in thin film at 298 K.

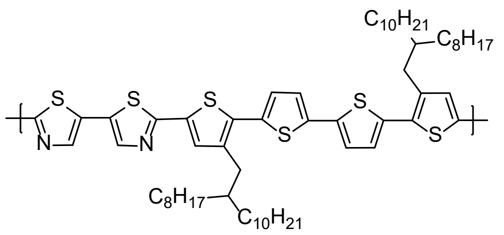

(a)

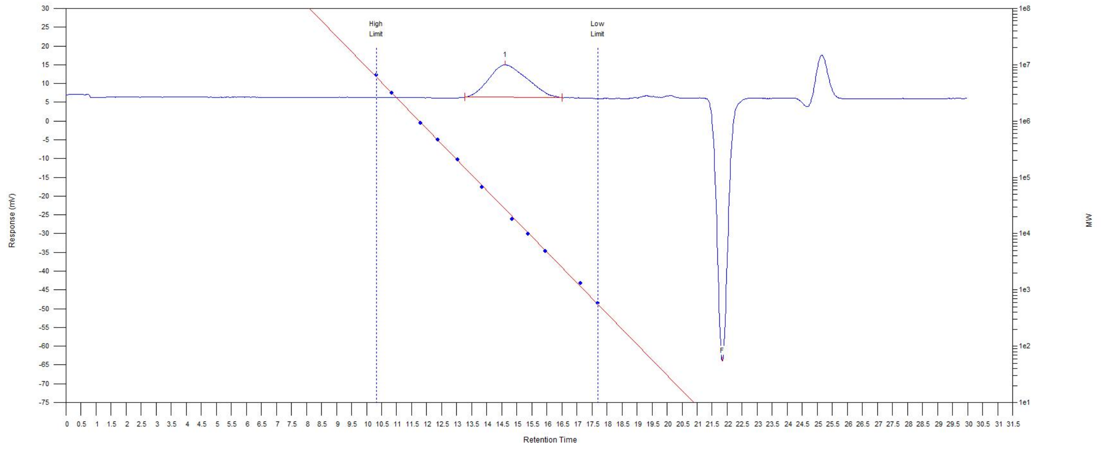

(b)

MW Averages

Mp: 27581	Mn: 17225	Mv: 26939	Mw: 28836
Mz: 43537	Mz+1: 58684	PD: 1.6741	

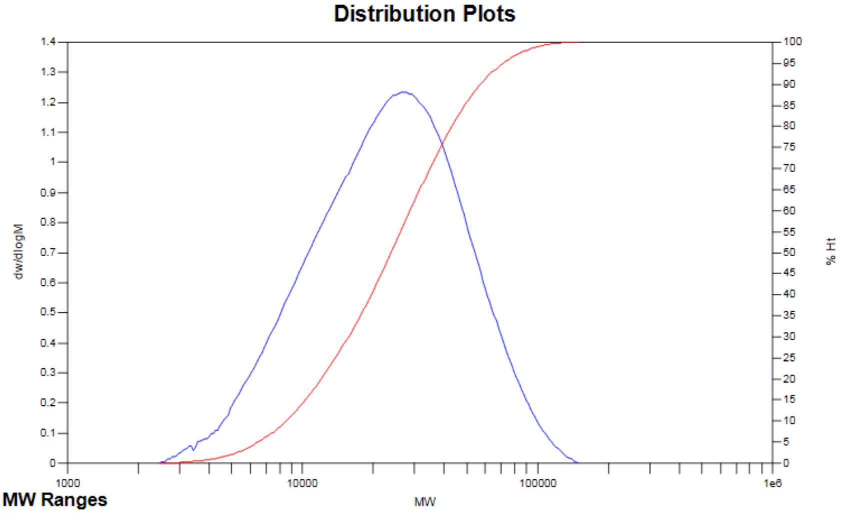

MW Ranges

Supplementary Fig. 129. Gel Permeation Chromatography (GPC) trace of **P10** (a) Trace data from refractive index (RI) detector. (b) Molecular weight distribution plots of **P10 (Table 3, Entry 10)**.

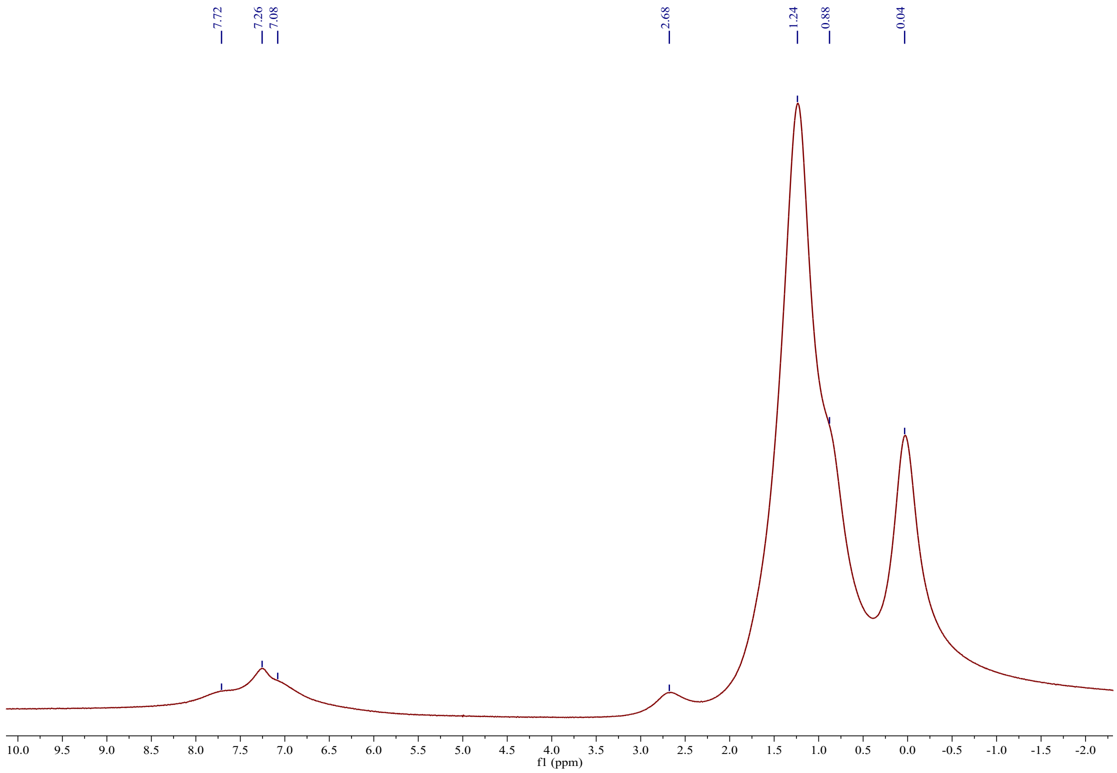

Supplementary Fig. 130. ^{1}H NMR spectrum of **P10** in $CDCl_3$ at 298 K.

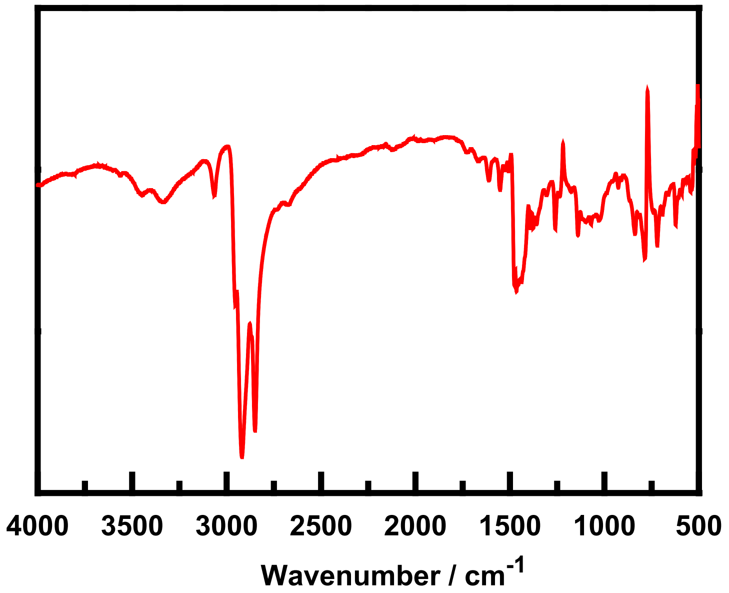

Supplementary Fig. 131. FT-IR spectrum of compound **P10** in thin film at 298 K.

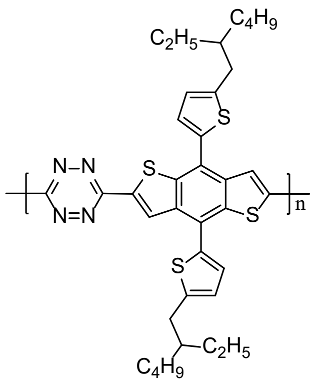

(a)

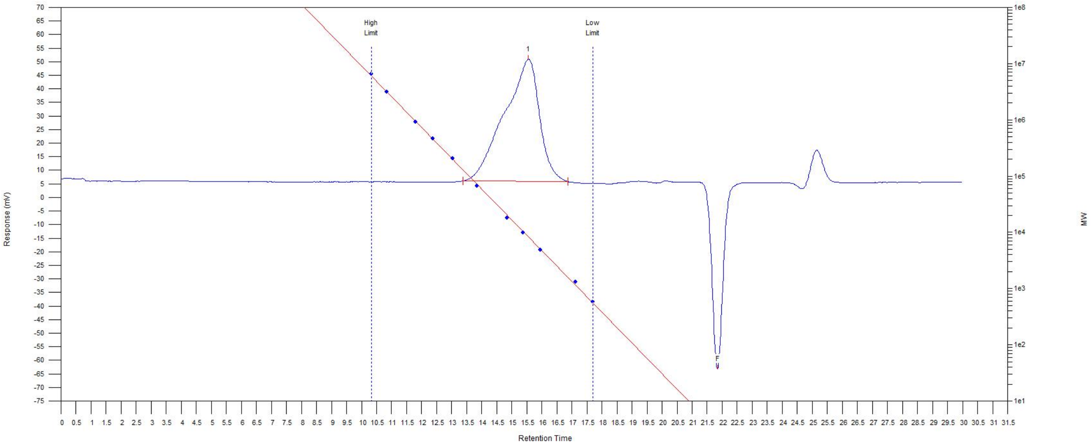

(b)

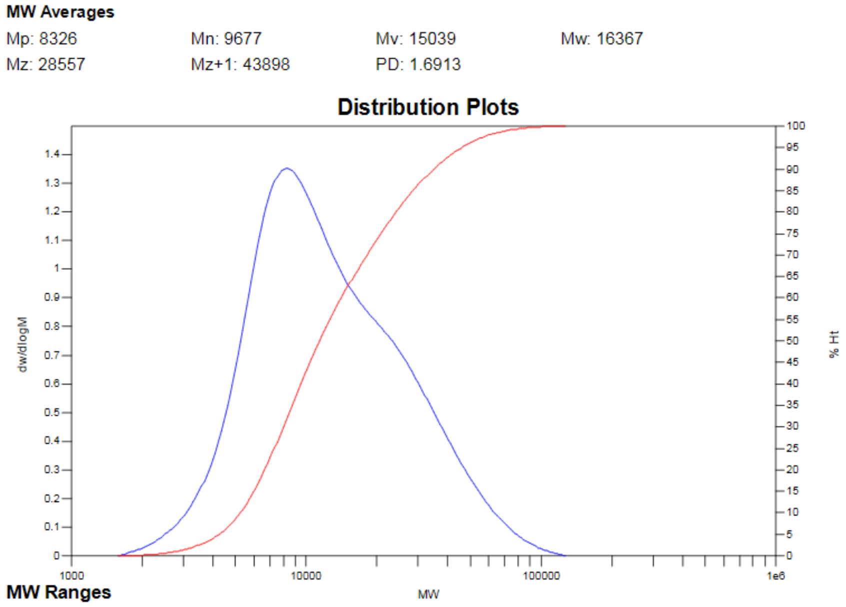

Supplementary Fig. 132. Gel Permeation Chromatography (GPC) trace of **P11** (a) Trace data from refractive index (RI) detector. (b) Molecular weight distribution plots of **P11 (Table 3, Entry 11)**.

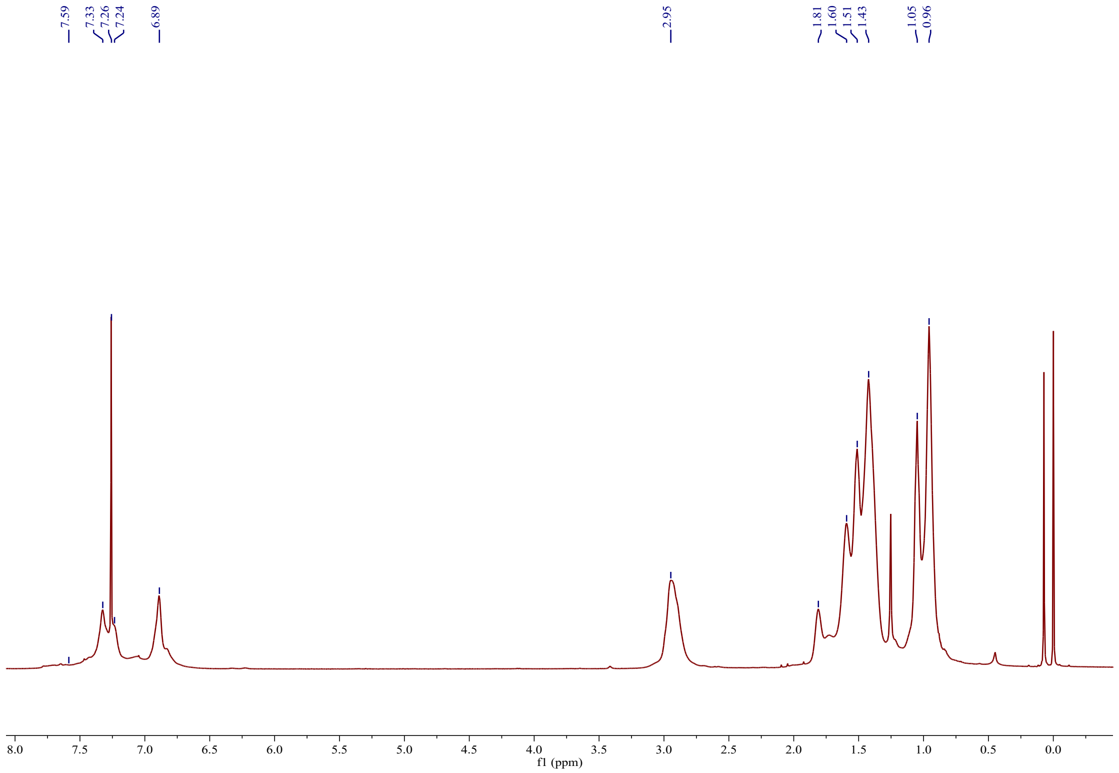

Supplementary Fig. 133. ^{1}H NMR spectrum of **P11** in $CDCl_3$ at 298 K.

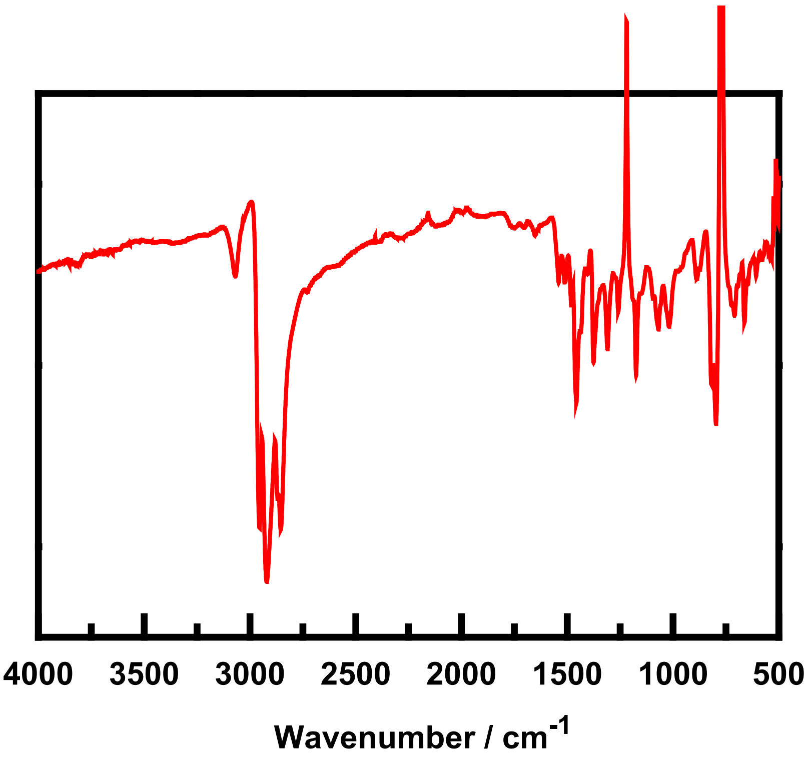

Supplementary Fig. 134. FT-IR spectrum of compound **P11** in thin film at 298 K.
